# Synthesis and optical properties of bis- and tris-alkynyl-2-trifluoromethylquinolines

**DOI:** 10.3762/bjoc.20.107

**Published:** 2024-05-29

**Authors:** Stefan Jopp, Franziska Spruner von Mertz, Peter Ehlers, Alexander Villinger, Peter Langer

**Affiliations:** 1 Universität Rostock, Institut für Chemie, Albert-Einstein-Str. 3a, 18059 Rostock, Germany; 2 Leibniz-Institut für Katalyse e.V. an der Universität Rostock, Albert-Einstein-Str. 29a, 18059 Rostock, Germany

**Keywords:** alkynes, catalysis, fluorescence, heterocycles, palladium

## Abstract

Three bis- or tris-brominated 2-trifluoromethylquinolines have been successfully applied in palladium-catalysed Sonogashira reactions, leading to several examples of alkynylated quinolines in good to excellent yields. Optical properties of selected products have been studied by steady state absorption and fluorescence spectroscopy which give insights of the influence of the substitution pattern and of the type of substituents on the optical properties.

## Introduction

**Figure 1:**
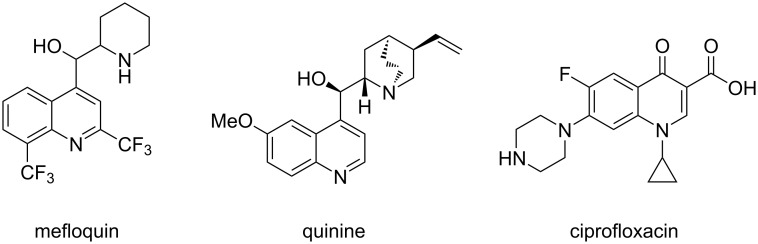
Natural and synthetic compounds containing a quinoline or quinolone core-structure.

Quinoline is a well-known core structure which can be found in several natural and synthetic products and many of them show interesting pharmacological properties [1–3]. Quinine, for example, is a widely known natural product which was first isolated from the cinchona tree besides many other quinoline-containing cinchona alkaloids [4]. It is applied as antimalarial agent and furthermore as a bitter flavour component. Mefloquin [5] and ciprofloxacin [6], on the other hand, are synthetic compounds containing a fluorinated quinoline and quinolone core structure and are used as antimalarial and antibacterial agents, respectively (Figure 1). Fluorine-containing quinolines and quinolinones are of particular interest, since fluorine atoms are known to enhance the pharmacological properties of organic molecules [7–15]. Fluorine atoms act as bioisosteres of hydrogen atoms, while CF_3_ groups are bioisosteres of hydroxy and methyl groups and are known to protect from metabolic oxidation [16]. Fluorine-containing arenes are metabolically more stable as compared to non-fluorinated arenes and they show a higher lipophilicity.

Known synthetic approaches towards 2-trifluoromethylquinolines include the cyclisation of anilines with trifluoroacetoacetate [17], the synthesis from enaminoketones [18] and the gold-catalysed cyclisation of propargylanilines [19].

Aside from its pharmaceutical significance quinoline compounds have been reported as strongly fluorescent with applications as electroluminescence materials [20–23], dyes, preservatives and as ligands in complex chemistry [24–27].

In the context of our interest in the application of cross-coupling reactions to polyhalogenated heterocycles [28–31], we studied Sonogashira reactions of brominated 2-trifluoromethylquinolines. The optical properties of selected products, bis- and tris-alkynylated quinolines, were studied to gain insights into how the substitution pattern at different positions may alter the optical properties.

## Results and Discussion

**Scheme 1:**
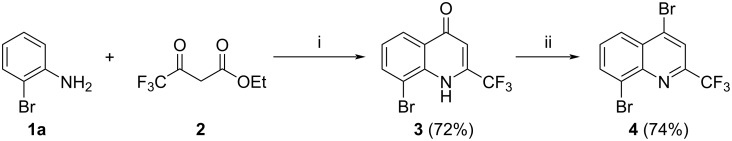
Synthesis of **4**. Reaction conditions: i: polyphosphoric acid, 150 °C, 2 h; ii: POBr_3_ (1.1 equiv), 150 °C, 2 h.

The starting material, 4,8-dibromo-2-(trifluoromethyl)quinoline (**4**), was synthesized from 2-bromoaniline (**1a**) and ethyl trifluoroacetoacetate (**2**), adapting a known procedure from Schlosser and Marull (Scheme 1) [17]. The cyclization of **1a** with **2a** chemoselectively afforded 8-bromo-2-trifluoromethyl-4-quinolone (**3**) rather than 8-bromo-4-trifluoromethyl-2-quinolone. Bromination of **3** with phosphoryl bromide gave **4**.

With quinoline **4** in hand, we studied palladium-catalysed Sonogashira reactions with phenylacetylene (**5a**). Gratifyingly, our initial test reaction, using Pd(OAc)_2_ as catalyst with XPhos as ligand, gave bis-alkynylated product **6a** in quantitative yield. Reducing the catalyst loading from 5 to 2.5 mol % or switching to more simple Pd(PPh_3_)_4_ still achieved quantitative yields. Less catalyst led to a reduced yield (Table 1). Consequently, we chose 2.5 mol % Pd(PPh_3_)_4_ for all further reactions.

**Table 1:** Optimization of the Sonogashira reaction.^a^

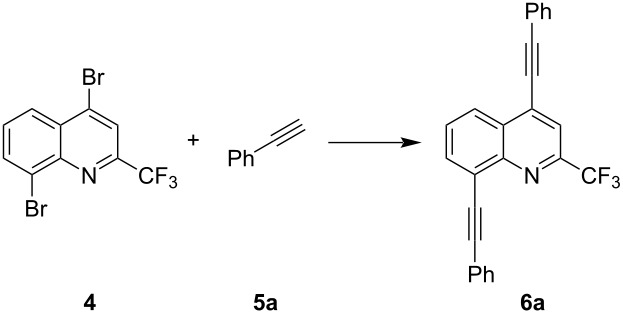			

Pd-catalyst (mol %)	Ligand (mol %)	Cu-catalyst (mol %)	Yield (%)

Pd(OAc)_2_ (5)	XPhos (10)	CuI (10)	99
Pd(OAc)_2_ (2.5)	XPhos (5)	CuI (5)	99
Pd(PPh_3_)_4_ (5)	–	CuI (10)	99
Pd(PPh_3_)_4_ (2.5)	–	CuI (5)	99
Pd(PPh_3_)_4_ (1.75)	–	CuI (3.5)	93
Pd(PPh_3_)_4_ (1)	–	CuI (2)	90

^a^Reaction conditions: Pd catalyst, ligand, Cu catalyst, phenylacetylene (3.0 equiv), dioxane, NEt_3_, 100 °C, 6 h.

**Scheme 2:**
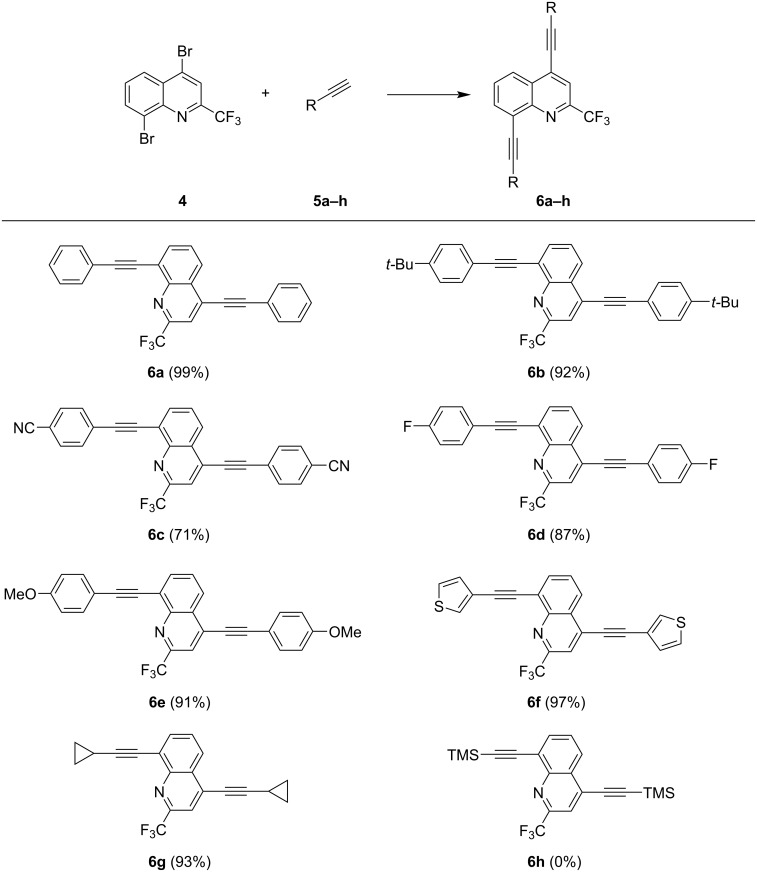
Synthesis of compounds **6a–h**. Reaction conditions: Pd(PPh_3_)_4_ (2.5 mol %), CuI (5 mol %), acetylene (3.0 equiv), dioxane, NEt_3_, 100 °C, 6 h; isolated yields.

As a next step, we analysed the scope of our methodology (Scheme 2). The optimized conditions allow cross-coupling reactions of various acetylenes containing electron-rich and electron-withdrawing functional groups, like methoxy or cyano, as well as thienyl and cyclopropyl groups. In general, all products were achieved in very good yields, ranging from 71 to 99%. Only product **6h**, containing a TMS group, could not be isolated at all, since the reaction resulted in an inseparable mixture of several products.

**Figure 2:**
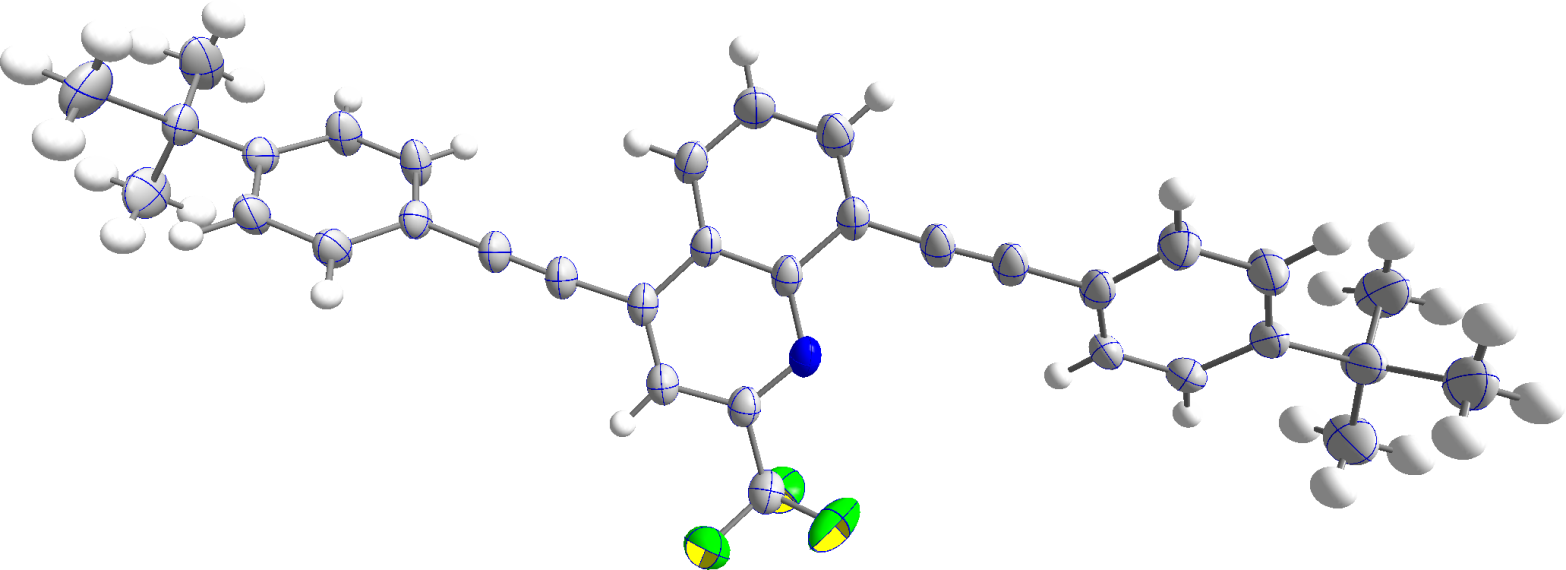
ORTEP of **6b** (CCDC 2322985).

The structure of **6b** could be independently confirmed by X-ray crystallography (Figure 2). Both phenyl rings are found to be twisted in an angle of approximately 45° from the quinoline core.

**Scheme 3:**
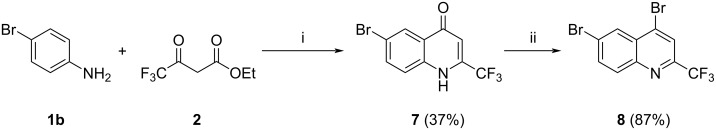
Synthesis of **8**. Reaction conditions: i: polyphosphoric acid, 150 °C, 2 h [33]; ii: POBr_3_ (1.1 equiv), 150 °C, 2 h.

**Scheme 4:**
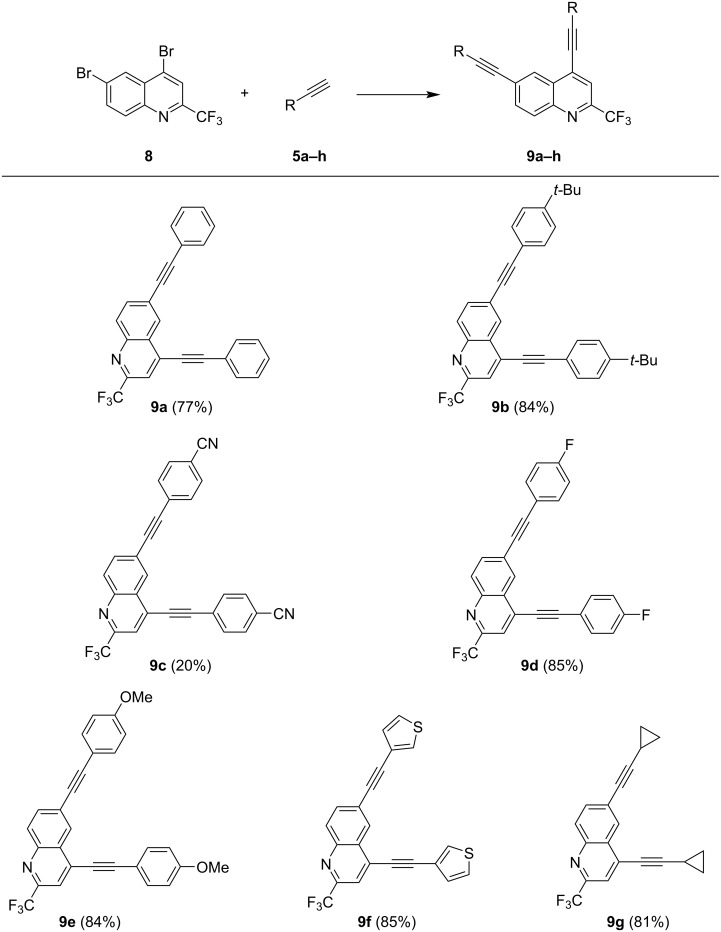
Synthesis of compounds **9a–g**: Reaction conditions: Pd(PPh_3_)_4_ (2.5 mol %), CuI (5 mol %), acetylene (3.0 equiv), dioxane, NEt_3_, 100 °C, 6 h, isolated yields.

As a next step of our synthetic studies, we synthesized 4,6-dibromo-2-trifluoromethylquinoline (**8**) [32], an isomer of **4**, using 4-bromoaniline (**1b**) instead of **1a** (Scheme 3). Afterwards, we studied the scope of the twofold Sonogashira reaction, using the same reaction conditions as before (Scheme 4).

All 2,6-bis-alkynylated quinolines **9** were obtained in good yields of 77–85%, except for **9c** containing a cyano group (20%), most likely due to its low solubility. The yields are generally lower as compared to isomeric 2,8-bis-alkynylated products **6a–g**, which might be due to a slightly lower reactivity of the 6-position in comparison to the 8-position.

**Figure 3:**
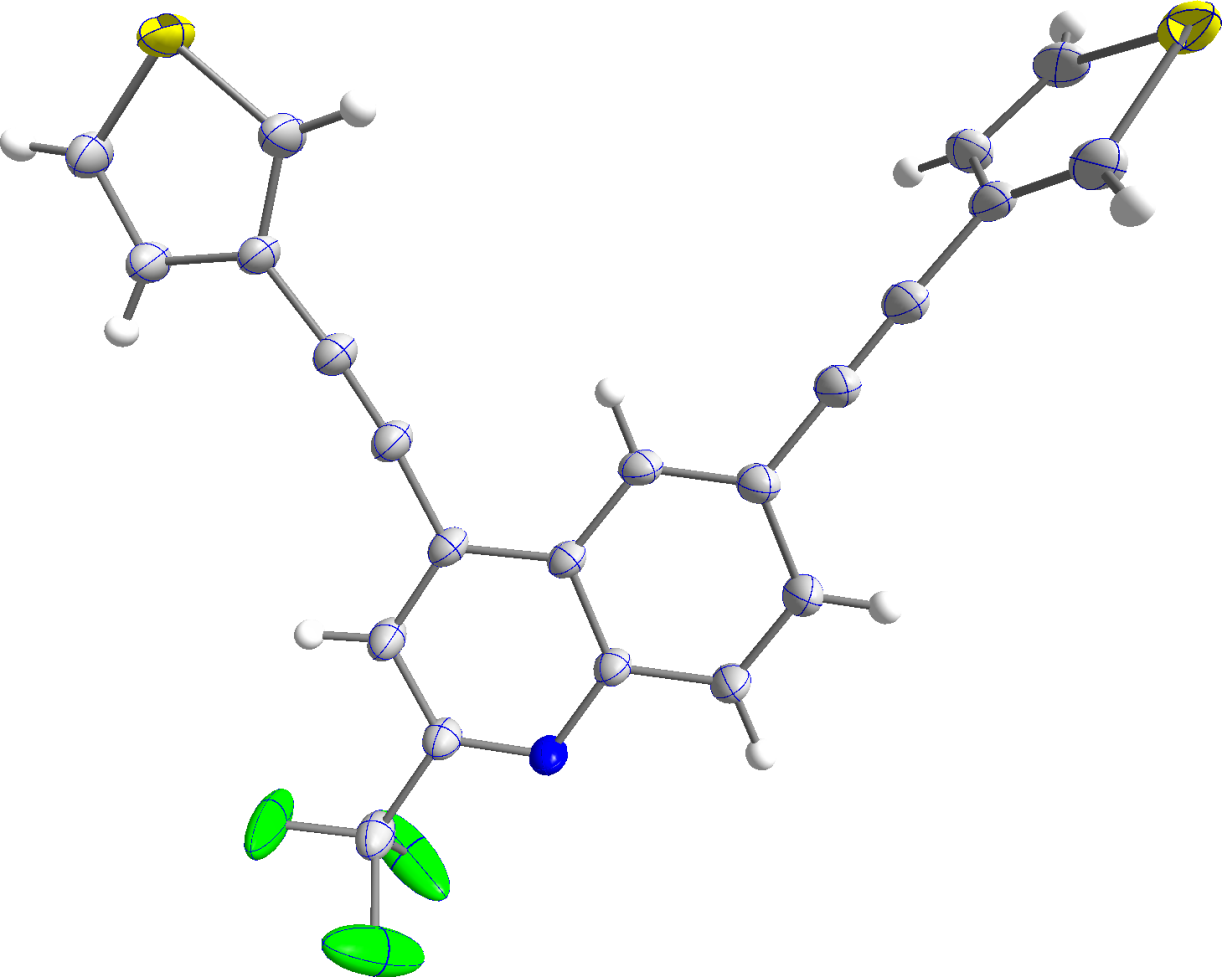
ORTEP of **9f** (CCDC 2322983).

The structure of product **9f** was confirmed by X-ray crystallography (Figure 3). The thiophene ring in 4-position is in plane with the quinoline core, while the other ring is twisted in an angle of approximately 85°. This might be explained by an electronic push–pull interaction of the thiophene and the quinoline moieties via the alkyne.

**Scheme 5:**
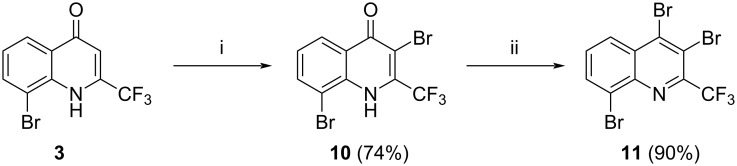
Synthesis of starting material **11**. Reaction conditions: i: AcOH, Br_2_ (1.1 equiv), reflux, 24 h; ii: POBr_3_ (1.1 equiv), 150 °C, 2 h.

Finally, we focussed on the synthesis of tris-alkynylated quinolines starting from 3,4,8-tribromo-2-(trifluoromethyl)quinoline (**11**). Starting material **11** was synthesized in very good yield from quinolone **3** by bromination in position 3, followed by treatment of brominated intermediate **10** with phosphoryl bromide (Scheme 5) [34].

**Scheme 6:**
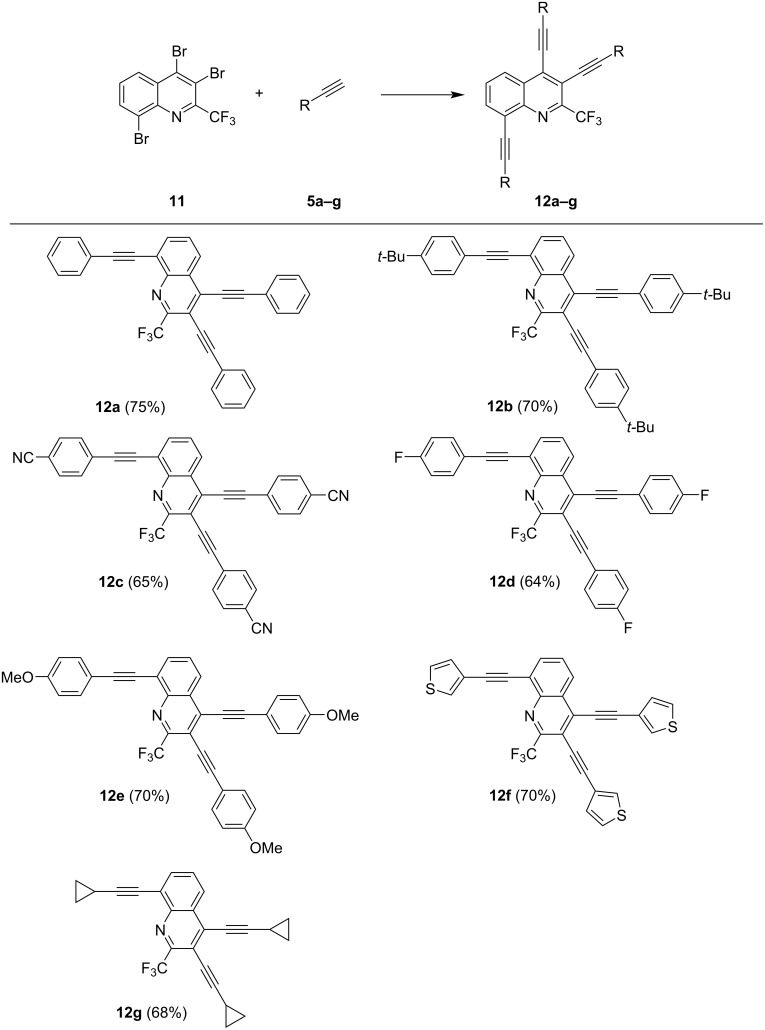
Synthesis of compounds **12a–g**. Reaction conditions: Pd(PPh_3_)_4_ (2.5 mol %), CuI (5 mol %), acetylene (4.0 equiv), dioxane, NEt_3_, 100 °C, 24 h; isolated yields.

**Figure 4:**
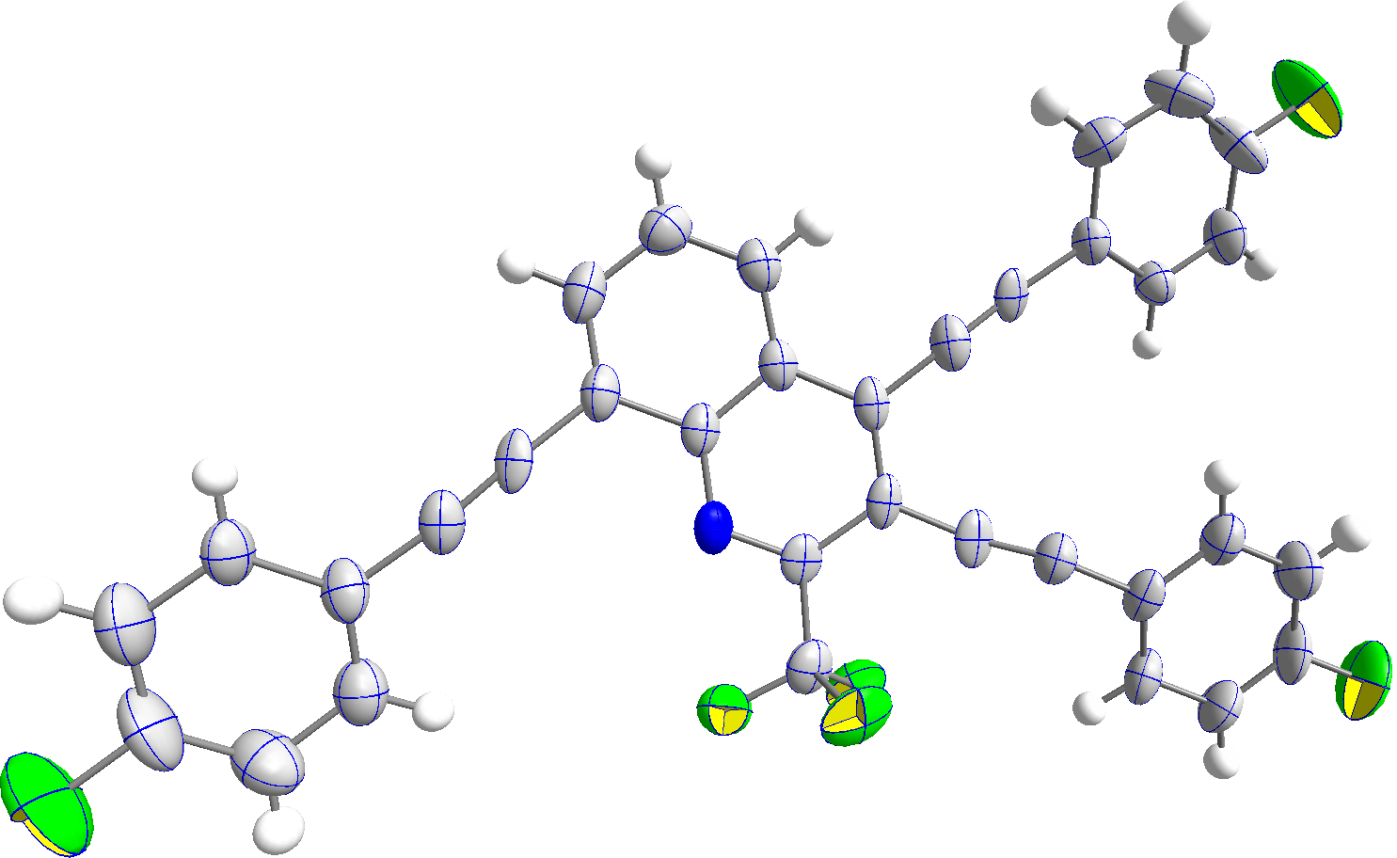
ORTEP of **12d** (CCDC 2322984).

To our delight, the optimized conditions for the synthesis of alkynylated quinolines **6** could be applied also to the three-fold Sonogashira reaction of **11**. However, the reaction time had to be prolonged from 6 to 24 h and the amount of acetylene was increased. Thus, tris-alkynylated quinolines **12a–g** were prepared in good 64–75% yields (Scheme 6). The yields of products **12a–g** were lower as compared to bis-alkynylated products **6a–g** which were, in fact, found as major side-products, due to dehalogenation at position 3. In particular, the side-product **6b** was isolated in 25% yield during the purification of **12b**. The structure of product **12d** was proven by X-ray crystallography (Figure 4). All three phenyl rings are found to be nearly in plane with the quinoline core, showing only a slight twist of 5–10°.

### Optical properties

Steady-state absorption and fluorescence measurements were carried out. Compounds **6a**, **9a**, and **12a** were selected to study the impact of the position of the alkynyl group on the optical properties. Comparison of the absorption and emission features of **12a**, **12b** and **12c** gives first insights on how the optical properties can be fine-tuned by the substitution pattern of the arylalkyne. The key optical properties are summarized in Table 2.

**Table 2:** Spectroscopic data of **6a**, **9a**, **12a**, **12c**, and **12e** in dichloromethane (10^−5^ M) at 20 °C (λ_ex_ = 380 nm).

	**6a**	**9a**	**12a**	**12c**	**12e**

λ_1,abs_ (nm)	280	267	279	298	276
ε_λ1_ (M^−1^cm^−1^)	17200	36600	36600	53900	77600
λ_2,abs_ (nm)	295	282	308	318	292^a^
ε_λ2_ (M^−1^cm^−1^)	17900	39600	42700	55400	64000
λ_3,abs_ (nm)	331	293	315	330*^b^*	303
ε_λ3_ (M^−1^cm^−1^)	17400	43200	43600	51100	56100
λ_4,abs_ (nm)	337	347	377	378	335
ε_λ4_ (M^−1^cm^−1^)	17100	20500	17500	21100	75700
λ_5,abs_ (nm)	365	362^a^	395^a^	397^a^	395
ε_λ5_ (M^−1^cm^−1^)	18800	17500	14300	15800	37400
λ_6,abs_ (nm)					416^a^
ε_λ6_ (M^−1^cm^−1^)					32089
λ_1,em_^380^ (nm)	457	419	459	458	494
Φ^b^	0.66	0.63	0.54	0.56	0.59

^a^Shoulder; ^b^fluorescence standard: perylene in cyclohexane (Φ = 0.94) [35].

**Figure 5:**
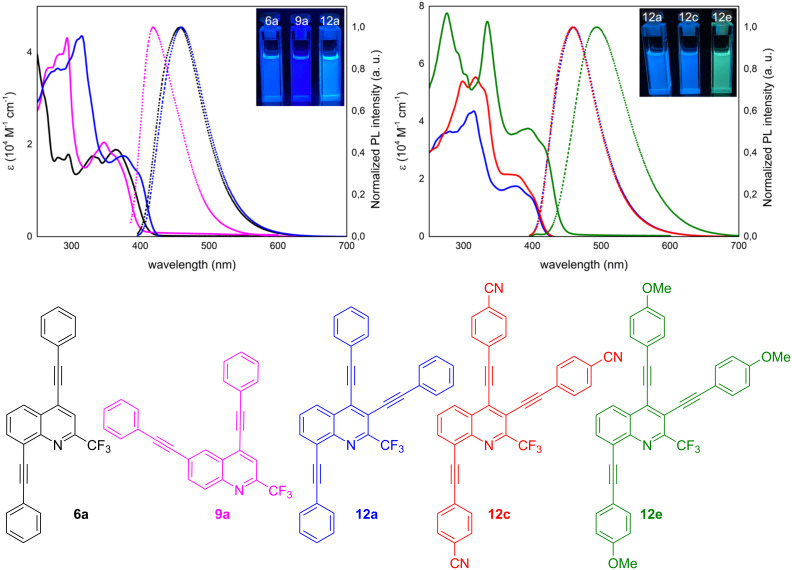
UV–vis and emission spectra of **6a**, **9a** and **12a** (left) and **12a**, **12c**, and **12e** (right, λ_ex_ = 380 nm) in DCM (*c* = 10^−5^ M) at 20 °C.

All molecules display relatively high quantum yields (QYs) with the highest being 0.66 for **6a** and the lowest 0.54 for **12a**. This is in accordance with similar poly-alkynylated molecules from the literature [31,36]. Differences in the absorption and photoluminescence spectra of **6a**, **9a** and **12a** can be explained by the different positions of the alkyne moieties (Figure 5). All absorption spectra exhibit a broad first absorption band with different fine structures. However, TD-DFT calculations using the long-range correlated CAM-B3LYP/6-311G(d,p) level of theory reveal that the lowest energy band for all three derivatives **6a**, **9a** and **12a** originate mainly from a HOMO→LUMO transition with some admixture of HOMO−1→LUMO+1 and HOMO−1→LUMO, respectively. A bathochromic shift is observed from **9a** over **6a** to **12a** in the first absorption maximum with a total of 0.29 eV. This trend can be observed in the emission spectrum as well. All three emission spectra are characterized by a single broad emission peak which is almost identical for **6a** and **12a** and is redshifted by 0.26 eV compared to **9a**. Thus, the position of the substituent at the quinoline core has a significant impact on the optical properties of the molecules. An alkynyl group located at the 8-position seems to have a greater impact on the properties than one at the 3-position, since **6a** and **12a** are almost identical in their emission.

The influence of the type of substituent located at the alkyne moieties of **12a**, **12c**, and **12e** was also studied. The intensity increases from **12a** to **12e** and a bathochromic shift of the absorption maxima can be observed. Interestingly, the emission spectra of **12a** and **12c** are identical which suggests that the strong acceptor groups of **12c** do not have an impact on the emission. On the other hand, the strong donor groups of **12e** cause a redshift of 0.20 eV as compared to the simple phenyl-substituted molecule. Electron-acceptor groups usually have a slighter impact on the emission than electron-donating groups [36]. These findings show that by varying the position and the substituents of the alkynyl groups the properties of the products can be tuned effectively.

## Conclusion

Three different brominated 2-trifluoromethylquinolines have been synthesized and applied to palladium-catalysed Sonogashira reactions. Optimised reaction conditions allow the synthesis of various bis- or tris-alkynylated products in one step. Products were generally obtained in high yields and intensive fluorescence. The photophysical properties of selected compounds were investigated via steady-state absorption and fluorescence spectroscopy and gives first insights into the structure–optical property relationship of polyalkynylated quinolines. In particular, high fluorescence quantum yields have been determined for all studied compounds. Variation of the substitution pattern on the quinoline scaffold and on the arylalkyne moiety permits the fine tuning of the optical properties.

## Supporting Information

File 1 Experimental part.

## Data Availability

All data that supports the findings of this study is available in the published article and/or the supporting information to this article.
