# Supplementary material for: Synthesis and optical properties of bis- and tris-alkynyl-2-trifluoromethylquinolines
Source: Beilstein J Org Chem. 2024 May 29;20:1246–55. doi: 10.3762/bjoc.20.107 (PMC11181212; doi:10.3762/bjoc.20.107)
Supplement: File 1 — Experimental part. [file Beilstein_J_Org_Chem-20-1246-s001.pdf]

BEILSTEIN JOURNAL OF ORGANIC CHEMISTRY

Supporting Information

for

Synthesis and optical properties of bis- and tris-alkynyl-2-trifluoromethylquinolines

Stefan Jopp, Franziska Spruner von Mertz, Peter Ehlers, Alexander Villinger and Peter Langer

Beilstein J. Org. Chem. **2024,** *20,* 1246–1255. doi:10.3762/bjoc.20.107

Experimental part

License and Terms: This is a supporting information file under the terms of the Creative Commons Attribution License (https://creativecommons.org/licenses/by/4.0). Please note that the reuse, redistribution and reproduction in particular requires that the author(s) and source are credited and that individual graphics may be subject to special legal provisions.
The license is subject to the *Beilstein Journal of Organic Chemistry* terms and conditions: (https://www.beilstein-journals.org/bjoc/terms)

Table of contents

1. X-ray crystallography data S2
2. DFT – Calculations S5
   Cartesian coordinates of the optimized ground-state (S0) structures S5
   TD-DFT calculations S8
3. Experimental procedures and data S10
4. NMR-Spectra (^{1}H-,^{13}C-, dept, and ^{19}F-NMR) S22

3 - 8-Bromo-2-trifluoromethyl-4-quinolinone S22
4 - 4,8-Dibromo-2-trifluoromethylquinoline S24
7 - 6-Bromo-2-trifluoromethyl-4-quinolinone S26
8 - 4,6-Dibromo-2-trifluoromethylquinoline S29
10 – 3,8-Dibromo-2-trifluoromethyl-4-quinolinone S32
11 - 3,4,8-Tribromo-2-trifluoromethylquinoline S34
6a - 4,8-Bis(phenylethynyl)-2-trifluoromethylquinoline S36
6b - 4,8-Bis(4'-*tert*-butylphenylethynyl)-2-trifluoromethylquinoline S39
6d - 4,8-Bis(4'-fluorophenylethynyl)-2-trifluoromethylquinoline S42
6e - 4,8-Bis(4'-methoxyphenylethynyl)-2-trifluoromethylquinoline S45
6f - 4,8-Bis(3'-thienylethynyl)-2-trifluoromethylquinoline S48
6g - 4,8-Bis(cyclopropylethynyl)-2-trifluoromethylquinoline S51
9a - 4,6-Bis(phenylethynyl)-2-trifluoromethylquinoline S53
9b - 4,6-Bis(4'-*tert*-butylphenylethynyl)-2-trifluoromethylquinoline S55
9d - 4,6-Bis(4'-fluorophenylethynyl)-2-trifluoromethylquinoline S57
9e - 4,6-Bis(4'-methoxyphenylethynyl)-2-trifluoromethylquinoline S59
9f - 4,6-Bis(3'-thienylethynyl)-2-trifluoromethylquinoline S61
9g – 4,6-Bis(cyclopropylethynyl)-2-trifluoromethylquinoline S63
12a - 3,4,8-Tris(phenylethynyl)-2-trifluoromethylquinoline S66
12b - 3,4,8-Tris(4'-*tert*-butylphenylethynyl)-2-trifluoromethylquinoline S68
12d - 3,4,8-Tris(4'-fluorophenylethynyl)-2-trifluoromethylquinoline S70
12e - 3,4,8-Tris(4'-methoxyphenylethynyl)-2-trifluoromethylquinoline S72
12f - 3,4,8-Tris(3'-thienylethynyl)-2-trifluoromethylquinoline S74
12g - 3,4,8-Tris(cyclopropylethynyl)-2-trifluoromethylquinoline S76

1. X-ray crystallography data

Table S1: X-ray data of ***6b***

Chem. Formula	$C_{34}H_{30}F_3N$
Form. Wght [g mol^{-1}]	509.59
color	colorless
Crsyt. system	triclinic
Space group (Hall group)	P -1 (-P 1)
a [Å]	9.8285 (3)
b [Å]	11.0214 (4)
c [Å]	13.5473 (5)
α [°]	83.944 (2)
β [°]	79.960 (2)
γ [°]	80.206 (2)
V [Å^3]	1419.61 (9)
Z	2
N_{ref}	6781
θ_{max} [°]	28.000
h,k,l_{max}	12,14,17
ρ_x[g cm^{-3}]	1.192
μ [mm^{-1}]	0.082
$\lambda_{MoK\backslash\alpha}$ [A]	0.71073
T [K]	173
F(000)	536.0
N_{par}	396
R	0.0461 (5068)
wR_2	0.1329 (6781)
S	1.039

Table S2: X-ray data of ***9f***

Chem. Formula	$C_{22}H_{10}F_3NS_2$
Form. Wght [g mol^{-1}]	409.43
color	colorless
Crsyt. system	triclinic
Space group (Hall group)	P -1 (-P 1)
a [Å]	5.2778 (2)
b [Å]	11.5411 (4)
c [Å]	15.2732 (5)
α [°]	76.491 (2)
β [°]	86.061 (2)
γ [°]	87.352 (2)
V [Å^3]	902.00 (6)
Z	2
N_{ref}	6485
θ_{max} [°]	32.499
h,k,l_{max}	7,17,23
ρ_x[g cm^{-3}]	1.507
μ [mm^{-1}]	0.332
$\lambda_{MoK\backslash\alpha}$ [A]	0.71073
T [K]	173
F(000)	416.0
N_{par}	294
R	0.0485 (4551)
wR_2	0.1356 (6485)
S	1.025

*Table S3: X-ray data of **12d***

Chem. Formula	$C_{34}H_{15}F_6N$
Form. Wght [g mol^{-1}]	551.47
color	yellow
Crsyt. system	monoclinic
Space group (Hall group)	C 2/c (-C 2yc)
a [Å]	31.408 (2)
b [Å]	7.1005 (4)
c [Å]	23.2841 (15)
α [°]	90
β [°]	91.001 (4)
γ [°]	90
V [Å^3]	5191.9 (6)
Z	8
N_{ref}	6244
θ_{max} [°]	27.998
h,k,l_{max}	41,9,30
ρ_x[g cm^{-3}]	1.411
μ [mm^{-1}]	0.112
$\lambda_{MoK\backslash\alpha}$ [A]	0.71073
T [K]	173
F(000)	2240.0
N_{par}	494
R	0.0503 (2846)
wR_2	0.1354 (6244)
S	0.994

2. DFT – Calculations

Density functional theory (DFT) and time-dependent DFT (TD-DFT) calculations were performed with Gaussian 09[67] using the B3LYP functional in combination with the 6-31+G(d,p) basis set and the CAM-B3LYP functional in combination with the 6-311G(d,p) basis set for TD-DFT calculations, respectively. To include solvent effects, a polarizable continuum model (PCM) of dichloromethane using the integral equation formalism variant (IEFPCM) was applied. The DFT calculations were performed for compounds **6a, 9a** and **12a**.

Cartesian coordinates of the optimized ground-state (S0) structures

Table 2. Cartesian coordinates of **6a** (E = -1353.42 Hartree)

	X	Y	Z
C	-0.1246120	1.6852640	-0.0099360
C	-0.5263180	-0.5718060	0.0001950
C	0.8731070	-0.8735990	-0.0013260
C	1.7905080	0.2318530	-0.0077420
C	1.2710250	1.5221790	-0.0133830
C	-1.4772640	-1.6548640	0.0047110
C	1.3002270	-2.2237600	0.0029730
H	1.9280940	2.3815900	-0.0204490
C	0.3737180	-3.2441630	0.0084350
C	-1.0074050	-2.9643410	0.0091470
H	2.3632390	-2.4381510	0.0016920
H	0.7050100	-4.2775540	0.0118090
H	-1.7223230	-3.7800170	0.0127990
N	-1.0013550	0.6991380	-0.0024370
C	-0.7356830	3.0739330	0.0087150
F	-1.6415720	3.2299980	-0.9770730
F	0.2003300	4.0368390	-0.1431340
F	-1.3724120	3.3138570	1.1757560
C	3.1947180	0.0234090	-0.0087520
C	4.4012910	-0.1392480	-0.0077070
C	-2.8721200	-1.3839730	0.0035160
C	-4.0720480	-1.1811470	0.0008210
C	5.8144440	-0.3167450	-0.0058690
C	6.6712070	0.8033890	-0.0160970
C	6.3733570	-1.6112980	0.0068360
C	8.0518130	0.6270510	-0.0135840
H	6.2430440	1.8001840	-0.0258230
C	7.7554650	-1.7757680	0.0093170
H	5.7165320	-2.4748300	0.0148290
C	8.5972570	-0.6599180	-0.0008540
H	8.7041300	1.4947050	-0.0214630
H	8.1773840	-2.7759000	0.0192370
H	9.6747140	-0.7927650	0.0011510
C	-5.4707340	-0.9093770	-0.0029580
C	-6.4104370	-1.9605560	0.0063840

**C**	-5.9336910	0.4229250	-0.0164720
**C**	-7.7746720	-1.6817280	0.0024150
**H**	-6.0589480	-2.9871470	0.0166980
**C**	-7.2998240	0.6906240	-0.0203100
**H**	-5.2122540	1.2333060	-0.0240310
**C**	-8.2238300	-0.3582470	-0.0108670
**H**	-8.4896740	-2.4988450	0.0096770
**H**	-7.6451140	1.7199920	-0.0307350
**H**	-9.2884720	-0.1453320	-0.0139210

Table 3. Cartesian coordinates of **9a** (E = -1353.42 Hartree)

	**X**	**Y**	**Z**
C	-0.6675242	-3.1986619	-0.0055722
C	0.6838032	-3.4277017	-0.0062626
C	1.6060806	-2.3446464	-0.0069977
C	1.0988036	-1.0046506	-0.0076636
C	-0.2966960	-0.7913940	-0.0069217
C	-1.1830734	-1.8633199	-0.0056549
H	-1.3661517	-4.0283136	-0.0047626
H	1.0863893	-4.4348748	-0.0060129
C	2.0550753	0.0681351	-0.0087882
H	-0.6761374	0.2240809	-0.0072296
C	3.4074240	-0.2589034	-0.0099935
C	3.7779418	-1.6152905	-0.0085167
H	4.1612059	0.5170180	-0.0119381
N	2.9346405	-2.6317038	-0.0064918
C	5.2427924	-2.0090001	0.0062714
F	5.5461713	-2.8261532	-1.0230218
F	5.5631488	-2.6656066	1.1422422
F	6.0544385	-0.9319998	-0.0785023
C	1.6416667	1.4253775	-0.0082693
C	-2.5895231	-1.6476049	-0.0039843
C	-3.7946342	-1.4755718	-0.0019060
C	1.2990761	2.5937800	-0.0062010
C	0.9108501	3.9638608	-0.0031814
C	1.8891559	4.9796784	-0.0013530
C	-0.4533040	4.3209827	-0.0015410
C	1.5068618	6.3179346	0.0020561
H	2.9391398	4.7064861	-0.0025550
C	-0.8240296	5.6625600	0.0018602
H	-1.2076989	3.5412121	-0.0028775
C	0.1522652	6.6629692	0.0036803
H	2.2662564	7.0936201	0.0034988
H	-1.8763446	5.9291277	0.0031581
H	-0.1416876	7.7080487	0.0064005
C	-5.2045824	-1.2678320	0.0009545
C	-6.0896406	-2.3656835	-0.0007970
C	-5.7343906	0.0390785	0.0058931
C	-7.4660270	-2.1563872	0.0023498

H	-5.6864100	-3.3730016	-0.0045752
C	-7.1123758	0.2371715	0.0090507
H	-5.0567975	0.8865534	0.0072899
C	-7.9817225	-0.8572796	0.0073046
H	-8.1385048	-3.0087705	0.0009780
H	-7.5094737	1.2476492	0.0129151
H	-9.0557353	-0.6986783	0.0097902

Table 4. Cartesian coordinates of **12a** (E = -1660.64 Hartree)

	X	Y	Z
C	-0.8007080	0.7586130	0.0107180
C	-1.7049170	-1.3564860	0.0102840
C	-0.4182290	-1.9727500	0.0070870
C	0.7346460	-1.1171430	0.0044450
C	0.5490800	0.2783450	0.0007190
C	-2.8852190	-2.1776860	0.0102940
C	-0.3222250	-3.3858320	0.0092560
C	-1.4650560	-4.1583010	0.0107510
C	-2.7410040	-3.5626720	0.0105050
H	0.6588330	-3.8471650	0.0098390
H	-1.3856160	-5.2406010	0.0121250
H	-3.6285860	-4.1859070	0.0106780
N	-1.8635160	-0.0056190	0.0141140
C	2.0396730	-1.6667640	0.0108070
C	1.6474840	1.1718870	-0.0173890
C	-4.1694070	-1.5701400	0.0083720
C	2.5595660	1.9765880	-0.0403220
C	-5.2655820	-1.0422230	0.0043040
C	3.1604500	-2.1430250	0.0247310
C	3.5856950	2.9630180	-0.0523480
C	3.2659640	4.3032920	0.2493970
C	4.9181150	2.6256470	-0.3638800
C	4.2599260	5.2773650	0.2421710
H	2.2384980	4.5588280	0.4869550
C	5.9040650	3.6086650	-0.3696070
H	5.1643610	1.5968570	-0.6051230
C	5.5795850	4.9340430	-0.0664140
H	4.0057020	6.3064370	0.4767090
H	6.9279820	3.3411240	-0.6118070
H	6.3521070	5.6968260	-0.0715950
C	4.4766970	-2.6851740	0.0423060
C	4.6911650	-4.0518360	-0.2312690
C	5.5822900	-1.8593180	0.3338750
C	5.9815550	-4.5732920	-0.2167290
H	3.8425470	-4.6893490	-0.4561300
C	6.8679960	-2.3920720	0.3477940
H	5.4181700	-0.8094820	0.5526170
C	7.0715790	-3.7472750	0.0720010

H	6.1379630	-5.6260550	-0.4302910
H	7.7132800	-1.7497920	0.5744510
H	8.0762840	-4.1583620	0.0828390
C	-6.5179890	-0.3634920	-0.0019350
C	-7.7337120	-1.0765780	-0.0018610
C	-6.5487580	1.0469740	-0.0092150
C	-8.9466020	-0.3925150	-0.0090300
H	-7.7138360	-2.1615060	0.0036740
C	-7.7667970	1.7206320	-0.0164100
H	-5.6116130	1.5942940	-0.0092180
C	-8.9678060	1.0049920	-0.0163940
H	-9.8779440	-0.9506500	-0.0089940
H	-7.7802360	2.8062990	-0.0221280
H	-9.9158730	1.5340940	-0.0221290
C	-1.0681150	2.2588400	0.0016860
F	-0.5668960	2.8363080	-1.1143570
F	-2.3787460	2.5400110	0.0463080
F	-0.4874520	2.8700680	1.0609950

TD-DFT calculations

Table 5. Calculated TD-DFT transitions of compound ***6a*** *at CAM B3LYP/6-311+G(d,p) level*

S_n	E (eV)	λ (nm)	*f*	Configuration	CI coefficient
S_1	3.3151	374.00	1.0553	HOMO-1 → LUMO<br>HOMO → LUMO	0.10523<br>0.67164
S_2	3.9717	312.17	0.1679	HOMO-9 → LUMO<br>HOMO-1 → LUMO<br>HOMO → LUMO+1	-0.10990<br>0.63800<br>-0.18925
S_3	4.2702	279.09	0.6355	HOMO-5 → LUMO<br>HOMO-4 → LUMO<br>HOMO-4 → LUMO+2<br>HOMO → LUMO+2	0.44274<br>-0.25585<br>0.13505<br>0.42129
S_4	4.4425	279.09	0.6355	HOMO-4 → LUMO<br>HOMO-1 → LUMO<br>HOMO-1 → LUMO+1<br>HOMO → LUMO+1<br>HOMO → LUMO+3	-0.13509<br>0.13831<br>-0.13611<br>0.59405<br>0.19617

*Table 6. Calculated TD-DFT transitions of compound **9a** at CAM B3LYP/6-311+G(d,p) level*

S_n	E (eV)	λ (nm)	*f*	Configuration	CI coefficient
S_1	3.3806	366.76	0.7035	HOMO-1 → LUMO+1	0.13067
				HOMO-1 → LUMO+1	-0.12660
				HOMO → LUMO	0.66431
S_2	4.0123	309.01	0.5248	HOMO-5 → LUMO	0.19465
				HOMO-2 → LUMO	-0.17599
				HOMO-2 → LUMO+1	-0.12319
				HOMO-1 → LUMO	0.57571
				HOMO → LUMO+2	-0.16347
S_3	4.2337	292.85	0.9190	HOMO-5 → LUMO	0.12480
				HOMO-2 → LUMO	-0.26859
				HOMO-1 → LUMO	-0.25375
				HOMO → LUMO+1	0.53516
S_4	4.5081	275.02	0.3889	HOMO-9 → LUMO	0.10789
				HOMO-2 → LUMO	0.54600
				HOMO-1 → LUMO	0.10004
				HOMO-1 → LUMO+1	-0.15181
				HOMO → LUMO+1	0.32116
				HOMO → LUMO+3	-0.12706

*Table 7. Calculated TD-DFT transitions of compound **12a** at CAM B3LYP/6-311+G(d,p) level*

S_n	E (eV)	λ (nm)	*f*	Configuration	CI coefficient
S_1	3.2201	385.03	0.6748	HOMO-1 → LUMO+1	0.12117
				HOMO → LUMO	0.66388
S_2	3.6442	340.22	0.6434	HOMO-6 → LUMO	-0.10103
				HOMO-1 → LUMO	0.60294
				HOMO → LUMO+1	0.27653
				HOMO → LUMO+3	0.10481
S_3	3.8748	319.98	0.0127	HOMO-8 → LUMO	-0.11480
				HOMO-7 → LUMO	-0.15399
				HOMO-6 → LUMO	0.10373
				HOMO-2 → LUMO	0.35367
				HOMO-1 → LUMO	-0.15072
				HOMO → LUMO+1	0.47310
				HOMO → LUMO+2	-0.13448
S_4	4.1239	300.65	1.4246	HOMO-2 → LUMO	0.52883
				HOMO-1 → LUMO	0.16087
				HOMO-1 → LUMO+2	-0.12470
				HOMO → LUMO+1	-0.31346

3. Experimental procedures and data

The nuclear magnetic resonance spectra ($^{1}H/^{13}C/^{19}F$ NMR) were recorded on a Bruker AVANCE 300 III, 250 II, or 500. The analyzed chemical shifts δ are referenced to residual solvents signals of the deuterated solvents $CDCl_3$ (δ = 7.26 ppm/77.0 ppm). Multiplicities due to spin−spin correlation are reported as follows, s = singlet, d = doublet, dd = double doublet, ddd = doublets of doublets, pt = pseudo triplet, m = multiplet, and further described through their coupling constants J. Infrared spectra (IR) were measured as attenuated total reflection (ATR) experiments with a Nicolet 380 FT-IR spectrometer. The signals have been characterized through their wave numbers ṽ and their corresponding absorption as very strong (vs), strong (s), medium (m) or weak (w). UV–vis spectra were recorded on a Cary 60 UV−vis spectrophotometer and emission spectra with an Agilent Cary Eclipse fluorescence spectrophotometer. Basic and high-resolution mass spectra (MS/HRMS) were measured on instruments which are paired with a preceding gas chromatograph (GC) or liquid chromatograph (LC). The samples have been ionized through electron impact ionization (EI) on an Agilent 6890/5973 or Agilent 7890/5977 GC–MS equipped with a HP-5 capillary column using helium carrier gas or by applying electron spray ionization (ESI) on an Agilent 1200/6210 Time-of-Flight (TOF) LC−MS. Melting points (mp) were determined by a Micro-Hot-Stage GalenTM III Cambridge Instruments and are not corrected. X-ray single-crystal structure analysis was performed on a Bruker Apex Kappa-II CCD diffractometer. CCDCs 2322983-2322985 contain supplementary crystallographic data for this paper. These data can be obtained free of charge from The Cambridge Crystallographic Data Centre via www.ccdc.cam.ac.uk/data_request/cif.

Synthesis of 8-bromo-2-trifluoromethyl-4-quinolinone (3)[i]

Polyphosphoric acid (0.8 g/mmol, 46.40 g) and ethyl 4,4,4-trifluoroacetoacetate (**2**, 58 mmol, 8.48 mL) were heated to 100 °C in a 500 mL flask. 2-Bromoaniline (**1a**, 58 mmol, 9.98 g) was added quickly and the reaction was stirred for 2 h at 150 °C. After cooling down, a 10% NaOH solution was added until a white precipitate is formed. Additional, 10% NaOH solution was added until the precipitate is dissolved again. Overall 360 mL 10% NaOH solution was used. The solution was filtered and the filtrate was made acidic via 36% HCl. The white precipitate was collected and dried to yield **3** as white solid (12.22 g, 72%). mp. 127–128 °C. ^{1}H NMR (300 MHz, DMSO-*d6*): δ = 7.39 (s, 1H,); 7.54 (pt, ^{3}J = 7.90 Hz, 1H); 8.18–8.24 (m, 2H). ^{13}C NMR (75 MHz, DMSO-*d6*): δ = 100.9 (q, ^{3}J = 2.0 Hz, CH); 121.3 (q, ^{1}J = 275.6 Hz, CF_3); 122.3 (CH); 122.8, 124.0 (C); 128.0, 134.9 (CH); 145.1 (C); 148.0 (q, ^{2}J = 33.5 Hz, *C*-CF_3); 163.9 (C=O). ^{19}F NMR (282 MHz, DMSO-*d6*): δ = -66.58. MS (EI, 70 eV): m/z (%) = 294 (11), 293 (M^+, ^{81}Br, 98), 292 (13), 291 (M^+, ^{79}Br, 100), 265 (18), 263 (19), 245 (49), 243 (48), 183 (10), 164 (14), 144 (16), 88 (10), 75 (16), 63 (10). HRMS (EI, 70 eV): Calculated for $C_{10}H_5{}^{79}BrF_3ON$ (M^+), 290.95011; measured 290.94938. Calculated for $C_{10}H_5{}^{81}BrF_3ON$ (M^+), 292.94807; measured 292.94752.

Synthesis of 4,8-dibromo-2-trifluoromethylquinoline (4)[i]

Phosphorus oxybromide (4.4 mmol, 1.26 g) was heated to 75 °C under reflux. **3** (4.0 mmol, 1.17 g) was added quickly and the reaction was stirred for 2 h at 150 °C. After cooling down, 10 mL ice-water were added carefully and the solution was made alkaline with a 10% NaOH solution. The aqueous solution was decanted and the remaining solid was collected and dried to yield **4** as yellow solid (1.05 g, 74%). mp. 85–86 °C. ^{1}H NMR (300 MHz, DMSO-d_6): δ = 7.81 (pt, ^3J = 8.00 Hz, 1H); 8.27 (dd, ^3J = 8.50 Hz, ^4J = 1.10 Hz, 1H); 8.39 (dd, ^3J = 7.60 Hz, ^4J = 1.10 Hz, 1H); 8.51 (s, 1H). ^{13}C NMR (75 MHz, DMSO-d_6): δ = 120.5 (q, ^1J = 275.6 Hz, CF_3); 122.2 (q, ^3J = 2.20 Hz, CH); 124.9 (C); 127.8 (CH); 129.5 (C); 131.3, 135.9 (CH); 136.5 (C); 143.7 (C); 146.9 (q, ^2J = 35.0 Hz, *C*-CF_3). ^{19}F NMR (282 MHz, DMSO-d_6): δ = -65.99. MS (EI, 70 eV): *m/z* (%) = 357 (M^+, 2x ^{81}Br, 50), 356 (11), 355 (M^+, 1x ^{79}Br, 1x ^{81}Br, 50), 353 (M^+, 2x ^{79}Br, 51), 286 (18), 207 (11), 205 (12), 100 (14), 99 (15), 74 (11). HRMS (EI, 70 eV): Calculated for $C_{10}H_4{}^{79}Br_2F_3N$ (M^+), 352.86571; measured 352.86542. Calculated for $C_{10}H_4{}^{79}Br^{81}BrF_3N$ (M^+), 354.86366; measured 354.86345. Calculated for $C_{10}H_4{}^{81}Br_2F_3N$ (M^+), 356.86162; measured 356.89097.

Synthesis of 6-bromo-2-trifluoromethyl-4-quinolinone (7)[ii]

7 was synthesized analogously to **3**. Polyphosphoric acid (0.8 g/mmol, 12.00 g), ethyl 4,4,4-trifluoroacetoacetate (**2**, 15 mmol, 2.19 mL) and 4-bromoaniline (**1b**, 15 mmol, 2.63 g) were used to yield **7** as white solid (1.63 g, 37%). The ^{13}C-NMR was not analyzable, due to low solubility. mp. 243–244 °C. ^{1}H NMR (300 MHz, DMSO-d_6): δ = 7.16 (s, 1H); 7.92 (s, 2H); 8.29 (s, 1H). ^{19}F NMR (282 MHz, DMSO-d_6): δ = -66.48. IR (ATR, cm^{-1}): $\tilde{\nu}$ = 3072 (w), 2881 (m), 2792 (m), 1608 (m), 1555 (s), 1508 (s), 1467 (s), 1419 (m), 1354 (m), 1297 (s), 1278 (s), 1193 (s), 1148 (s), 1134 (s), 1108 (s), 1090 (s), 942 (m), 854 (s), 827 (s), 723 (s), 555 (s), 538 (s). MS (EI, 70 eV): *m/z* (%) = 294 (11), 293 (M^+, ^{81}Br, 94), 292 (14), 291 (M^+, ^{79}Br, 100), 245 (13), 243 (14), 224 (18), 222 (19), 184 (20), 183 (11), 164 (10), 134 (10), 115 (11), 114 (10), 88 (13), 75 (14), 74 (11), 69 (13), 63 (13), 62 (11). HRMS (EI, 70 eV): Calculated for $C_{10}H_5{}^{79}BrF_3ON$ (M^+), 290.95011; measured 290.95070. Calculated for $C_{10}H_5{}^{81}BrF_3ON$ (M^+), 292.94807; measured 292.94898.

Synthesis of 4,6-dibromo-2-trifluoromethylquinoline (8)[iii]

8 was synthesized analogously to **4**. Phosphorus oxybromide (4.4 mmol, 1.26 g) and **7** (4.0 mmol, 1.17 g) were used to yield **8** as gray solid (1.24 g, 87%). mp. 120–121 °C. ^{1}H NMR (300 MHz, $CDCl_3$): δ = 7.93 (dd, ^3J = 8.87 Hz, ^4J = 2.08 Hz, 1H); 8.04 (s, 1H); 8.08 (d, ^3J = 8.87 Hz, 1H); 8.43 (d, ^4J = 2.08 Hz, 1H). ^{13}C NMR (75 MHz, $CDCl_3$): δ = 120.7 (q, ^1J = 275.6 Hz, CF_3); 121.8 (q, ^3J = 2.20 Hz, CH); 124.7 (C); 129.1 (CH); 129.6 (C); 132.2 (CH); 134.5 (C); 135.4 (CH); 146.2 (C); 147.9 (q, ^2J = 35.6 Hz, *C*-CF_3). ^{19}F NMR (282 MHz, $CDCl_3$): δ = -67.58. IR (ATR, cm^{-1}): $\tilde{\nu}$ = 3084 (w), 3059 (w), 1743 (w), 1602 (w), 1579 (w), 1547 (m), 1478 (m), 1449 (m), 1335 (m), 1194 (m), 1141 (s), 1094 (m), 1064 (m), 873 (m), 836 (m), 766 (m), 681 (s),

640 (m), 608 (m). MS (EI, 70 eV): *m/z* (%) = 357 (M^+, 2x ^{81}Br, 50), 356 (11), 355 (M^+, 1x ^{79}Br, 1x ^{81}Br, 50), 353 (M^+, 2x ^{79}Br, 51), 286 (15), 276 (10), 274 (10), 207 (11), 205 (11), 100 (20), 99 (20), 74 (16), 69 (11). HRMS (EI, 70 eV): Calculated for $C_{10}H_4{}^{79}Br_2F_3N$ (M^+), 352.86571; measured 352.86648. Calculated for $C_{10}H_4{}^{79}Br^{81}BrF_3N$ (M^+), 354.86366; measured 354.86461.

Synthesis of 3,8-dibromo-2-trifluoromethyl-4-quinolinone (10)

3 (35.0 mmol, 10.22 g) was dissolved in glacial acetic acid (2.5 mL/mmol, 87.5 mL) and bromine (38.5 mmol, 6.15 g) was added slowly. The reaction was heated under reflux for 24 h. After cooling down an orange precipitate was formed. The precipitate was filtered, washed with water, collected and dried to yield **10** as white solid (9.61 g, 74%). The product exists in $CDCl_3$ as a mixture of keto- and enol-tautomers in a ratio of 84:16. mp. 162–163 °C. ^1H NMR (250 MHz, $CDCl_3$): δ = 7.35 (pt, ^3J = 7.96 Hz, 1H); 7.49 (pt, ^3J = 7.96 Hz, enol); 7.94 (dd, ^3J = 7.72 Hz, ^4J = 1.26 Hz, 1H); 8.12 (dd, ^3J = 7.72 Hz, ^4J = 1.26 Hz, enol); 8.23 (dd, ^3J = 8.35 Hz, ^4J = 1.26 Hz, enol); 8.35 (dd, ^3J = 8.35 Hz, ^4J = 1.26 Hz, 1H); 9.02 (s, 1H, NH, enol). ^{13}C NMR (63 MHz, $CDCl_3$): δ = 107.6, 111.8 (C); 119.7 (q, ^1J = 276.9 Hz, CF_3); 121.9 (C); 124.5 (CH, enol); 126.2, 126.8 (CH); 128.9 (CH, enol); 134.8 (C); 135.1 (CH, enol); 135.7 (q, ^2J = 34.2 Hz, *C*-CF_3); 136.3 (CH); 172.8 (C=O). ^{19}F NMR (282 MHz, $CDCl_3$): δ = -66.23 (enol); -66.11 (keto). IR (ATR, cm^{-1}): $\tilde{\nu}$ = 3343 (m), 2068 (w), 1941 (w), 1887 (w), 1834 (w), 1731 (w), 1644 (w), 1595 (m), 1558 (m), 1525 (m), 1442 (m), 1392 (m), 1364 (m), 1310 (m), 1248 (m), 1186 (s), 1165 (s), 1135 (s), 1102 (s), 955 (m), 935 (s), 809 (m), 755 (s), 742 (s), 630 (m), 601 (s), 526 (m). MS (EI, 70 eV): *m/z* (%) = 373 (M^+, 2x ^{81}Br, 48), 372 (14), 371 (M^+, 1x ^{79}Br, 1x ^{81}Br, 100), 369 (M^+, 2x ^{79}Br, 52), 323 (20), 321 (10), 183 (12), 163 (23). HRMS (EI, 70 eV): Calculated for $C_{10}H_4{}^{79}Br_2F_3ON$ (M^+), 369.86845; measured 369.86856. Calculated for $C_{10}H_4{}^{79}Br^{81}BrF_3ON$ (M^+), 371.86644; measured 371.86682. Calculated for $C_{10}H_4{}^{81}Br_2F_3ON$ (M^+), 373.86448; measured 373.86464.

Synthesis of 3,4,8-tribromo-2-trifluoromethylquinoline (11) [34]

11 was synthesized analogously to **4**. Phosphorus oxybromide (4.4 mmol, 1.26 g) and **10** (4.0 mmol, 1.48 g) were used to yield **11** as white solid (1.56 g, 90%). mp. 132–133 °C. ^1H NMR (300 MHz, $CDCl_3$): δ = 7.60 (pt, ^3J = 8.13 Hz, 1H); 8.17 (dd, ^3J = 7.53 Hz, ^4J = 0.93 Hz, 1H); 8.27 (dd, ^3J = 8.52 Hz, ^4J = 0.92 Hz, 1H). ^{13}C NMR (75 MHz, $CDCl_3$): δ = 118.2 (C); 120.3 (q, ^1J = 276.7 Hz, CF_3); 126.4 (C); 127.6 (CH); 130.9 (C); 131.2, 135.1 (CH); 140.1 (C); 141.8 (q, ^3J = 1.10 Hz, C-3); 145.8 (q, ^2J = 35.0 Hz, *C*-CF_3). ^{19}F NMR (282 MHz, $CDCl_3$): δ = -66.15. IR (ATR, cm^{-1}): $\tilde{\nu}$ = 3107 (w), 1972 (w), 1894 (w), 1814 (w), 1714 (w), 1598 (w), 1442 (m), 1379 (m), 1350 (m), 1305 (m), 1290 (m), 1258 (m), 1190 (s), 1129 (s), 1003 (m), 892 (m), 804 (m), 760 (s), 712 (s), 623 (m). MS (EI, 70 eV): *m/z* (%) = 437 (M^+, 3x ^{81}Br, 34), 436 (10), 435 (M^+, 1x ^{79}Br, 2x ^{81}Br, 94), 434 (11), 433 (M^+, 2x ^{79}Br, 1x ^{81}Br, 100), 431 (M^+, 3x ^{79}Br, 35), 364 (10), 354 (13), 285 (21), 283 (11), 194 (18), 99 (15), 69 (15). HRMS (EI, 70 eV): Calculated for $C_{10}H_3{}^{79}Br_3F_3N$ (M^+),

430.77622; measured 430.77700. Calculated for $C_{10}H_3{}^{79}Br_2{}^{81}BrF_3N$ (M^+), 432.77418; measured 432.77443. Calculated for $C_{10}H_3{}^{79}Br^{81}Br_2F_3N$ (M^+), 434.77213; measured 434.77200. Calculated for $C_{10}H_3{}^{81}Br_3F_3N$ (M^+), 436.77008; measured 436.77002.

General procedure A for the twofold Sonogashira reaction

4 or **8** (0.5 mmol, 177 mg), $Pd(PPh_3)_4$ (0.0125 mmol, 2.5 mol %, 14.4 mg), copper iodide (0.025 mmol, 5 mol %, 4.8 mg) and corresponding acetylene **5** (1.5 mmol) were dissolved in dioxane (2 mL) and NEt_3 (1 mL). The reaction mixture was stirred at 100 °C for 6 h. After cooling to room temperature, the solution was diluted with water and extracted with ethyl acetate (3×). The crude product **6** or **9** was purified by column chromatography.

General procedure B for the threefold Sonogashira reaction

11 (0.5 mmol, 217 mg), $Pd(PPh_3)_4$ (0.0125 mmol, 2.5 mol %, 14.4 mg), copper iodide (0.025 mmol, 5 mol %, 4.8 mg) and corresponding acetylene **5** (2.0 mmol) were dissolved in dioxane (2 mL) and NEt_3 (1 mL). The reaction mixture was stirred at 100 °C for 24 h. After cooling to room temperature, the solution was diluted with water and extracted with ethyl acetate (3×). The crude product **12** was purified by column chromatography.

4,8-Bis(phenylethynyl)-2-trifluoromethylquinoline (6a)

6a was synthesized according to general procedure A using **4** and phenylacetylene (**5a**, 1.5 mmol, 165 µL) and was purified via column chromatography (heptane/dichloromethane 10:1). Yield: 197 mg (99%). mp. 146–147 °C. ^{1}H NMR (300 MHz, $CDCl_3$): δ = 7.38–7.48 (m, 6H); 7.67–7.74 (m, 5H); 7.94 (s, 1H); 8.08 (dd, ^3J = 7.30 Hz, ^4J = 1.35 Hz, 1H); 8.39 (dd, ^3J = 8.40 Hz, ^4J = 1.35 Hz, 1H). ^{13}C NMR (75 MHz, $CDCl_3$): δ = 84.4, 86.3, 97.2, 100.7 (C≡C); 119.9 (q, ^3J = 2.20 Hz, CH); 121.4 (q, ^1J = 275.0 Hz, CF_3); 121.6, 123.3, 124.9 (C); 126.0, 128.4 (CH); 128.5 (C); 128.5, 128.6, 128.7, 129.9, 132.0, 132.1 (CH); 132.4 (C); 134.7 (CH); 147.1 (C); 147.8 (q, ^2J = 35.2 Hz, *C*-CF_3). ^{19}F NMR (282 MHz, $CDCl_3$): δ = -67.56. IR (ATR, cm^{-1}): $\tilde{\nu}$ = 3051 (w), 2923 (w), 2852 (w), 2197 (m), 1958 (w), 1888 (w), 1818 (w), 1740 (w), 1583 (m), 1503 (m), 1461 (m), 1389 (m), 1278 (m), 1171 (m), 1115 (s), 929 (m), 879 (m), 750 (m), 682 (s), 535 (m). MS (EI, 70 eV): *m/z* (%) = 398 (26), 397 (M^+, 100), 329 (14), 328 (50), 327 (10), 326 (15). HRMS (EI, 70 eV): Calculated for $C_{26}H_{14}F_3N$ (M^+), 397.10729; measured 397.10817.

4,8-Bis(4'-*tert*-butylphenylethynyl)-2-trifluoromethylquinoline (6b)

6b was synthesized according to general procedure A using **4** and 4-*tert*-butylphenylacetylene (**5b**, 1.5 mmol, 270 µL) and was purified via column chromatography (heptane/dichloromethane 10:1). Yield: 234 mg (92%). mp. 159–160 °C. ^{1}H NMR (500 MHz, $CDCl_3$): δ = 1.36 (s, 9H, *t*-Bu); 1.37 (s, 9H, *t*Bu); 7.43 (d, ^3J = 8.51 Hz, 2H); 7.47 (d, ^3J = 8.51 Hz, 2H); 7.62 (d, ^3J = 8.51 Hz, 2H); 7.64 (d, ^3J = 8.51 Hz, 2H);

7.70 (pt, ^3J = 7.70 Hz, 1H); 7.92 (s, 1H); 8.06 (dd, ^3J = 7.25 Hz, ^4J = 1.26 Hz, 1H); 8.38 (dd, ^3J = 8.19 Hz, ^4J = 1.26 Hz, 1H). ^{13}C NMR (125 MHz, $CDCl_3$): δ = 31.1, 31.2 (CH_3); 34.9, 35.0 (*C*(Me)$_3$); 84.0, 85.8, 97.4, 101.1 (C≡C); 118.6 (C); 119.8 (q, ^3J = 1.80 Hz, CH); 120.3 (C); 121.5 (q, ^1J = 275.5 Hz, CF_3); 125.2 (C); 125.4, 125.7, 125.9, 128.5 (CH); 128.6 (C); 131.7, 131.9 (CH); 132.6 (C); 134.5 (CH); 147.1 (C); 147.8 (q, ^2J = 35.1 Hz, *C*-CF_3); 151.9, 153.5 (C). ^{19}F NMR (282 MHz, $CDCl_3$): δ = -67.52. IR (ATR, cm^{-1}): $\tilde{\nu}$ = 3068 (w), 2960 (m), 2867 (w), 2203 (m), 1587 (m), 1507 (m), 1462 (m), 1408 (m), 1362 (m), 1279 (m), 1185 (m), 1123 (s), 1105 (s), 1017 (m), 933 (m), 882 (m), 832 (s), 763 (s), 737 (m), 627 (m), 560 (s). MS (EI, 70 eV): *m/z* (%) = 510 (24), 509 (M^+, 66), 495 (35), 494 (100), 478 (20), 464 (14), 240 (15), 211 (10). HRMS (ESI): Calculated for $C_{34}H_{30}F_3N$ ($[M+H]^+$), 510.24031; measured 510.24024.

4,8-Bis(4'-cyanophenylethynyl)-2-trifluoromethylquinoline (6c)

6c was synthesized according to general procedure A using **4** and 4-cyanophenylacetylene (**5c**, 1.5 mmol, 191 mg). During aqueous work-up the product precipitates. The solid was filtered and washed with dichloromethane. Product **6c** was insoluble even at high temperatures, thus making an NMR impossible. Yield: 158 mg (71%). mp. 292–293 °C. IR (ATR, cm^{-1}): $\tilde{\nu}$ = 2974 (w), 2936 (w), 2674 (w), 2490 (w), 2222 (m), 1603 (m), 1505 (m), 1463 (m), 1409 (m), 1393 (m), 1285 (m), 1182 (m), 1113 (s), 932 (m), 886 (m), 839 (s), 766 (s), 744 (m), 640 (m), 552 (s). MS (EI, 70 eV): *m/z* (%) = 448 (32), 447 (M^+, 100), 379 (15), 378 (52), 376 (11). HRMS (EI, 70 eV): Calculated for $C_{28}H_{12}F_3N_3$ (M^+), 447.09778; measured 447.09818.

4,8-Bis(4'-fluorophenylethynyl)-2-trifluoromethylquinoline (6d)

6d was synthesized according to general procedure A using **4** and 4-fluorophenylacetylene (**5d**, 1.5 mmol, 180 mg) and was purified via column chromatography (heptane/dichloromethane 10:1). Yield: 188 mg (87%). mp. 159–160 °C. ^{1}H NMR (300 MHz, $CDCl_3$): δ = 7.07–7.16 (m, 4H); 7.64–7.71 (m, 5H); 7.91 (s, 1H); 8.05 (dd, ^3J = 7.18 Hz, ^4J = 1.32 Hz, 1H); 8.35 (dd, ^3J = 8.31 Hz, ^4J = 1.32 Hz, 1H). ^{13}C NMR (75 MHz, $CDCl_3$): δ = 84.1, 86.0, 96.1, 99.5 (C≡C); 115.7 (d, ^2J = 22.0 Hz, CH); 116.2 (d, ^2J = 22.5 Hz, CH); 117.7 (d, ^4J = 3.30 Hz, C); 119.3 (d, ^4J = 3.85 Hz, C); 119.9 (q, ^3J = 1.65 Hz, CH); 121.4 (q, ^1J = 275.6 Hz, CF_3); 124.8 (C); 126.0 (CH); 128.4 (C); 128.6 (CH); 132.3 (C); 133.9 (d, ^3J = 8.26 Hz, CH); 134.1 (d, ^3J = 8.25 Hz, CH); 134.6 (CH); 147.0 (C); 147.8 (q, ^2J = 35.4 Hz, *C*-CF_3); 162.8 (d, ^1J = 249.7 Hz, CF); 163.4 (d, ^1J = 251.9 Hz, CF). ^{19}F NMR (282 MHz, $CDCl_3$): δ = -110.27 (CF); -107.85 (CF); -67.52 (CF_3). IR (ATR, cm^{-1}): $\tilde{\nu}$ = 3053 (w), 2217 (w), 2198 (m), 1891 (w), 1824 (w), 1760 (w), 1585 (m), 1507 (s), 1409 (m), 1279 (m), 1222 (s), 1150 (s), 1117 (s), 1094 (s), 933 (m), 879 (m), 830 (s), 765 (m), 748 (m), 649 (m), 530 (s). MS (EI, 70 eV): *m/z* (%) = 434 (24), 433 (M^+, 100), 365 (12), 364 (44). HRMS (EI, 70 eV): Calculated for $C_{26}H_{12}F_5N$ (M^+), 433.08844; measured 433.08898.

4,8-Bis(4′-methoxyphenylethynyl)-2-trifluoromethylquinoline (6e)

6e was synthesized according to general procedure A using **4** and 4-methoxyphenylacetylene (**5e**, 1.5 mmol, 198 mg) and was purified via column chromatography (heptane/dichloromethane 4:1). Yield: 208 mg (91%). mp. 152–153 °C. ^{1}H NMR (250 MHz, $CDCl_3$): δ = 3.85 (s, 3H, OMe); 3.87 (s, 3H, OMe); 6.91–6.97 (m, 4H); 7.59–7.71 (m, 5H); 7.88 (s, 1H); 8.02 (dd, ^{3}J = 7.25 Hz, ^{4}J = 1.42 Hz, 1H); 8.34 (dd, ^{3}J = 8.35 Hz, ^{4}J = 1.42 Hz, 1H). ^{13}C NMR (63 MHz, $CDCl_3$): δ = 55.3, 55.4 (OMe); 83.6, 85.3, 97.3, 101.2 (C≡C); 113.6 (C); 114.0, 114.3 (CH); 115.4 (C); 119.5 (q, ^{3}J = 1.98 Hz, CH); 121.5 (q, ^{1}J = 275.5 Hz, CF_3); 125.2 (C); 125.7, 128.4 (CH); 128.5, 132.7 (C); 133.4, 133.7, 134.2 (CH); 147.0 (C); 147.6 (q, ^{2}J = 35.1 Hz, *C*-CF_3); 159.9, 160.8 (C). ^{19}F NMR (282 MHz, $CDCl_3$): δ = -67.51. IR (ATR, cm^{-1}): $\tilde{\nu}$ = 2920 (w), 2846 (w), 2203 (w), 1603 (m), 1565 (m), 1511 (m), 1458 (m), 1409 (m), 1294 (m), 1277 (m), 1248 (m), 1172 (m), 1116 (s), 1026 (s), 896 (m), 831 (s), 763 (s), 641 (m), 533 (s). MS (EI, 70 eV): *m/z* (%) = 458 (30), 457 (M^+, 100), 442 (12), 228 (10).

4,8-Bis(3′-thienylethynyl)-2-trifluoromethylquinoline (6f)

6f was synthesized according to general procedure A using **4** and 3-ethynylthiophene (**5f**, 1.5 mmol, 150 µL) and was purified via column chromatography (heptane/dichloromethane 10:1). Yield: 198 mg (97%). mp. 163–164 °C. ^{1}H NMR (300 MHz, $CDCl_3$): δ = 7.32–7.42 (m, 4H); 7.67–7.76 (m, 3H); 7.90 (s, 1H); 8.05 (dd, ^{3}J = 7.17 Hz, ^{4}J = 1.32 Hz, 1H); 8.35 (dd, ^{3}J = 8.50 Hz, ^{4}J = 1.32 Hz, 1H). ^{13}C NMR (75 MHz, $CDCl_3$): δ = 84.2, 85.9, 92.2, 95.9 (C≡C); 119.8 (q, ^{3}J = 1.83 Hz, CH); 120.7 (C); 121.4 (q, ^{1}J = 275.6 Hz, CF_3); 122.4, 124.9 (C); 125.3, 125.9, 126.2 (CH); 128.4 (C); 128.5, 129.4, 129.8, 130.1, 131.1 (CH); 132.4 (C); 134.6 (CH); 147.0 (C); 147.8 (q, ^{2}J = 35.2 Hz, *C*-CF_3). ^{19}F NMR (282 MHz, $CDCl_3$): δ = -67.52. IR (ATR, cm^{-1}): $\tilde{\nu}$ = 3097 (w), 2922 (w), 2206 (m), 1899 (w), 1829 (w), 1766 (w), 1680 (w), 1587 (m), 1463 (m), 1406 (m), 1376 (m), 1305 (m), 1268 (m), 1170 (m), 1129 (s), 1113 (s), 973 (m), 884 (m), 822 (m), 773 (s), 762 (s), 679 (m), 615 (m), 549 (m). MS (EI, 70 eV): *m/z* (%) = 411 (12), 410 (26), 409 (M^+, 100), 340 (19). HRMS (EI, 70 eV): Calculated for $C_{22}H_{10}S_2F_3N$ (M^+), 409.02013; measured 409.02073.

4,8-Bis(cyclopropylethynyl)-2-trifluoromethylquinoline (6g)

6g was synthesized according to general procedure A using **4** and cyclopropylacetylene (**5g**, 1.5 mmol, 130 µL) and was purified via column chromatography (heptane/dichloromethane 10:1). Yield: 151 mg (93%). mp. 90–91 °C. ^{1}H NMR (300 MHz, $CDCl_3$): δ = 0.94–1.06 (m, 8H); 1.57–1.67 (m, 2H); 7.57 (pt, ^{3}J = 7.84 Hz, 1H); 7.71 (s, 1H); 7.87 (dd, ^{3}J = 7.36 Hz, ^{4}J = 1.32 Hz, 1H); 8.15 (dd, ^{3}J = 8.30 Hz, ^{4}J = 1.32 Hz, 1H). ^{13}C NMR (75 MHz, $CDCl_3$): δ = 0.57, 0.78 (CH); 8.08, 9.42 (CH_2); 71.5, 72.5, 101.7, 106.3 (C≡C); 119.7 (q, ^{3}J = 1.65 Hz, CH); 121.4 (q, ^{1}J = 275.3 Hz, CF_3); 125.3 (CH); 125.4 (C); 128.2 (CH); 128.9, 133.2 (C); 134.4 (CH); 147.2 (C); 147.5 (q, ^{2}J = 35.0 Hz, *C*-CF_3). ^{19}F NMR (282 MHz, $CDCl_3$): δ = -67.57. IR (ATR, cm^{-1}): $\tilde{\nu}$ = 3013 (w), 2924 (w), 2853 (w), 2218 (m), 1741 (w), 1588 (m), 1502 (m), 1466 (m), 1409 (m), 1305

(m), 1270 (m), 1233 (m), 1174 (m), 1127 (s), 1106 (s), 1027 (m), 949 (m), 874 (s), 811 (m), 766 (s), 742 (m), 707 (m), 645 (m). MS (EI, 70 eV): *m/z* (%) = 326 (19), 325 (M^+, 100), 297 (12), 296 (20), 257 (21), 256 (93), 255 (66), 254 (56), 253 (13), 252 (14), 241 (12), 229 (13), 228 (37), 227 (13), 226 (15), 215 (11). HRMS (EI, 70 eV): Calculated for $C_{20}H_{14}F_3N$ (M^+), 325.10729; measured 325.10682.

4,6-Bis(phenylethynyl)-2-trifluoromethylquinoline (9a)

9a was synthesized according to general procedure A using **8** and phenylacetylene (**5a**, 1.5 mmol, 165 µL) and was purified via column chromatography (heptane/dichloromethane 10:1). Yield: 152 mg (77%). mp. 110–111 °C. ^{1}H NMR (250 MHz, $CDCl_3$): δ = 7.38–7.41 (m, 3H); 7.45-7.46 (m, 3H); 7.60–7.64 (m, 2H); 7.69–7.73 (m, 2H); 7.90 (s, 1H); 7.93 (dd, ^3J = 8.82 Hz, ^4J = 1.73 Hz, 1H); 8.19 (d, ^3J = 8.82 Hz, 1H); 8.53 (d, ^4J = 1.73 Hz, 1H). ^{13}C NMR (63 MHz, $CDCl_3$): δ = 84.2, 88.7, 92.5, 101.0 (C≡C); 119.9 (q, ^3J = 1.83 Hz, CH); 121.3 (q, ^1J = 275.5 Hz, CF_3); 121.6, 122.6, 124.4, 128.2 (C); 128.5, 128.7, 128.9, 129.9, 130.5 (CH); 131.6 (C); 131.8, 132.1, 133.8 (CH); 146.5 (C); 147.8 (q, ^2J = 34.9 Hz, *C*-CF_3). ^{19}F NMR (282 MHz, $CDCl_3$): δ = -67.66. IR (ATR, cm^{-1}): $\tilde{\nu}$ = 3054 (w), 2922 (w), 2851 (w), 2211 (m), 1954 (w), 1876 (w), 1819 (w), 1764 (w), 1578 (m), 1491 (m), 1443 (m), 1392 (m), 1365 (m), 1317 (m), 1278 (m), 1173 (m), 1120 (s), 1093 (s), 918 (m), 888 (m), 844 (m), 752 (s), 684 (s), 542 (m), 526 (m). MS (EI, 70 eV): *m/z* (%) = 398 (29), 397 (M^+, 100), 325 (10). HRMS (EI, 70 eV): Calculated for $C_{26}H_{14}F_3N$ (M^+), 397.10729; measured 397.10747.

4,6-Bis(4'-*tert*-butylphenylethynyl)-2-trifluoromethylquinoline (9b)

9b was synthesized according to general procedure A using **8** and 4-*tert*-butylphenylacetylene (**5b**, 1.5 mmol, 270 µL) and was purified via column chromatography (heptane/dichloromethane 10:1). Yield: 215 mg (84%). mp. 94–95 °C. ^{1}H NMR (300 MHz, $CDCl_3$): δ = 1.36 (s, 9H, *t*-Bu); 1.38 (s, 9H, *t*Bu); 7.42 (d, ^3J = 8.49 Hz, 2H); 7.48 (d, ^3J = 8.49 Hz, 2H); 7.56 (d, ^3J = 8.49 Hz, 2H); 7.66 (d, ^3J = 8.49 Hz, 2H); 7.89 (s, 1H); 7.93 (dd, ^3J = 8.68 Hz, ^4J = 1.89 Hz, 1H); 8.18 (d, ^3J = 8.68 Hz, 1H); 8.53 (d, ^4J = 1.89 Hz, 1H). ^{13}C NMR (63 MHz, $CDCl_3$): δ = 31.1, 31.2 (CH_3); 34.9, 35.0 (*C*(Me)$_3$); 83.8, 88.2, 92.8, 101.4 (C≡C); 118.6, 119.6 (C); 119.7 (q, ^3J = 2.27 Hz, CH); 121.3 (q, ^1J = 275.4 Hz, CF_3); 124.6 (C); 125.5, 125.7 (CH); 128.3 (C); 128.9, 130.4, 131.6 (CH); 131.8 (C); 131.9, 133.9 (CH); 146.5 (C); 147.7 (q, ^2J = 34.8 Hz, *C*-CF_3); 152.3, 153.5 (C). ^{19}F NMR (282 MHz, $CDCl_3$): δ = -67.63. IR (ATR, cm^{-1}): $\tilde{\nu}$ = 2959 (m), 2867 (w), 2207 (m), 1912 (w), 1579 (m), 1505 (m), 1463 (m), 1392 (m), 1363 (m), 1278 (m), 1184 (m), 1132 (s), 1091 (s), 920 (m), 878 (m), 832 (s), 711 (m), 559 (s). MS (EI, 70 eV): *m/z* (%) = 510 (34), 509 (M^+, 94), 495 (35), 494 (100), 438 (11), 240 (23), 211 (18), 57 (19), 41 (20). HRMS (EI, 70 eV): Calculated for $C_{34}H_{30}F_3N$ (M^+), 509.23249; measured 509.23344.

4,6-Bis(4'-cyanophenylethynyl)-2-trifluoromethylquinoline (9c)

9c was synthesized according to general procedure A using **8** and 4-cyanophenylacetylene (**5c**, 1.5 mmol, 191 mg). During aqueous work-up the product precipitates. The solid was filtered and washed with dichloromethane. Product **9c** was insoluble even at high temperatures, thus making an NMR impossible. Yield: 43 mg (20%). mp. 242–243 °C. IR (ATR, cm^{-1}): $\tilde{\nu}$ = 3078 (w), 2226 (m), 1925 (w), 1731 (w), 1673 (w), 1601 (m), 1502 (m), 1396 (m), 1319 (w), 1286 (m), 1173 (m), 1125 (s), 1092 (s), 971 (m), 834 (s), 720 (m), 614 (m), 552 (s). MS (EI, 70 eV): *m/z* (%) = 448 (32), 447 (M^+, 100), 223 (10). HRMS (EI, 70 eV): Calculated for $C_{28}H_{12}F_3N_3$ (M^+), 447.09778; measured 447.09839.

4,6-Bis(4'-fluorophenylethynyl)-2-trifluoromethylquinoline (9d)

9d was synthesized according to general procedure A using **8** and 4-fluorophenylacetylene (**5d**, 1.5 mmol, 180 mg) and was purified via column chromatography (heptane/dichloromethane 10:1). Yield: 183 mg (85%). mp. 197–198 °C. ^{1}H NMR (300 MHz, $CDCl_3$): δ = 7.07–7.19 (m, 4H); 7.57–7.61 (m, 2H); 7.67–7.72 (m, 2H); 7.88 (s, 1H); 7.91 (dd, ^3J = 8.88 Hz, ^4J = 1.89 Hz, 1H); 8.19 (d, ^3J = 8.88 Hz, 1H); 8.48 (d, ^4J = 1.89 Hz, 1H). ^{13}C NMR (75 MHz, $CDCl_3$): δ = 83.9, 88.4, 91.5, 99.9 (C≡C); 115.9 (d, ^2J = 22.0 Hz, CH); 116.2 (d, ^2J = 22.5 Hz, CH); 117.7 (d, ^4J = 3.85 Hz, C); 118.6 (d, ^4J = 3.85 Hz, C); 120.0 (q, ^3J = 2.20 Hz, CH); 121.3 (q, ^1J = 275.6 Hz, CF_3); 124.3, 128.1 (C); 128.8, 130.7 (CH); 131.5 (C); 133.7 (d, ^3J = 8.80 Hz, CH); 133.8 (CH); 134.2 (d, ^3J = 8.80 Hz, CH); 146.6 (C); 147.9 (q, ^2J = 35.2 Hz, *C*-CF_3); 162.9 (d, ^1J = 250.8 Hz, CF); 163.5 (d, ^1J = 252.5 Hz, CF). ^{19}F NMR (282 MHz, $CDCl_3$): δ = -109.53 (CF); -107.75 (CF); -67.71 (CF_3). IR (ATR, cm^{-1}): $\tilde{\nu}$ = 3056 (w), 2922 (w), 2852 (w), 2214 (m), 1577 (m), 1508 (s), 1397 (m), 1317 (m), 1278 (m), 1218 (m), 1174 (m), 1134 (s), 1088 (m), 917 (m), 888 (m), 877 (m), 832 (s), 749 (m), 729 (m), 639 (m), 530 (s). MS (EI, 70 eV): *m/z* (%) = 434 (28), 433 (M^+, 100), 361 (8), 216 (8). HRMS (EI, 70 eV): Calculated for $C_{26}H_{12}F_5N$ (M^+), 433.08844; measured 433.08831.

4,6-Bis(4'-methoxyphenylethynyl)-2-trifluoromethylquinoline (9e)

9e was synthesized according to general procedure A using **8** and 4-methoxyphenylacetylene (**5e**, 1.5 mmol, 198 mg) and was purified via column chromatography (heptane/dichloromethane 4:1). Yield: 192 mg (84%). mp. 131–132 °C. ^{1}H NMR (300 MHz, $CDCl_3$): δ = 3.85 (s, 3H, OMe); 3.87 (s, 3H, OMe); 6.92 (d, ^3J = 8.88 Hz, 2H); 6.97 (d, ^3J = 8.88 Hz, 2H); 7.55 (d, ^3J = 8.67 Hz, 2H); 7.65 (d, ^3J = 8.67 Hz, 2H); 7.85 (s, 1H); 7.89 (dd, ^3J = 8.88 Hz, ^4J = 1.89 Hz, 1H); 8.15 (d, ^3J = 8.88 Hz, 1H); 8.49 (d, ^4J = 1.89 Hz, 1H). ^{13}C NMR (75 MHz, $CDCl_3$): δ = 55.3, 55.4 (OMe); 83.5, 87.7, 92.7, 101.6 (C≡C); 113.6 (C); 114.1, 114.4 (CH); 114.7 (C); 119.6 (q, ^3J = 2.02 Hz, CH); 121.4 (q, ^1J = 275.6 Hz, CF_3); 124.6, 128.3 (C); 128.6, 130.4 (CH); 132.0 (C); 133.4, 133.8, 133.9 (CH); 146.5 (C); 147.6 (q, ^2J = 34.8 Hz, *C*-CF_3); 160.1, 160.9 (C). ^{19}F NMR (282 MHz, $CDCl_3$): δ = -67.66. IR (ATR, cm^{-1}): $\tilde{\nu}$ = 2934 (w), 2840 (w), 2544 (w), 2202 (m), 2051 (w), 1980 (w), 1891 (w), 1742 (w), 1600 (m), 1573 (m), 1509 (s), 1464 (m), 1392 (m), 1317 (m),

1293 (m), 1276 (m), 1247 (s), 1169 (s), 1132 (s), 1089 (s), 1022 (s), 917 (m), 889 (m), 871 (m), 825 (s), 728 (m), 615 (m), 531 (s). MS (EI, 70 eV): *m/z* (%) = 458 (31), 457 (M^+, 100), 229 (11). HRMS (EI, 70 eV): Calculated for $C_{28}H_{18}F_3O_2N$ (M^+), 457.12841; measured 457.12837.

4,6-Bis(3'-thienylethynyl)-2-trifluoromethylquinoline (9f)

9f was synthesized according to general procedure A using **8** and 3-ethynylthiophene (**5f**, 1.5 mmol, 150 µL) and was purified via column chromatography (heptane/dichloromethane 10:1). Yield: 174 mg (85%). mp. 157–158 °C. ^{1}H NMR (300 MHz, $CDCl_3$): δ = 7.26–7.42 (m, 4H); 7.64 (dd, ^4J = 3.02 Hz, ^4J = 1.32 Hz, 1H); 7.79 (dd, ^4J = 3.02 Hz, ^4J = 1.32 Hz, 1H); 7.87 (s, 1H); 7.90 (dd, ^3J = 8.67 Hz, ^4J = 1.89 Hz, 1H); 8.17 (d, ^3J = 8.67 Hz, 1H); 8.49 (d, ^4J = 1.89 Hz, 1H). ^{13}C NMR (63 MHz, $CDCl_3$): δ = 83.9, 87.7, 88.3, 96.2 (C≡C); 119.8 (q, ^3J = 2.14 Hz, CH); 120.7 (C); 121.3 (q, ^1J = 275.1 Hz, CF_3); 121.6, 124.3 (C); 125.7, 126.1 (CH); 128.2 (C); 128.8, 129.7, 129.8, 129.9, 130.5, 131.2 (CH); 131.7 (C); 133.7 (CH); 146.5 (C); 147.8 (q, ^2J = 35.2 Hz, *C*-CF_3). ^{19}F NMR (282 MHz, $CDCl_3$): δ = -67.68. IR (ATR, cm^{-1}): $\tilde{\nu}$ = 3100 (w), 2922 (w), 2200 (m), 1959 (w), 1760 (w), 1578 (m), 1490 (m), 1362 (m), 1317 (m), 1266 (m), 1174 (m), 1123 (s), 1093 (s), 944 (m), 883 (m), 846 (m), 778 (s), 716 (m), 620 (s), 555 (m). MS (EI, 70 eV): *m/z* (%) = 411 (12), 410 (26), 409 (M^+, 100). HRMS (EI, 70 eV): Calculated for $C_{22}H_{10}S_2F_3N$ (M^+), 409.02013; measured 409.01962.

4,6-Bis(cyclopropylethynyl)-2-trifluoromethylquinoline (9g)

9g was synthesized according to general procedure A using **8** and cyclopropylacetylene (**5g**, 1.5 mmol, 130 µL) and was purified via column chromatography (heptane/dichloromethane 10:1). Yield: 132 mg (81%). mp. 103–104 °C. ^{1}H NMR (300 MHz, $CDCl_3$): δ = 0.88–0.96 (m, 4H); 0.99–1.07 (m, 4H); 1.49–1.57 (m, 1H); 1.60–1.69 (m, 1H); 7.68 (s, 1H); 7.72 (dd, ^3J = 8.70 Hz, ^4J = 1.89 Hz, 1H); 8.05 (d, ^3J = 8.70 Hz, 1H); 8.24 (d, ^4J = 1.89 Hz, 1H). ^{13}C NMR (75 MHz, $CDCl_3$): δ = 0.34, 0.68 (CH); 8.86, 9.49 (CH_2); 71.4, 75.5, 96.9, 106.7 (C≡C); 119.7 (q, ^3J = 2.38 Hz, CH); 121.4 (q, ^1J = 275.2 Hz, CF_3); 124.8, 128.7 (C); 128.7, 130.2 (CH); 132.3 (C); 133.9 (CH); 146.2 (C); 147.4 (q, ^2J = 34.7 Hz, *C*-CF_3). ^{19}F NMR (282 MHz, $CDCl_3$): δ = -67.75. IR (ATR, cm^{-1}): $\tilde{\nu}$ = 3013 (w), 2224 (m), 1580 (m), 1491 (m), 1403 (m), 1342 (m), 1316 (m), 1257 (m), 1131 (s), 1090 (m), 1029 (m), 976 (m), 900 (m), 876 (s), 842 (m), 810 (m), 689 (m). MS (EI, 70 eV): *m/z* (%) = 326 (22), 325 (M^+, 100), 289 (10), 240 (19). HRMS (EI, 70 eV): Calculated for $C_{20}H_{14}F_3N$ (M^+), 325.10729; measured 325.10708.

3,4,8-Tris(phenylethynyl)-2-trifluoromethylquinoline (12a)

12a was synthesized according to general procedure B using **11** and phenylacetylene (**5a**, 2.0 mmol, 220 µL) and was purified via column chromatography (heptane/dichloromethane 15:1). Yield: 187 mg (75%). mp. 126–127 °C. ^{1}H NMR (300 MHz, $CDCl_3$): δ = 7.38–7.47 (m, 9H); 7.63–7.74 (m, 7H); 8.01 (dd, ^3J = 7.18 Hz, ^4J = 1.13 Hz, 1H); 8.34 (dd, ^3J = 8.31 Hz, ^4J = 1.13 Hz, 1H). ^{13}C NMR (75 MHz, $CDCl_3$): δ =

83.4, 84.3, 86.1, 97.3, 101.5, 105.1 (C≡C); 116.9 (C); 121.2 (q, ^{1}J = 276.5 Hz, CF_3); 121.9, 122.5, 123.2, 125.0 (C); 126.0 (CH); 127.7 (C); 128.4, 128.5, 128.7, 129.2, 129.3, 129.9, 131.8, 132.0, 132.1, 134.5 (CH); 137.7, 144,3 (C); 147.2 (q, ^{2}J = 33.5 Hz, *C*-CF_3). ^{19}F NMR (282 MHz, $CDCl_3$): δ = -66.36. IR (ATR, cm^{-1}): $\tilde{\nu}$ = 3052 (w), 2923 (w), 2852 (w), 2203 (w), 1955 (w), 1882 (w), 1732 (w), 1672 (w), 1595 (m), 1493 (s), 1468 (m), 1441 (m), 1332 (m), 1294 (m), 1186 (m), 1114 (s), 1068 (m), 1008 (m), 931 (m), 750 (s), 684 (s), 513 (s). MS (EI, 70 eV): *m/z* (%) = 498 (36), 497 (M^+, 100), 428 (10), 426 (13). HRMS (EI, 70 eV): Calculated for $C_{34}H_{18}F_3N$ (M^+), 497.13859; measured 497.13817.

3,4,8-Tris(4'-*tert*-butylphenylethynyl)-2-trifluoromethylquinoline (12b)

12b was synthesized according to general procedure B using **11** and 4-*tert*-butylphenylacetylene (**5b**, 2.0 mmol, 360 µL) and was purified via column chromatography (heptane/dichloromethane 15:1). Yield: 230 mg (70%). mp. 175–176 °C. ^{1}H NMR (300 MHz, $CDCl_3$): δ = 1.36 (s, 18H, 2x *t*-Bu); 1.38 (s, 9H, *t*-Bu); 7.42–7.48 (m, 6H); 7.59–7.69 (m, 7H); 8.00 (dd, ^{3}J = 7.17 Hz, ^{4}J = 1.13 Hz, 1H); 8.34 (dd, ^{3}J = 8.31 Hz, ^{4}J = 1.13 Hz, 1H). ^{13}C NMR (75 MHz, $CDCl_3$): δ = 31.1, 31.2, 31.3 (CH_3); 34.9, 35.0, 35.1 (*C*(Me)$_3$); 82.9, 84.0, 85.6, 97.5, 101.7, 105.5 (C≡C); 117.0, 119.0, 119.6, 120.7 (C); 121.3 (q, ^{1}J = 276.7 Hz, CF_3); 125.2 (C); 125.4, 125.5, 125.7, 125.8 (CH); 127.9 (C); 129.1, 131.6, 131.7, 131.9, 134.3 (CH); 134.8, 144.3 (C); 147.3 (q, ^{2}J = 33.4 Hz, *C*-CF_3); 152.0, 152.7, 153.5 (C). ^{19}F NMR (282 MHz, $CDCl_3$): δ = -66.41. IR (ATR, cm^{-1}): $\tilde{\nu}$ = 2961 (m), 2204 (m), 1901 (w), 1603 (w), 1554 (w), 1514 (m), 1467 (m), 1363 (m), 1266 (m), 1181 (s), 1128 (s), 1016 (m), 1006 (m), 936 (m), 830 (s), 773 (s), 705 (m), 627 (m), 557 (s). MS (EI, 70 eV): *m/z* (%) = 667 (18), 666 (65), 665 (M^+, 100), 651 (30), 650 (70), 318 (10). HRMS (EI, 70 eV): Calculated for $C_{46}H_{42}F_3N$ (M^+), 665.32639; measured 665.32484.

3,4,8-Tris(4'-cyanophenylethynyl)-2-trifluoromethylquinoline (12c)

12c was synthesized according to general procedure B using **11** and 4-cyanophenylacetylene (**5c**, 2.0 mmol, 254 mg). During aqueous work-up the product precipitates. The solid was filtered and washed with dichloromethane. Product **12c** was insoluble even at high temperatures, thus making an NMR impossible. Yield: 186 mg (65%). mp. 307–308 °C. IR (ATR, cm^{-1}): $\tilde{\nu}$ = 3091 (w), 2935 (w), 2674 (w), 2490 (w), 2223 (s), 1918 (w), 1740 (w), 1601 (s), 1500 (m), 1391 (m), 1334 (m), 1301 (m), 1254 (m), 1185 (s), 1124 (s), 1008 (m), 933 (m), 834 (s), 771 (s), 725 (m), 639 (m), 550 (s). MS (EI, 70 eV): *m/z* (%) = 573 (26), 572 (M^+, 67), 527 (13), 525 (13), 447 (18), 444 (25), 443 (100), 429 (16), 308 (10), 256 (13), 229 (15), 185 (17), 183 (20), 152 (17). HRMS (EI, 70 eV): Calculated for $C_{37}H_{15}F_3N_4$ ($[M+H]^+$), 573.13216; measured 573.13294.

3,4,8-Tris(4'-fluorophenylethynyl)-2-trifluoromethylquinoline (12d)

12d was synthesized according to general procedure B using **11** and 4-fluorophenylacetylene (**5d**, 2.0 mmol, 240 mg) and was purified via column chromatography (heptane/dichloromethane 15:1).

Yield: 175 mg (64%). mp. 190–191 °C. ^{1}H NMR (300 MHz, $CDCl_3$): δ = 7.06–7.14 (m, 6H); 7.54–7.67 (m, 7H); 7.94 (dd, ^3J = 7.18 Hz, ^4J = 1.32 Hz, 1H); 8.22 (dd, ^3J = 8.31 Hz, ^4J = 1.32 Hz, 1H). ^{13}C NMR (75 MHz, $CDCl_3$): δ = 83.0, 84.0, 85.4, 96.3, 100.3, 103.9 (C≡C); 115.7 (d, ^2J = 22.0 Hz, CH); 116.0 (d, ^2J = 22.0 Hz, CH); 116.2 (d, ^2J = 22.5 Hz, CH); 116.7 (C); 117.9 (d, ^4J = 3.85 Hz, C); 118.5 (d, ^4J = 3.85 Hz, C); 119.3 (d, ^4J = 3.30 Hz, C); 121.2 (q, ^1J = 276.7 Hz, CF_3); 124.9 (C); 125.8 (CH); 127.6 (C); 129.2 (CH); 133.7 (d, ^3J = 8.25 Hz, CH); 133.9 (d, ^3J = 8.80 Hz, CH); 134.0 (d, ^3J = 8.80 Hz, CH); 134.4 (CH); 134.5, 144.2 (C); 147.1 (q, ^2J = 33.3 Hz, *C*-CF_3); 162.8 (d, ^1J = 250.3 Hz, CF); 163.2 (d, ^1J = 251.4 Hz, CF); 163.5 (d, ^1J = 252.5 Hz, CF). ^{19}F NMR (282 MHz, $CDCl_3$): δ = -110.08 (CF); -108.62 (CF); -107.49 (CF); -66.41 (CF_3). IR (ATR, cm^{-1}): $\tilde{\nu}$ = 3053 (w), 2925 (w), 2560 (w), 2206 (m), 1888 (w), 1598 (m), 1551 (w), 1505 (s), 1333 (m), 1222 (s), 1191 (m), 1151 (s), 1114 (s), 1090 (m), 1011 (m), 933 (m), 827 (s), 800 (m), 768 (s), 740 (m), 689 (m), 600 (m), 526 (s). MS (EI, 70 eV): *m/z* (%) = 552 (38), 551 (M^+, 100), 480 (10), 275 (10). HRMS (EI, 70 eV): Calculated for $C_{34}H_{15}F_6N$ (M^+), 551.11032; measured 551.11194.

3,4,8-Tris(4′-methoxyphenylethynyl)-2-trifluoromethylquinoline (12e)

12e was synthesized according to general procedure B using **11** and 4-methoxyphenylacetylene (**5e**, 2.0 mmol, 264 mg) and was purified via column chromatography (heptane/dichloromethane 2:1). Yield: 204 mg (70%). mp. 119–120 °C. ^{1}H NMR (300 MHz, $CDCl_3$): δ = 3.85 (s, 6H, 2x OMe); 3.86 (s, 3H, OMe); 6.90–6.94 (m, 6H); 7.54–7.68 (m, 7H); 7.94 (dd, ^3J = 7.38 Hz, ^4J = 1.32 Hz, 1H); 8.28 (dd, ^3J = 8.31 Hz, ^4J = 1.32 Hz, 1H). ^{13}C NMR (75 MHz, $CDCl_3$): δ = 55.2, 55.3, 55.4 (OMe); 82.6, 83.8, 85.2, 97.4, 101.5, 105.6 (C≡C); 114.0, 114.2, 114.3 (CH); 114.7, 115.4, 116.9 (C); 121.4 (q, ^1J = 276.7 Hz, CF_3); 125.2 (C); 125.6 (CH); 127.8 (C); 128.9, 133.4, 133.5, 133.7, 133.8 (CH); 134.6, 144.1 (C); 147.0 (q, ^2J = 33.6 Hz, *C*-CF_3); 159.9, 160.4, 160.9 (C). ^{19}F NMR (282 MHz, $CDCl_3$): δ = -66.42. IR (ATR, cm^{-1}): $\tilde{\nu}$ = 3063 (w), 3004 (w), 2931 (w), 2837 (w), 2542 (w), 2199 (m), 2050 (w), 1886 (w), 1602 (m), 1566 (w), 1509 (s), 1455 (m), 1440 (m), 1415 (w), 1363 (w), 1334 (w), 1291 (m), 1247 (s), 1168 (m), 1130 (s), 1029 (s), 934 (m), 824 (s), 768 (s), 739 (m), 689 (m), 640 (m), 530 (s). MS (EI, 70 eV): *m/z* (%) = 589 (30), 588 (63), 587 (M^+, 100), 457 (10), 149 (11), 99 (20), 97 (15), 85 (12), 83 (14), 71 (18), 69 (19), 57 (32), 56 (11), 55 (19), 44 (81), 43 (19), 41 (17). HRMS (EI, 70 eV): Calculated for $C_{37}H_{24}F_3O_3N$ (M^+), 587.17028; measured 587.17191.

3,4,8-Tris(3′-thienylethynyl)-2-trifluoromethylquinoline (12f)

12f was synthesized according to general procedure B using **11** and 3-ethynylthiophene (**5f**, 2.0 mmol, 200 µL) and was purified via column chromatography (heptane/dichloromethane 15:1). Yield: 181 mg (70%). mp. 155–156 °C. ^{1}H NMR (300 MHz, $CDCl_3$): δ = 7.27–7.41 (m, 6H); 7.65–7.76 (m, 4H); 7.98 (dd, ^3J = 7.17 Hz, ^4J = 1.32 Hz, 1H); 8.28 (dd, ^3J = 8.31 Hz, ^4J = 1.32 Hz, 1H). ^{13}C NMR (63 MHz, $CDCl_3$): δ = 82.9, 84.1, 85.7, 92.4, 96.6, 100.3 (C≡C); 116.9, 121.1 (C); 121.2 (q, ^1J = 276.4 Hz, CF_3); 121.7, 122.3,

124.6 (C); 125.4, 125.9, 126.2 (CH); 127.7 (C); 129.1, 129.5, 129.7, 129.8, 130.1, 130.2, 131.1, 134.4 (CH); 134.7, 144.2 (C); 146.8 (q, ^{2}J = 33.4 Hz, *C*-CF_3). ^{19}F NMR (282 MHz, $CDCl_3$): δ = -66.43. IR (ATR, cm^{-1}): $\tilde{\nu}$ = 3106 (w), 2922 (m), 2207 (m), 1524 (w), 1470 (m), 1369 (m), 1296 (m), 1177 (m), 1124 (s), 1078 (m), 972 (m), 868 (m), 779 (s), 769 (s), 687 (m), 620 (s), 554 (m). MS (EI, 70 eV): *m/z* (%) = 517 (18), 516 (32), 515 (M^+, 100). HRMS (ESI): Calculated for $C_{28}H_{12}S_3F_3N$ ($[M+H]^+$), 516.01567; measured 516.01564.

3,4,8-Tris(cyclopropylethynyl)-2-trifluoromethylquinoline (12g)

12g was synthesized according to general procedure B using **11** and cyclopropylacetylene (**5g**, 2.0 mmol, 170 µL) and was purified via column chromatography (heptane/dichloromethane 15:1). Yield: 132 mg (68%). mp. 120–121 °C. ^{1}H NMR (300 MHz, $CDCl_3$): δ = 0.91–1.12 (m, 12H); 1.54–1.72 (m, 3H); 7.53 (pt, ^{3}J = 7.84 Hz, 1H); 7.79 (dd, ^{3}J = 7.36 Hz, ^{4}J = 1.32 Hz, 1H); 8.08 (dd, ^{3}J = 8.31 Hz, ^{4}J = 1.32 Hz, 1H). ^{13}C NMR (63 MHz, $CDCl_3$): δ = 0.76, 0.92, 1.00 (CH); 9.08, 9.27, 9.92 (CH_2); 69.7, 71.3, 72.4, 101.8, 105.9, 101.4 (C≡C); 117.3 (C); 121.2 (q, ^{1}J = 276.6 Hz, CF_3); 125.2 (CH); 125.3, 128.2 (C); 128.6, 134.0 (CH); 135.5, 144.1 (C); 147.3 (q, ^{2}J = 32.9 Hz, *C*-CF_3). ^{19}F NMR (282 MHz, $CDCl_3$): δ = -66.71. IR (ATR, cm^{-1}): $\tilde{\nu}$ = 3095 (w), 3010 (w), 2923 (w), 2852 (w), 2219 (m), 1590 (w), 1474 (m), 1368 (m), 1294 (m), 1206 (m), 1162 (m), 1120 (s), 1028 (m), 947 (m), 873 (m), 811 (m), 769 (s), 692 (m), 565 (m). MS (EI, 70 eV): *m/z* (%) = 390 (27), 389 (M^+, 100), 388 (18), 321 (19), 320 (65), 319 (19), 318 (24), 317 (14), 316 (12), 305 (16), 304 (43), 303 (28), 302 (23), 292 (16), 291 (25), 290 (25), 289 (21), 279 (19), 278 (37), 277 (10), 276 (10), 265 (12), 263 (10), 145 (15). HRMS (ESI): Calculated for $C_{25}H_{18}F_3N$ ($[M+H]^+$), 390.14641; measured 390.14629.

4. NMR-Spectra (^{1}H-, ^{13}C-, dept, and ^{19}F-NMR)

(The spectra are listed in the order ^{1}H-NMR, ^{13}C-NMR, dept, and lastly ^{19}F-NMR.)

3 - 8-Bromo-2-trifluoromethyl-4-quinolinone

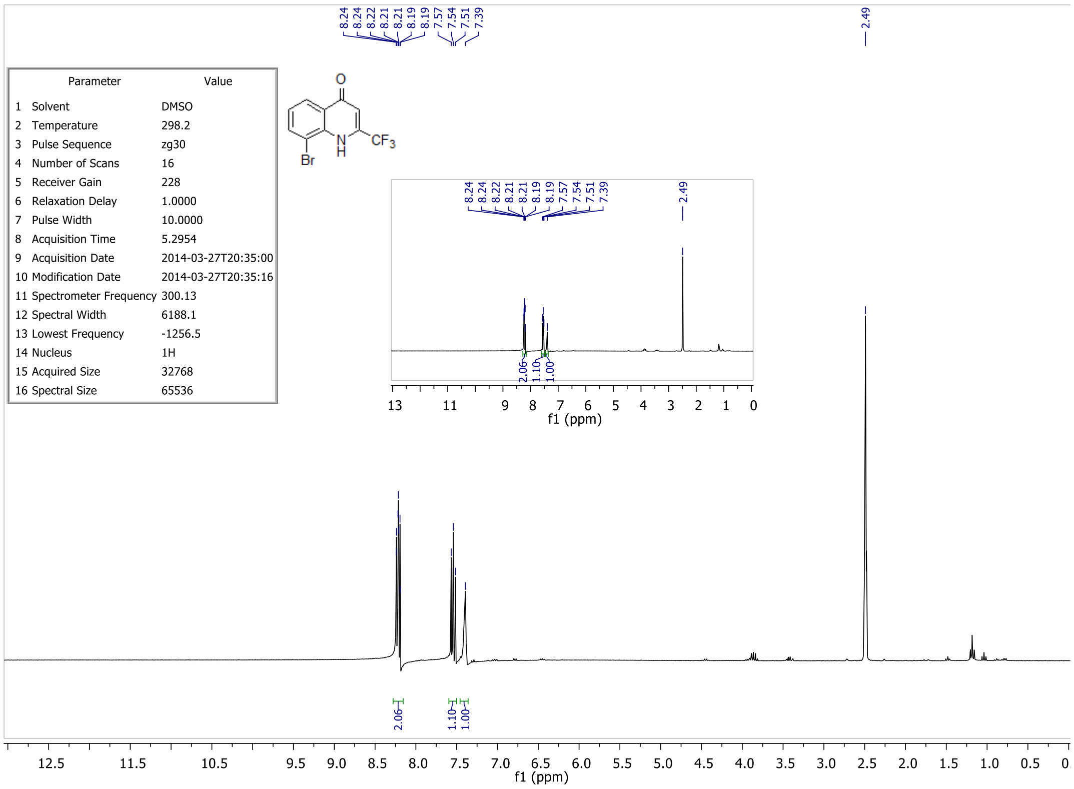

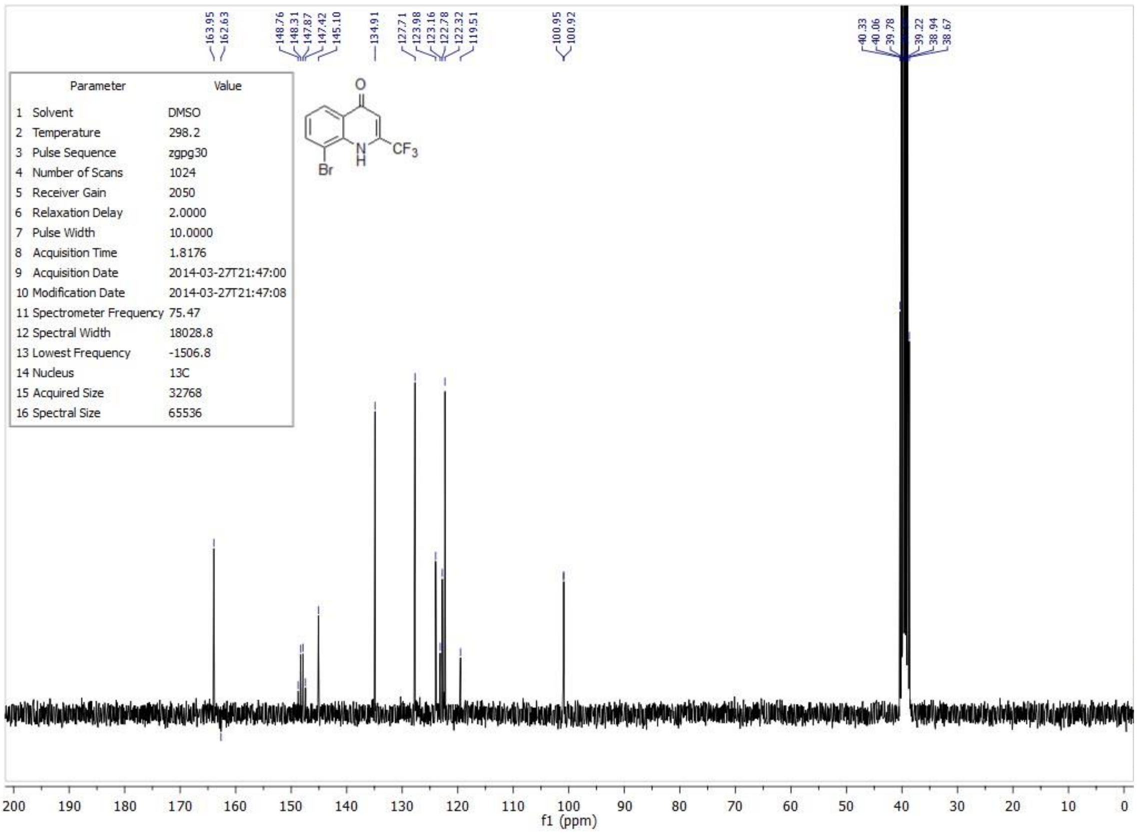

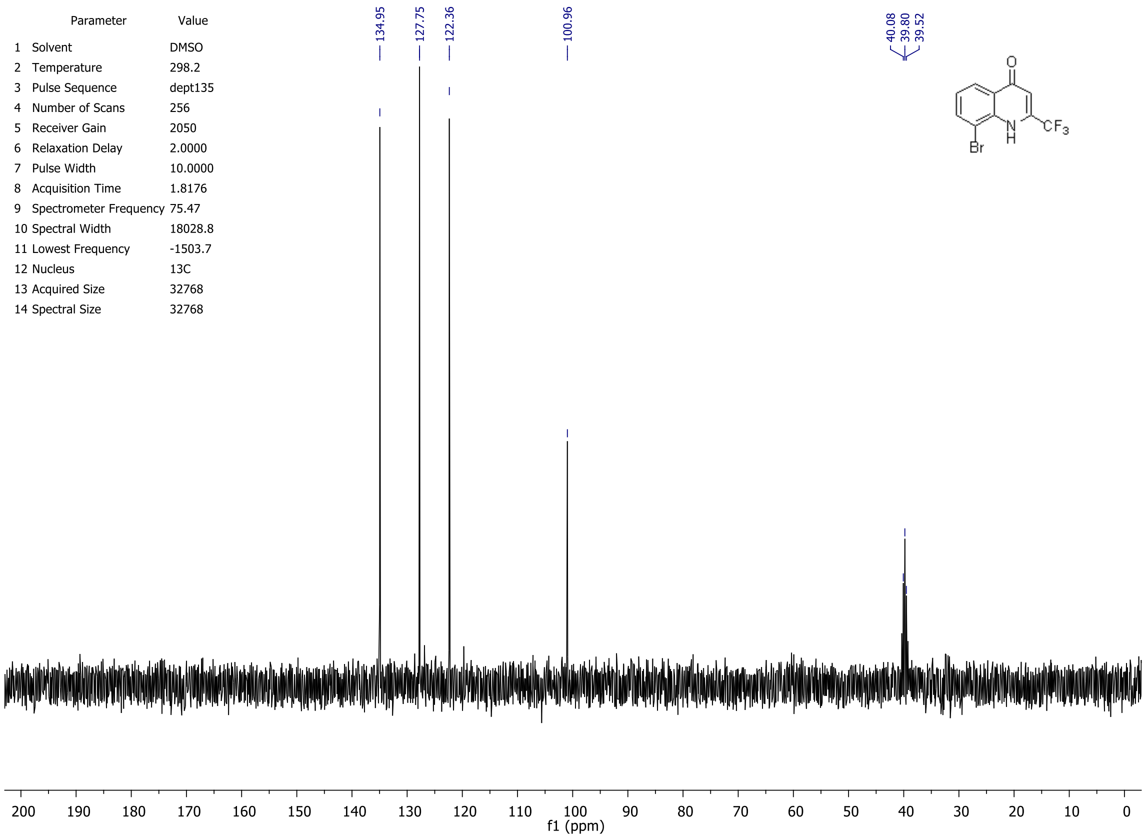
Parameter Value
1 Solvent DMSO
2 Temperature 298.2
3 Pulse Sequence dept135
4 Number of Scans 256
5 Receiver Gain 2050
6 Relaxation Delay 2.0000
7 Pulse Width 10.0000
8 Acquisition Time 1.8176
9 Spectrometer Frequency 75.47
10 Spectral Width 18028.8
11 Lowest Frequency -1503.7
12 Nucleus 13C
13 Acquired Size 32768
14 Spectral Size 32768
134.95
127.75
122.36
100.96
40.08
39.80
39.52
O
N
H
CF3
Br
200 190 180 170 160 150 140 130 120 110 100 90 80 70 60 50 40 30 20 10 0
f1 (ppm)

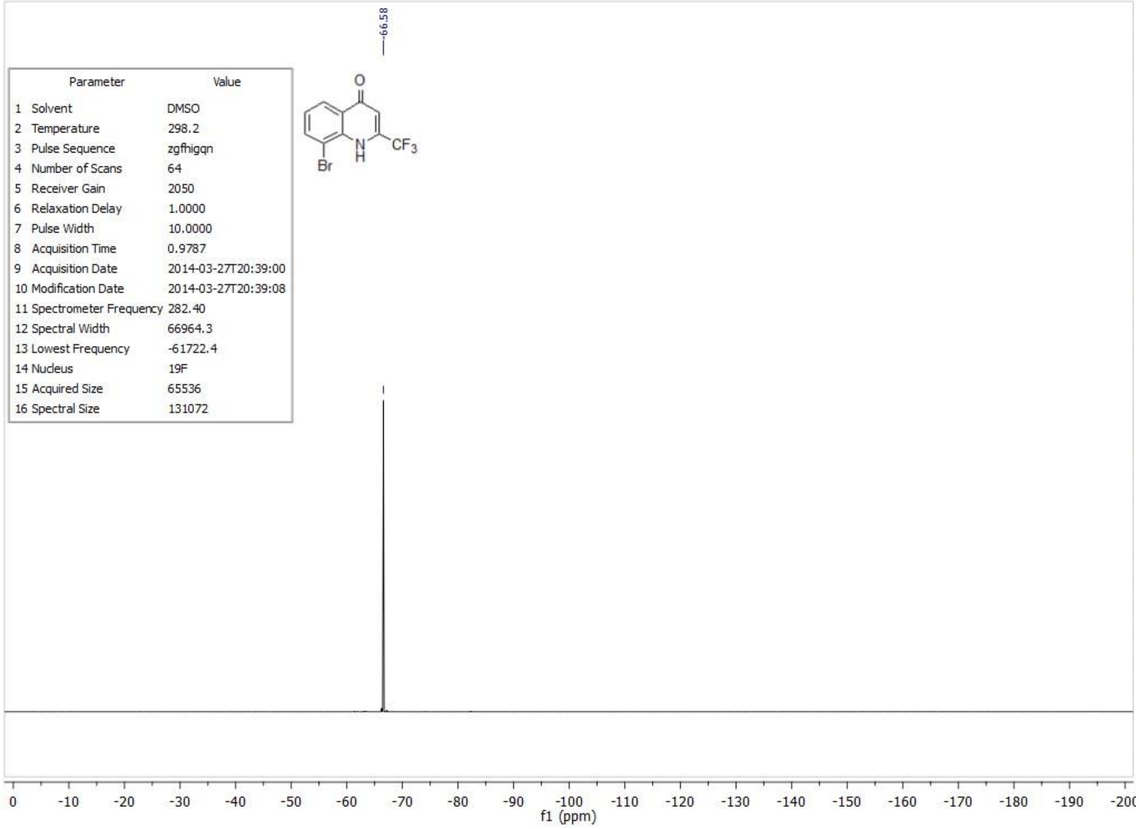
-66.58
Parameter Value
1 Solvent DMSO
2 Temperature 298.2
3 Pulse Sequence zgfhigqn
4 Number of Scans 64
5 Receiver Gain 2050
6 Relaxation Delay 1.0000
7 Pulse Width 10.0000
8 Acquisition Time 0.9787
9 Acquisition Date 2014-03-27T20:39:00
10 Modification Date 2014-03-27T20:39:08
11 Spectrometer Frequency 282.40
12 Spectral Width 66964.3
13 Lowest Frequency -61722.4
14 Nucleus 19F
15 Acquired Size 65536
16 Spectral Size 131072
O
N
H
CF3
Br
0 -10 -20 -30 -40 -50 -60 -70 -80 -90 -100 -110 -120 -130 -140 -150 -160 -170 -180 -190 -200
f1 (ppm)

4 - 4,8-Dibromo-2-trifluoromethylquinoline

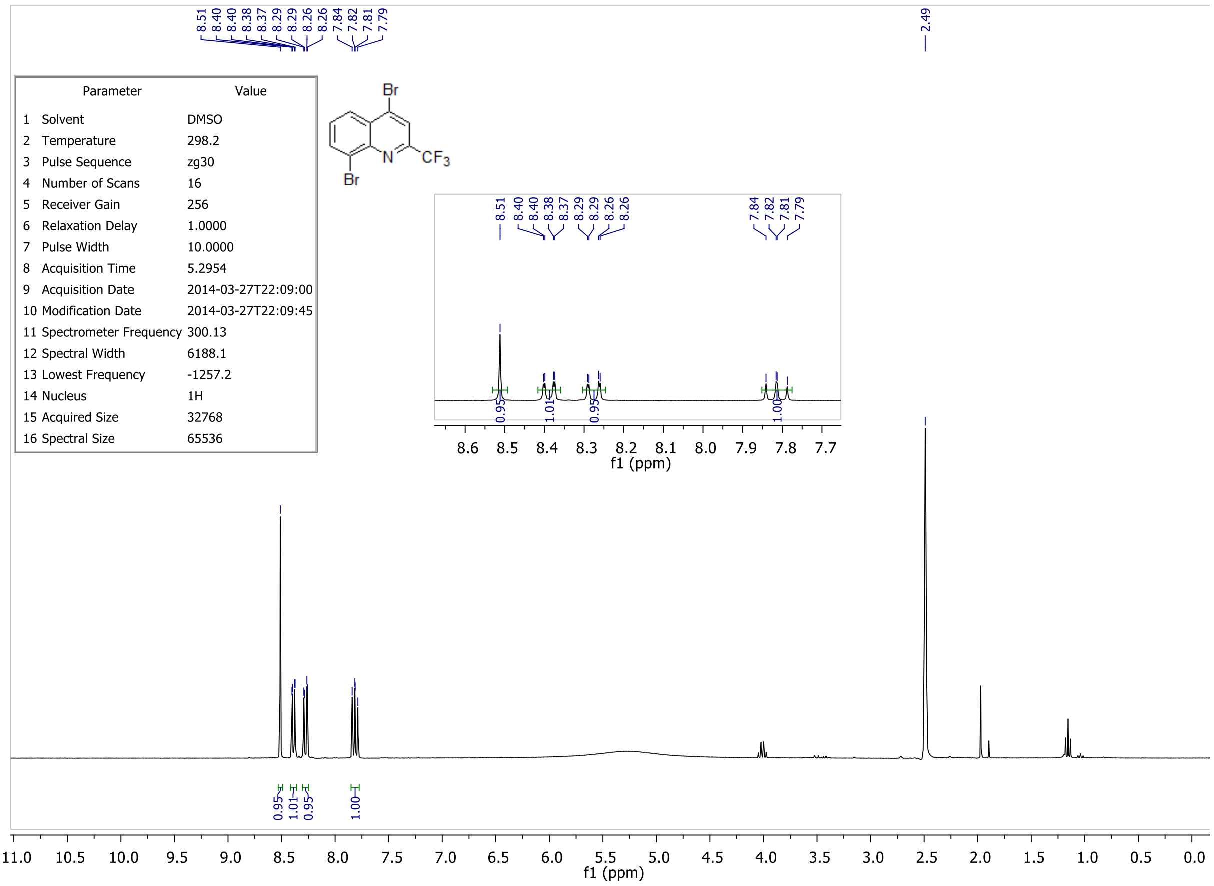

	Parameter	Value
1	Solvent	DMSO
2	Temperature	298.2
3	Pulse Sequence	zg30
4	Number of Scans	16
5	Receiver Gain	256
6	Relaxation Delay	1.0000
7	Pulse Width	10.0000
8	Acquisition Time	5.2954
9	Acquisition Date	2014-03-27T22:09:00
10	Modification Date	2014-03-27T22:09:45
11	Spectrometer Frequency	300.13
12	Spectral Width	6188.1
13	Lowest Frequency	-1257.2
14	Nucleus	1H
15	Acquired Size	32768
16	Spectral Size	65536

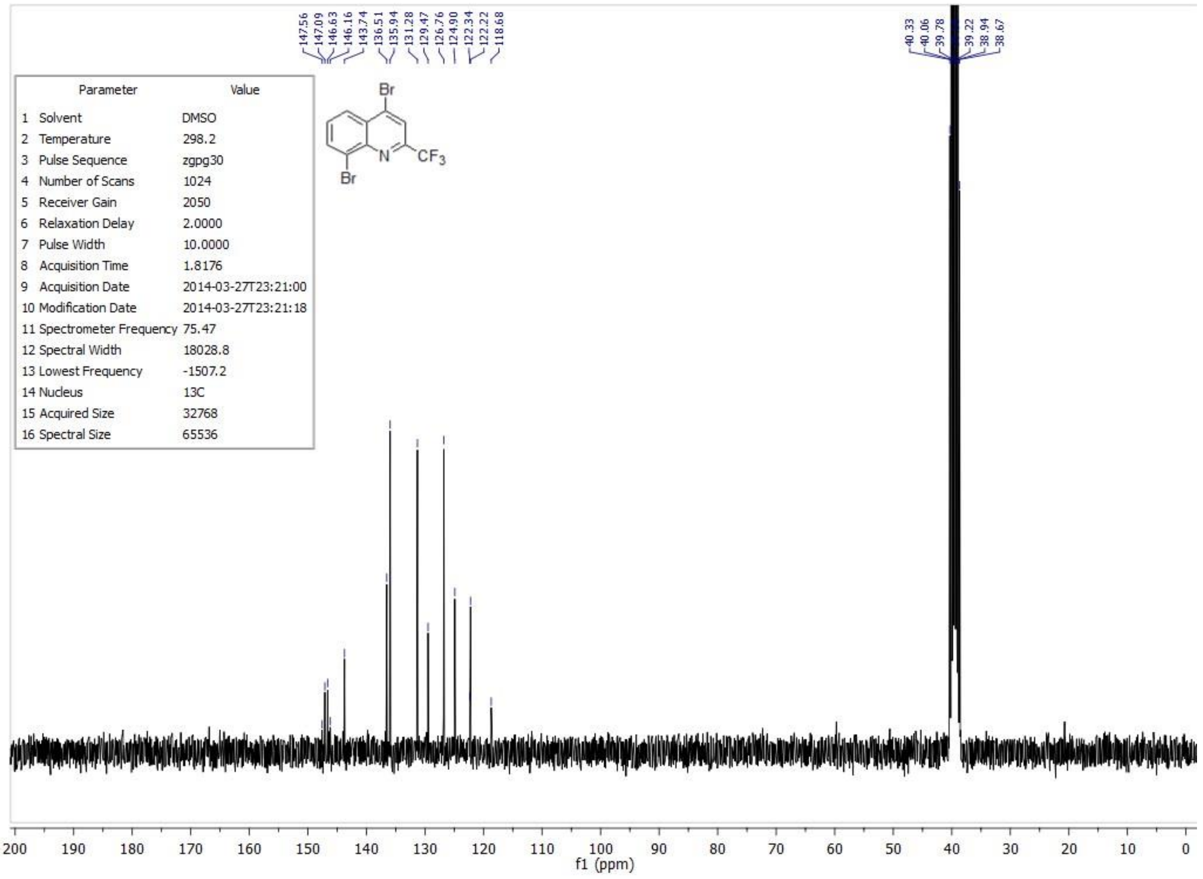
147.56
147.09
146.63
146.16
143.74
136.51
135.94
131.28
129.47
126.76
124.90
122.34
122.22
118.68
40.33
40.06
39.78
39.22
38.94
38.67
Parameter Value
1 Solvent DMSO
2 Temperature 298.2
3 Pulse Sequence zgpg30
4 Number of Scans 1024
5 Receiver Gain 2050
6 Relaxation Delay 2.0000
7 Pulse Width 10.0000
8 Acquisition Time 1.8176
9 Acquisition Date 2014-03-27T23:21:00
10 Modification Date 2014-03-27T23:21:18
11 Spectrometer Frequency 75.47
12 Spectral Width 18028.8
13 Lowest Frequency -1507.2
14 Nucleus 13C
15 Acquired Size 32768
16 Spectral Size 65536
Br
N
CF3
Br
f1 (ppm)

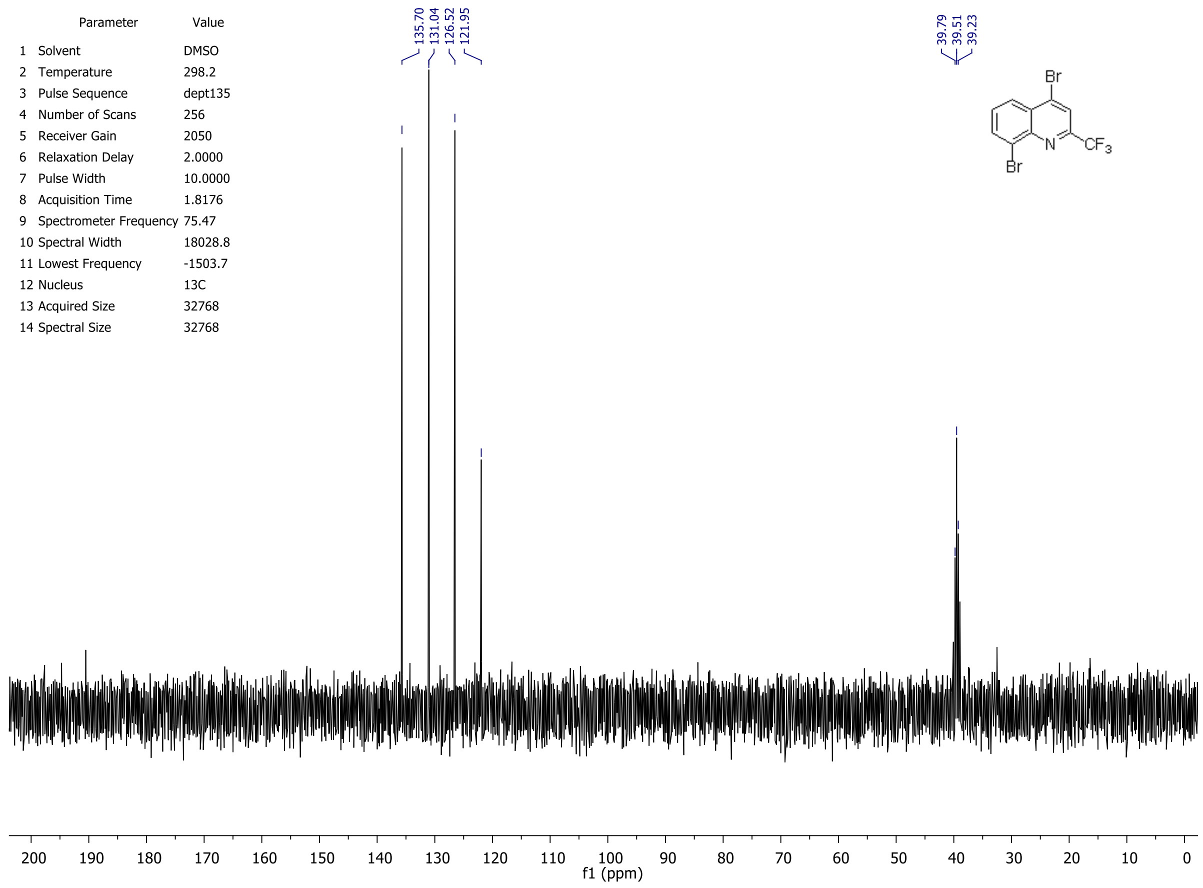
Parameter Value
1 Solvent DMSO
2 Temperature 298.2
3 Pulse Sequence dept135
4 Number of Scans 256
5 Receiver Gain 2050
6 Relaxation Delay 2.0000
7 Pulse Width 10.0000
8 Acquisition Time 1.8176
9 Spectrometer Frequency 75.47
10 Spectral Width 18028.8
11 Lowest Frequency -1503.7
12 Nucleus 13C
13 Acquired Size 32768
14 Spectral Size 32768
135.70
131.04
126.52
121.95
39.79
39.51
39.23
Br
N
CF3
Br
f1 (ppm)

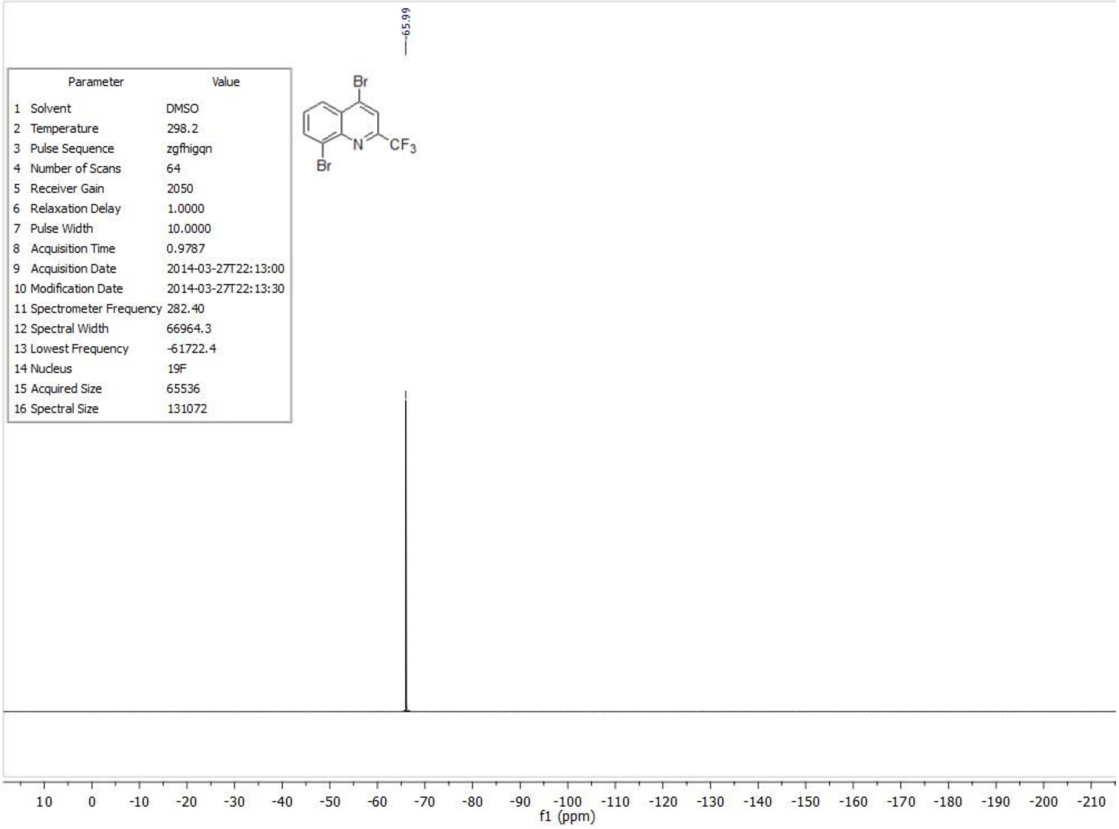

7 - 6-Bromo-2-trifluoromethyl-4-quinolinone

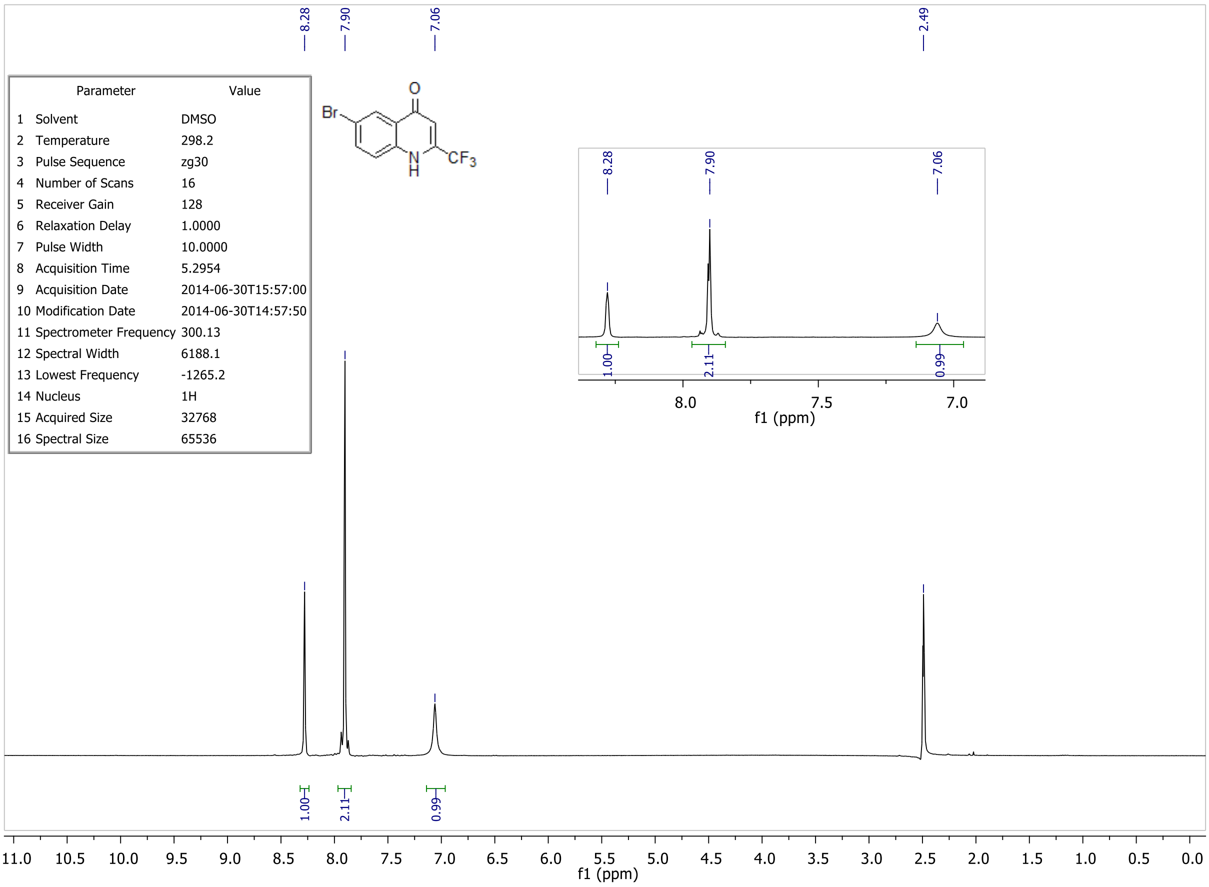

	Parameter	Value
1	Solvent	DMSO
2	Temperature	298.3
3	Pulse Sequence	zgpg30
4	Number of Scans	1024
5	Receiver Gain	2050
6	Relaxation Delay	2.0000
7	Pulse Width	10.0000
8	Acquisition Time	1.8176
9	Spectrometer Frequency	75.47
10	Spectral Width	18028.8
11	Lowest Frequency	-1505.9
12	Nucleus	13C
13	Acquired Size	32768
14	Spectral Size	32768

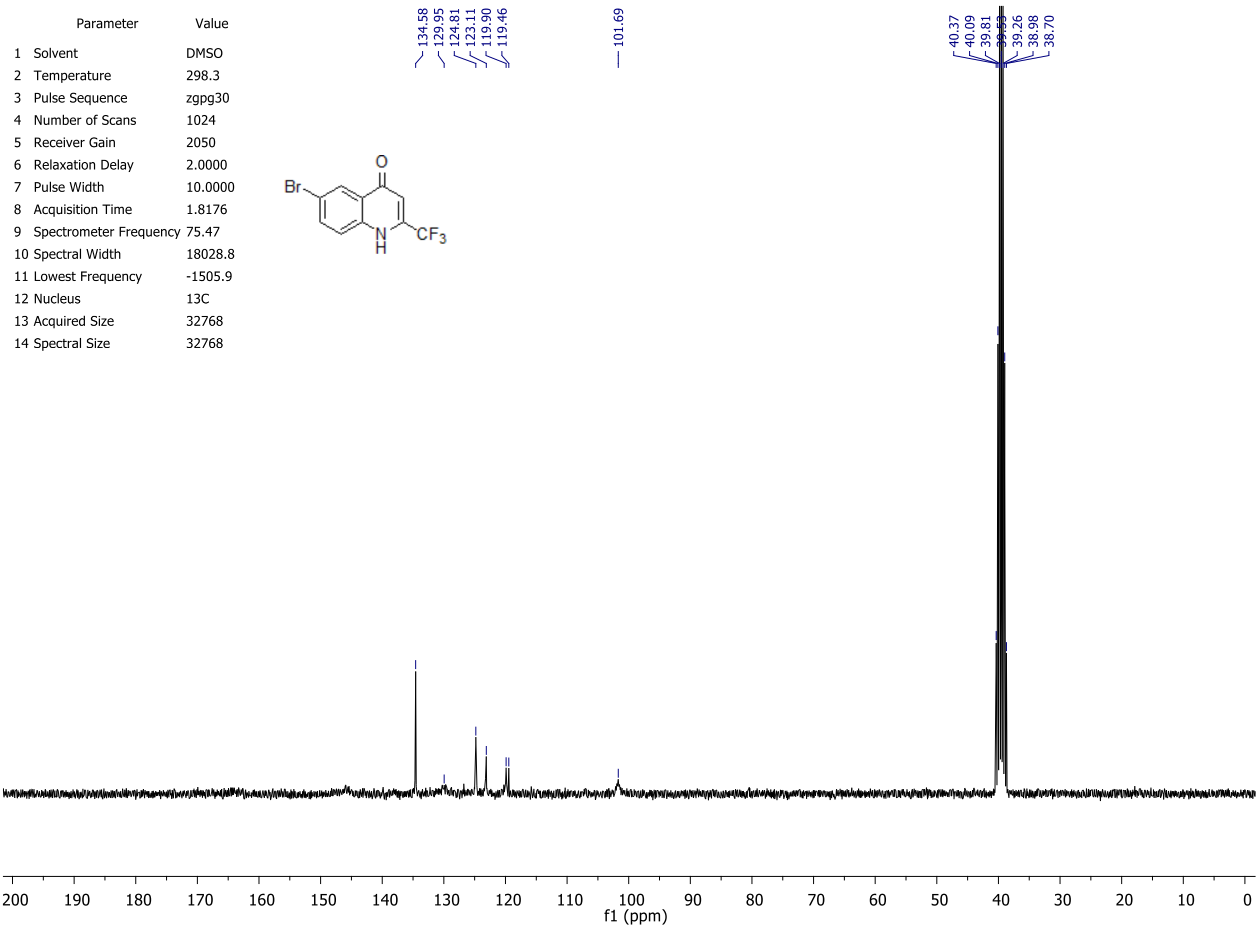

	Parameter	Value
1	Solvent	DMSO
2	Temperature	298.2
3	Pulse Sequence	dept135
4	Number of Scans	256
5	Receiver Gain	2050
6	Relaxation Delay	2.0000
7	Pulse Width	10.0000
8	Acquisition Time	1.8176
9	Spectrometer Frequency	75.47
10	Spectral Width	18028.8
11	Lowest Frequency	-1505.9
12	Nucleus	13C
13	Acquired Size	32768
14	Spectral Size	32768

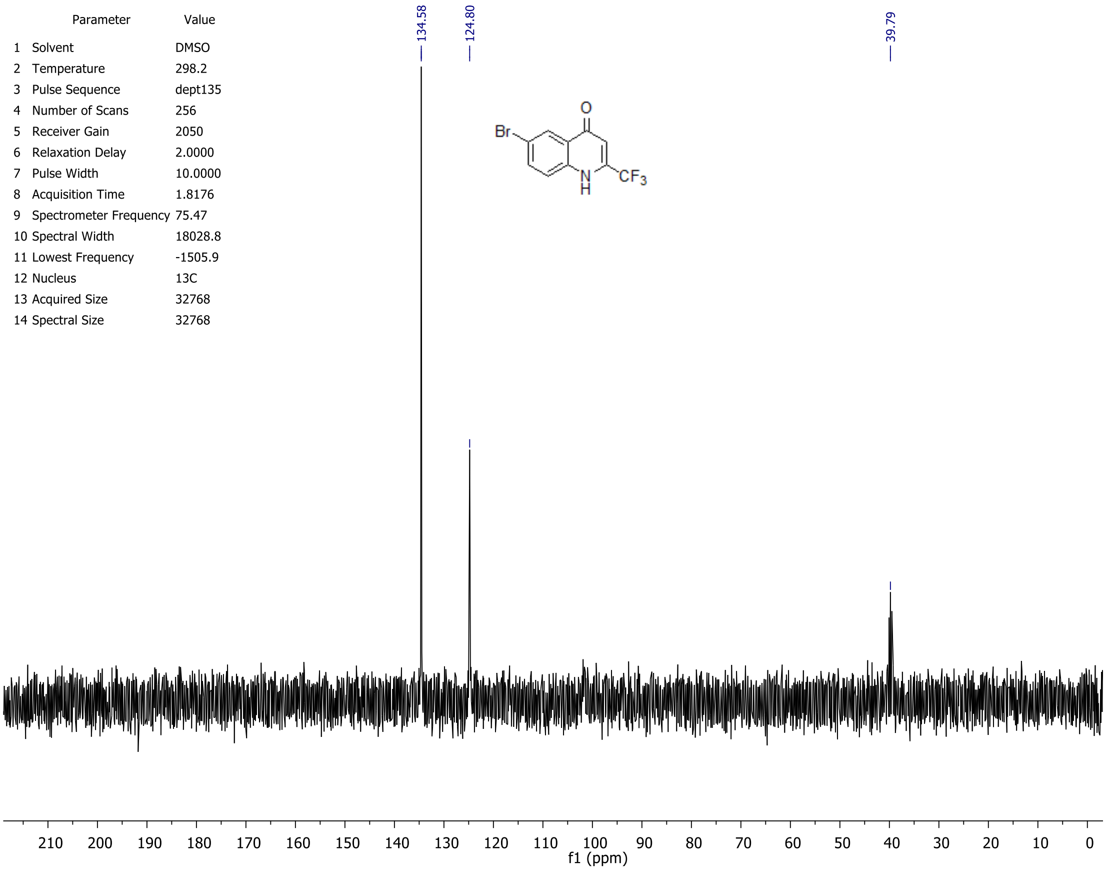

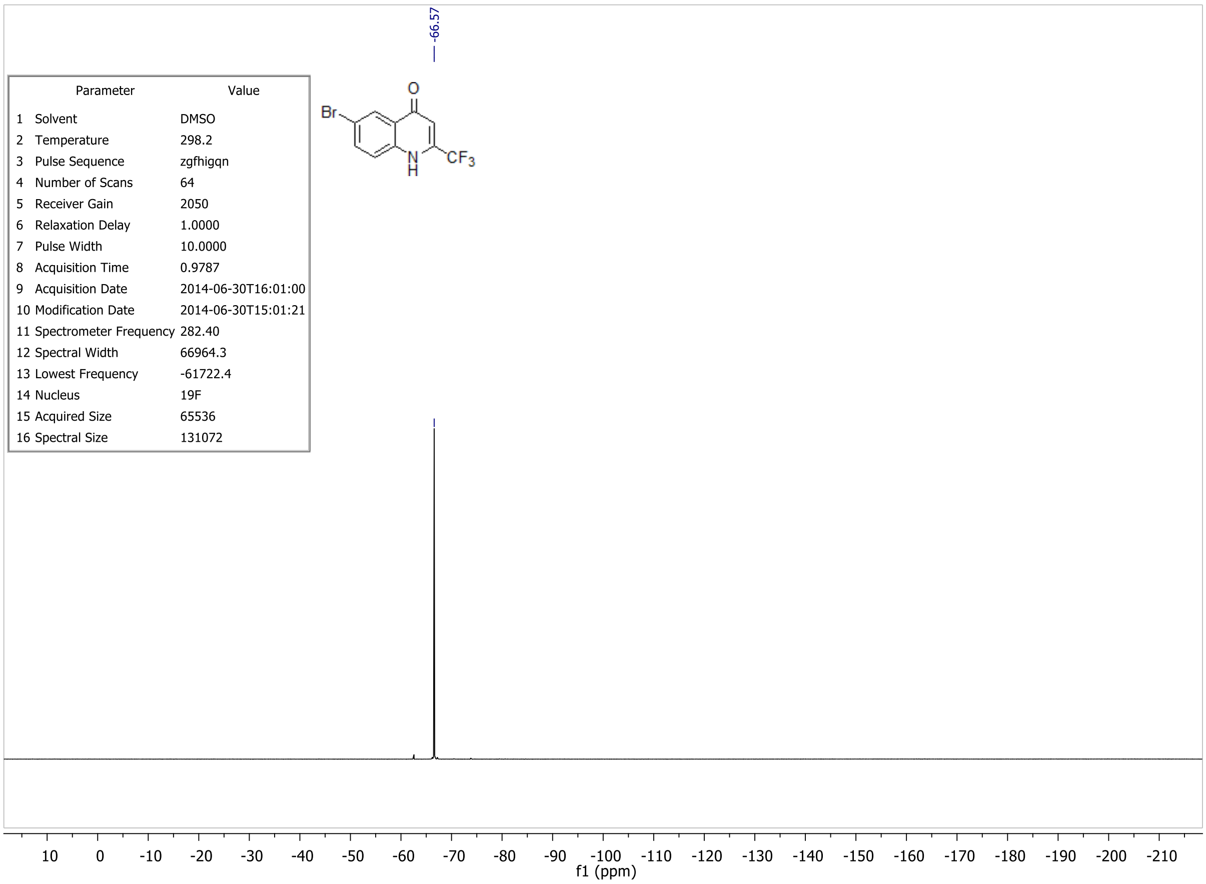
-66.57
Parameter Value
1 Solvent DMSO
2 Temperature 298.2
3 Pulse Sequence zgfhigqn
4 Number of Scans 64
5 Receiver Gain 2050
6 Relaxation Delay 1.0000
7 Pulse Width 10.0000
8 Acquisition Time 0.9787
9 Acquisition Date 2014-06-30T16:01:00
10 Modification Date 2014-06-30T15:01:21
11 Spectrometer Frequency 282.40
12 Spectral Width 66964.3
13 Lowest Frequency -61722.4
14 Nucleus 19F
15 Acquired Size 65536
16 Spectral Size 131072
Br
O
N
H
CF3
10 0 -10 -20 -30 -40 -50 -60 -70 -80 -90 -100 -110 -120 -130 -140 -150 -160 -170 -180 -190 -200 -210
f1 (ppm)

8 - 4,6-Dibromo-2-trifluoromethylquinoline

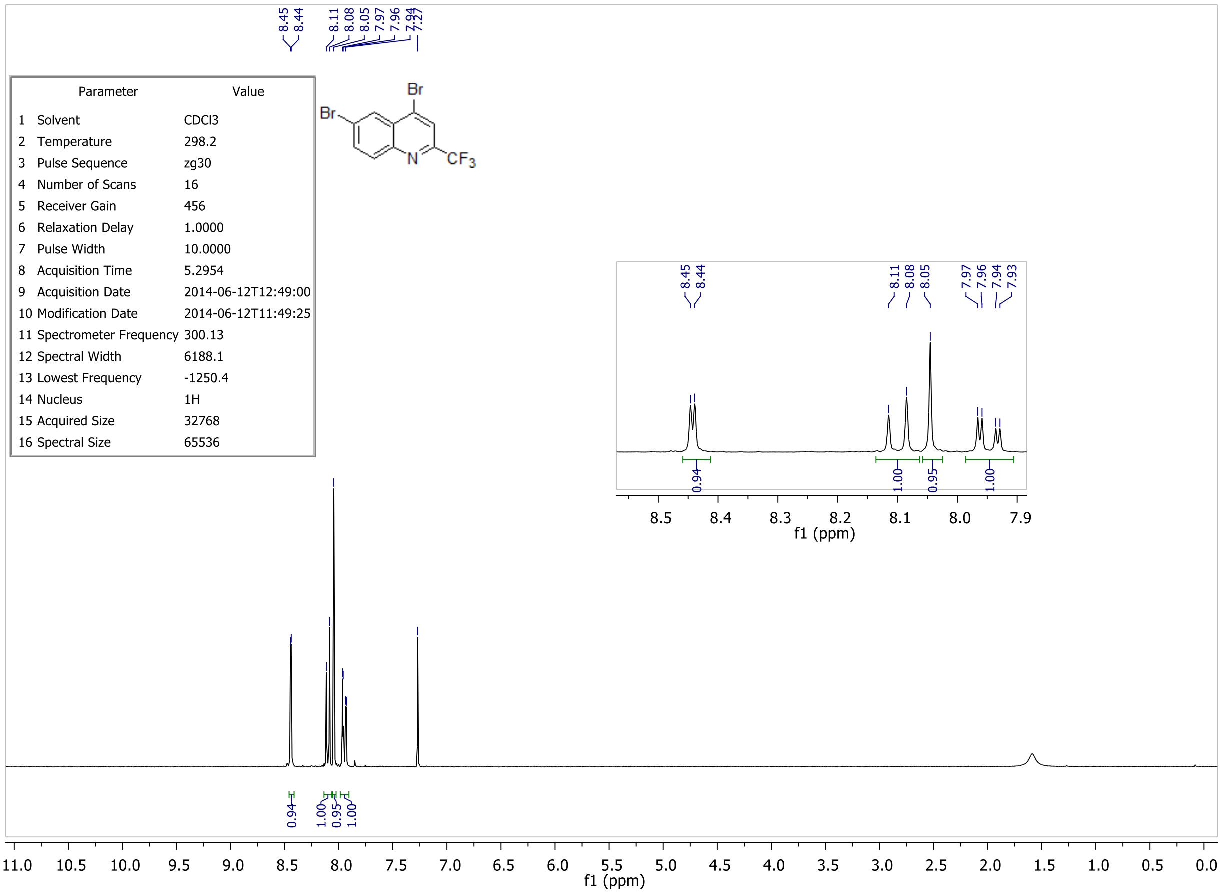

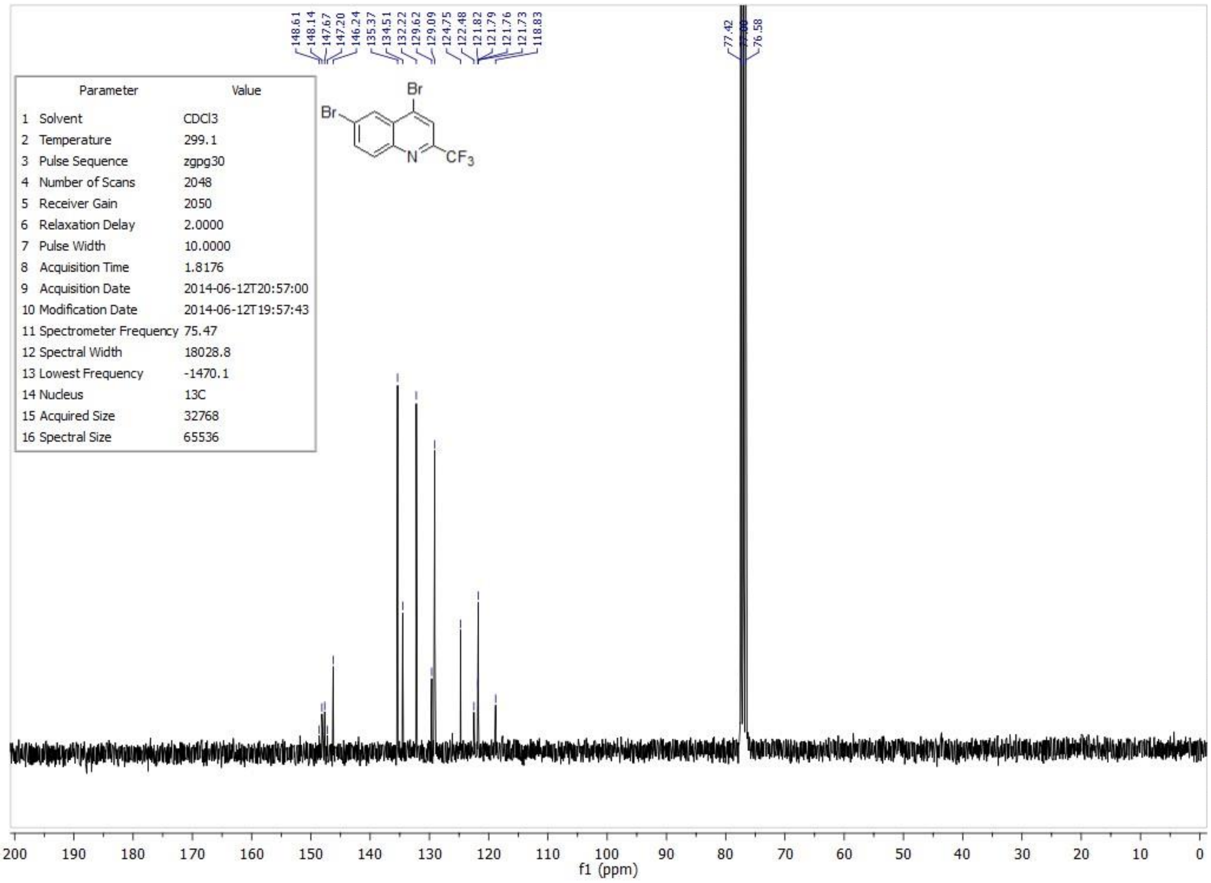
148.61
148.14
147.67
147.20
146.24
135.37
134.51
132.22
129.62
129.09
124.75
122.48
121.82
121.79
121.76
121.73
118.83
77.42
76.58
Parameter Value
1 Solvent CDCl3
2 Temperature 299.1
3 Pulse Sequence zgpg30
4 Number of Scans 2048
5 Receiver Gain 2050
6 Relaxation Delay 2.0000
7 Pulse Width 10.0000
8 Acquisition Time 1.8176
9 Acquisition Date 2014-06-12T20:57:00
10 Modification Date 2014-06-12T19:57:43
11 Spectrometer Frequency 75.47
12 Spectral Width 18028.8
13 Lowest Frequency -1470.1
14 Nucleus 13C
15 Acquired Size 32768
16 Spectral Size 65536
Br
Br
N
CF3
f1 (ppm)

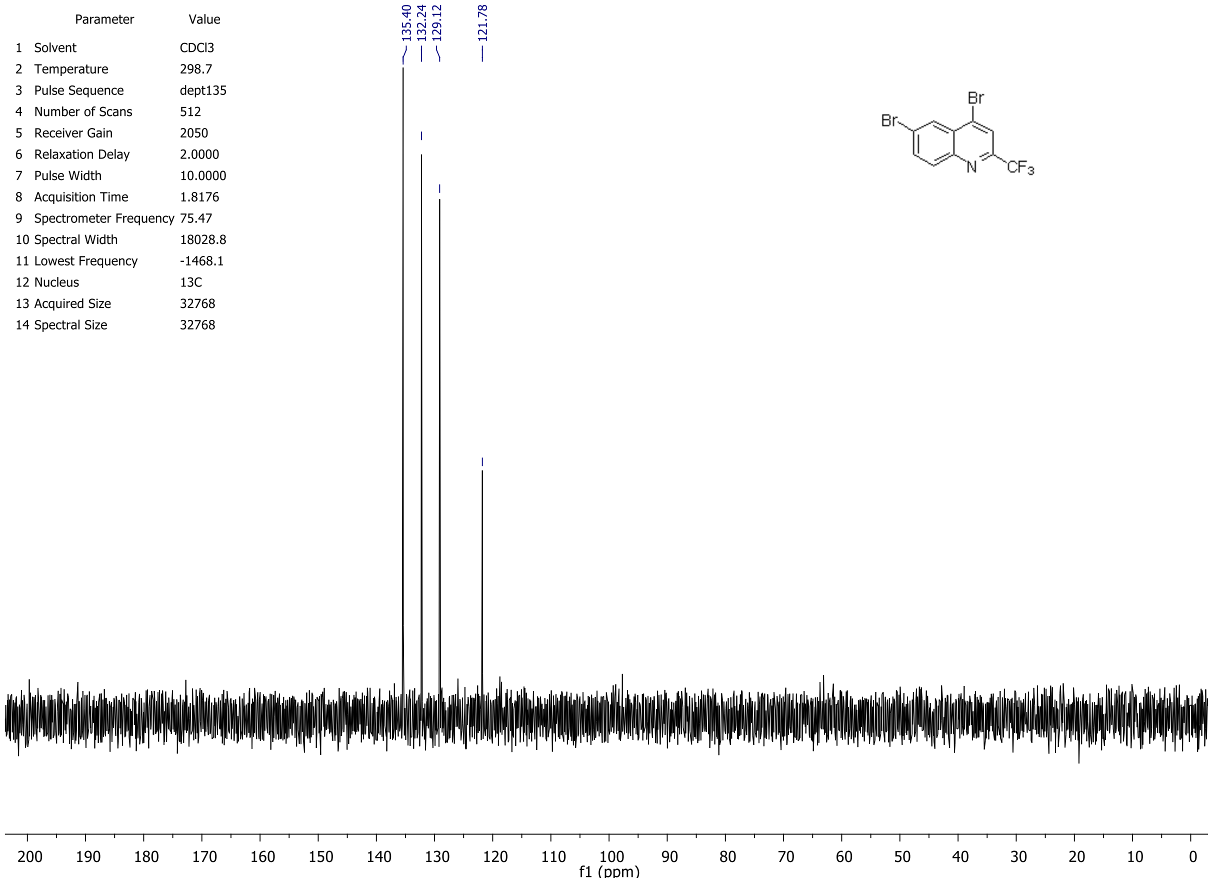
Parameter Value
1 Solvent CDCl3
2 Temperature 298.7
3 Pulse Sequence dept135
4 Number of Scans 512
5 Receiver Gain 2050
6 Relaxation Delay 2.0000
7 Pulse Width 10.0000
8 Acquisition Time 1.8176
9 Spectrometer Frequency 75.47
10 Spectral Width 18028.8
11 Lowest Frequency -1468.1
12 Nucleus 13C
13 Acquired Size 32768
14 Spectral Size 32768
135.40
132.24
129.12
121.78
Br
Br
N
CF3
f1 (ppm)

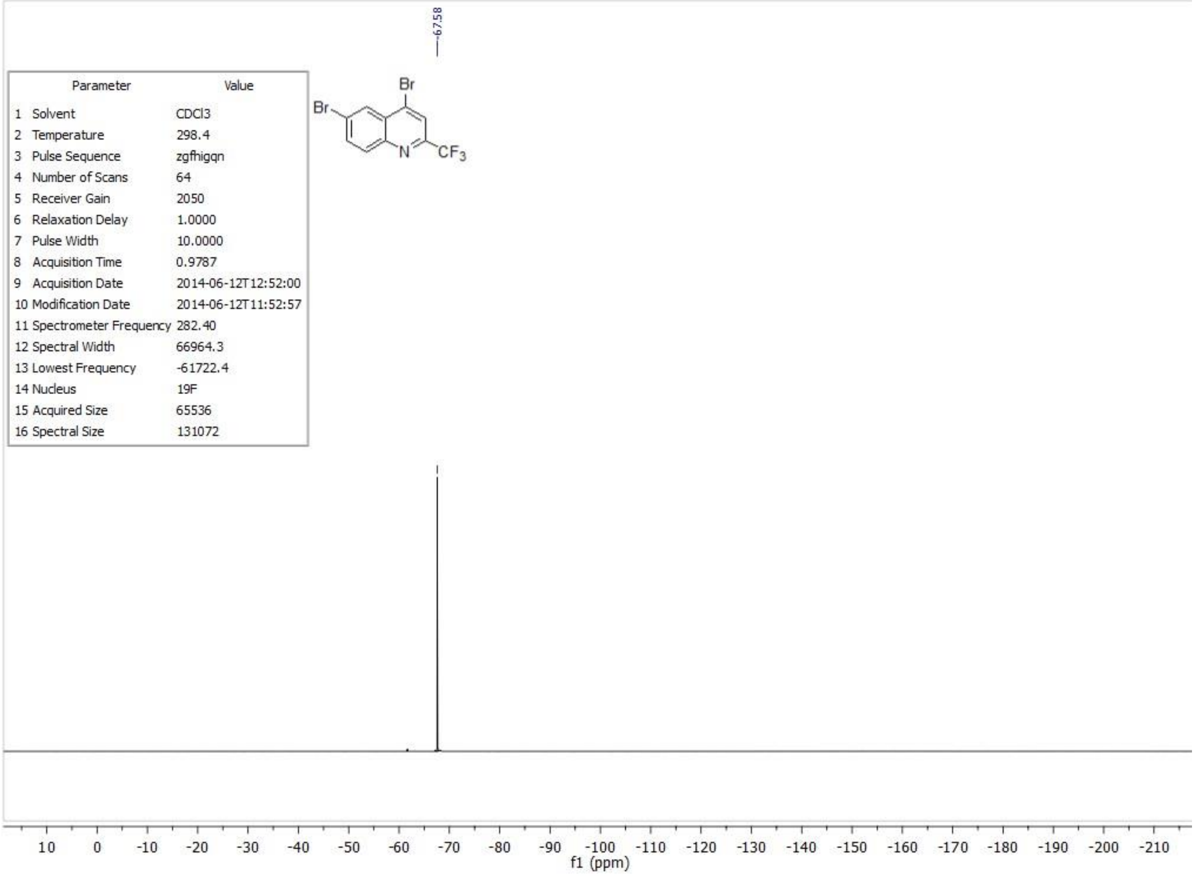
-67.58
Parameter Value
1 Solvent CDCl3
2 Temperature 298.4
3 Pulse Sequence zgfhigqn
4 Number of Scans 64
5 Receiver Gain 2050
6 Relaxation Delay 1.0000
7 Pulse Width 10.0000
8 Acquisition Time 0.9787
9 Acquisition Date 2014-06-12T12:52:00
10 Modification Date 2014-06-12T11:52:57
11 Spectrometer Frequency 282.40
12 Spectral Width 66964.3
13 Lowest Frequency -61722.4
14 Nucleus 19F
15 Acquired Size 65536
16 Spectral Size 131072
Br
Br
N
CF3
10 0 -10 -20 -30 -40 -50 -60 -70 -80 -90 -100 -110 -120 -130 -140 -150 -160 -170 -180 -190 -200 -210
f1 (ppm)

10 – 3,8-Dibromo-2-trifluoromethyl-4-quinolinone

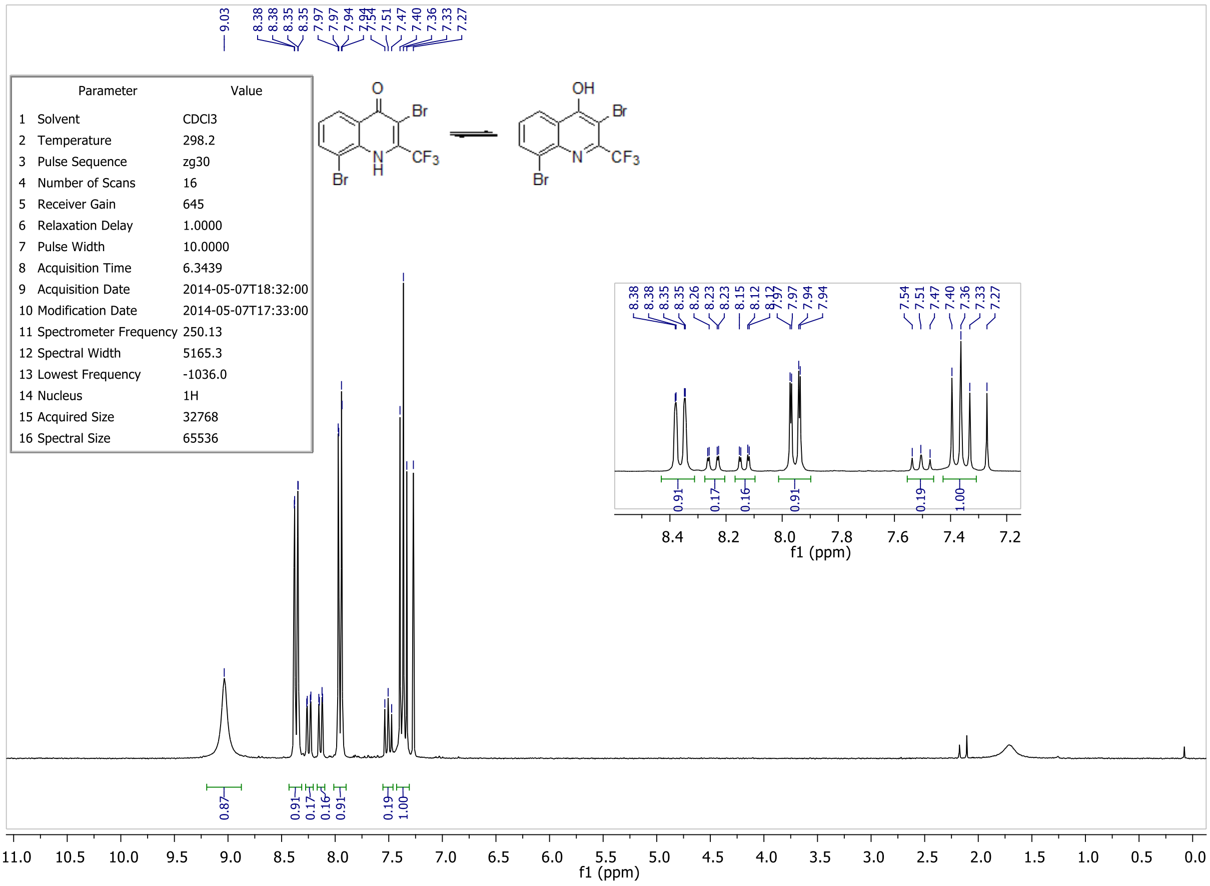

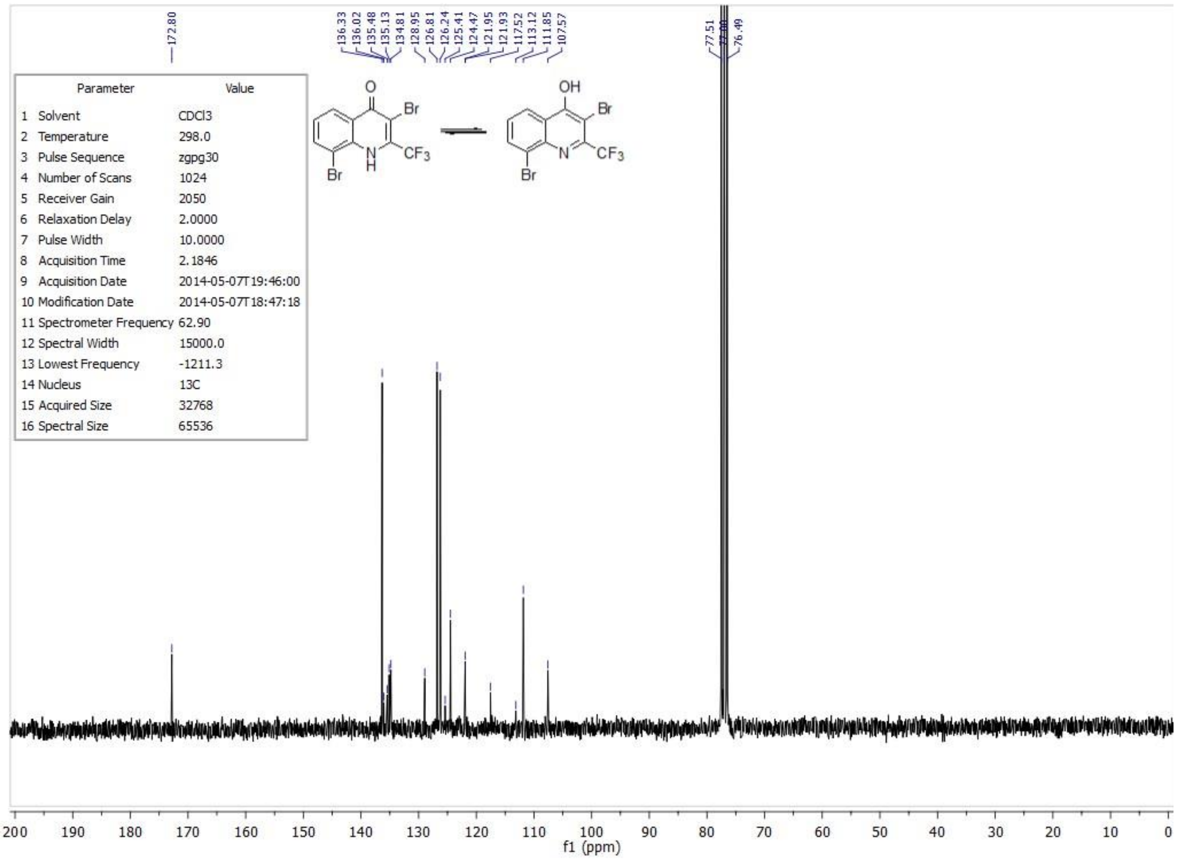

	Parameter	Value
1	Solvent	CDCl3
2	Temperature	298.0
3	Pulse Sequence	dept135
4	Number of Scans	256
5	Receiver Gain	1290
6	Relaxation Delay	2.0000
7	Pulse Width	10.0000
8	Acquisition Time	2.1846
9	Spectrometer Frequency	62.90
10	Spectral Width	15000.0
11	Lowest Frequency	-1210.9
12	Nucleus	13C
13	Acquired Size	32768
14	Spectral Size	32768

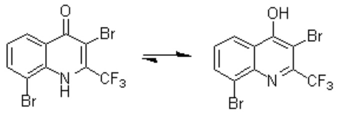

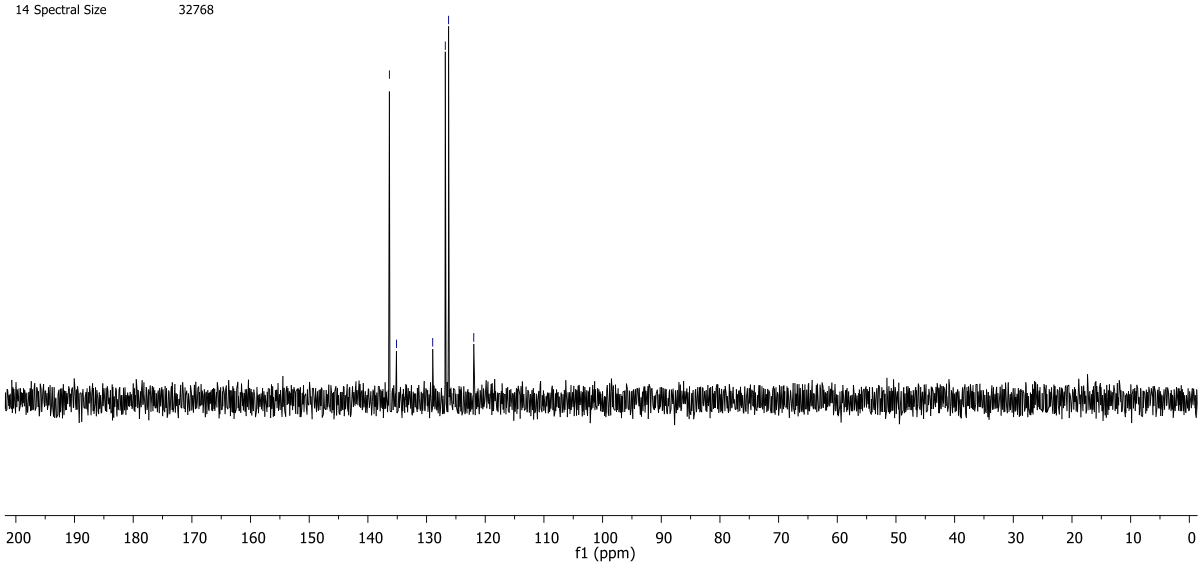

	Parameter	Value
1	Solvent	CDCl3
2	Temperature	298.2
3	Pulse Sequence	zgfhigqn
4	Number of Scans	64
5	Receiver Gain	2050
6	Relaxation Delay	1.0000
7	Pulse Width	10.0000
8	Acquisition Time	0.9787
9	Acquisition Date	2014-05-08T10:17:00
10	Modification Date	2014-05-08T09:17:53
11	Spectrometer Frequency	282.40
12	Spectral Width	66964.3
13	Lowest Frequency	-61722.4
14	Nucleus	19F
15	Acquired Size	65536
16	Spectral Size	131072

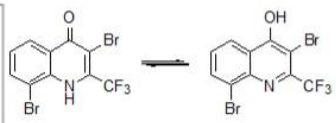

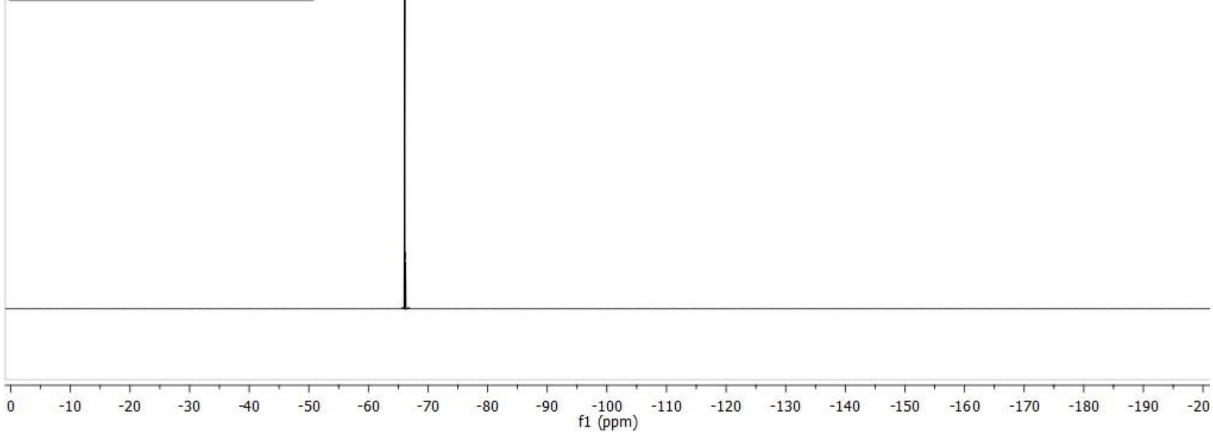

11 - 3,4,8-Tribromo-2-trifluoromethylquinoline

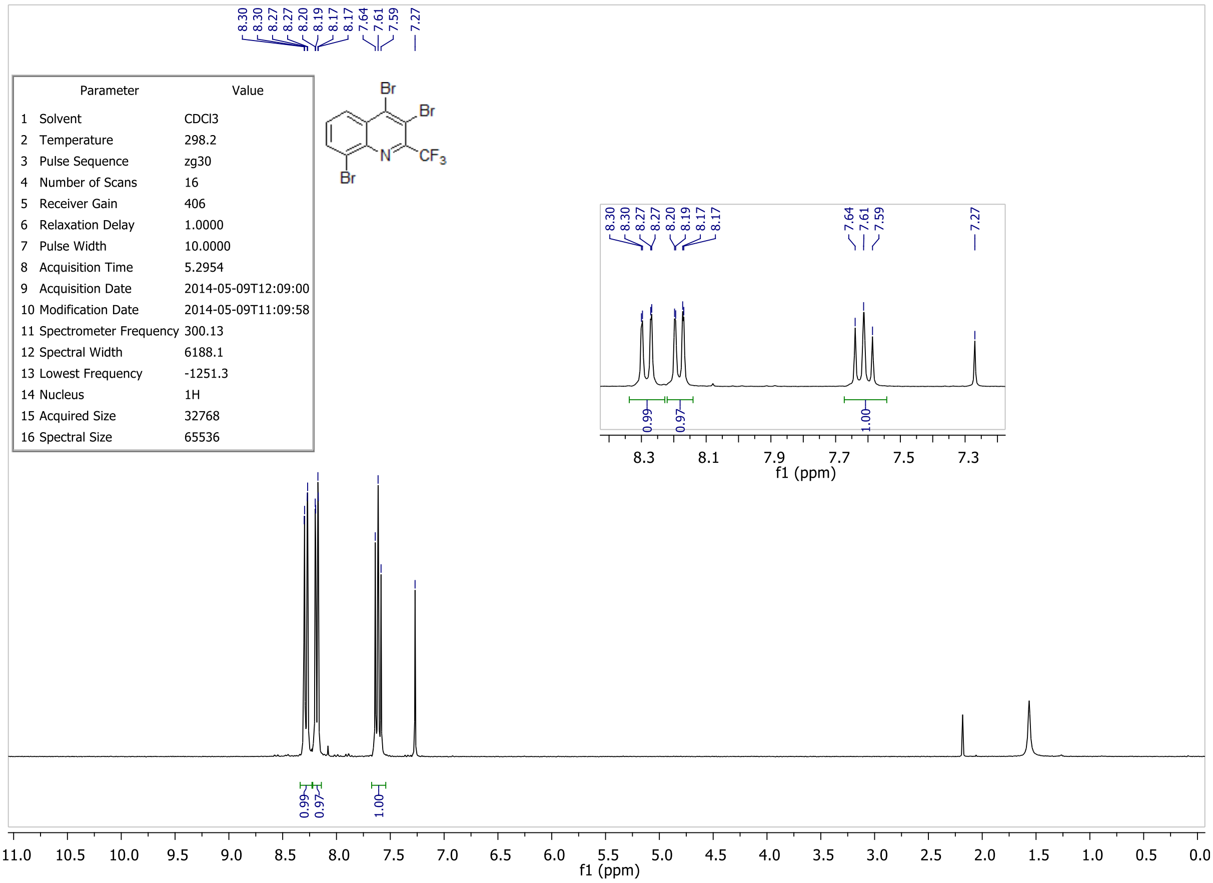

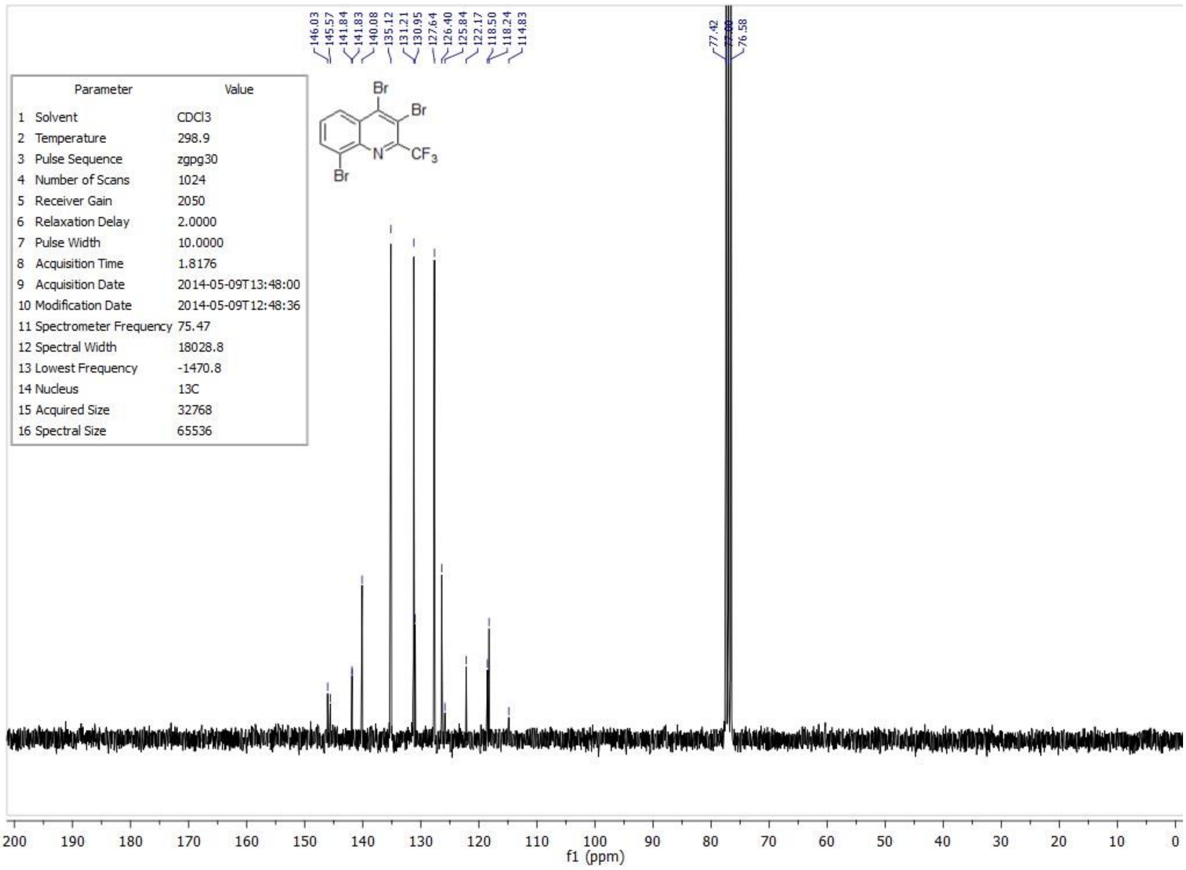

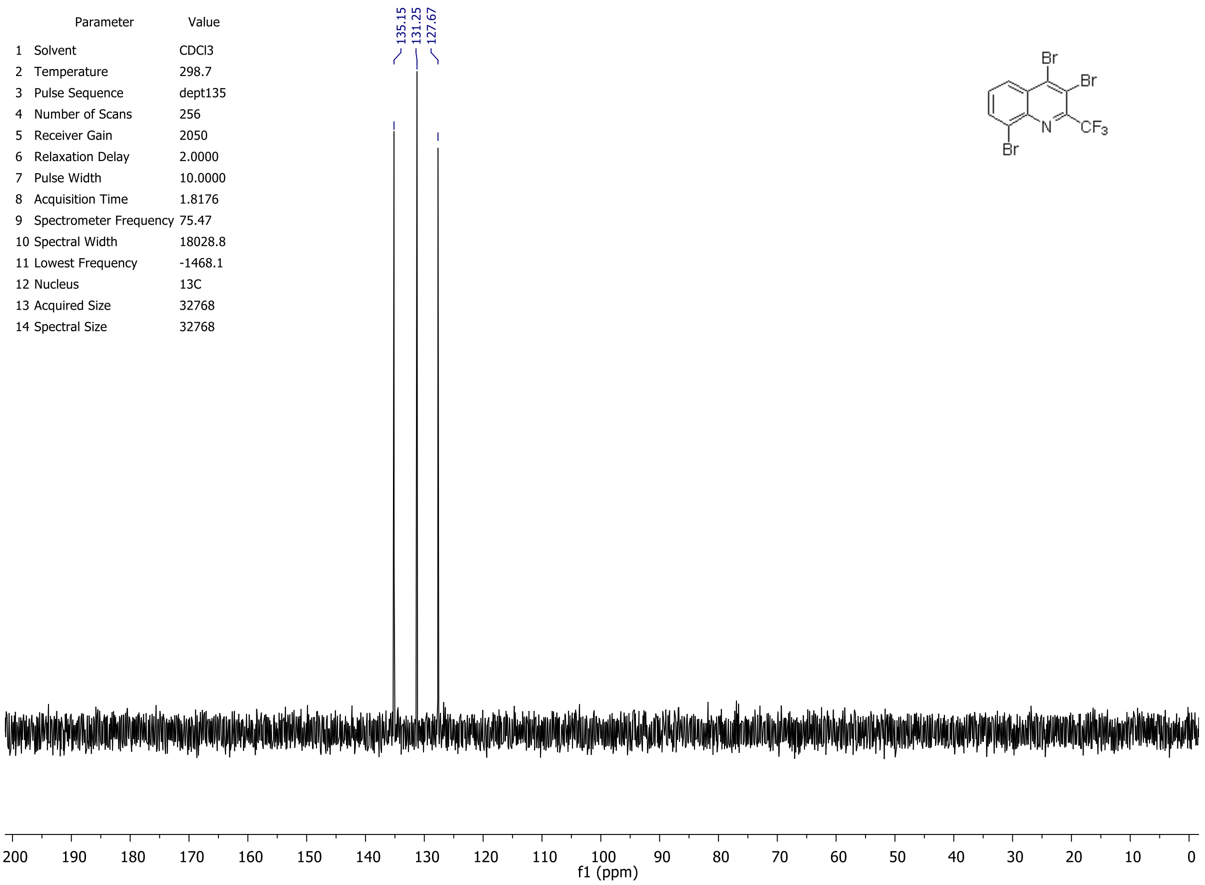
Parameter Value
1 Solvent CDCl3
2 Temperature 298.7
3 Pulse Sequence dept135
4 Number of Scans 256
5 Receiver Gain 2050
6 Relaxation Delay 2.0000
7 Pulse Width 10.0000
8 Acquisition Time 1.8176
9 Spectrometer Frequency 75.47
10 Spectral Width 18028.8
11 Lowest Frequency -1468.1
12 Nucleus 13C
13 Acquired Size 32768
14 Spectral Size 32768
135.15
131.25
127.67
Br
Br
N
CF3
Br
200 190 180 170 160 150 140 130 120 110 100 90 80 70 60 50 40 30 20 10 0
f1 (ppm)

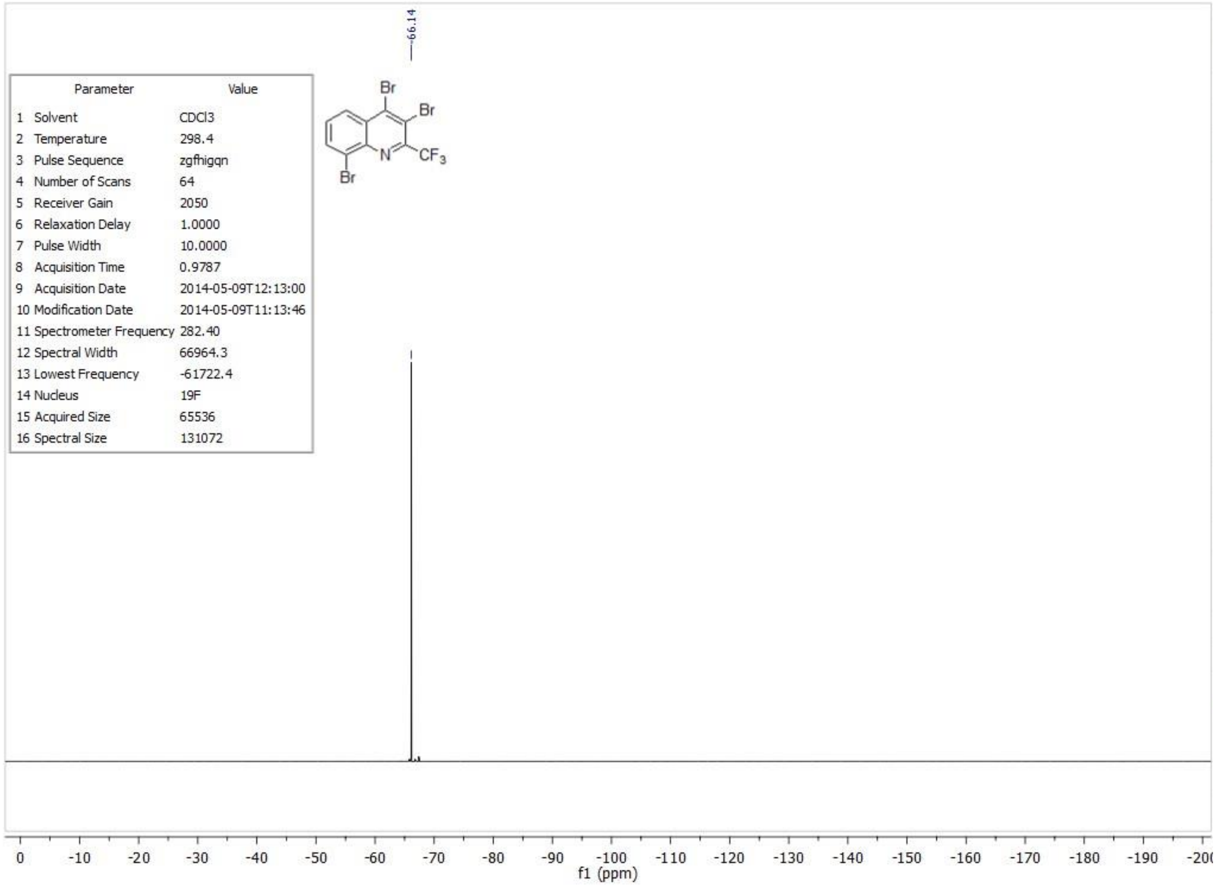
-66.14
Parameter Value
1 Solvent CDCl3
2 Temperature 298.4
3 Pulse Sequence zgfhigqn
4 Number of Scans 64
5 Receiver Gain 2050
6 Relaxation Delay 1.0000
7 Pulse Width 10.0000
8 Acquisition Time 0.9787
9 Acquisition Date 2014-05-09T12:13:00
10 Modification Date 2014-05-09T11:13:46
11 Spectrometer Frequency 282.40
12 Spectral Width 66964.3
13 Lowest Frequency -61722.4
14 Nucleus 19F
15 Acquired Size 65536
16 Spectral Size 131072
Br
Br
N
CF3
Br
0 -10 -20 -30 -40 -50 -60 -70 -80 -90 -100 -110 -120 -130 -140 -150 -160 -170 -180 -190 -200
f1 (ppm)

6a - 4,8-Bis(phenylethynyl)-2-trifluoromethylquinoline

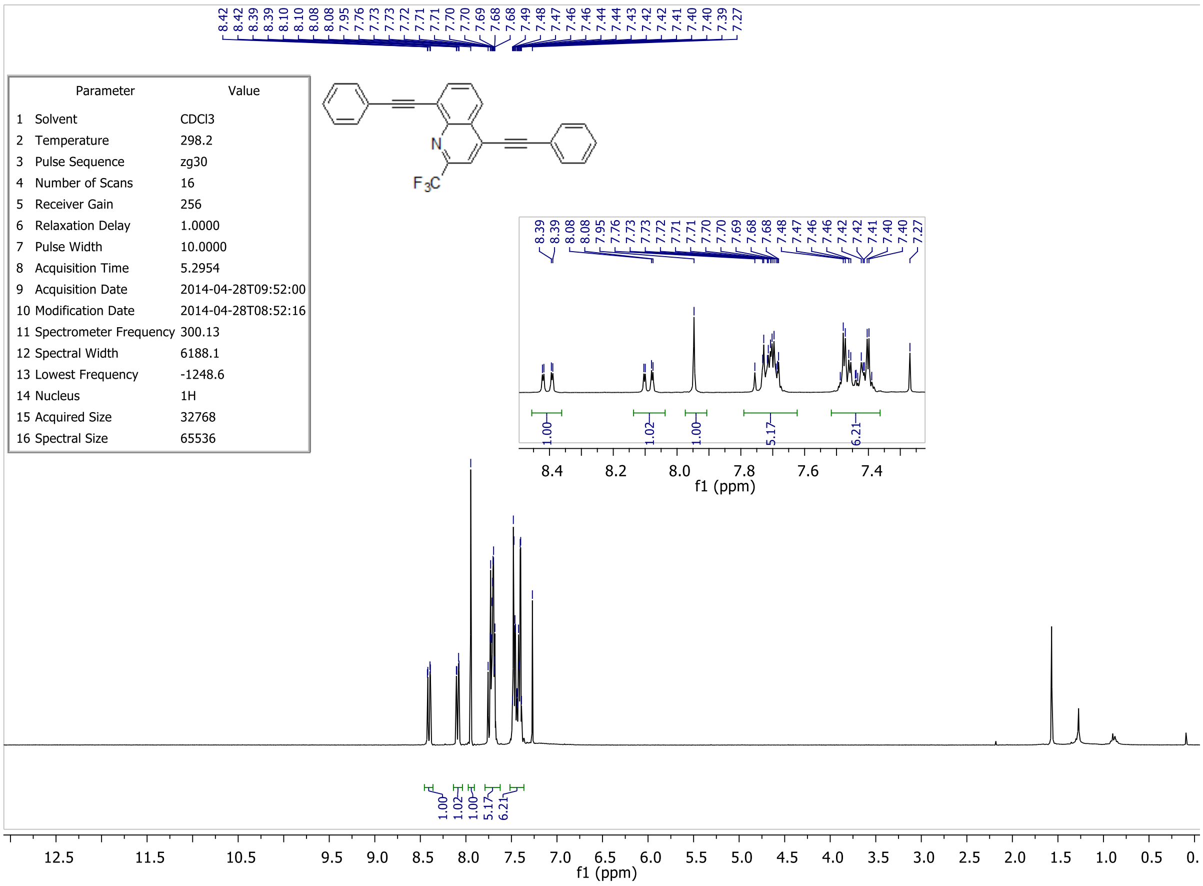

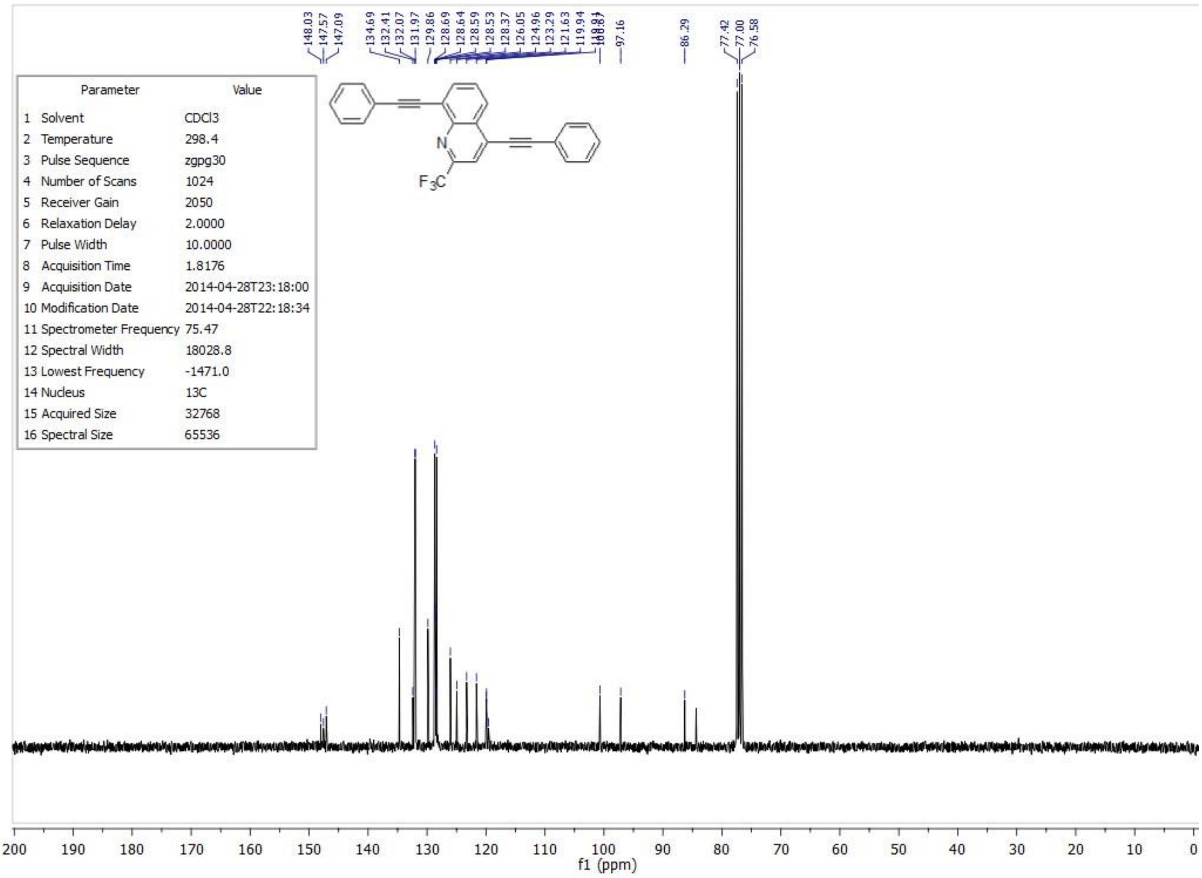

	Parameter	Value
1	Solvent	CDCl3
2	Temperature	298.4
3	Pulse Sequence	zgpg30
4	Number of Scans	1024
5	Receiver Gain	2050
6	Relaxation Delay	2.0000
7	Pulse Width	10.0000
8	Acquisition Time	1.8176
9	Acquisition Date	2014-04-28T23:18:00
10	Modification Date	2014-04-28T22:18:34
11	Spectrometer Frequency	75.47
12	Spectral Width	18028.8
13	Lowest Frequency	-1471.0
14	Nucleus	13C
15	Acquired Size	32768
16	Spectral Size	65536

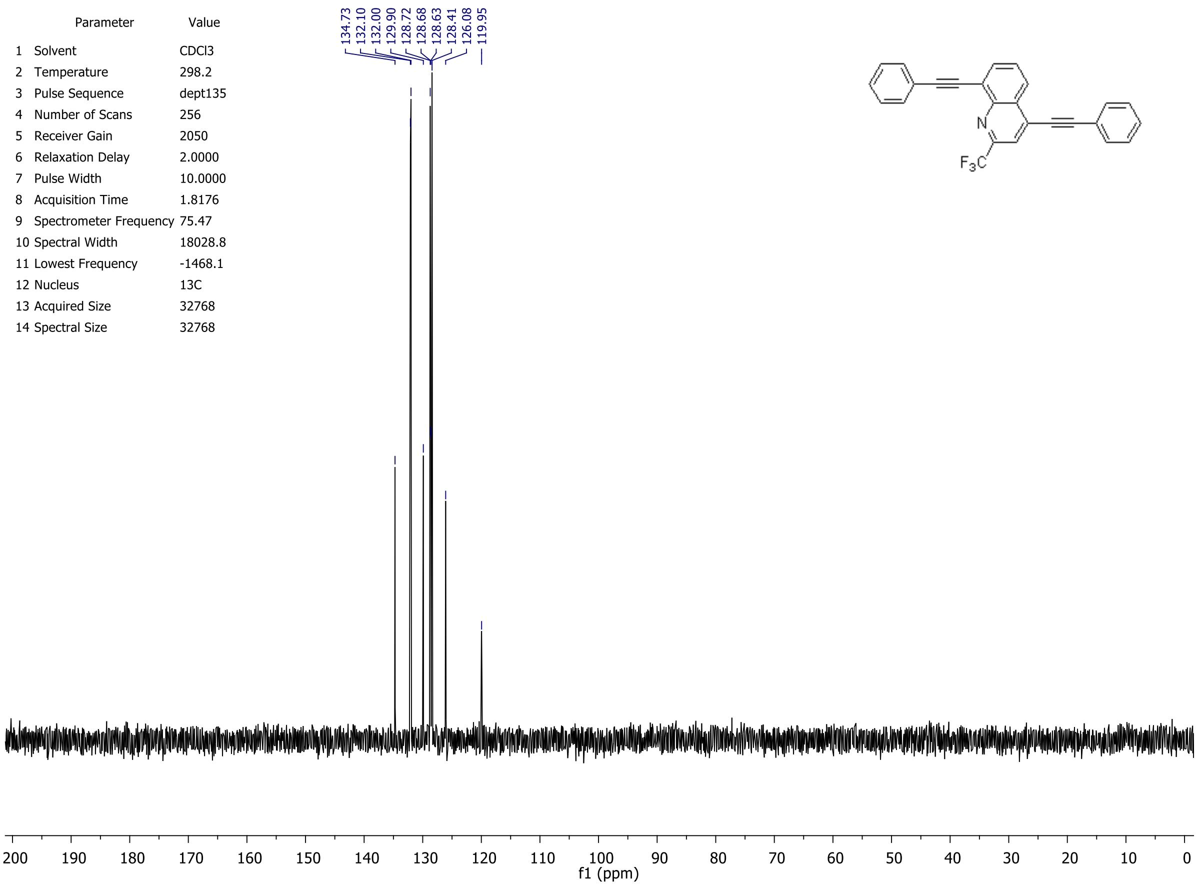

	Parameter	Value
1	Solvent	CDCl3
2	Temperature	298.2
3	Pulse Sequence	dept135
4	Number of Scans	256
5	Receiver Gain	2050
6	Relaxation Delay	2.0000
7	Pulse Width	10.0000
8	Acquisition Time	1.8176
9	Spectrometer Frequency	75.47
10	Spectral Width	18028.8
11	Lowest Frequency	-1468.1
12	Nucleus	13C
13	Acquired Size	32768
14	Spectral Size	32768

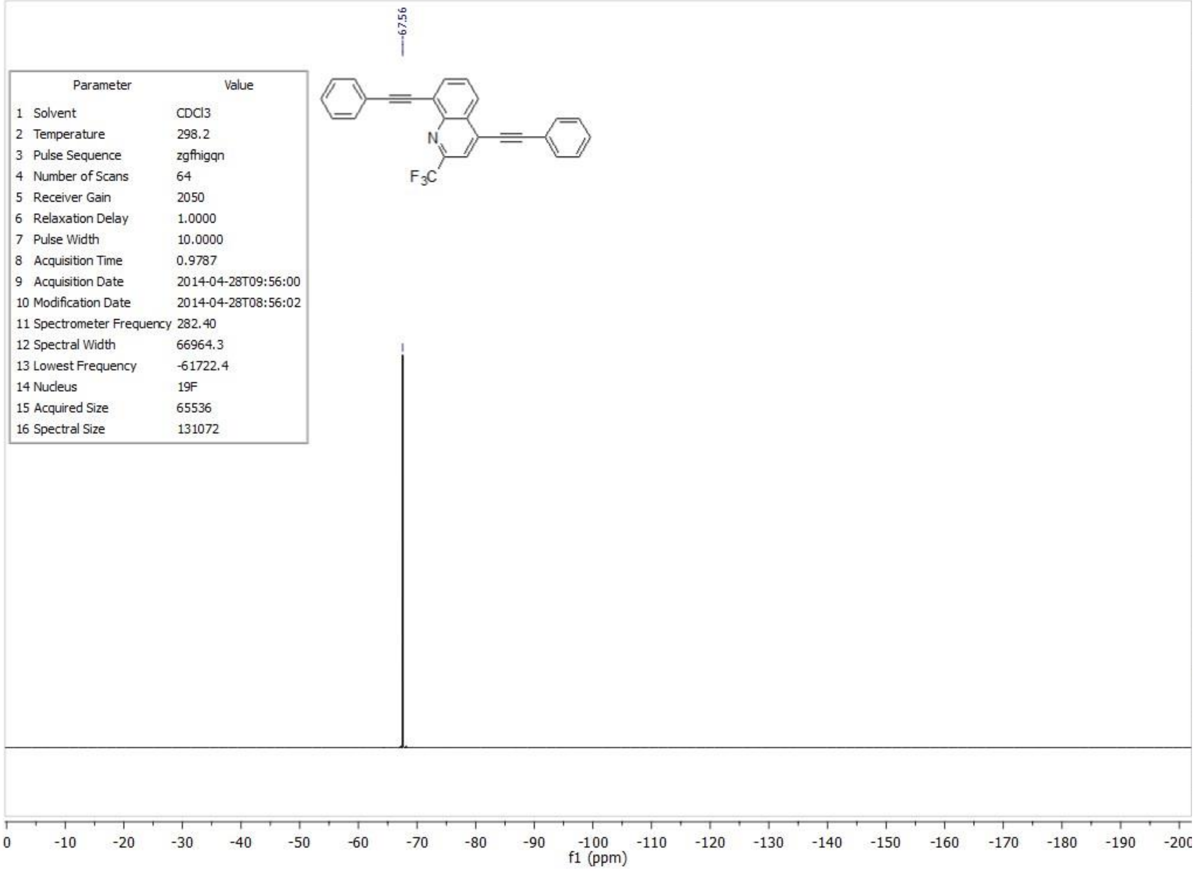

-67.56
Parameter Value
1 Solvent CDCl3
2 Temperature 298.2
3 Pulse Sequence zgfhigqn
4 Number of Scans 64
5 Receiver Gain 2050
6 Relaxation Delay 1.0000
7 Pulse Width 10.0000
8 Acquisition Time 0.9787
9 Acquisition Date 2014-04-28T09:56:00
10 Modification Date 2014-04-28T08:56:02
11 Spectrometer Frequency 282.40
12 Spectral Width 66964.3
13 Lowest Frequency -61722.4
14 Nucleus 19F
15 Acquired Size 65536
16 Spectral Size 131072
N
F3C
0 -10 -20 -30 -40 -50 -60 -70 -80 -90 -100 -110 -120 -130 -140 -150 -160 -170 -180 -190 -200
f1 (ppm)

6b - 4,8-Bis(4‘-tert-butylphenylethynyl)-2-trifluoromethylquinoline

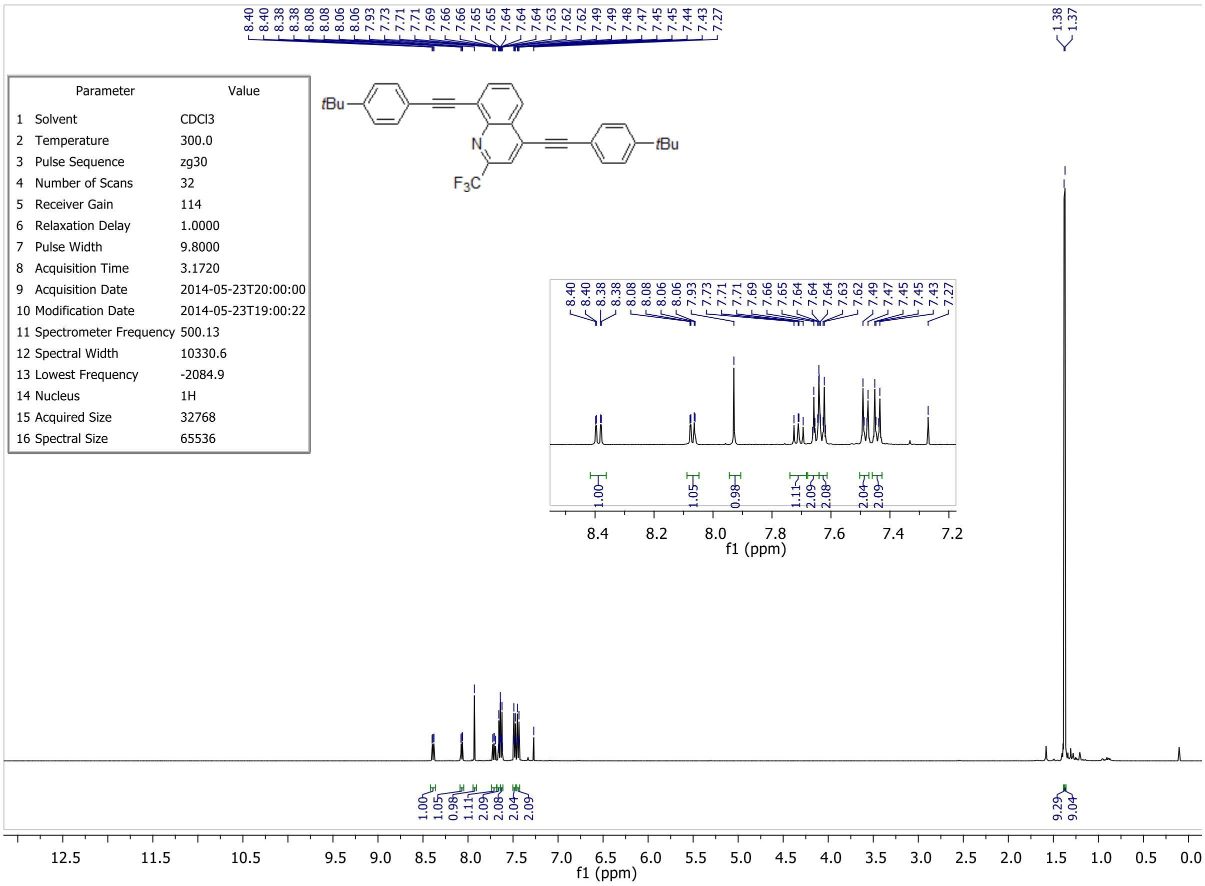

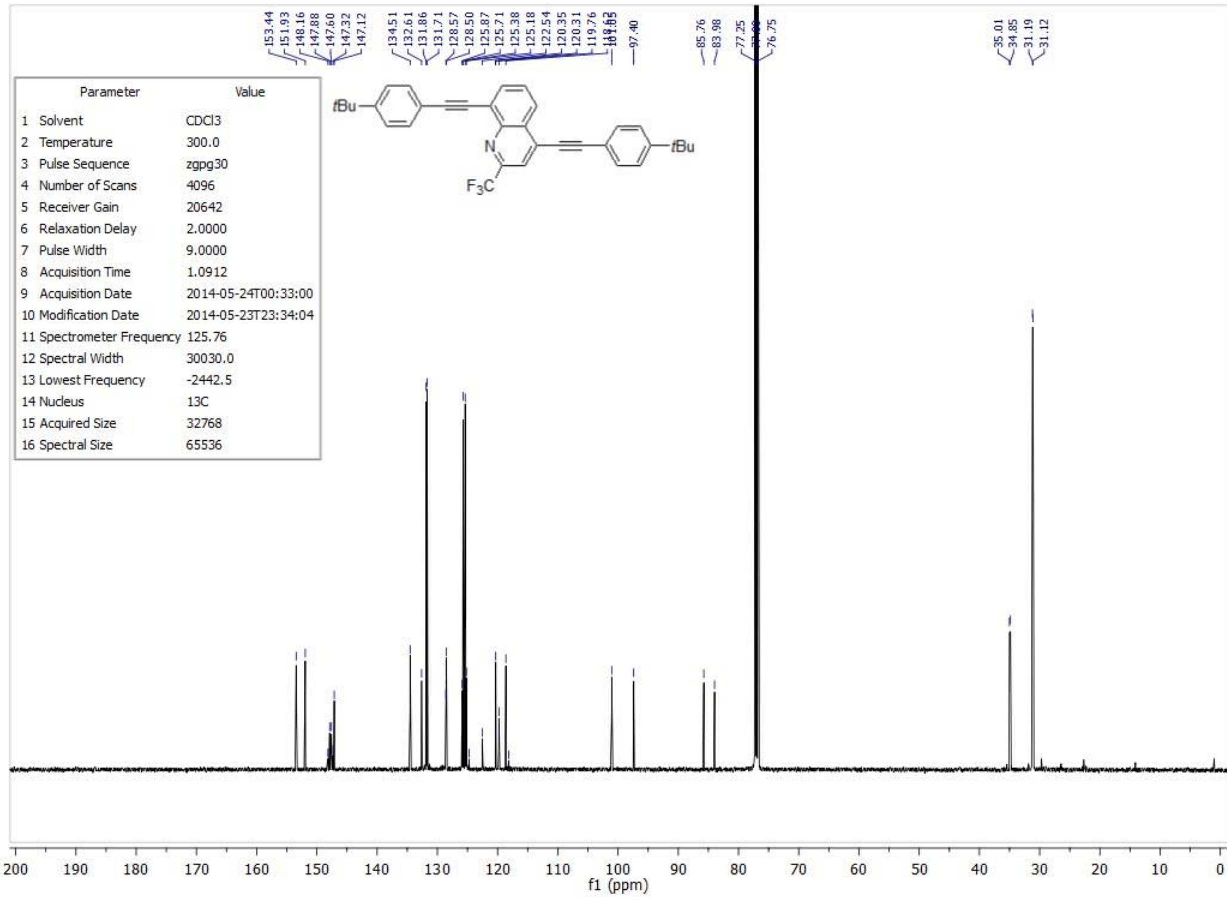
153.44
151.93
148.16
147.88
147.60
147.32
147.12
134.51
132.61
131.86
131.71
128.57
128.50
125.87
125.71
125.38
125.18
122.54
120.35
120.31
119.76
97.40
85.76
83.98
77.25
76.75
35.01
34.85
31.19
31.12
Parameter Value
1 Solvent CDCl3
2 Temperature 300.0
3 Pulse Sequence zgpg30
4 Number of Scans 4096
5 Receiver Gain 20642
6 Relaxation Delay 2.0000
7 Pulse Width 9.0000
8 Acquisition Time 1.0912
9 Acquisition Date 2014-05-24T00:33:00
10 Modification Date 2014-05-23T23:34:04
11 Spectrometer Frequency 125.76
12 Spectral Width 30030.0
13 Lowest Frequency -2442.5
14 Nucleus 13C
15 Acquired Size 32768
16 Spectral Size 65536
tBu
N
tBu
F3C
200 190 180 170 160 150 140 130 120 110 100 90 80 70 60 50 40 30 20 10 0
f1 (ppm)

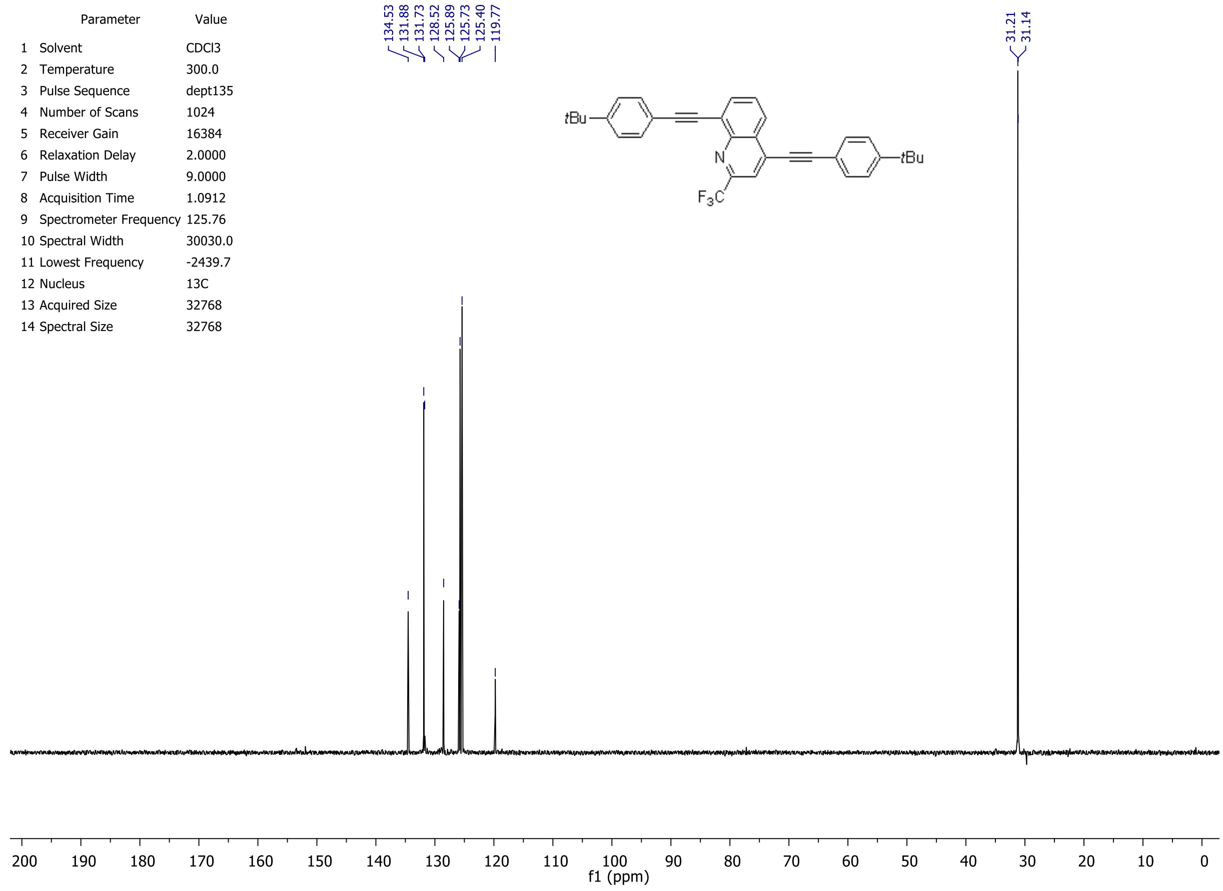
Parameter Value
1 Solvent CDCl3
2 Temperature 300.0
3 Pulse Sequence dept135
4 Number of Scans 1024
5 Receiver Gain 16384
6 Relaxation Delay 2.0000
7 Pulse Width 9.0000
8 Acquisition Time 1.0912
9 Spectrometer Frequency 125.76
10 Spectral Width 30030.0
11 Lowest Frequency -2439.7
12 Nucleus 13C
13 Acquired Size 32768
14 Spectral Size 32768
134.53
131.88
131.73
128.52
125.89
125.73
125.40
119.77
31.21
31.14
tBu
N
tBu
F3C
200 190 180 170 160 150 140 130 120 110 100 90 80 70 60 50 40 30 20 10 0
f1 (ppm)

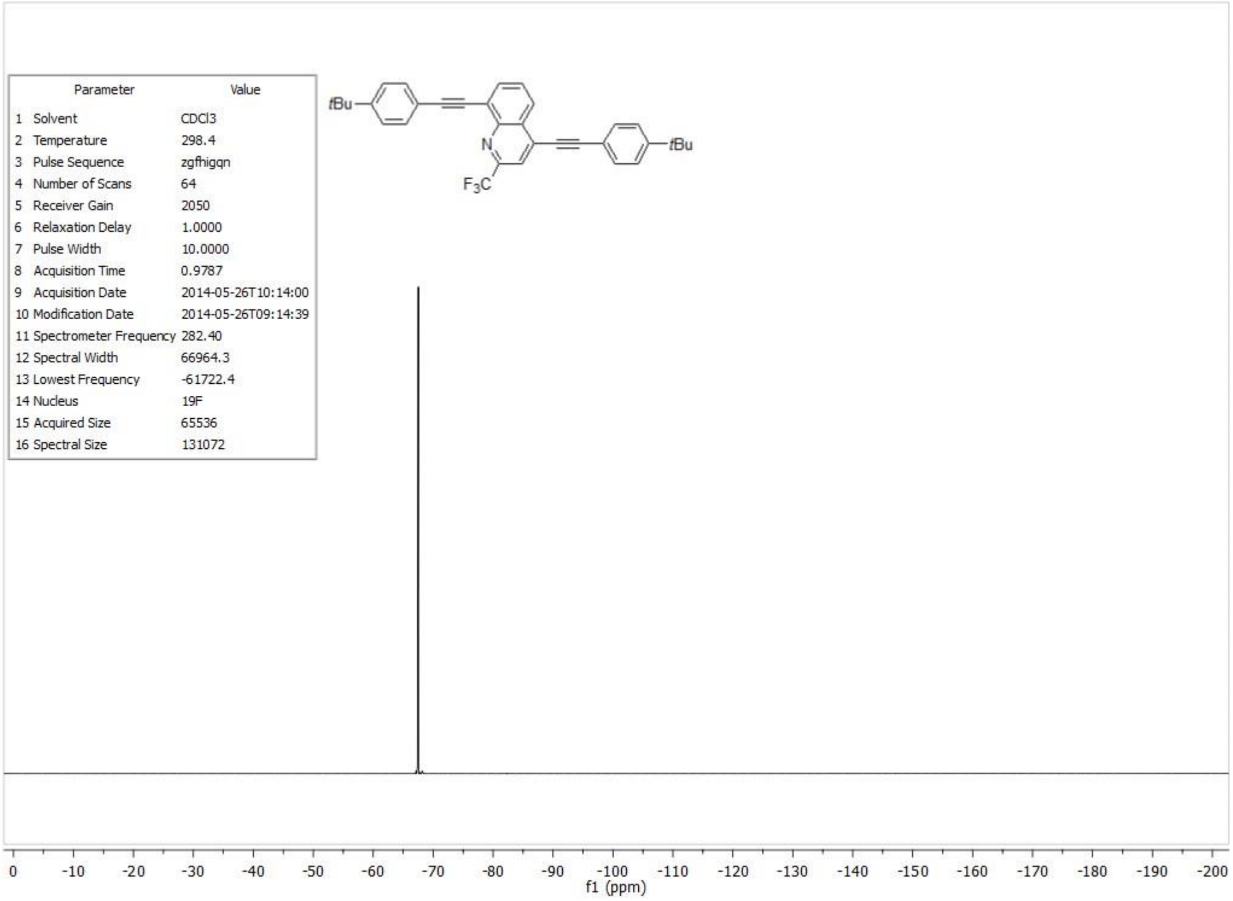
Parameter Value
1 Solvent CDCl3
2 Temperature 298.4
3 Pulse Sequence zgfhigqn
4 Number of Scans 64
5 Receiver Gain 2050
6 Relaxation Delay 1.0000
7 Pulse Width 10.0000
8 Acquisition Time 0.9787
9 Acquisition Date 2014-05-26T10:14:00
10 Modification Date 2014-05-26T09:14:39
11 Spectrometer Frequency 282.40
12 Spectral Width 66964.3
13 Lowest Frequency -61722.4
14 Nucleus 19F
15 Acquired Size 65536
16 Spectral Size 131072
tBu
N
F3C
tBu
0 -10 -20 -30 -40 -50 -60 -70 -80 -90 -100 -110 -120 -130 -140 -150 -160 -170 -180 -190 -200
f1 (ppm)

6d - 4,8-Bis(4‘-fluorophenylethynyl)-2-trifluoromethylquinoline

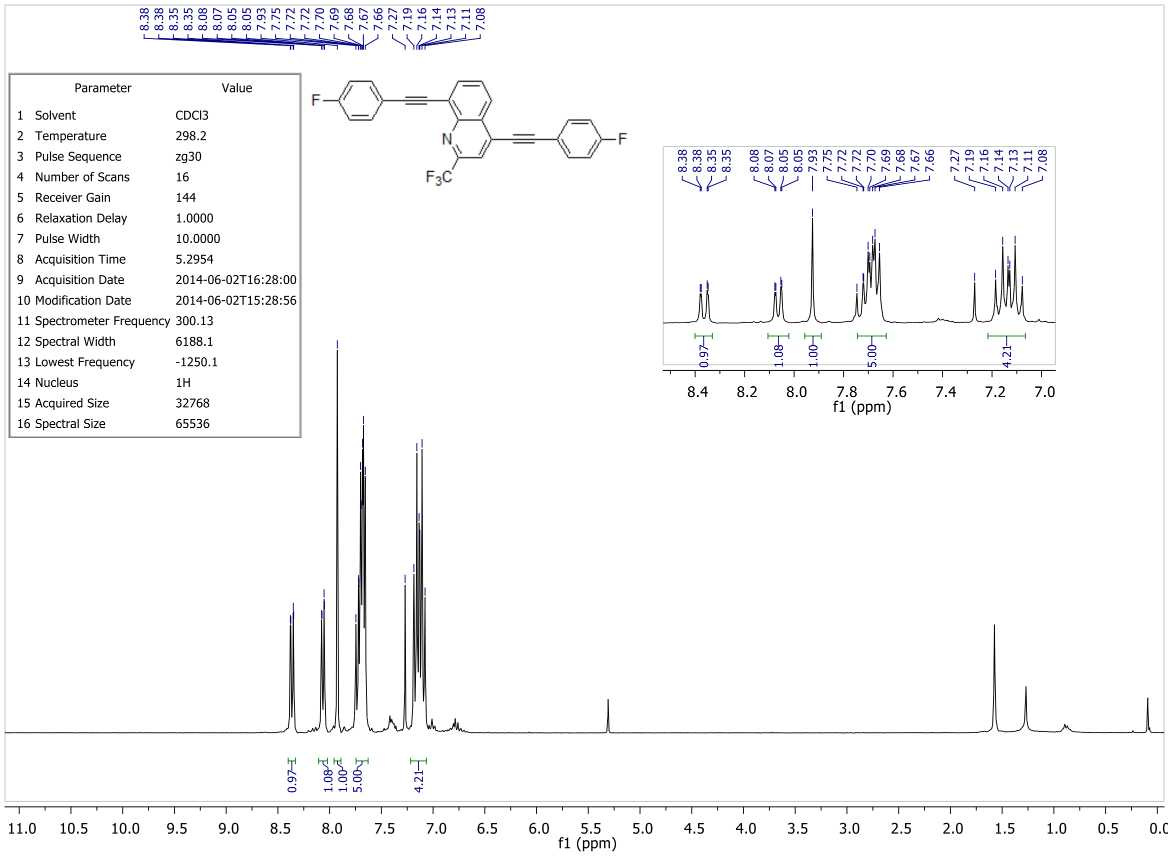

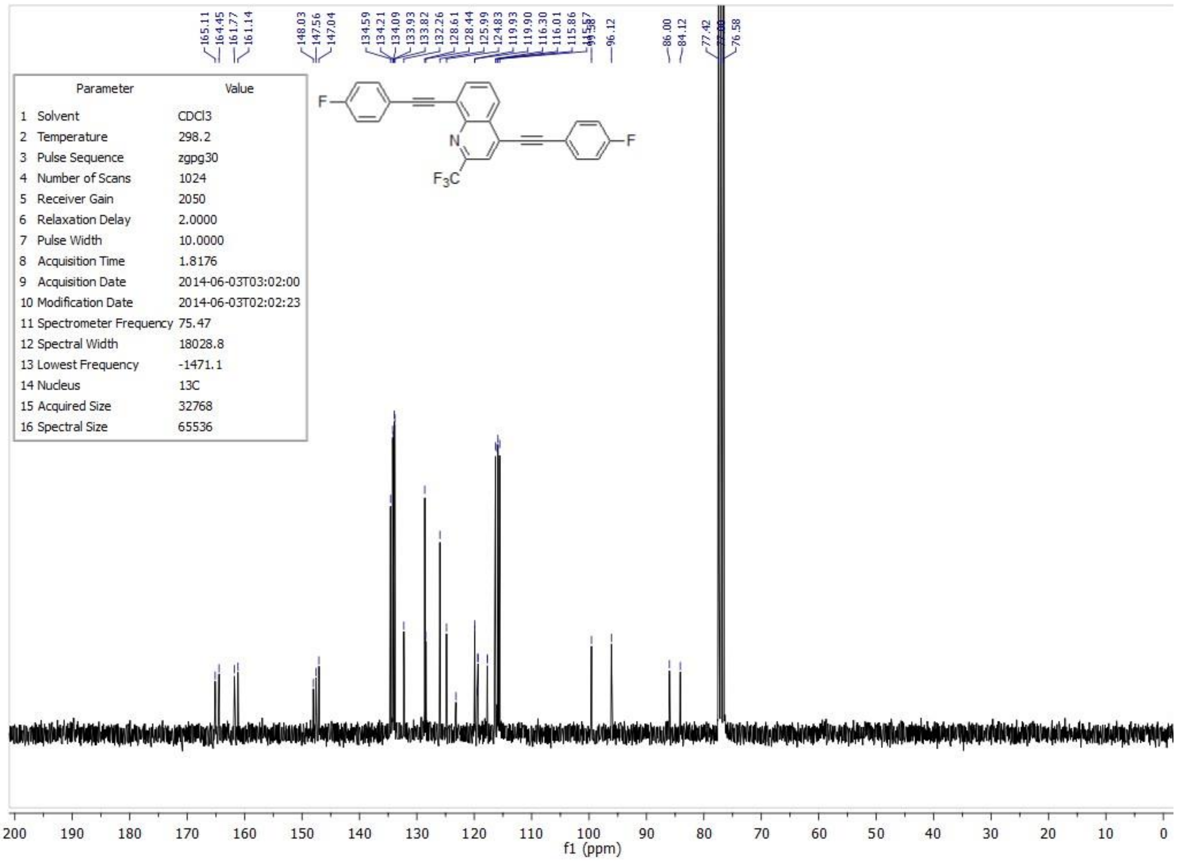

Parameter	Value
1 Solvent	CDCl3
2 Temperature	298.2
3 Pulse Sequence	zgpg30
4 Number of Scans	1024
5 Receiver Gain	2050
6 Relaxation Delay	2.0000
7 Pulse Width	10.0000
8 Acquisition Time	1.8176
9 Acquisition Date	2014-06-03T03:02:00
10 Modification Date	2014-06-03T02:02:23
11 Spectrometer Frequency	75.47
12 Spectral Width	18028.8
13 Lowest Frequency	-1471.1
14 Nucleus	13C
15 Acquired Size	32768
16 Spectral Size	65536

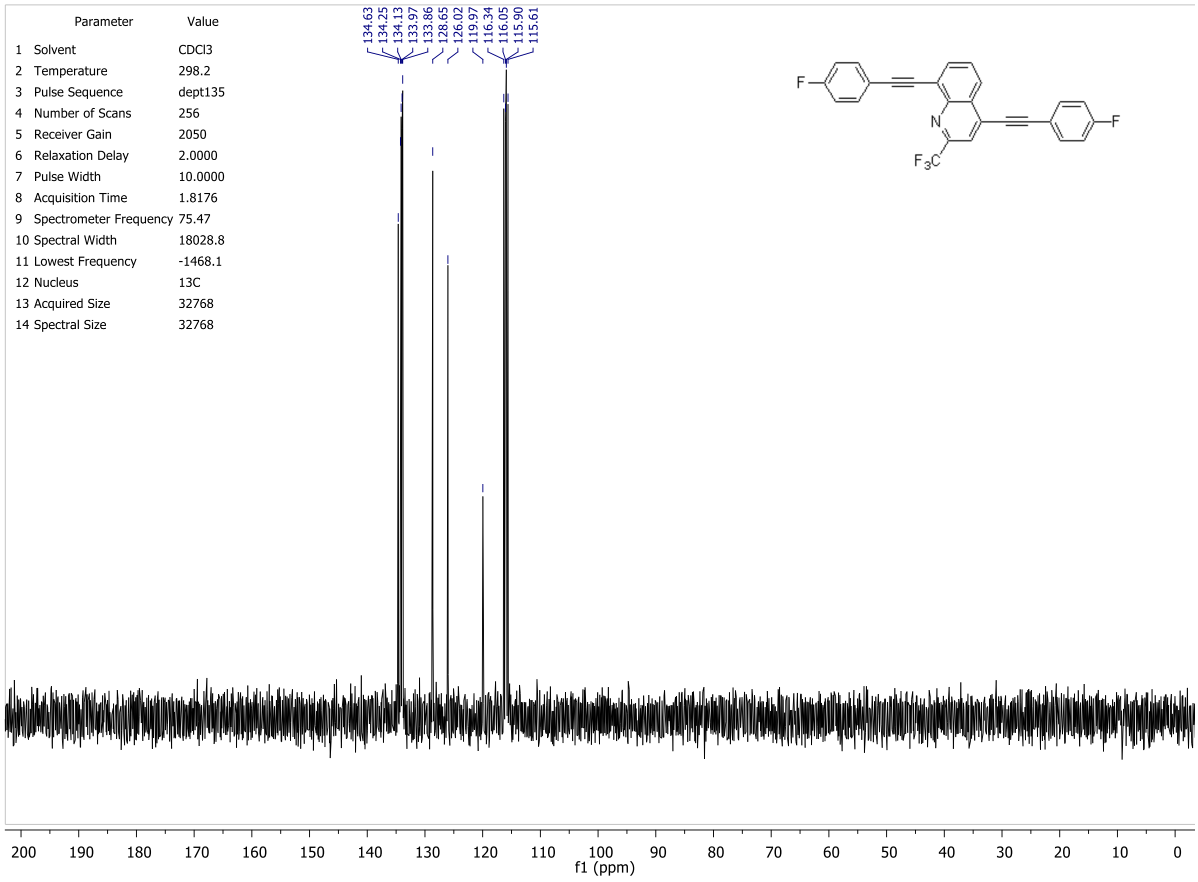

Parameter	Value
1 Solvent	CDCl3
2 Temperature	298.2
3 Pulse Sequence	dept135
4 Number of Scans	256
5 Receiver Gain	2050
6 Relaxation Delay	2.0000
7 Pulse Width	10.0000
8 Acquisition Time	1.8176
9 Spectrometer Frequency	75.47
10 Spectral Width	18028.8
11 Lowest Frequency	-1468.1
12 Nucleus	13C
13 Acquired Size	32768
14 Spectral Size	32768

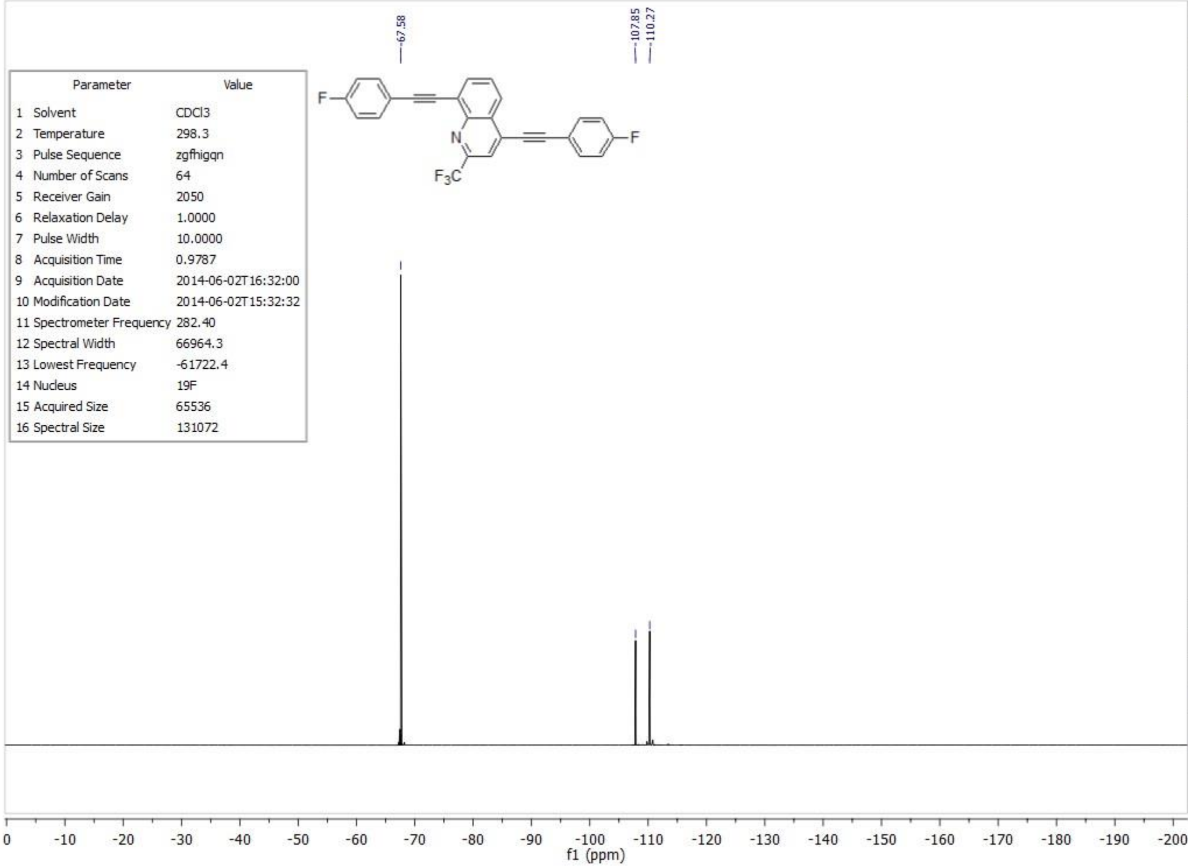
-67.58
-107.85
-110.27
Parameter Value
1 Solvent CDCl3
2 Temperature 298.3
3 Pulse Sequence zgfhigqn
4 Number of Scans 64
5 Receiver Gain 2050
6 Relaxation Delay 1.0000
7 Pulse Width 10.0000
8 Acquisition Time 0.9787
9 Acquisition Date 2014-06-02T16:32:00
10 Modification Date 2014-06-02T15:32:32
11 Spectrometer Frequency 282.40
12 Spectral Width 66964.3
13 Lowest Frequency -61722.4
14 Nucleus 19F
15 Acquired Size 65536
16 Spectral Size 131072
F
N
F3C
F
0 -10 -20 -30 -40 -50 -60 -70 -80 -90 -100 -110 -120 -130 -140 -150 -160 -170 -180 -190 -200
f1 (ppm)

6e - 4,8-Bis(4'-methoxyphenylethynyl)-2-trifluoromethylquinoline

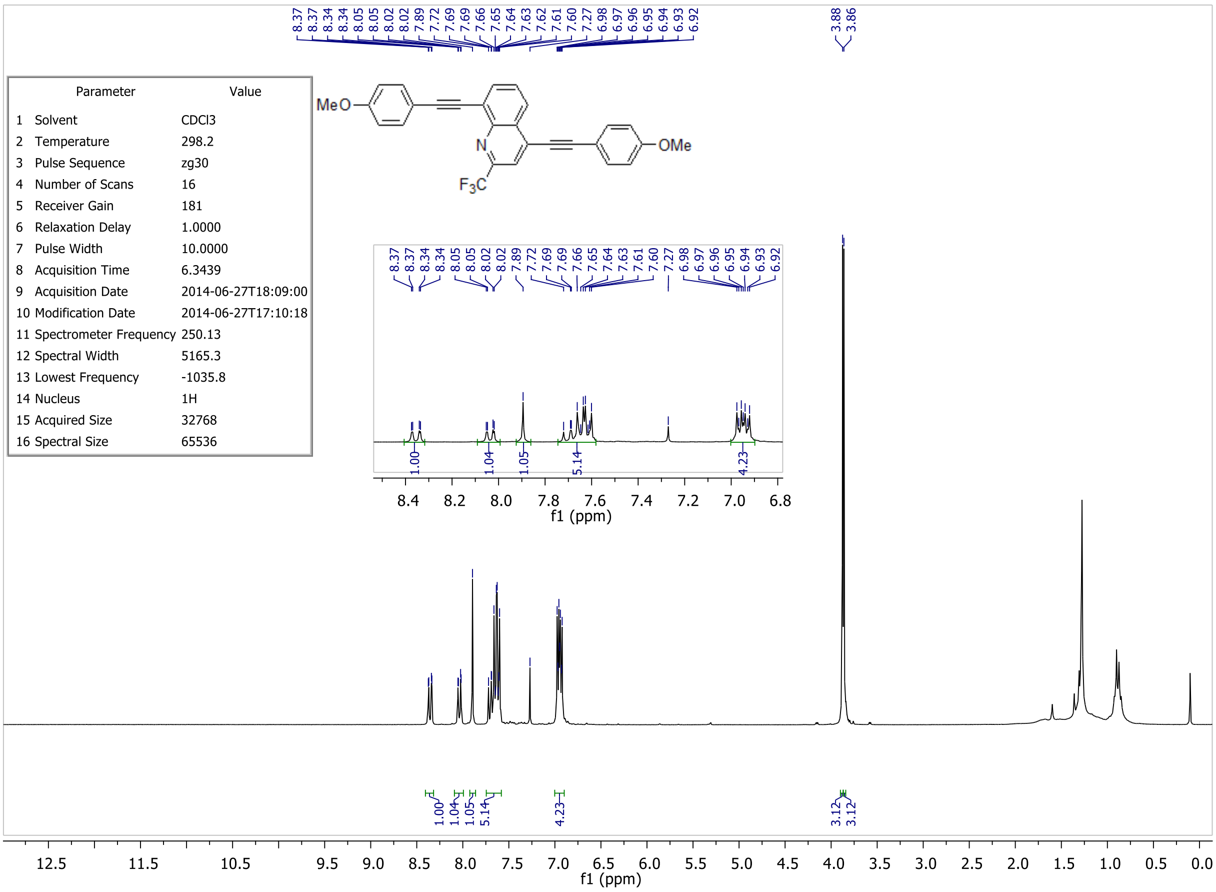

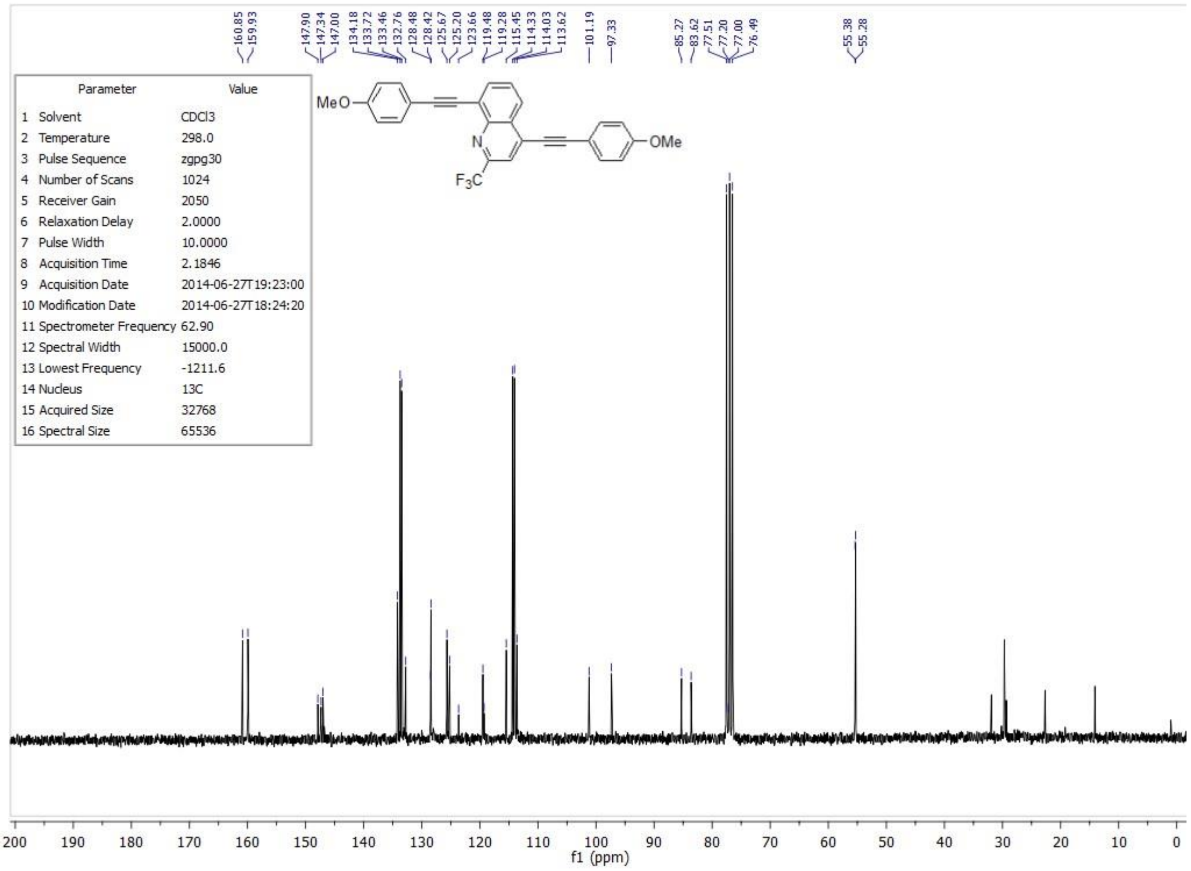
160.85
159.93
147.90
147.34
147.00
134.18
133.72
133.46
132.76
128.48
128.42
125.67
125.20
123.66
119.48
119.28
115.45
114.33
114.03
113.62
101.19
97.33
85.27
83.62
77.51
77.20
77.00
76.49
55.38
55.28
Parameter Value
1 Solvent CDCl3
2 Temperature 298.0
3 Pulse Sequence zgpg30
4 Number of Scans 1024
5 Receiver Gain 2050
6 Relaxation Delay 2.0000
7 Pulse Width 10.0000
8 Acquisition Time 2.1846
9 Acquisition Date 2014-06-27T19:23:00
10 Modification Date 2014-06-27T18:24:20
11 Spectrometer Frequency 62.90
12 Spectral Width 15000.0
13 Lowest Frequency -1211.6
14 Nucleus 13C
15 Acquired Size 32768
16 Spectral Size 65536
MeO
N
OMe
F3C
200 190 180 170 160 150 140 130 120 110 100 90 80 70 60 50 40 30 20 10 0
f1 (ppm)

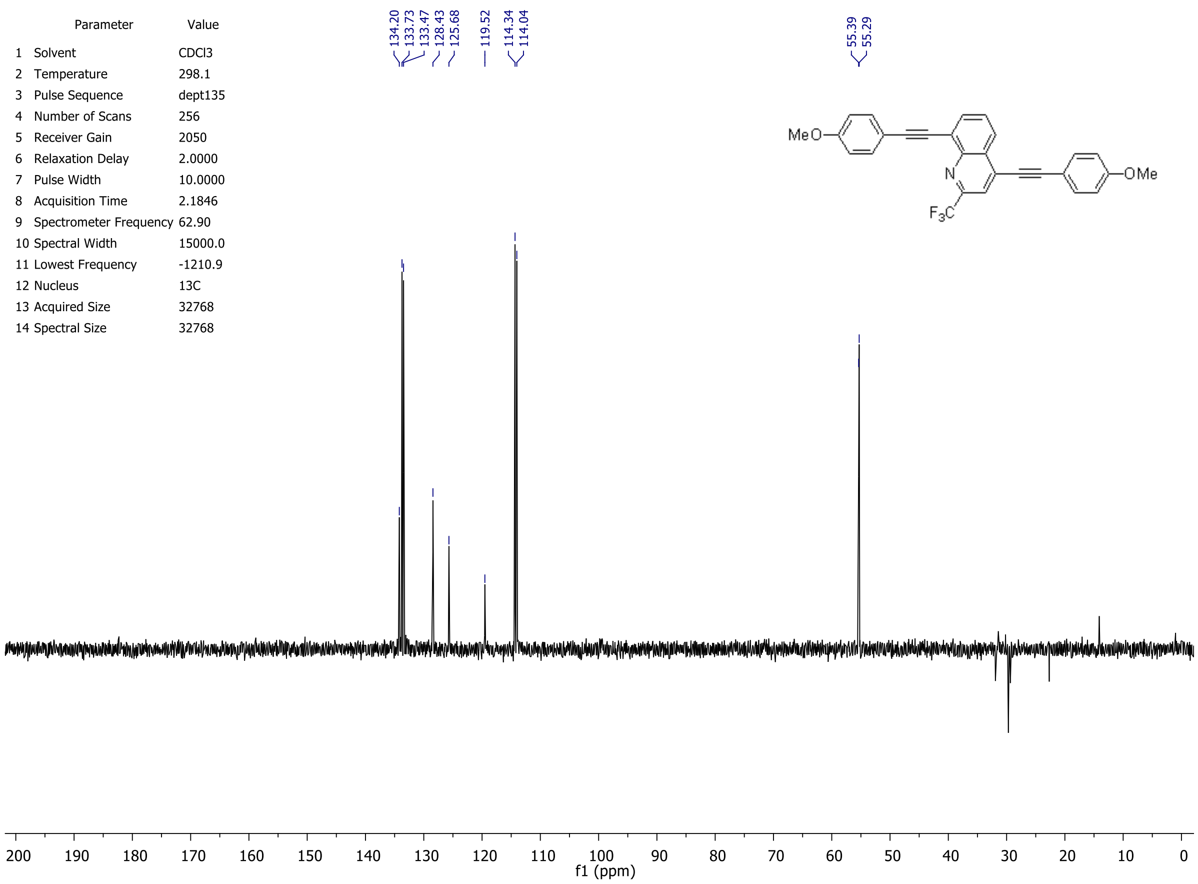
Parameter Value
1 Solvent CDCl3
2 Temperature 298.1
3 Pulse Sequence dept135
4 Number of Scans 256
5 Receiver Gain 2050
6 Relaxation Delay 2.0000
7 Pulse Width 10.0000
8 Acquisition Time 2.1846
9 Spectrometer Frequency 62.90
10 Spectral Width 15000.0
11 Lowest Frequency -1210.9
12 Nucleus 13C
13 Acquired Size 32768
14 Spectral Size 32768
134.20
133.73
133.47
128.43
125.68
119.52
114.34
114.04
55.39
55.29
MeO
N
OMe
F3C
200 190 180 170 160 150 140 130 120 110 100 90 80 70 60 50 40 30 20 10 0
f1 (ppm)

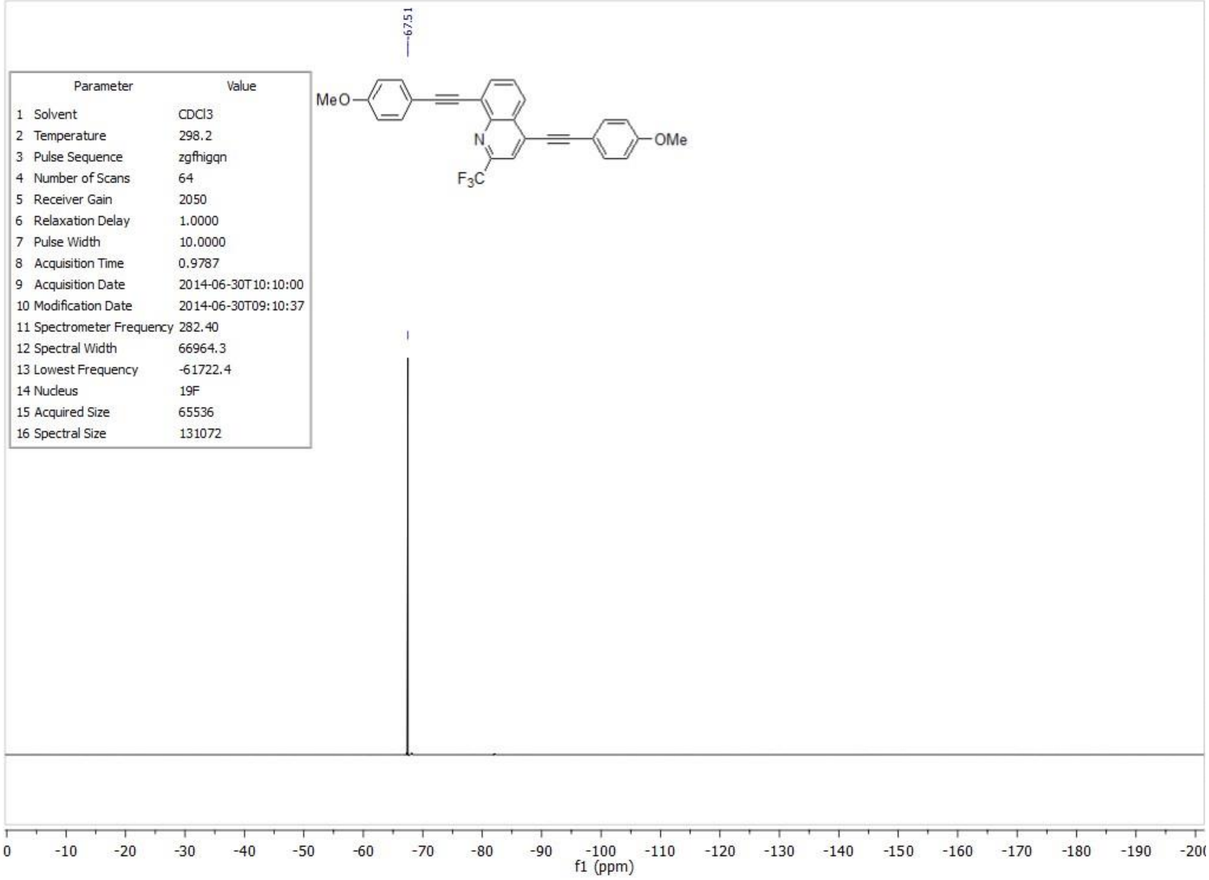

-67.51
Parameter Value
1 Solvent CDCl3
2 Temperature 298.2
3 Pulse Sequence zgfhigqn
4 Number of Scans 64
5 Receiver Gain 2050
6 Relaxation Delay 1.0000
7 Pulse Width 10.0000
8 Acquisition Time 0.9787
9 Acquisition Date 2014-06-30T10:10:00
10 Modification Date 2014-06-30T09:10:37
11 Spectrometer Frequency 282.40
12 Spectral Width 66964.3
13 Lowest Frequency -61722.4
14 Nucleus 19F
15 Acquired Size 65536
16 Spectral Size 131072
MeO
OMe
N
F3C
0 -10 -20 -30 -40 -50 -60 -70 -80 -90 -100 -110 -120 -130 -140 -150 -160 -170 -180 -190 -200
f1 (ppm)

6f - 4,8-Bis(3'-thienylethynyl)-2-trifluoromethylquinoline

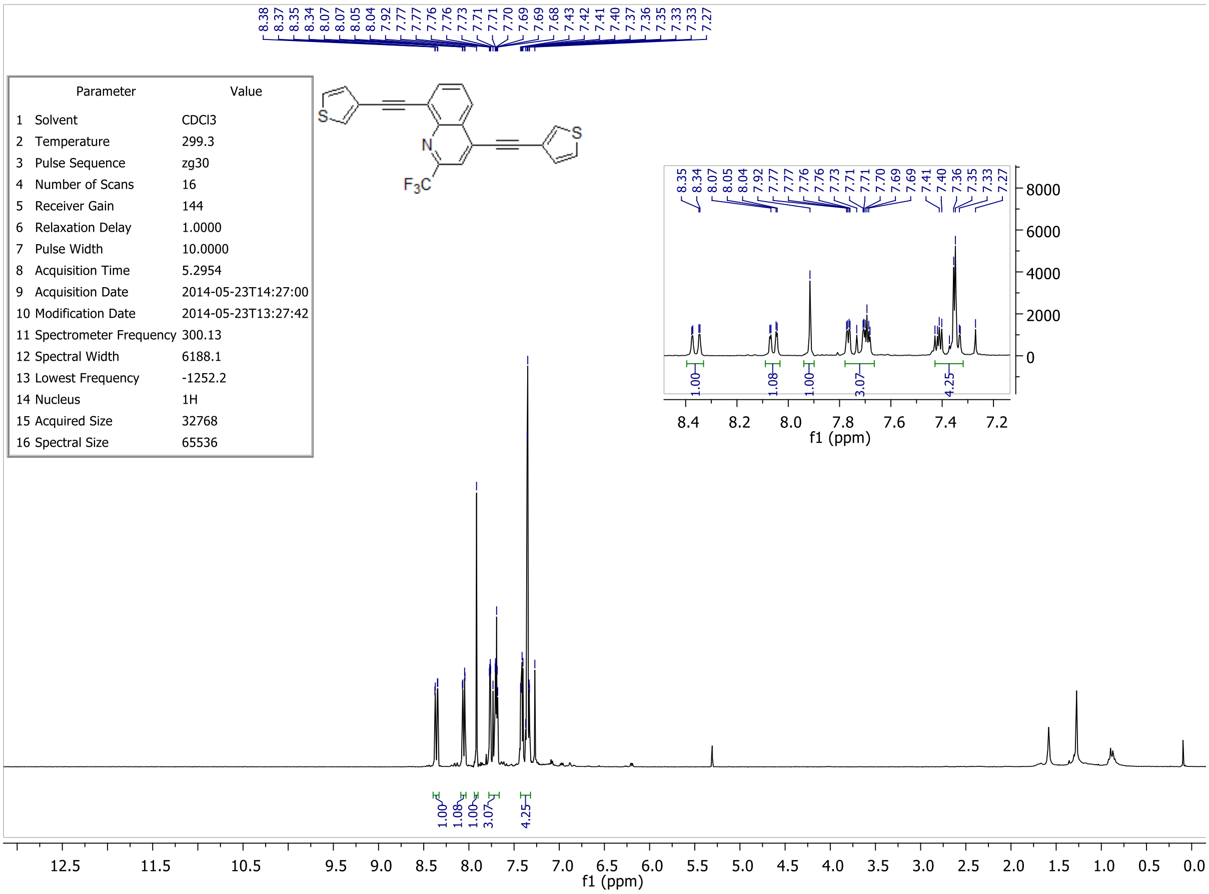

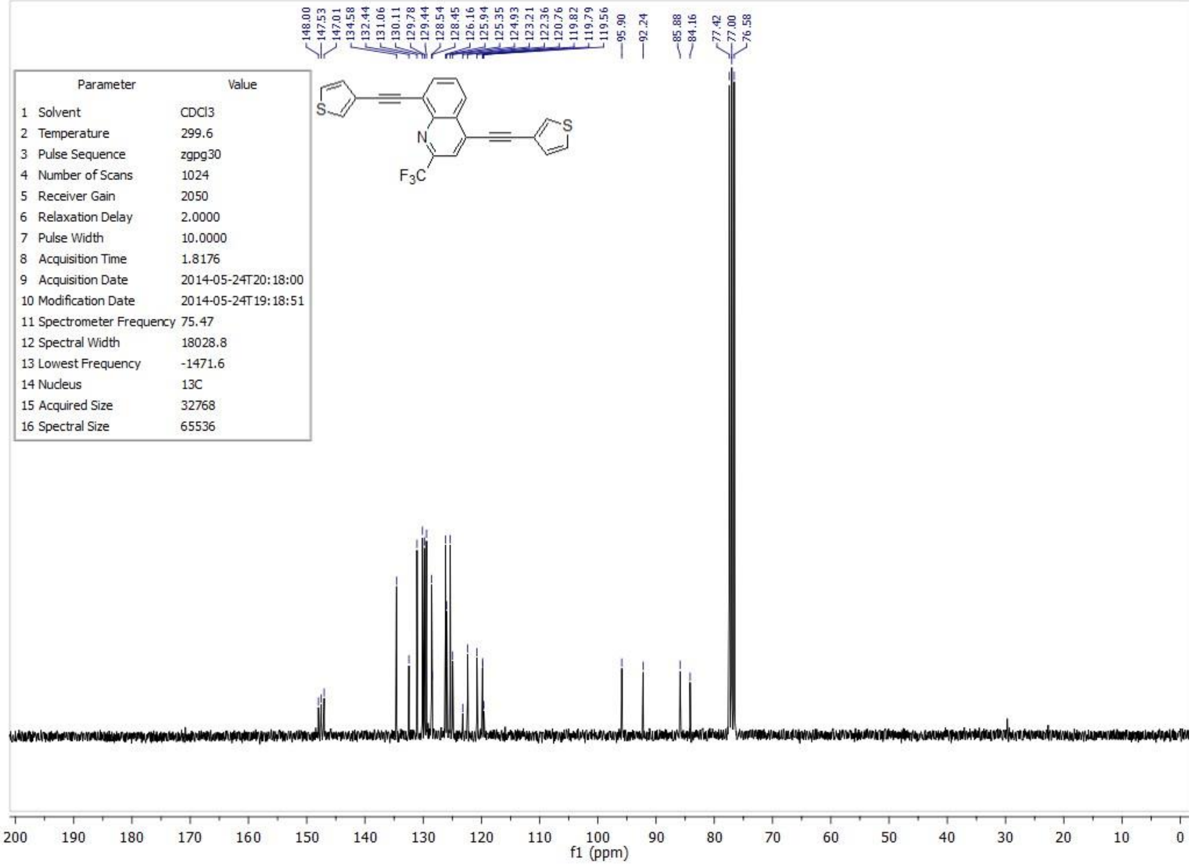
148.00
147.53
147.01
134.58
132.44
131.06
130.11
129.78
129.44
128.54
128.45
126.16
125.94
125.35
124.93
123.21
122.36
120.76
119.82
119.79
119.56
95.90
92.24
85.88
84.16
77.42
77.00
76.58
Parameter Value
1 Solvent CDCl3
2 Temperature 299.6
3 Pulse Sequence zgpg30
4 Number of Scans 1024
5 Receiver Gain 2050
6 Relaxation Delay 2.0000
7 Pulse Width 10.0000
8 Acquisition Time 1.8176
9 Acquisition Date 2014-05-24T20:18:00
10 Modification Date 2014-05-24T19:18:51
11 Spectrometer Frequency 75.47
12 Spectral Width 18028.8
13 Lowest Frequency -1471.6
14 Nucleus 13C
15 Acquired Size 32768
16 Spectral Size 65536
S
S
N
F3C
200 190 180 170 160 150 140 130 120 110 100 90 80 70 60 50 40 30 20 10 0
f1 (ppm)

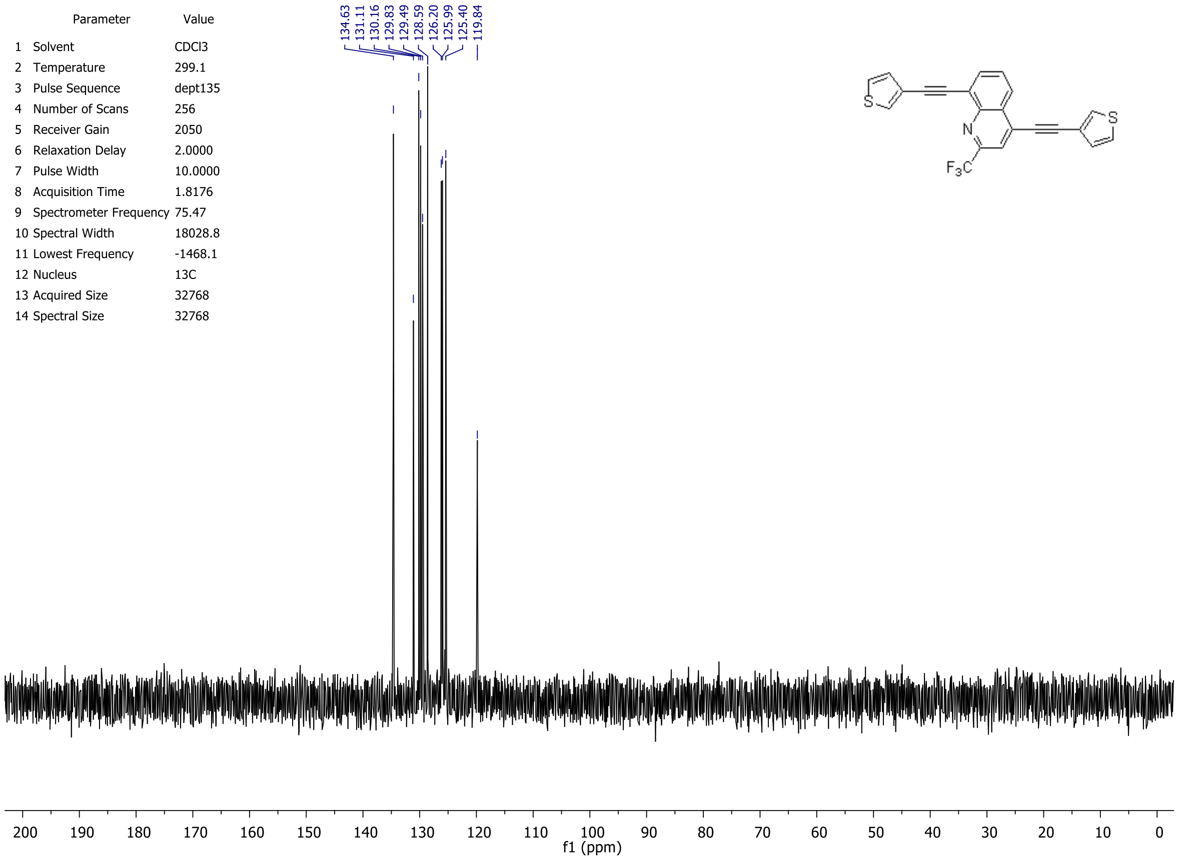
Parameter Value
1 Solvent CDCl3
2 Temperature 299.1
3 Pulse Sequence dept135
4 Number of Scans 256
5 Receiver Gain 2050
6 Relaxation Delay 2.0000
7 Pulse Width 10.0000
8 Acquisition Time 1.8176
9 Spectrometer Frequency 75.47
10 Spectral Width 18028.8
11 Lowest Frequency -1468.1
12 Nucleus 13C
13 Acquired Size 32768
14 Spectral Size 32768
134.63
131.11
130.16
129.83
129.49
128.59
126.20
125.99
125.40
119.84
S
S
N
F3C
200 190 180 170 160 150 140 130 120 110 100 90 80 70 60 50 40 30 20 10 0
f1 (ppm)

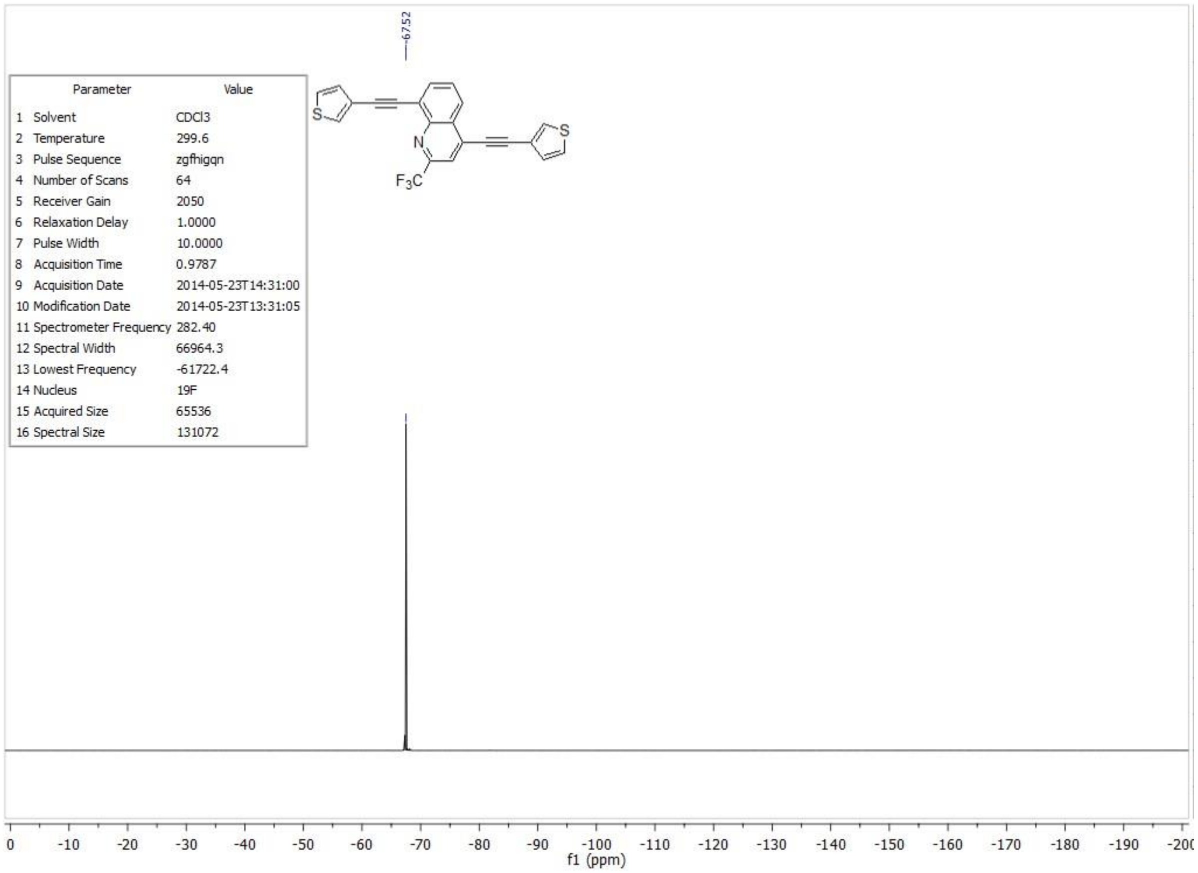

-67.52
Parameter Value
1 Solvent CDCl3
2 Temperature 299.6
3 Pulse Sequence zgfhigqn
4 Number of Scans 64
5 Receiver Gain 2050
6 Relaxation Delay 1.0000
7 Pulse Width 10.0000
8 Acquisition Time 0.9787
9 Acquisition Date 2014-05-23T14:31:00
10 Modification Date 2014-05-23T13:31:05
11 Spectrometer Frequency 282.40
12 Spectral Width 66964.3
13 Lowest Frequency -61722.4
14 Nucleus 19F
15 Acquired Size 65536
16 Spectral Size 131072
S
N
S
F3C
0 -10 -20 -30 -40 -50 -60 -70 -80 -90 -100 -110 -120 -130 -140 -150 -160 -170 -180 -190 -200
f1 (ppm)

6g - 4,8-Bis(cyclopropylethynyl)-2-trifluoromethylquinoline

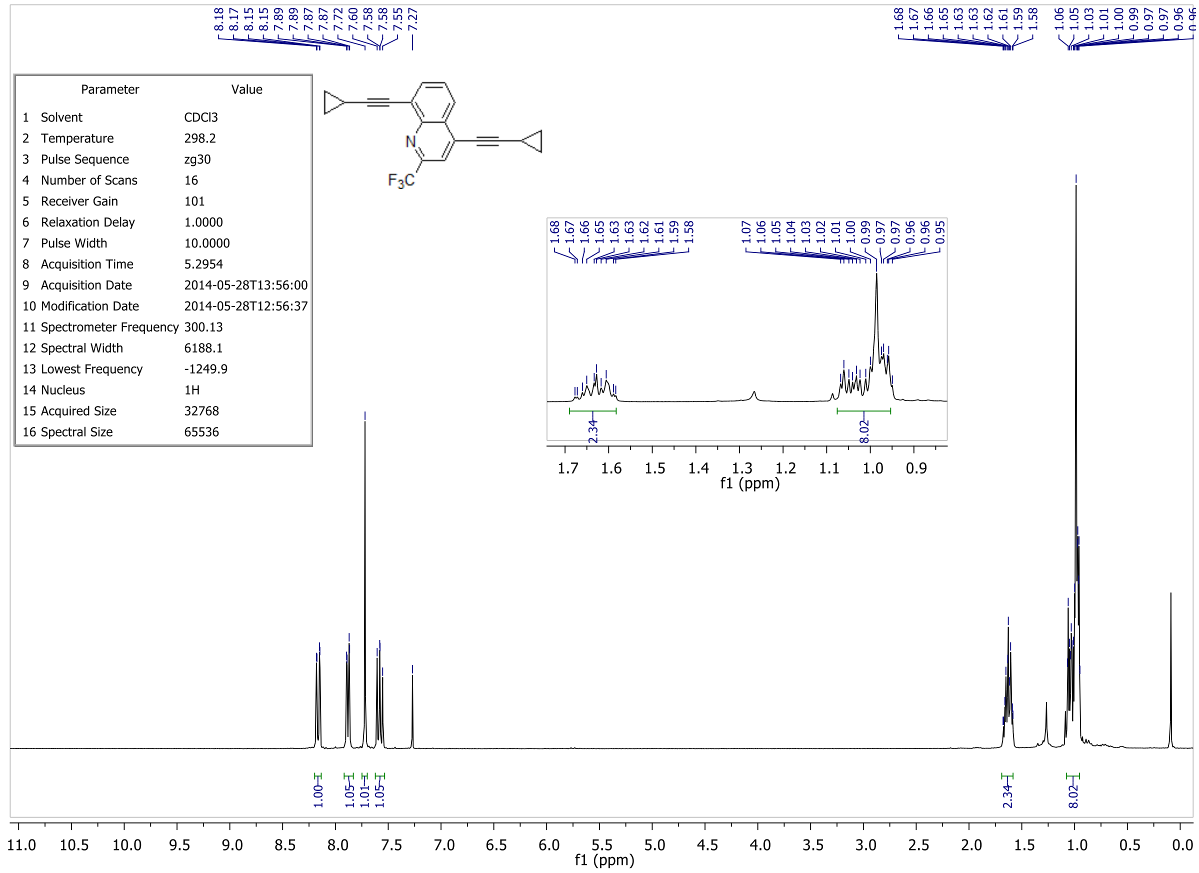

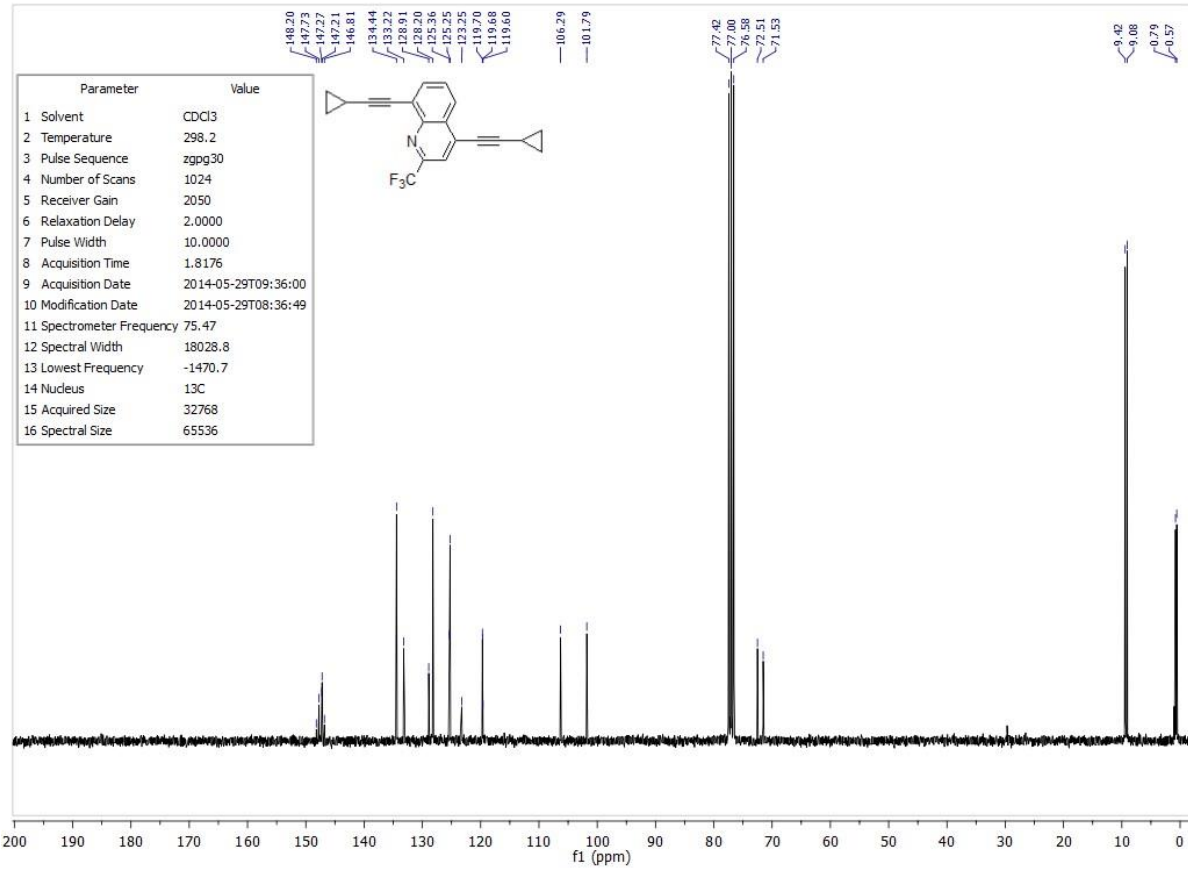

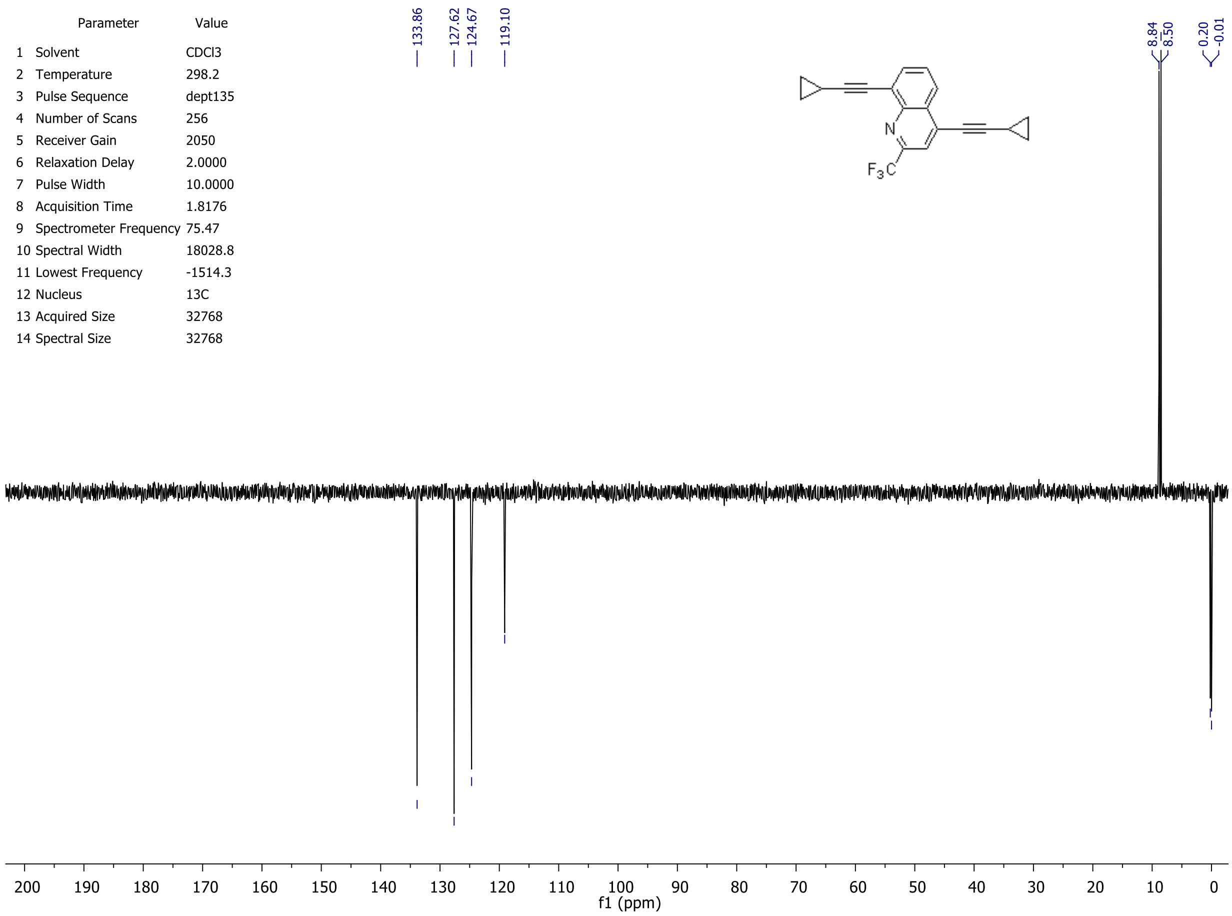

	Parameter	Value
1	Solvent	CDCl3
2	Temperature	298.2
3	Pulse Sequence	dept135
4	Number of Scans	256
5	Receiver Gain	2050
6	Relaxation Delay	2.0000
7	Pulse Width	10.0000
8	Acquisition Time	1.8176
9	Spectrometer Frequency	75.47
10	Spectral Width	18028.8
11	Lowest Frequency	-1514.3
12	Nucleus	13C
13	Acquired Size	32768
14	Spectral Size	32768

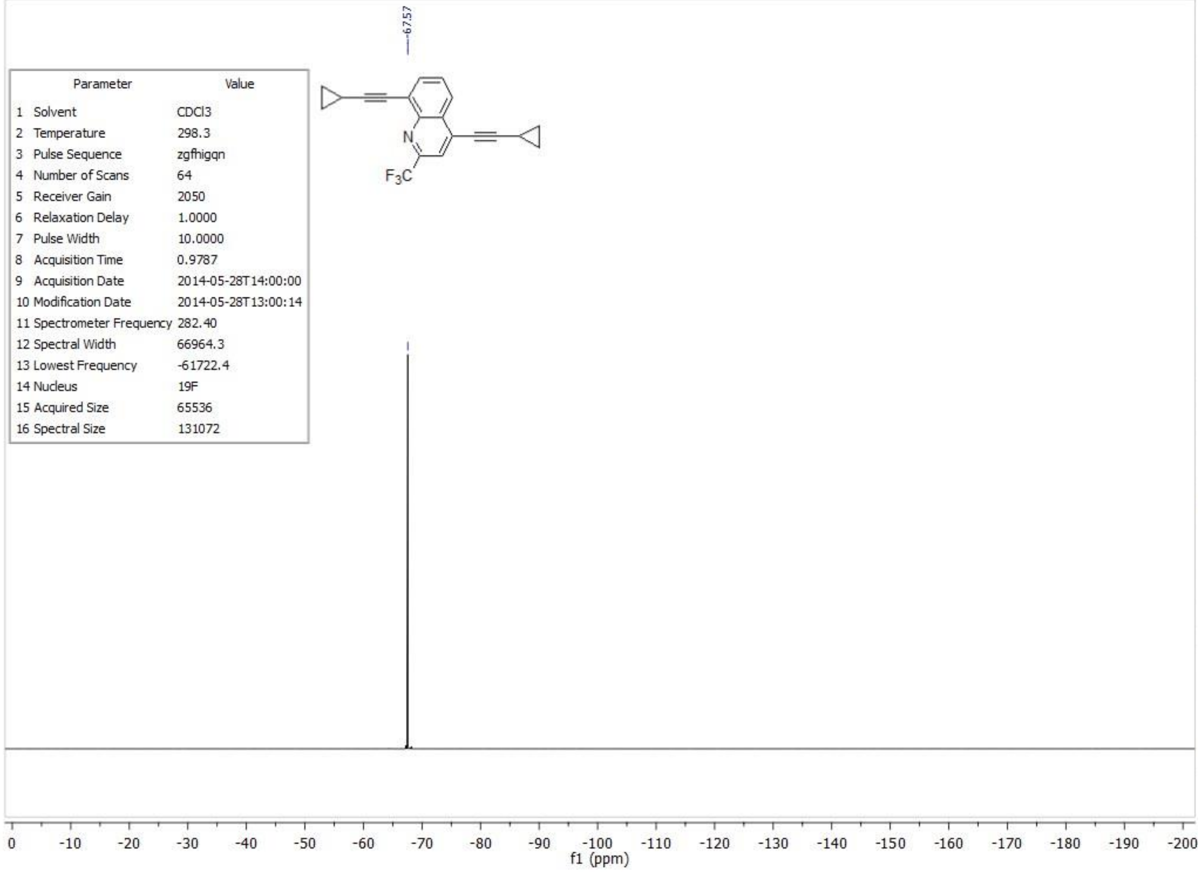

	Parameter	Value
1	Solvent	CDCl3
2	Temperature	298.3
3	Pulse Sequence	zgfhigqn
4	Number of Scans	64
5	Receiver Gain	2050
6	Relaxation Delay	1.0000
7	Pulse Width	10.0000
8	Acquisition Time	0.9787
9	Acquisition Date	2014-05-28T14:00:00
10	Modification Date	2014-05-28T13:00:14
11	Spectrometer Frequency	282.40
12	Spectral Width	66964.3
13	Lowest Frequency	-61722.4
14	Nucleus	19F
15	Acquired Size	65536
16	Spectral Size	131072

9a - 4,6-Bis(phenylethynyl)-2-trifluoromethylquinoline

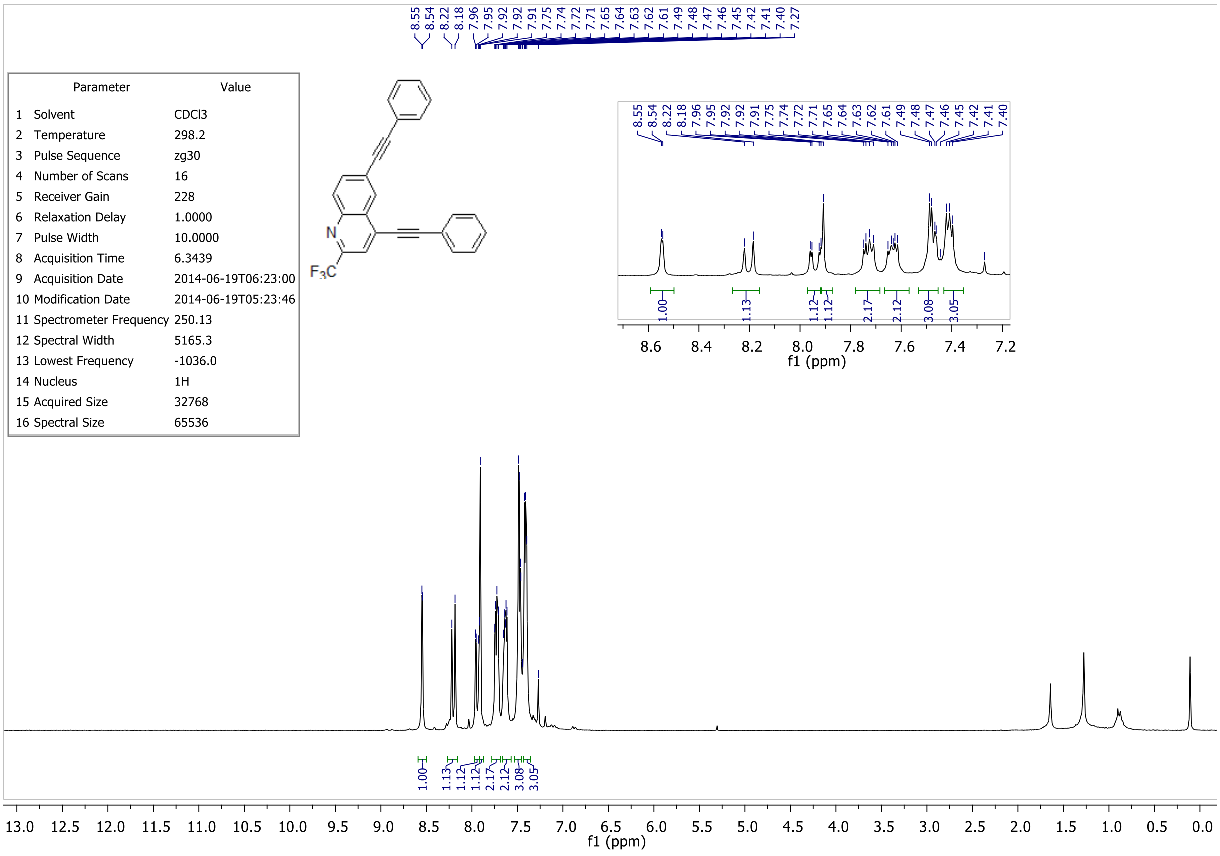

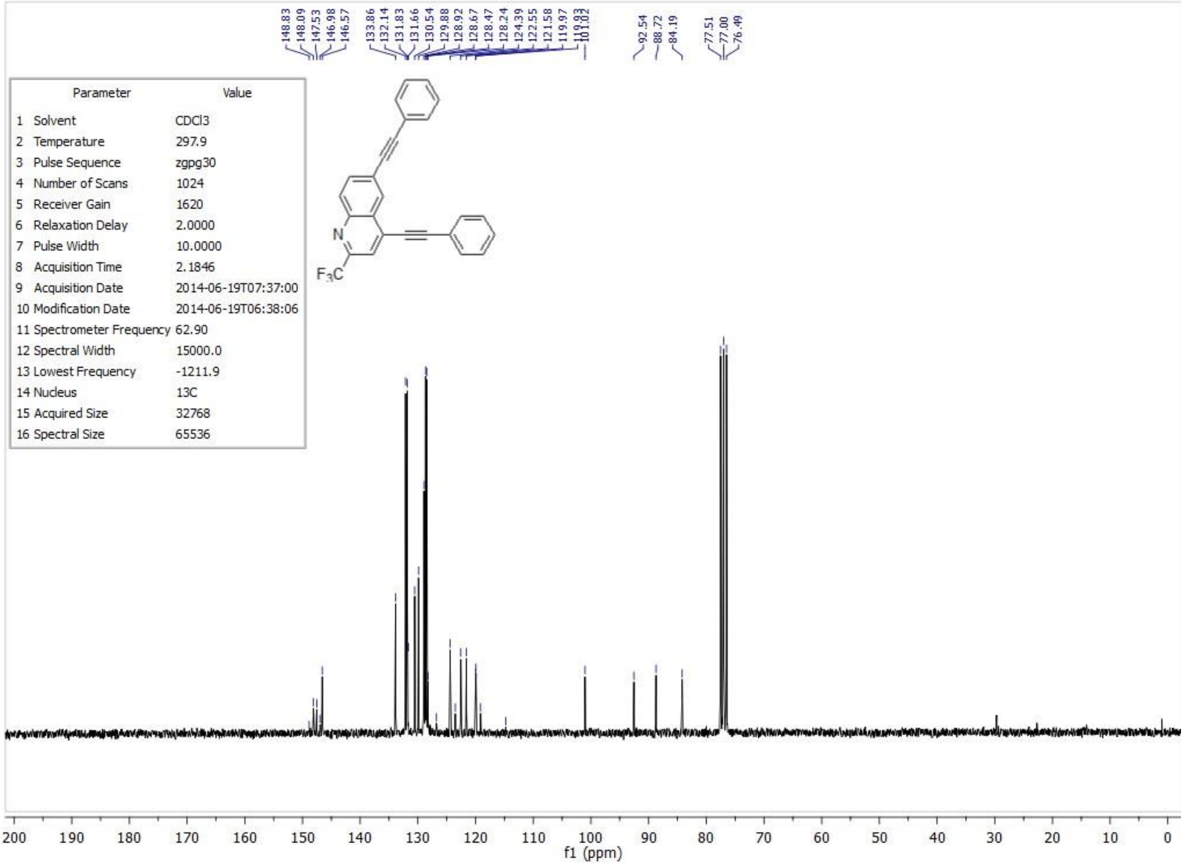

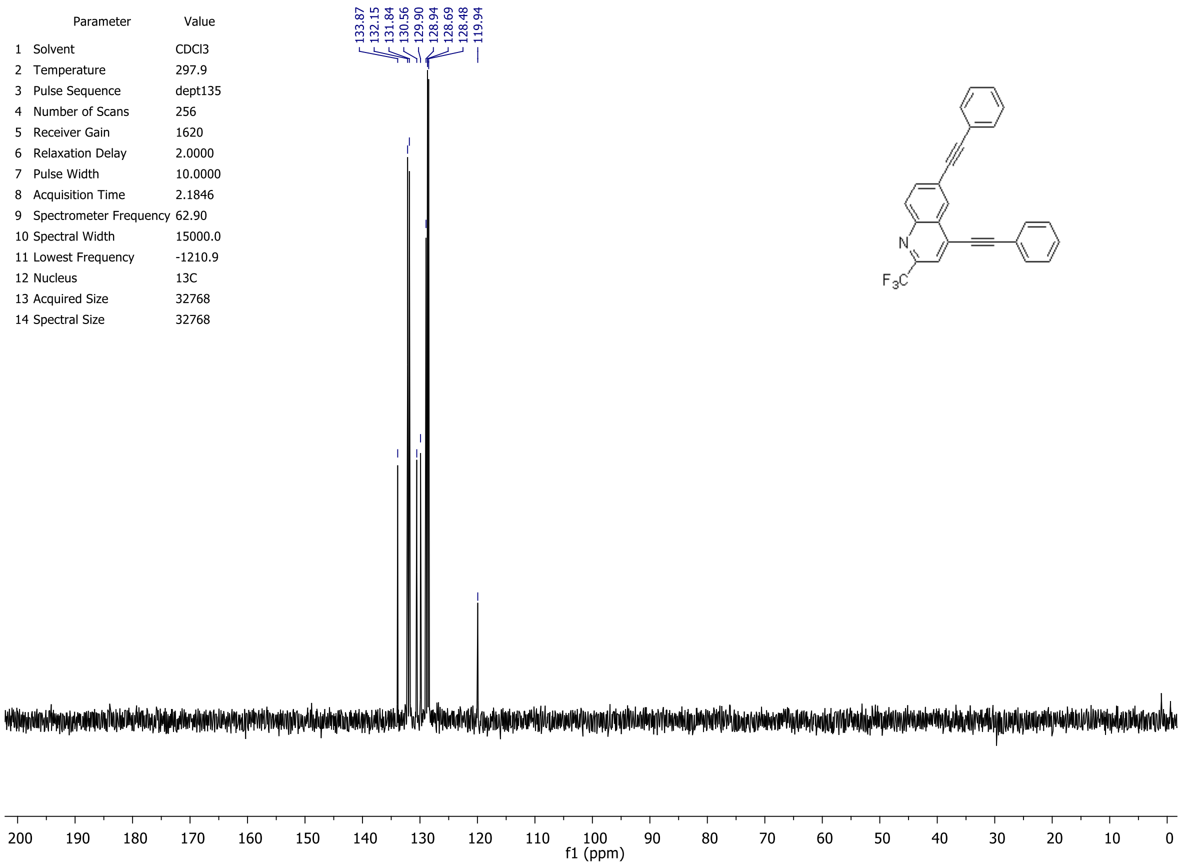

	Parameter	Value
1	Solvent	CDCl3
2	Temperature	297.9
3	Pulse Sequence	dept135
4	Number of Scans	256
5	Receiver Gain	1620
6	Relaxation Delay	2.0000
7	Pulse Width	10.0000
8	Acquisition Time	2.1846
9	Spectrometer Frequency	62.90
10	Spectral Width	15000.0
11	Lowest Frequency	-1210.9
12	Nucleus	13C
13	Acquired Size	32768
14	Spectral Size	32768

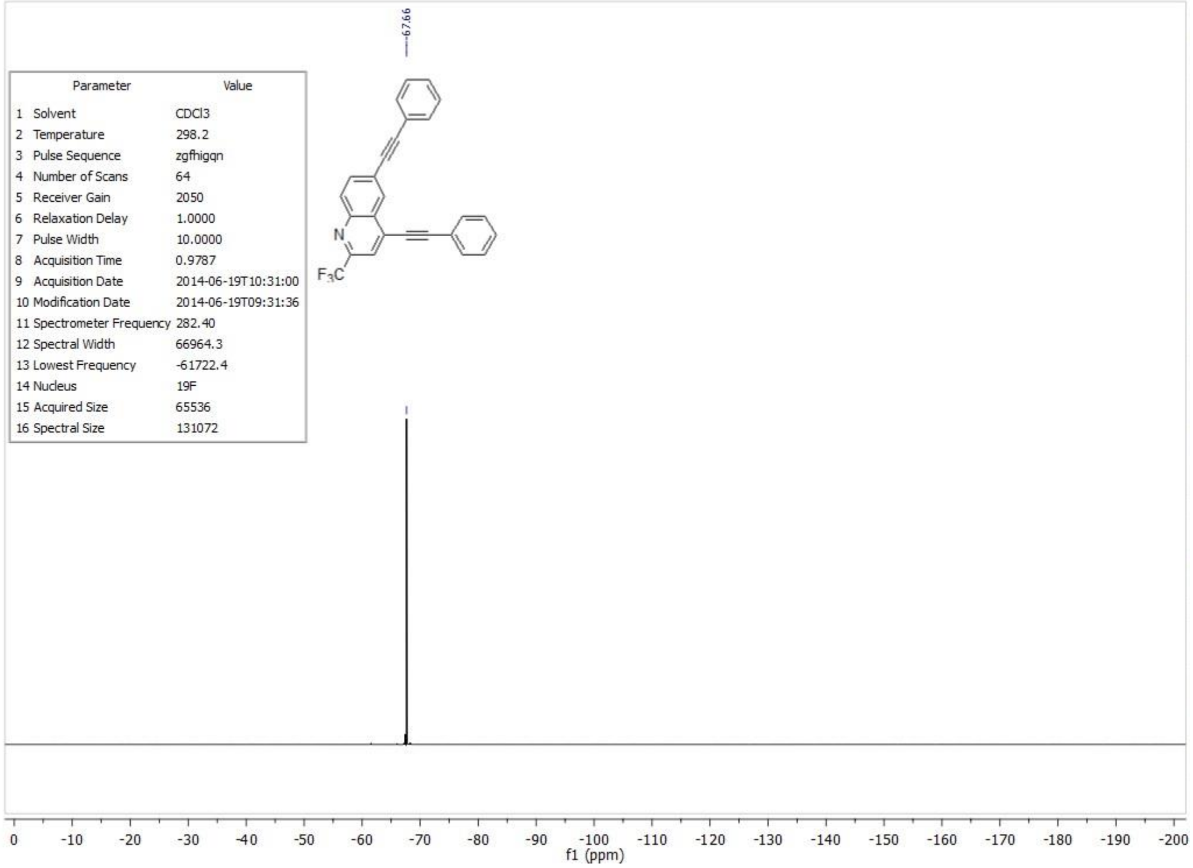

	Parameter	Value
1	Solvent	CDCl3
2	Temperature	298.2
3	Pulse Sequence	zgfhigqn
4	Number of Scans	64
5	Receiver Gain	2050
6	Relaxation Delay	1.0000
7	Pulse Width	10.0000
8	Acquisition Time	0.9787
9	Acquisition Date	2014-06-19T10:31:00
10	Modification Date	2014-06-19T09:31:36
11	Spectrometer Frequency	282.40
12	Spectral Width	66964.3
13	Lowest Frequency	-61722.4
14	Nucleus	19F
15	Acquired Size	65536
16	Spectral Size	131072

9b - 4,6-Bis(4'-tert-butylphenylethynyl)-2-trifluoromethylquinoline

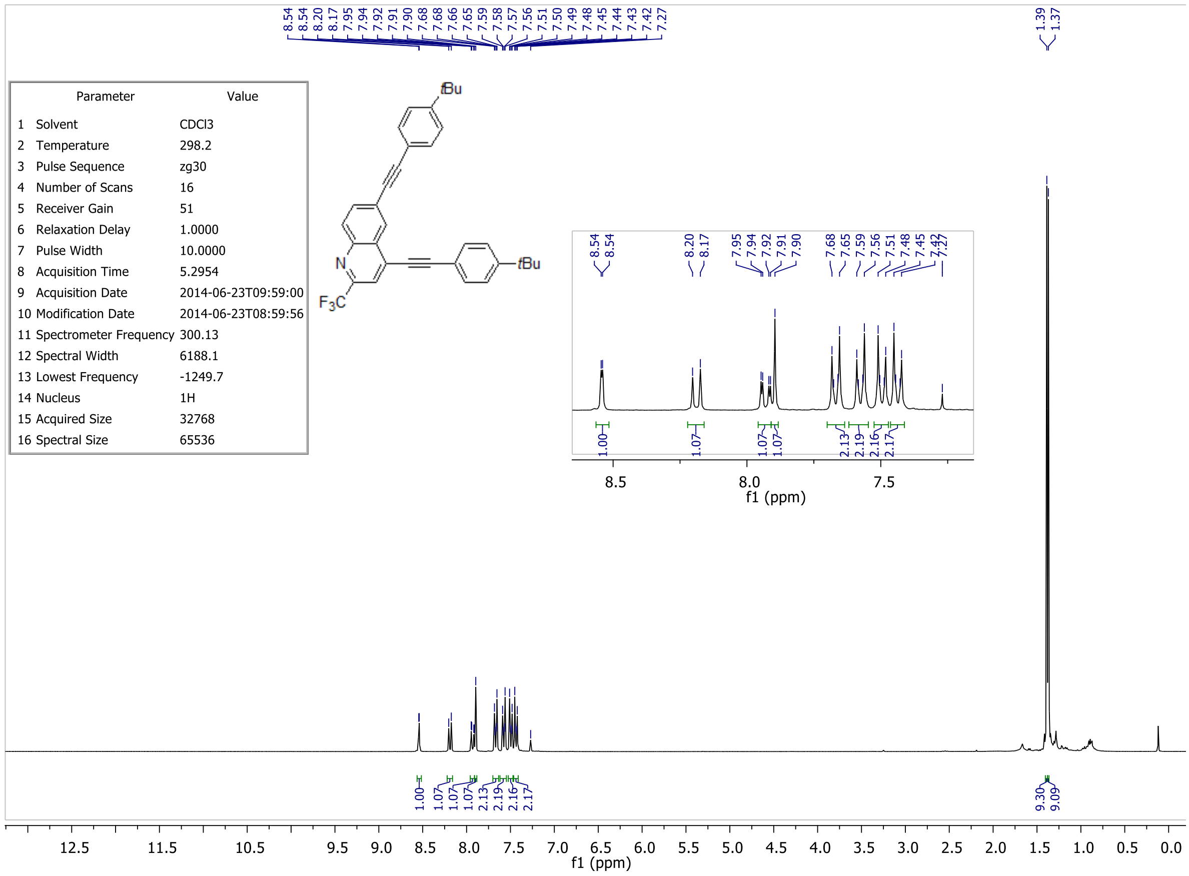

	Parameter	Value
1	Solvent	CDCl3
2	Temperature	298.2
3	Pulse Sequence	zg30
4	Number of Scans	16
5	Receiver Gain	51
6	Relaxation Delay	1.0000
7	Pulse Width	10.0000
8	Acquisition Time	5.2954
9	Acquisition Date	2014-06-23T09:59:00
10	Modification Date	2014-06-23T08:59:56
11	Spectrometer Frequency	300.13
12	Spectral Width	6188.1
13	Lowest Frequency	-1249.7
14	Nucleus	1H
15	Acquired Size	32768
16	Spectral Size	65536

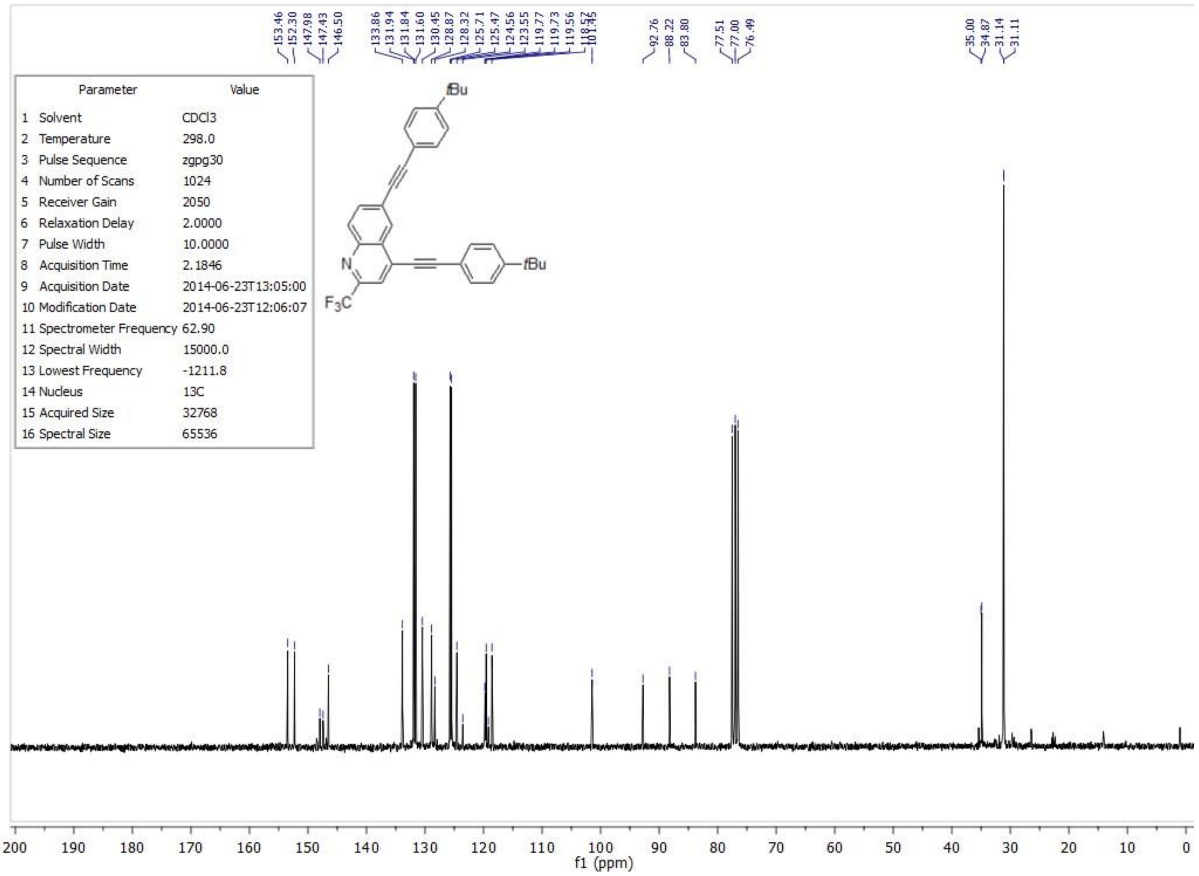
153.46
152.30
147.98
147.43
146.50
133.86
131.94
131.84
131.60
130.45
128.87
128.32
125.71
125.47
124.56
123.55
119.77
119.73
119.56
101.45
92.76
88.22
83.80
77.51
77.00
76.49
35.00
34.87
31.14
31.11
Parameter Value
1 Solvent CDCl3
2 Temperature 298.0
3 Pulse Sequence zgpg30
4 Number of Scans 1024
5 Receiver Gain 2050
6 Relaxation Delay 2.0000
7 Pulse Width 10.0000
8 Acquisition Time 2.1846
9 Acquisition Date 2014-06-23T13:05:00
10 Modification Date 2014-06-23T12:06:07
11 Spectrometer Frequency 62.90
12 Spectral Width 15000.0
13 Lowest Frequency -1211.8
14 Nucleus 13C
15 Acquired Size 32768
16 Spectral Size 65536
tBu
N
tBu
F3C
f1 (ppm)

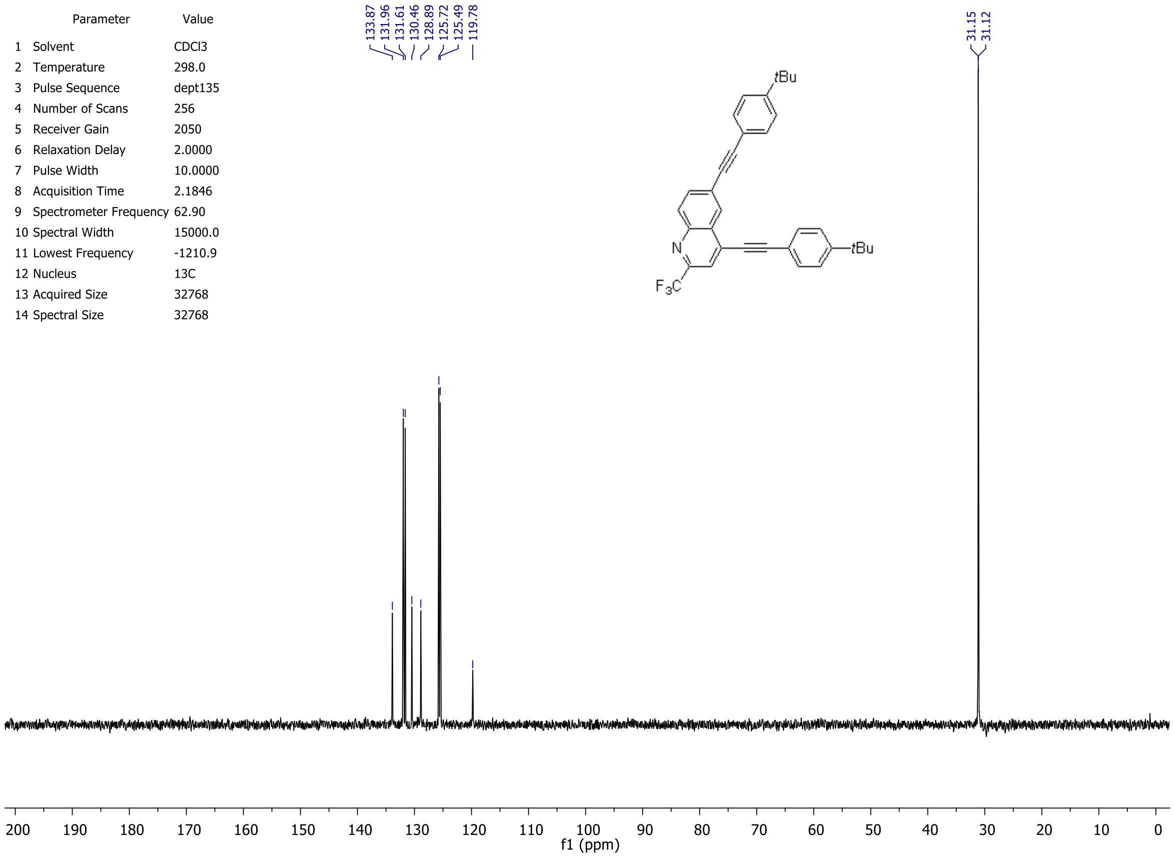
Parameter Value
1 Solvent CDCl3
2 Temperature 298.0
3 Pulse Sequence dept135
4 Number of Scans 256
5 Receiver Gain 2050
6 Relaxation Delay 2.0000
7 Pulse Width 10.0000
8 Acquisition Time 2.1846
9 Spectrometer Frequency 62.90
10 Spectral Width 15000.0
11 Lowest Frequency -1210.9
12 Nucleus 13C
13 Acquired Size 32768
14 Spectral Size 32768
133.87
131.96
131.61
130.46
128.89
125.72
125.49
119.78
31.15
31.12
tBu
N
tBu
F3C
f1 (ppm)

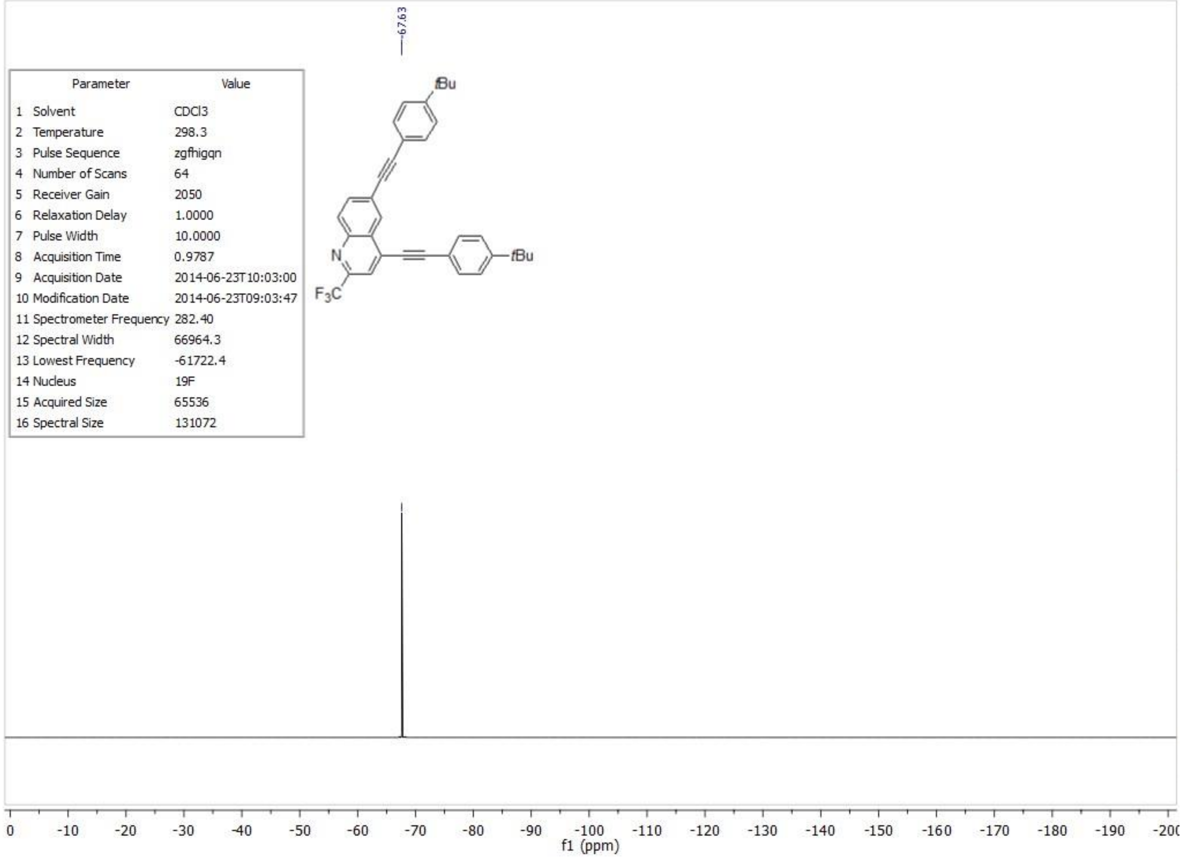

9d - 4,6-Bis(4'-fluorophenylethynyl)-2-trifluoromethylquinoline

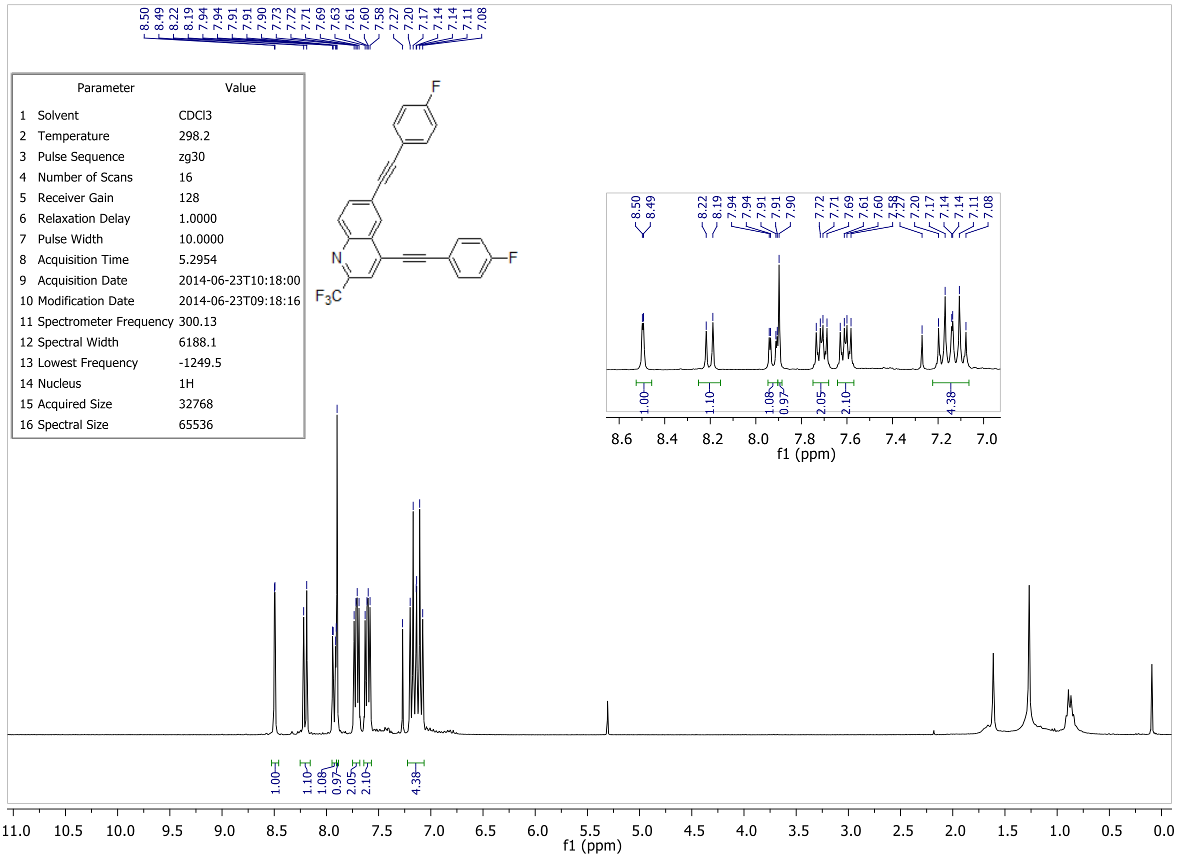

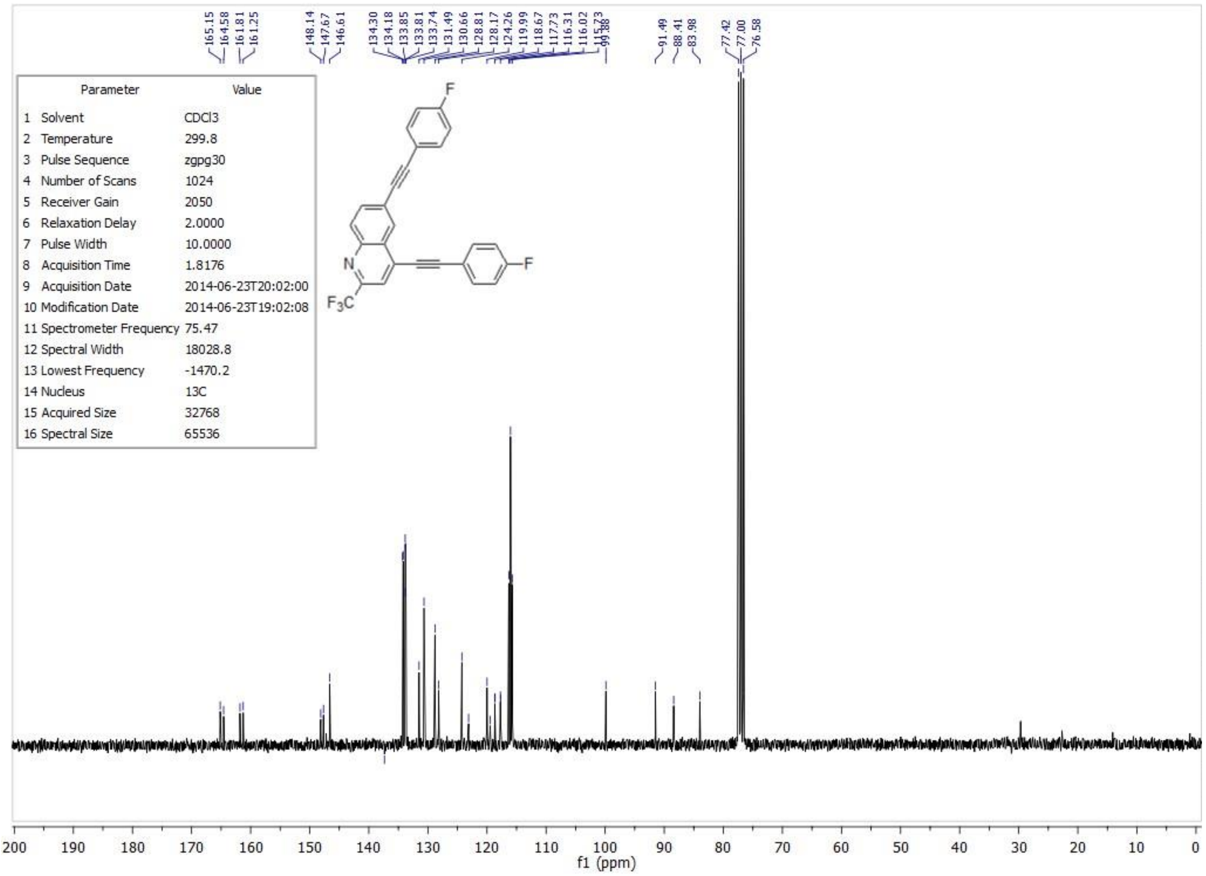
165.15
164.58
161.81
161.25
148.14
147.67
146.61
134.30
134.18
133.85
133.81
133.74
131.49
130.66
128.81
128.17
124.26
119.99
118.67
117.73
116.31
116.02
91.49
88.41
83.98
77.42
77.00
76.58
Parameter Value
1 Solvent CDCl3
2 Temperature 299.8
3 Pulse Sequence zgpg30
4 Number of Scans 1024
5 Receiver Gain 2050
6 Relaxation Delay 2.0000
7 Pulse Width 10.0000
8 Acquisition Time 1.8176
9 Acquisition Date 2014-06-23T20:02:00
10 Modification Date 2014-06-23T19:02:08
11 Spectrometer Frequency 75.47
12 Spectral Width 18028.8
13 Lowest Frequency -1470.2
14 Nucleus 13C
15 Acquired Size 32768
16 Spectral Size 65536
F
N
F
F3C
f1 (ppm)

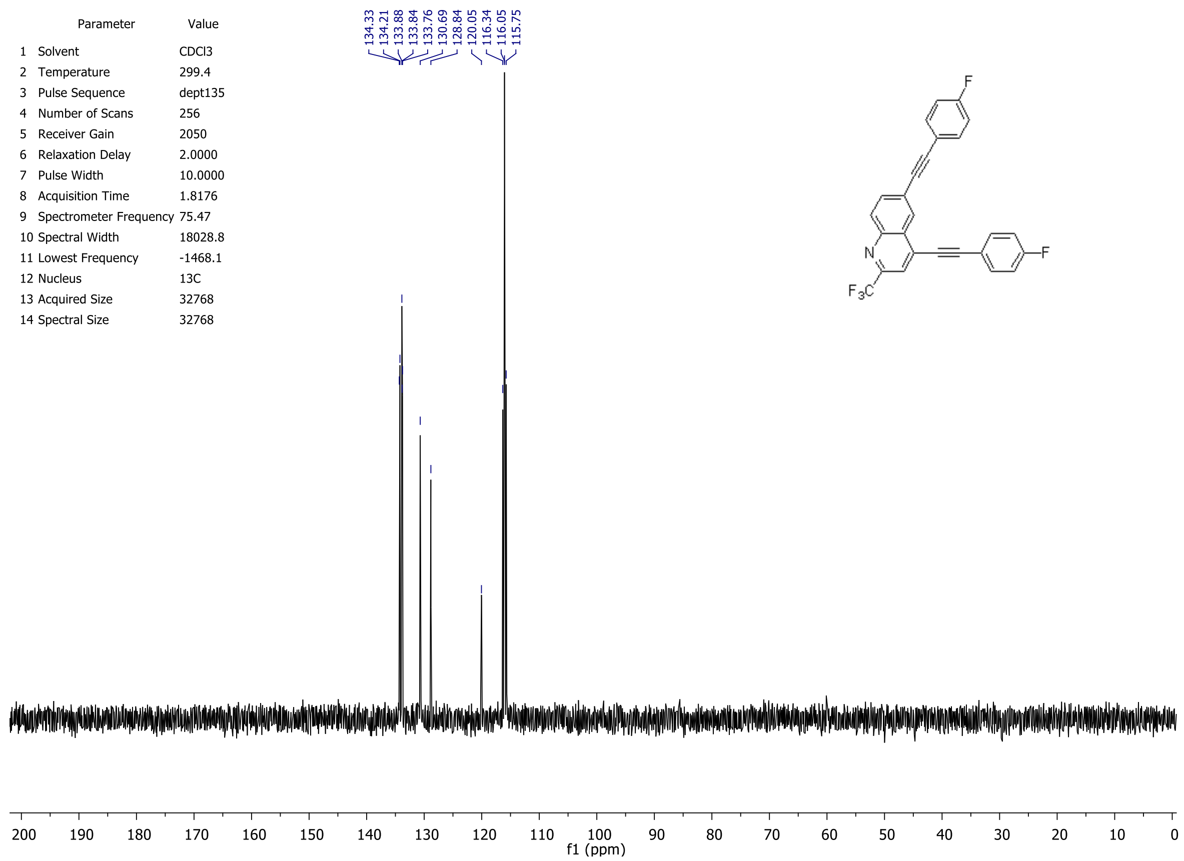
Parameter Value
1 Solvent CDCl3
2 Temperature 299.4
3 Pulse Sequence dept135
4 Number of Scans 256
5 Receiver Gain 2050
6 Relaxation Delay 2.0000
7 Pulse Width 10.0000
8 Acquisition Time 1.8176
9 Spectrometer Frequency 75.47
10 Spectral Width 18028.8
11 Lowest Frequency -1468.1
12 Nucleus 13C
13 Acquired Size 32768
14 Spectral Size 32768
134.33
134.21
133.88
133.84
133.76
130.69
128.84
120.05
116.34
116.05
115.75
F
N
F
F3C
f1 (ppm)

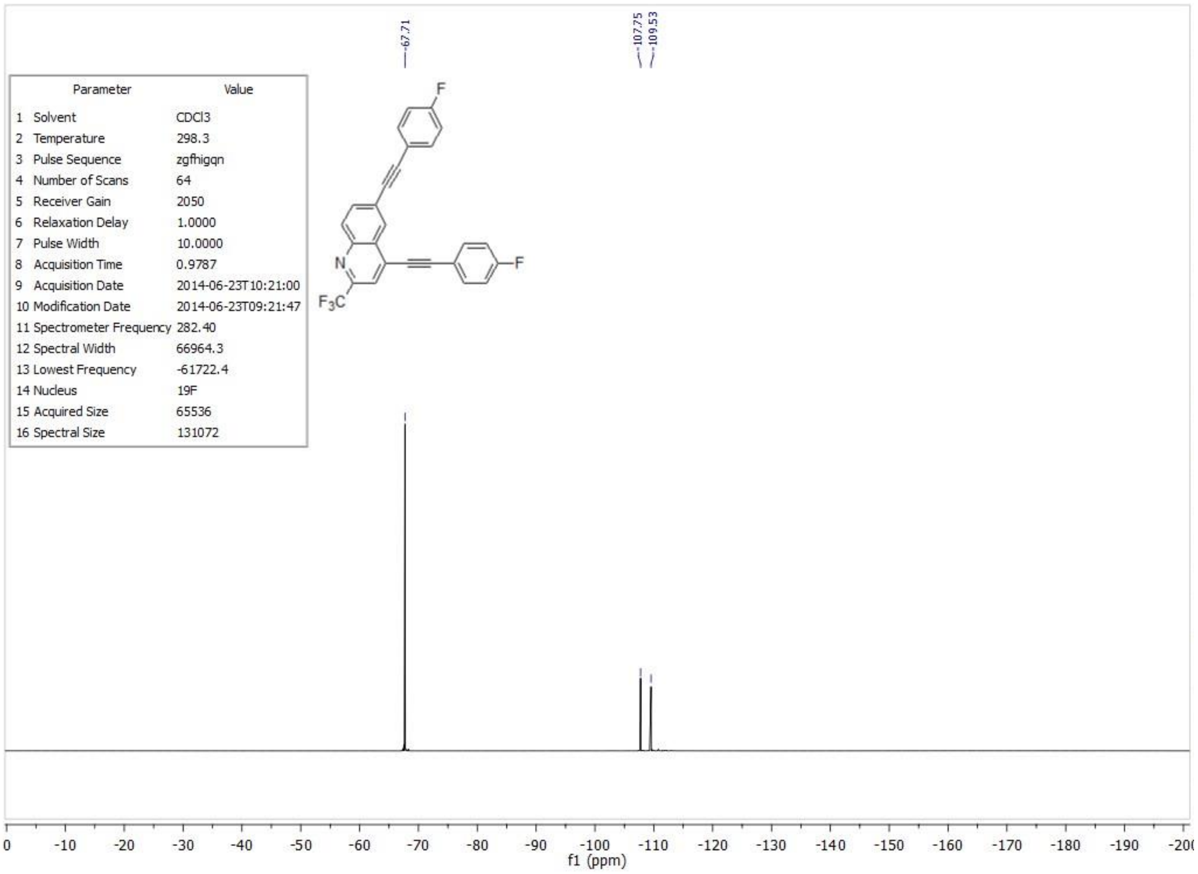

9e - 4,6-Bis(4'-methoxyphenylethynyl)-2-trifluoromethylquinoline

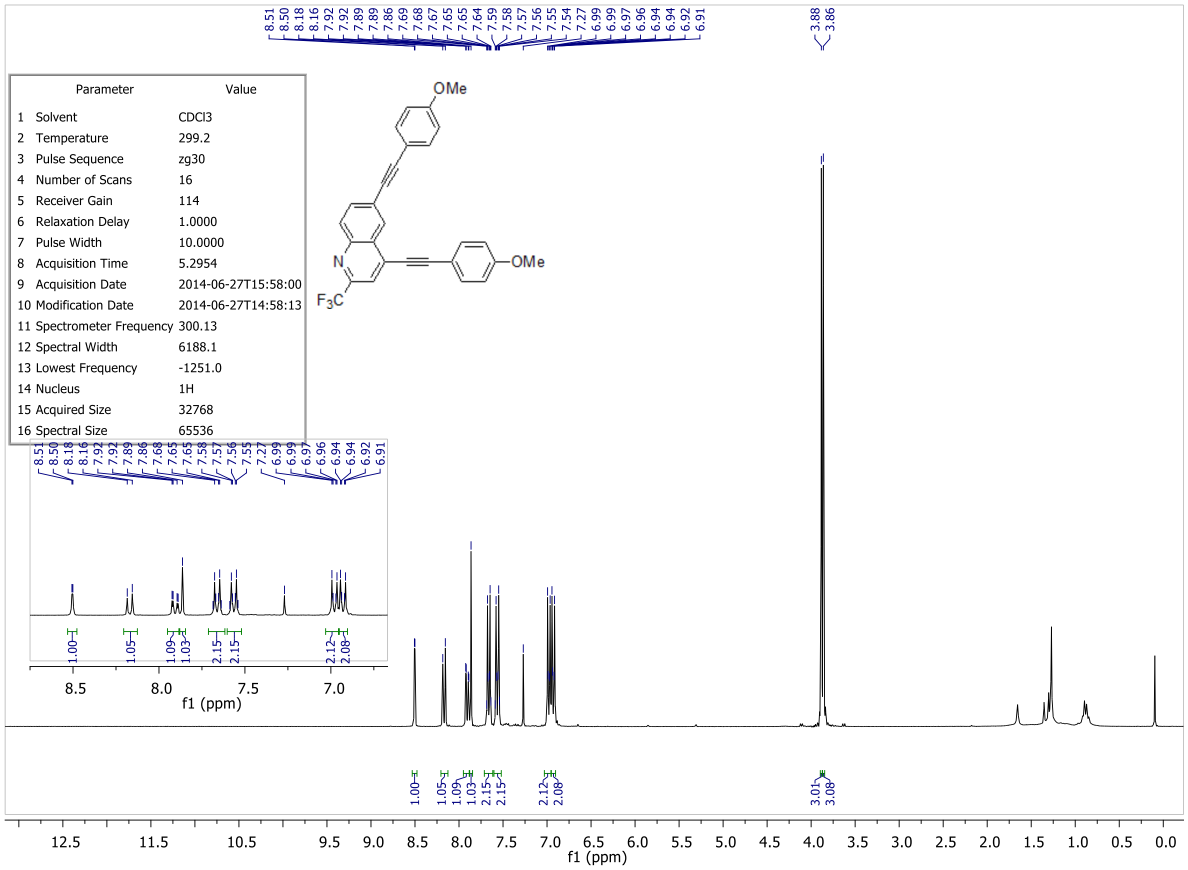

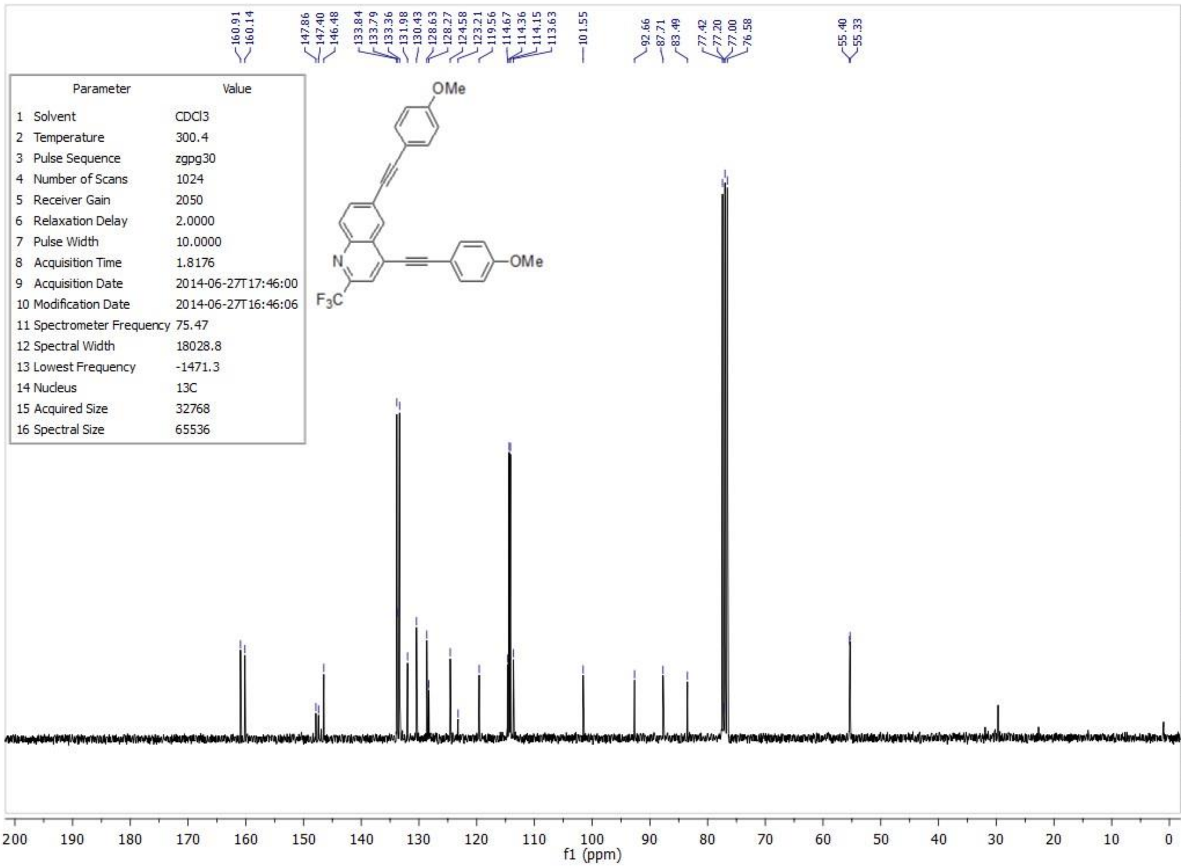

	Parameter	Value
1	Solvent	CDCl3
2	Temperature	300.4
3	Pulse Sequence	zgpg30
4	Number of Scans	1024
5	Receiver Gain	2050
6	Relaxation Delay	2.0000
7	Pulse Width	10.0000
8	Acquisition Time	1.8176
9	Acquisition Date	2014-06-27T17:46:00
10	Modification Date	2014-06-27T16:46:06
11	Spectrometer Frequency	75.47
12	Spectral Width	18028.8
13	Lowest Frequency	-1471.3
14	Nucleus	13C
15	Acquired Size	32768
16	Spectral Size	65536

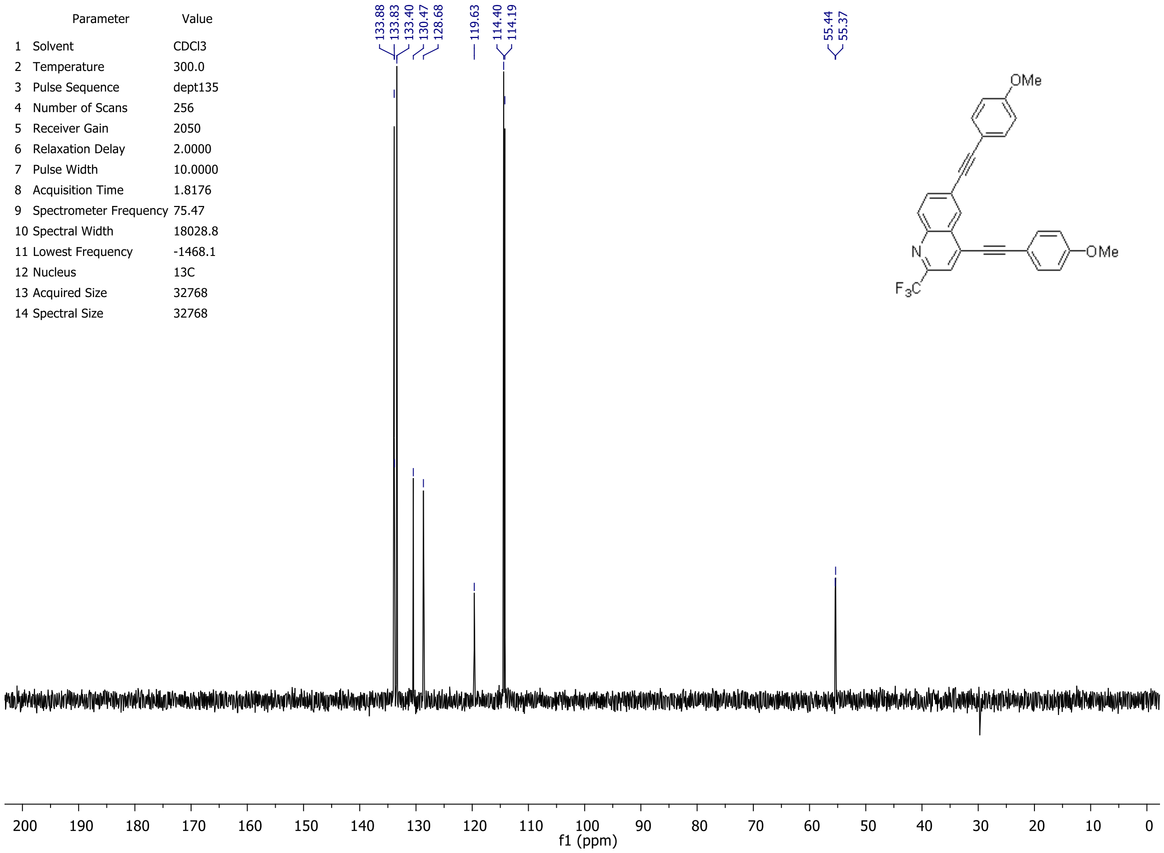

	Parameter	Value
1	Solvent	CDCl3
2	Temperature	300.0
3	Pulse Sequence	dept135
4	Number of Scans	256
5	Receiver Gain	2050
6	Relaxation Delay	2.0000
7	Pulse Width	10.0000
8	Acquisition Time	1.8176
9	Spectrometer Frequency	75.47
10	Spectral Width	18028.8
11	Lowest Frequency	-1468.1
12	Nucleus	13C
13	Acquired Size	32768
14	Spectral Size	32768

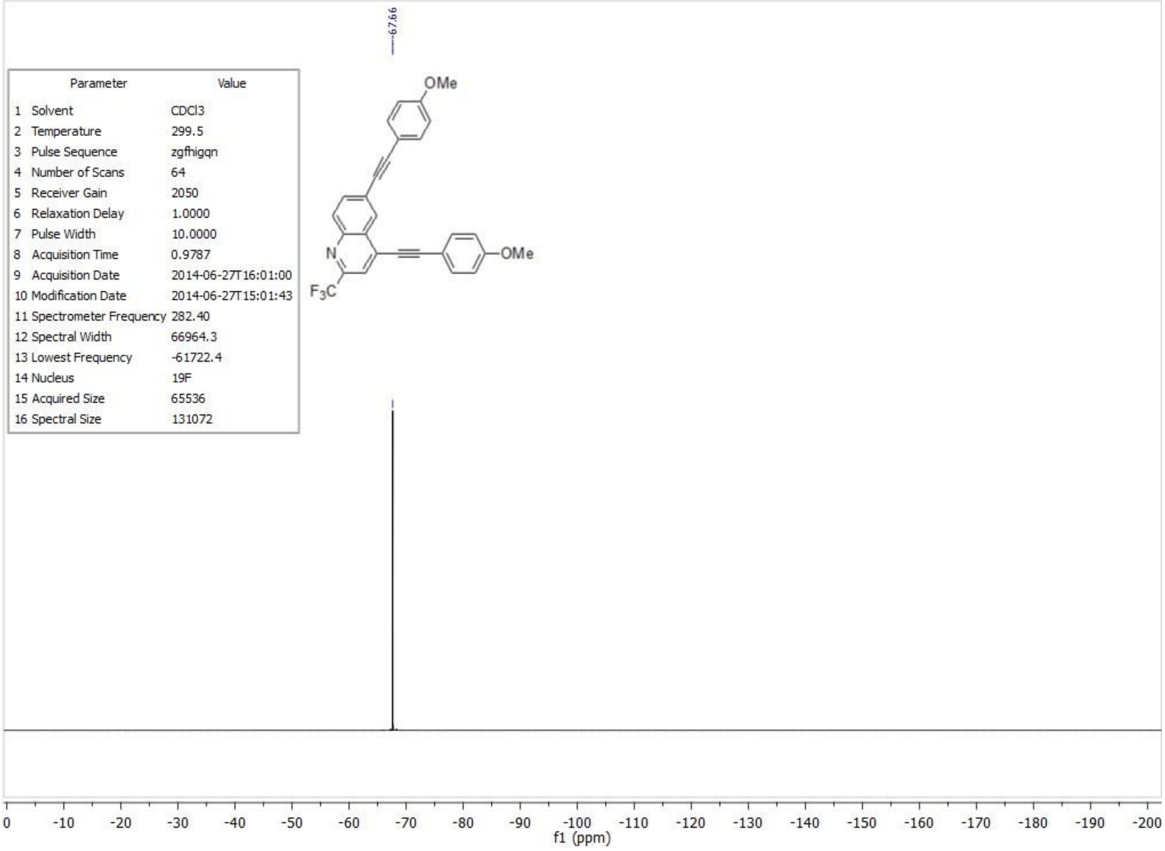

9f - 4,6-Bis(3‘-thienylethynyl)-2-trifluoromethylquinoline

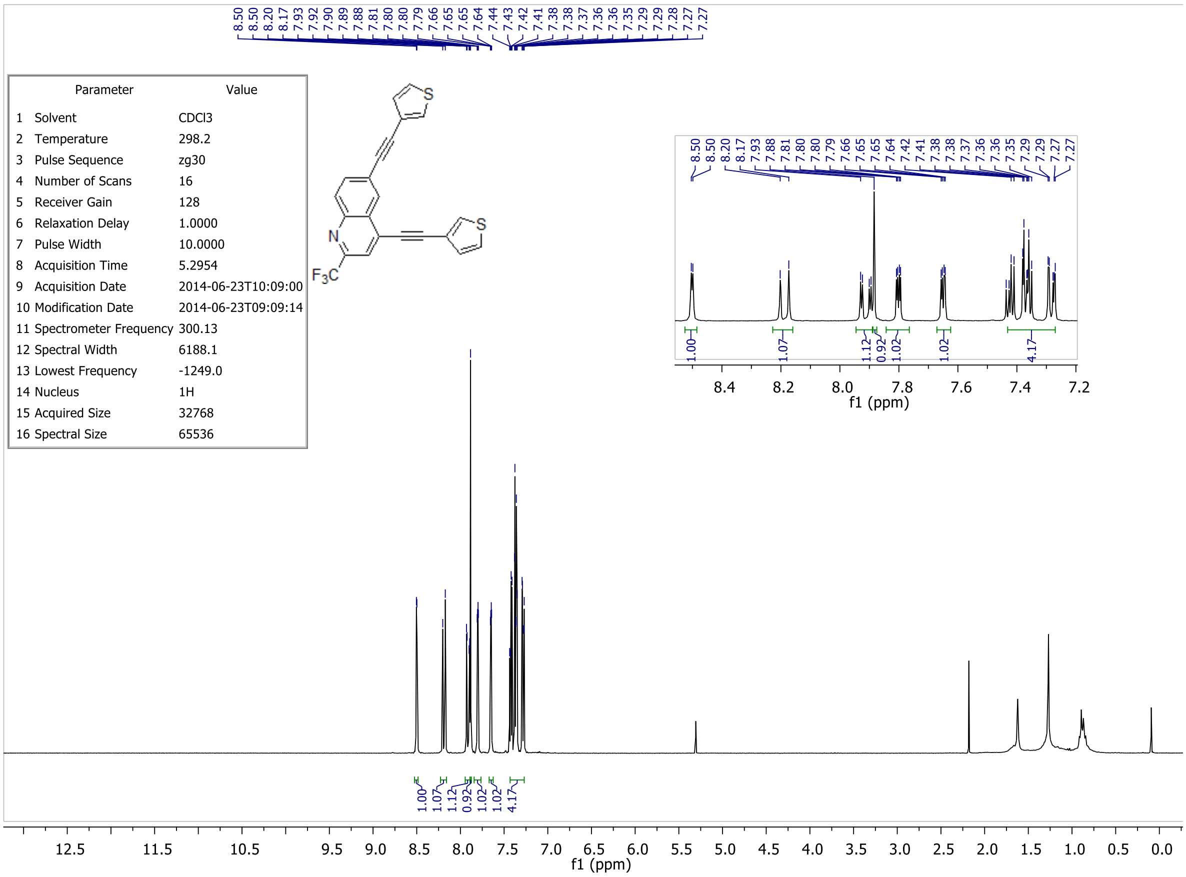

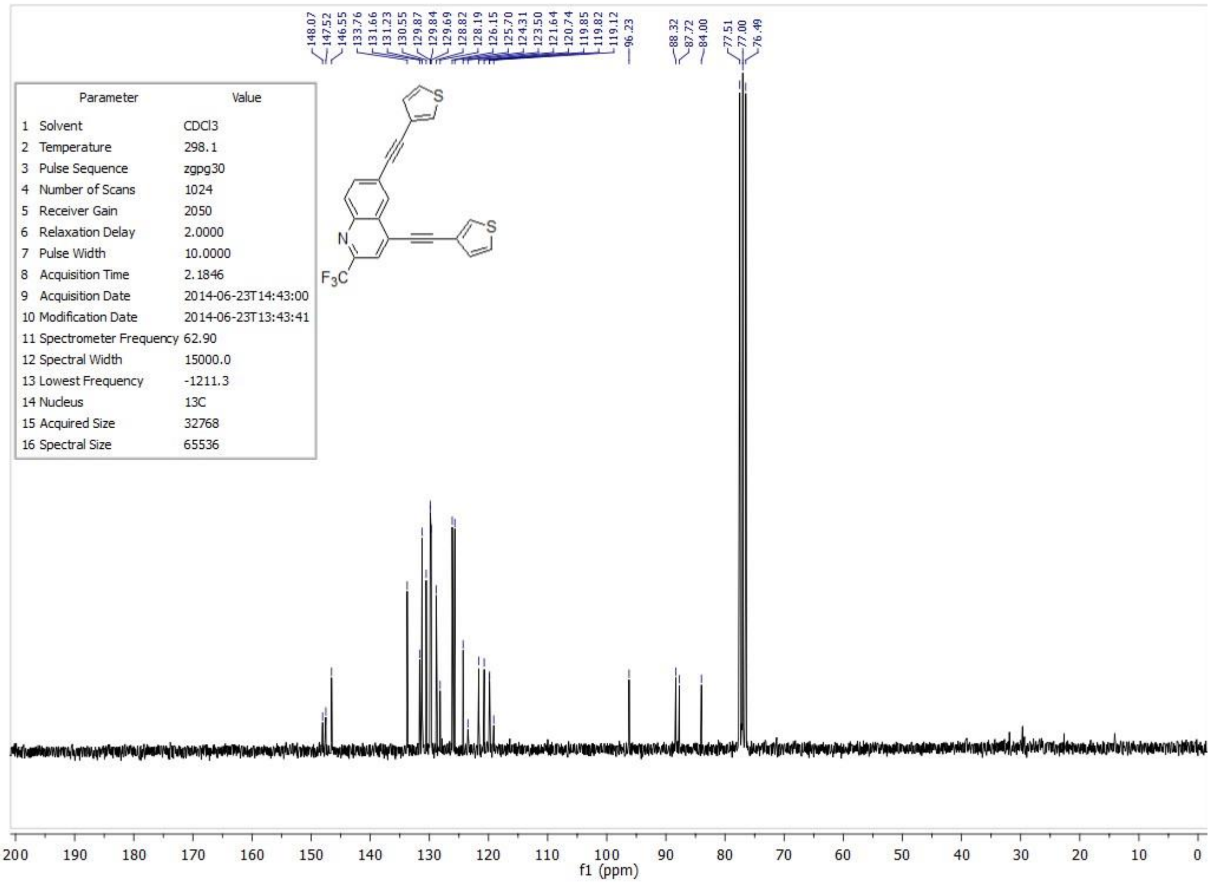
148.07
147.52
146.55
133.76
131.66
131.23
130.55
129.87
129.84
129.69
128.82
128.19
126.15
125.70
124.31
123.50
121.64
120.74
119.85
119.82
119.12
96.23
88.32
87.72
84.00
77.51
77.00
76.49
Parameter Value
1 Solvent CDCl3
2 Temperature 298.1
3 Pulse Sequence zgpg30
4 Number of Scans 1024
5 Receiver Gain 2050
6 Relaxation Delay 2.0000
7 Pulse Width 10.0000
8 Acquisition Time 2.1846
9 Acquisition Date 2014-06-23T14:43:00
10 Modification Date 2014-06-23T13:43:41
11 Spectrometer Frequency 62.90
12 Spectral Width 15000.0
13 Lowest Frequency -1211.3
14 Nucleus 13C
15 Acquired Size 32768
16 Spectral Size 65536
S
N
S
F3C
200 190 180 170 160 150 140 130 120 110 100 90 80 70 60 50 40 30 20 10 0
f1 (ppm)

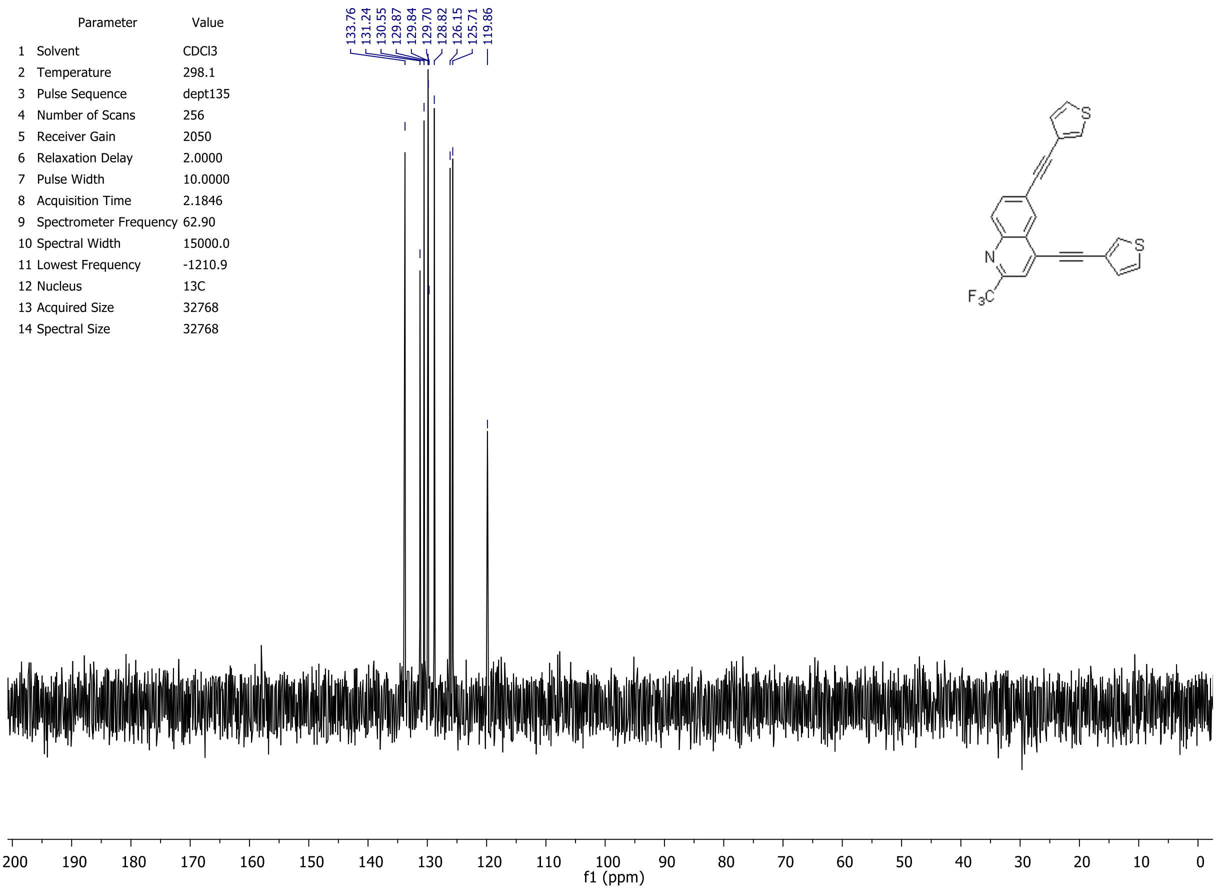
133.76
131.24
130.55
129.87
129.84
129.70
128.82
126.15
125.71
119.86
Parameter Value
1 Solvent CDCl3
2 Temperature 298.1
3 Pulse Sequence dept135
4 Number of Scans 256
5 Receiver Gain 2050
6 Relaxation Delay 2.0000
7 Pulse Width 10.0000
8 Acquisition Time 2.1846
9 Spectrometer Frequency 62.90
10 Spectral Width 15000.0
11 Lowest Frequency -1210.9
12 Nucleus 13C
13 Acquired Size 32768
14 Spectral Size 32768
S
N
S
F3C
200 190 180 170 160 150 140 130 120 110 100 90 80 70 60 50 40 30 20 10 0
f1 (ppm)

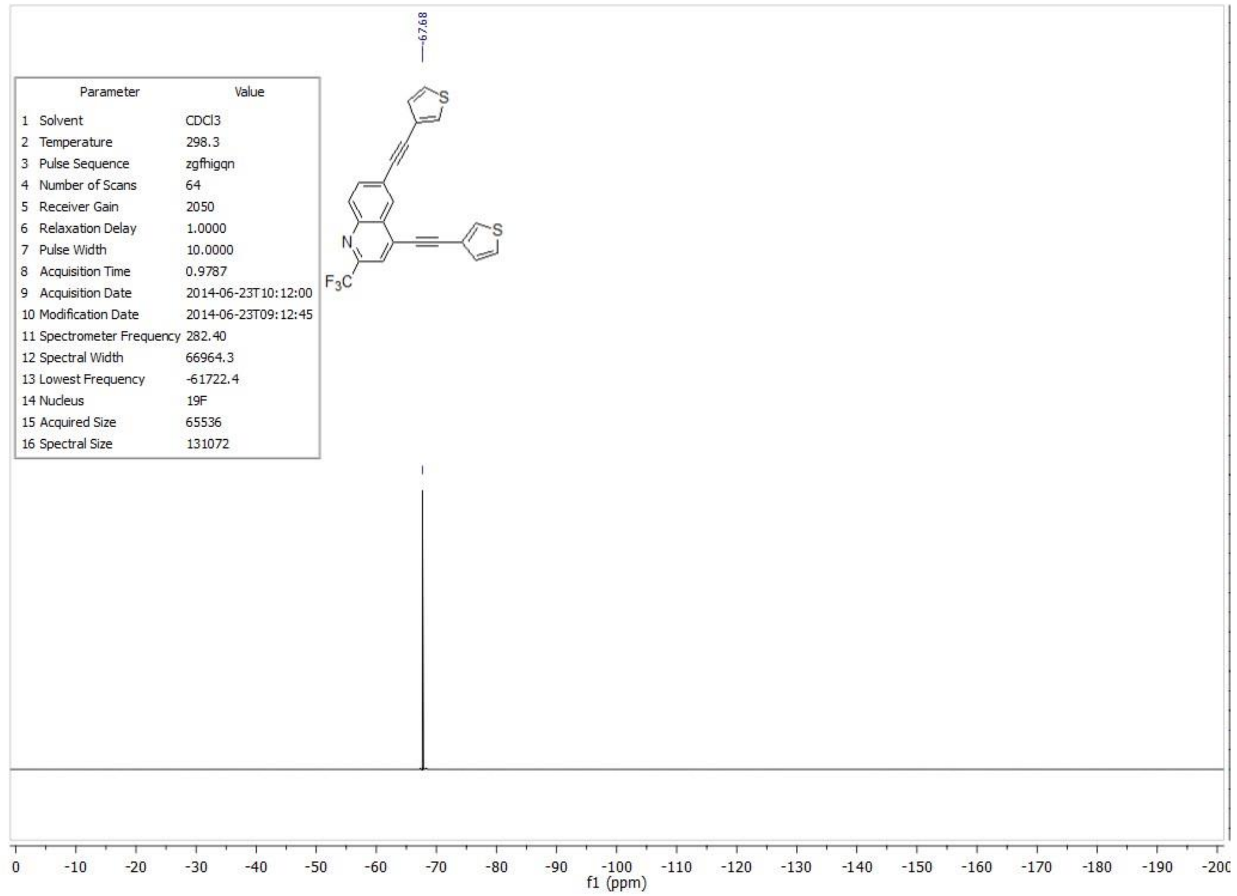

9g – 4,6-Bis(cyclopropylethynyl)-2-trifluoromethylquinoline

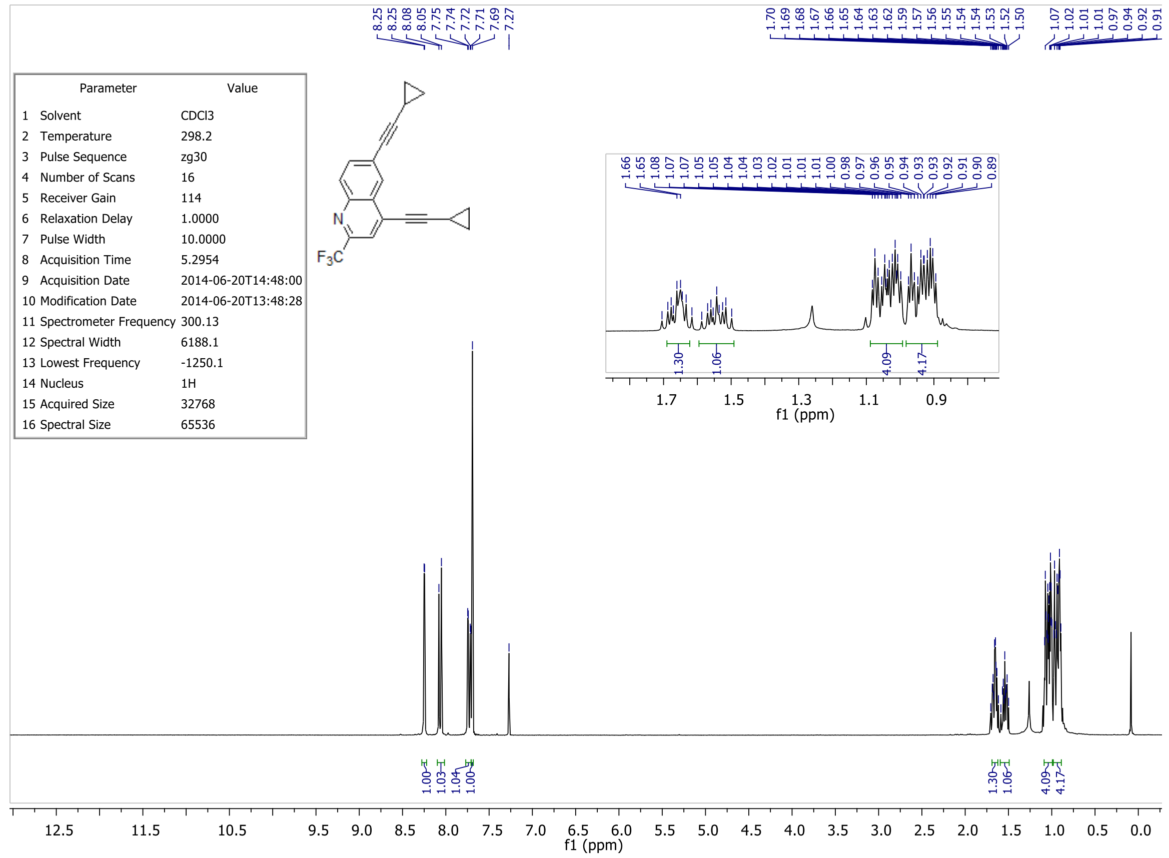

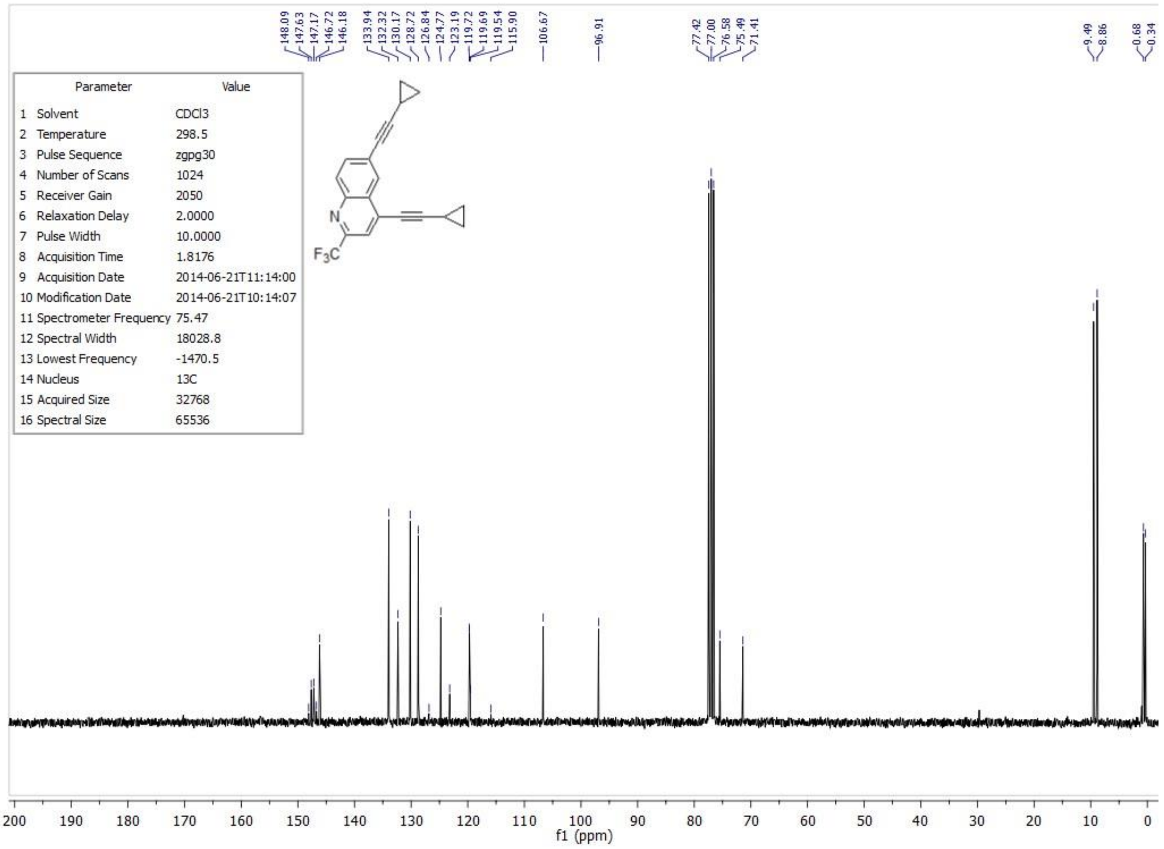

Parameter	Value
1 Solvent	CDCl3
2 Temperature	298.5
3 Pulse Sequence	zgpg30
4 Number of Scans	1024
5 Receiver Gain	2050
6 Relaxation Delay	2.0000
7 Pulse Width	10.0000
8 Acquisition Time	1.8176
9 Acquisition Date	2014-06-21T11:14:00
10 Modification Date	2014-06-21T10:14:07
11 Spectrometer Frequency	75.47
12 Spectral Width	18028.8
13 Lowest Frequency	-1470.5
14 Nucleus	13C
15 Acquired Size	32768
16 Spectral Size	65536

Parameter	Value
1 Solvent	CDCl3
2 Temperature	298.2
3 Pulse Sequence	dept135
4 Number of Scans	256
5 Receiver Gain	2050
6 Relaxation Delay	2.0000
7 Pulse Width	10.0000
8 Acquisition Time	1.8176
9 Spectrometer Frequency	75.47
10 Spectral Width	18028.8
11 Lowest Frequency	-1522.0
12 Nucleus	13C
13 Acquired Size	32768
14 Spectral Size	32768

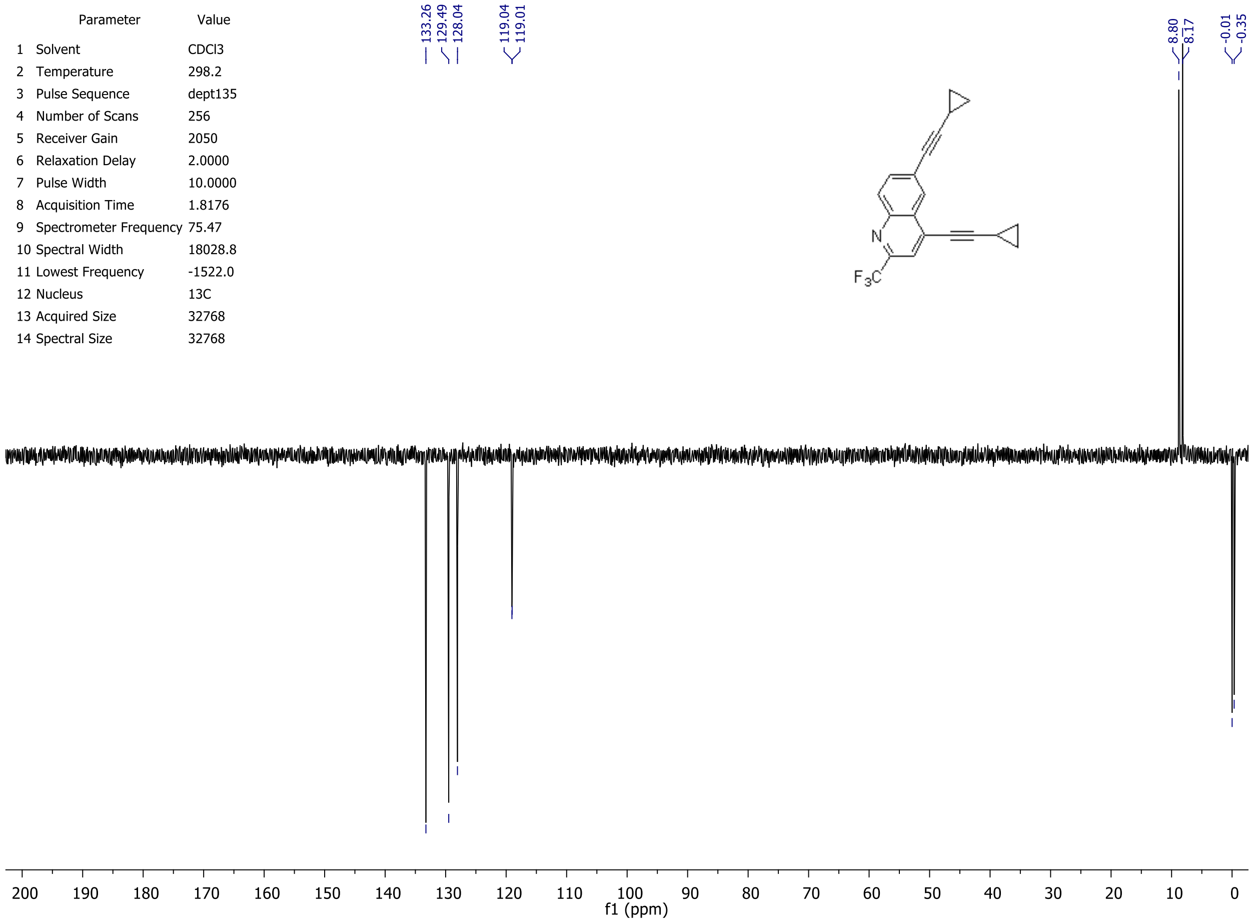

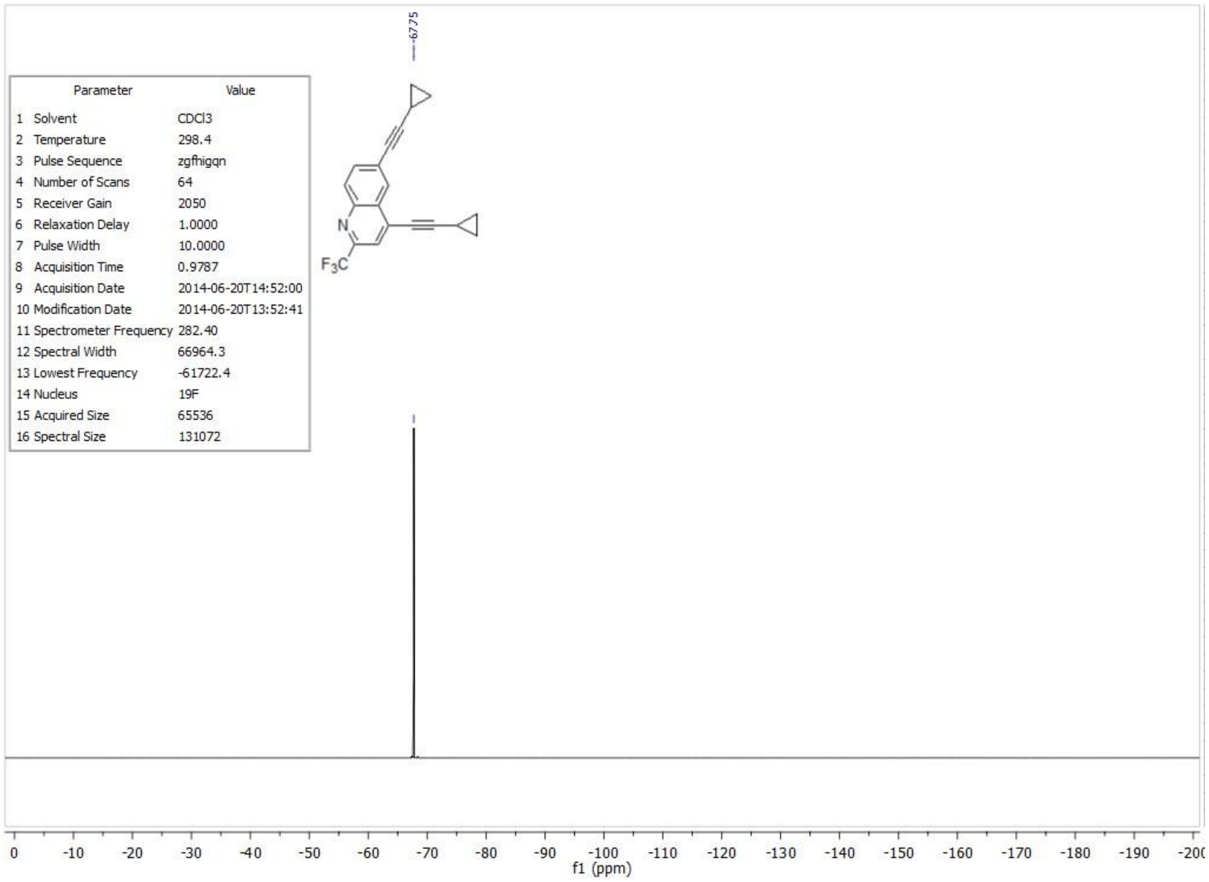
-67.75
Parameter Value
1 Solvent CDCl3
2 Temperature 298.4
3 Pulse Sequence zgfhigqn
4 Number of Scans 64
5 Receiver Gain 2050
6 Relaxation Delay 1.0000
7 Pulse Width 10.0000
8 Acquisition Time 0.9787
9 Acquisition Date 2014-06-20T14:52:00
10 Modification Date 2014-06-20T13:52:41
11 Spectrometer Frequency 282.40
12 Spectral Width 66964.3
13 Lowest Frequency -61722.4
14 Nucleus 19F
15 Acquired Size 65536
16 Spectral Size 131072
N
F3C
0 -10 -20 -30 -40 -50 -60 -70 -80 -90 -100 -110 -120 -130 -140 -150 -160 -170 -180 -190 -200
f1 (ppm)

12a - 3,4,8-Tris(phenylethynyl)-2-trifluoromethylquinoline

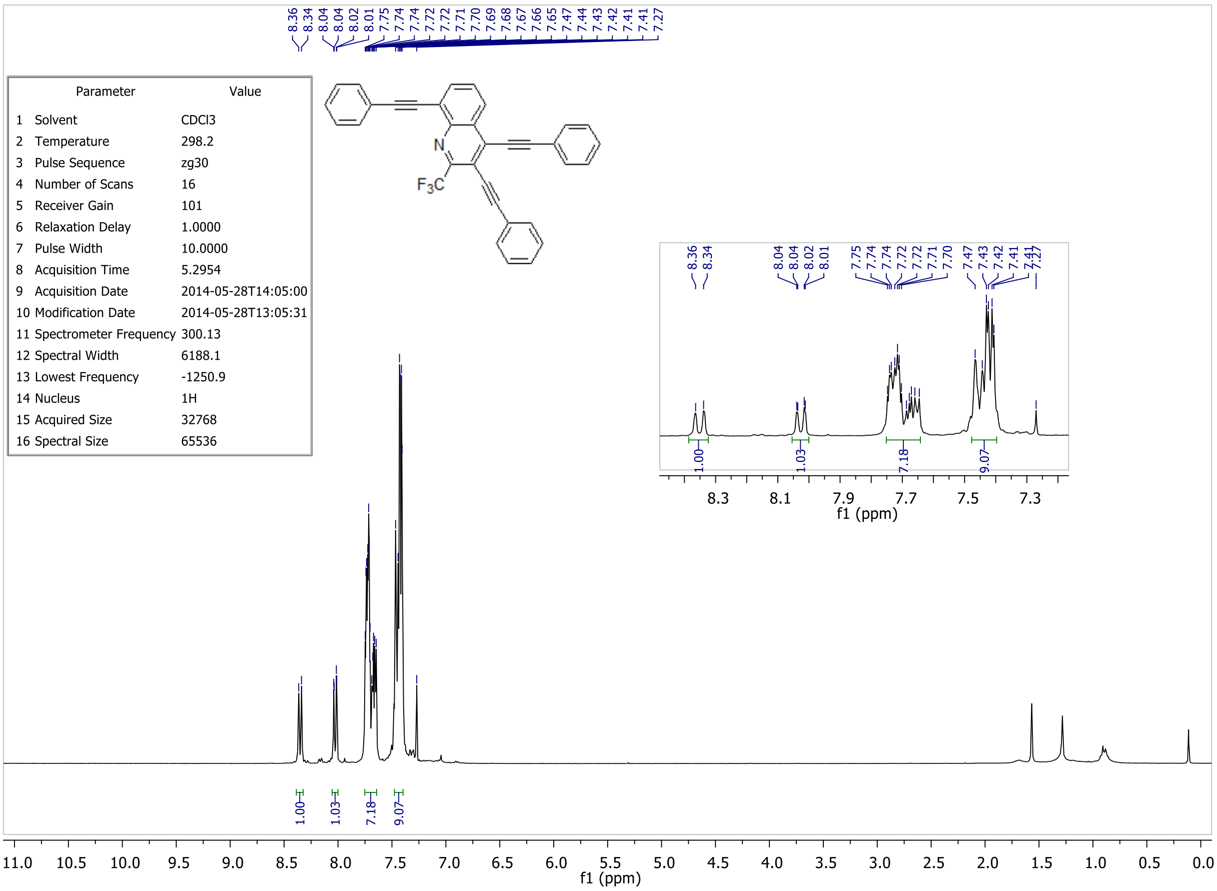

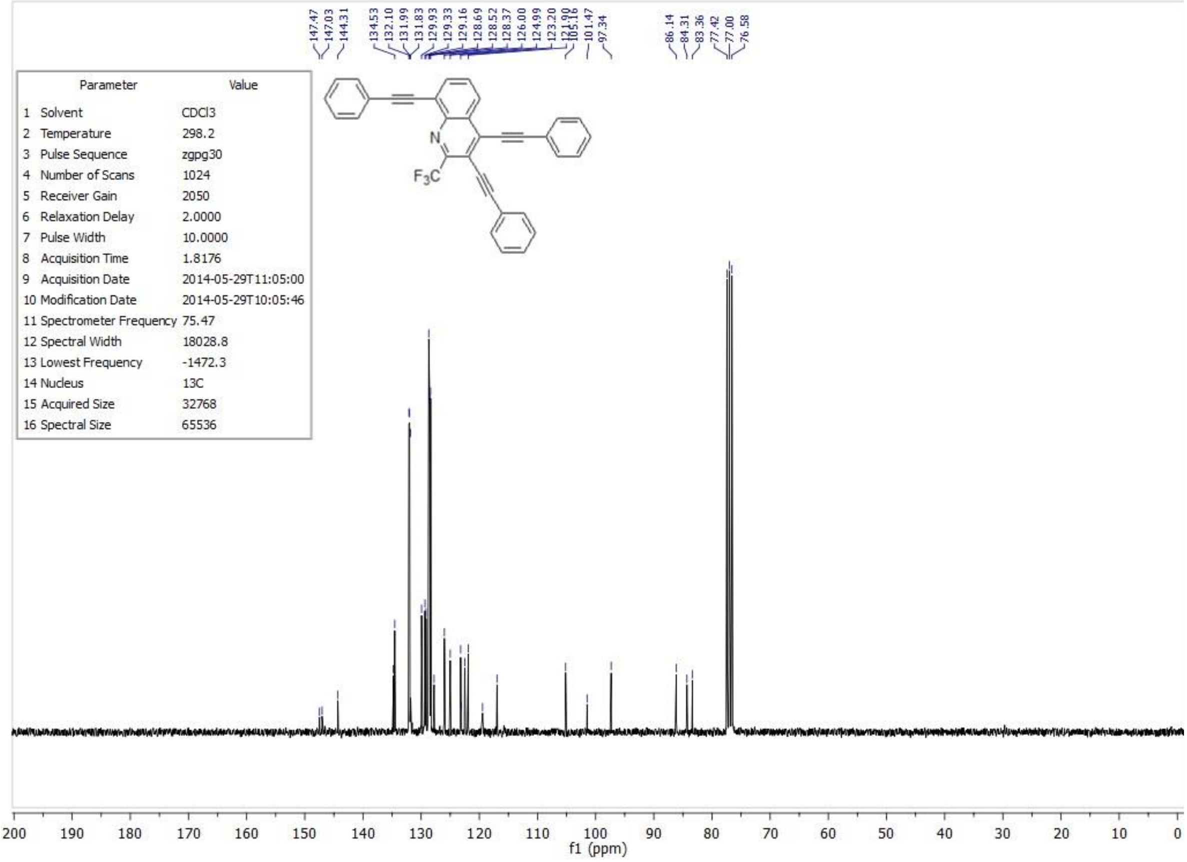

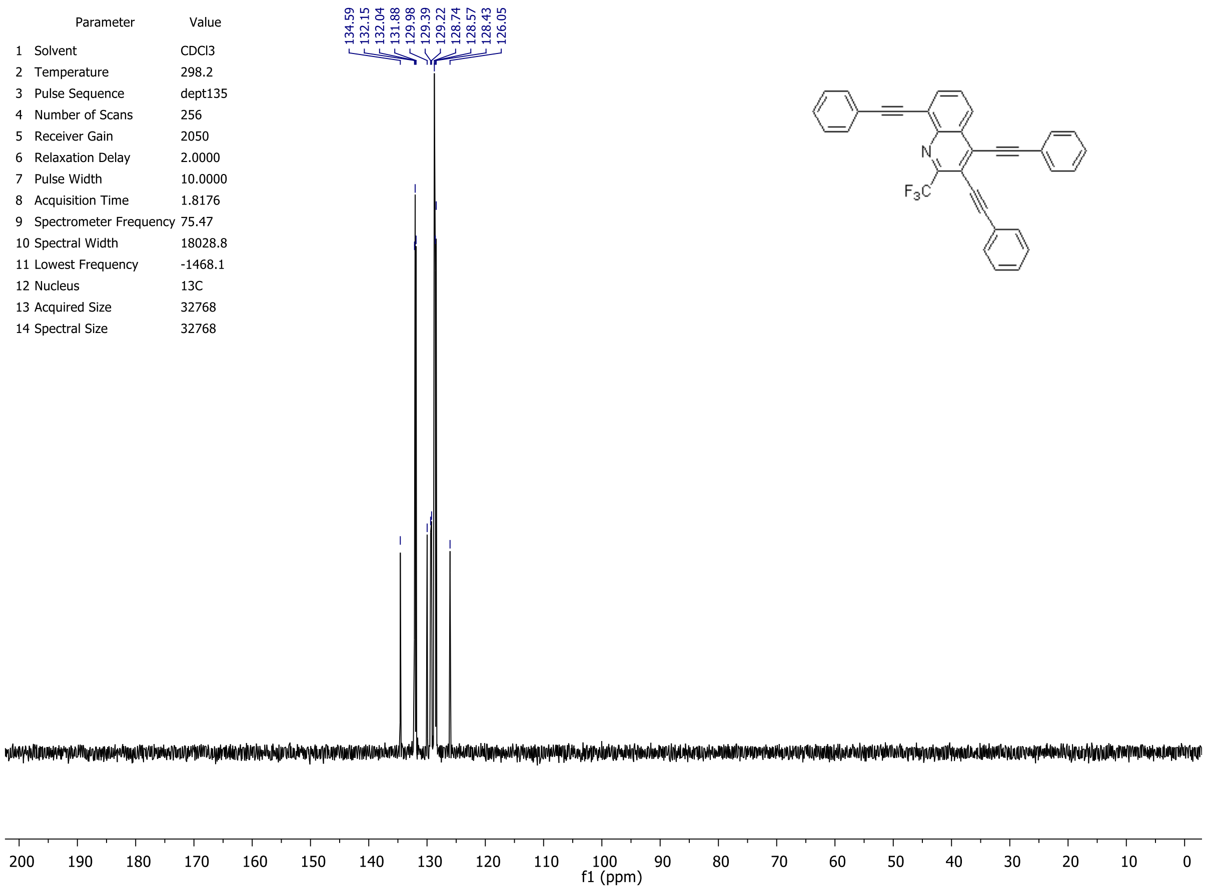

	Parameter	Value
1	Solvent	CDCl3
2	Temperature	298.2
3	Pulse Sequence	dept135
4	Number of Scans	256
5	Receiver Gain	2050
6	Relaxation Delay	2.0000
7	Pulse Width	10.0000
8	Acquisition Time	1.8176
9	Spectrometer Frequency	75.47
10	Spectral Width	18028.8
11	Lowest Frequency	-1468.1
12	Nucleus	13C
13	Acquired Size	32768
14	Spectral Size	32768

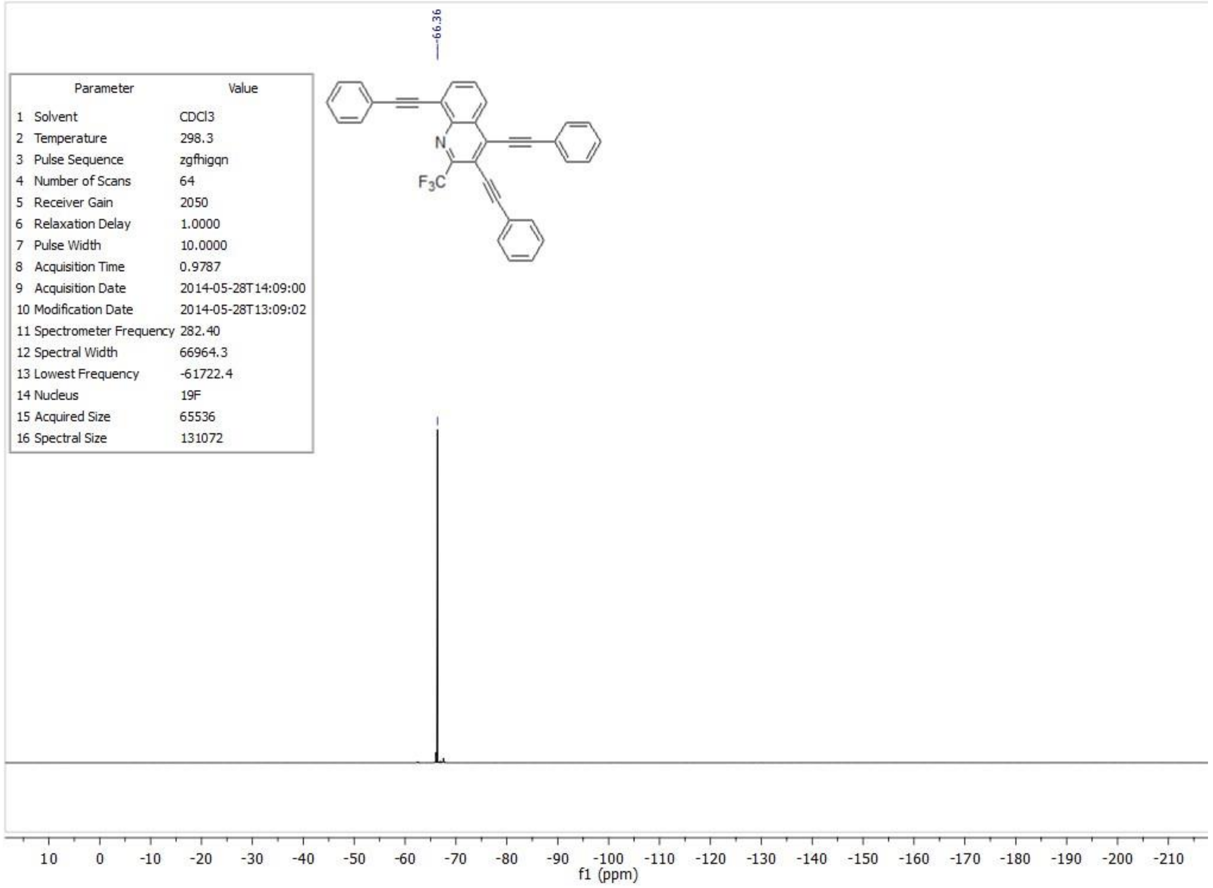

	Parameter	Value
1	Solvent	CDCl3
2	Temperature	298.3
3	Pulse Sequence	zgfhigqn
4	Number of Scans	64
5	Receiver Gain	2050
6	Relaxation Delay	1.0000
7	Pulse Width	10.0000
8	Acquisition Time	0.9787
9	Acquisition Date	2014-05-28T14:09:00
10	Modification Date	2014-05-28T13:09:02
11	Spectrometer Frequency	282.40
12	Spectral Width	66964.3
13	Lowest Frequency	-61722.4
14	Nucleus	19F
15	Acquired Size	65536
16	Spectral Size	131072

12b - 3,4,8-Tris(4′-tert-butylphenylethynyl)-2-trifluoromethylquinoline

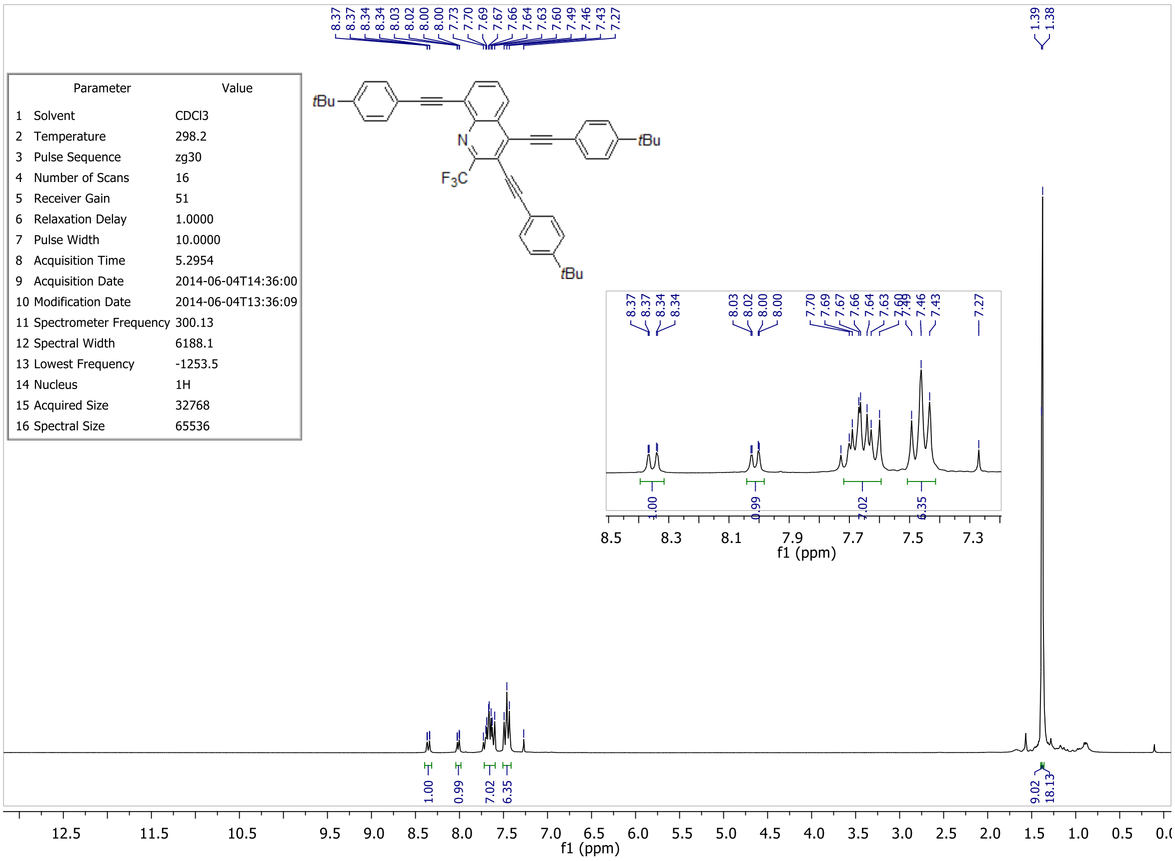

	Parameter	Value
1	Solvent	CDCl3
2	Temperature	298.2
3	Pulse Sequence	zg30
4	Number of Scans	16
5	Receiver Gain	51
6	Relaxation Delay	1.0000
7	Pulse Width	10.0000
8	Acquisition Time	5.2954
9	Acquisition Date	2014-06-04T14:36:00
10	Modification Date	2014-06-04T13:36:09
11	Spectrometer Frequency	300.13
12	Spectral Width	6188.1
13	Lowest Frequency	-1253.5
14	Nucleus	1H
15	Acquired Size	32768
16	Spectral Size	65536

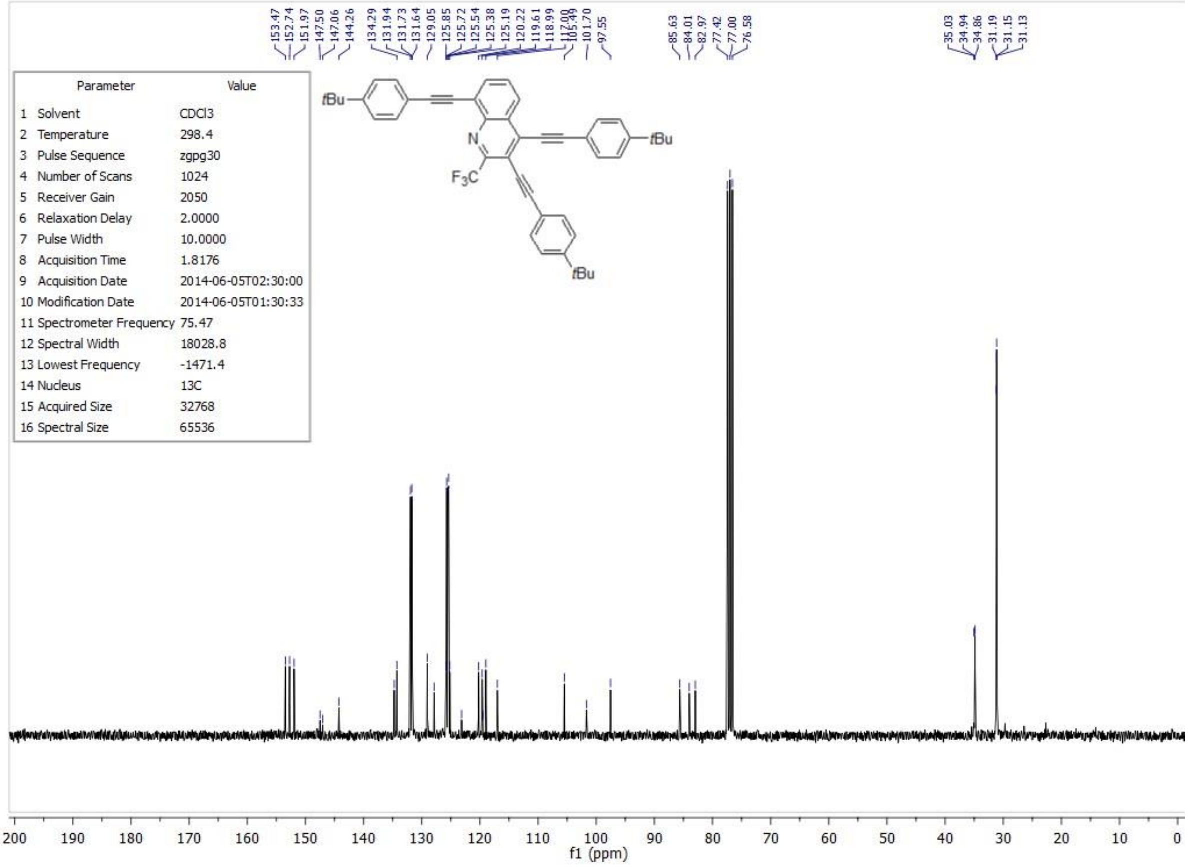

	Parameter	Value
1	Solvent	CDCl3
2	Temperature	298.4
3	Pulse Sequence	zgpg30
4	Number of Scans	1024
5	Receiver Gain	2050
6	Relaxation Delay	2.0000
7	Pulse Width	10.0000
8	Acquisition Time	1.8176
9	Acquisition Date	2014-06-05T02:30:00
10	Modification Date	2014-06-05T01:30:33
11	Spectrometer Frequency	75.47
12	Spectral Width	18028.8
13	Lowest Frequency	-1471.4
14	Nucleus	13C
15	Acquired Size	32768
16	Spectral Size	65536

Parameter	Value
1 Solvent	CDCl3
2 Temperature	298.2
3 Pulse Sequence	dept135
4 Number of Scans	256
5 Receiver Gain	2050
6 Relaxation Delay	2.0000
7 Pulse Width	10.0000
8 Acquisition Time	1.8176
9 Spectrometer Frequency	75.47
10 Spectral Width	18028.8
11 Lowest Frequency	-1468.1
12 Nucleus	13C
13 Acquired Size	32768
14 Spectral Size	32768

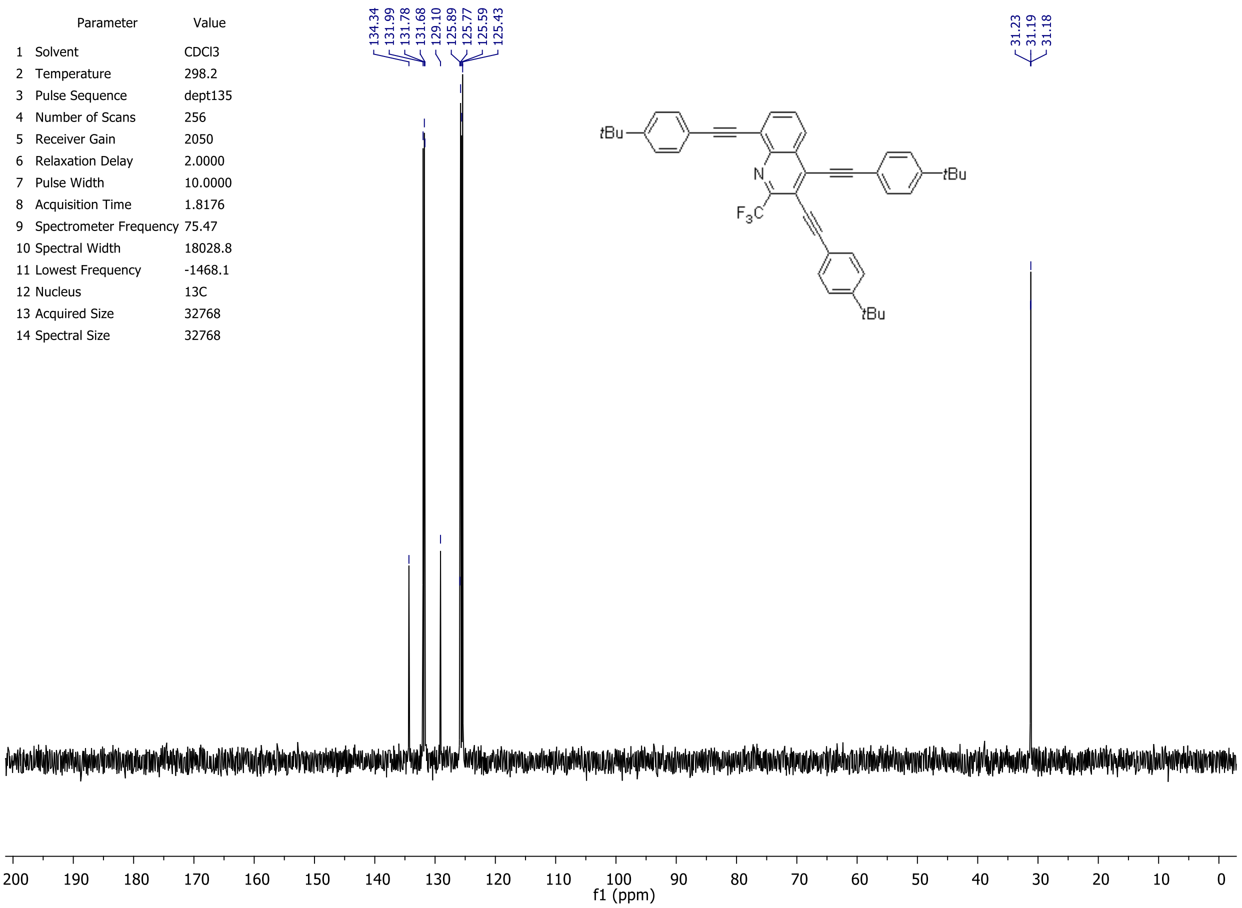

Parameter	Value
1 Solvent	CDCl3
2 Temperature	298.3
3 Pulse Sequence	zgfhigqn
4 Number of Scans	64
5 Receiver Gain	2050
6 Relaxation Delay	1.0000
7 Pulse Width	10.0000
8 Acquisition Time	0.9787
9 Acquisition Date	2014-06-04T14:40:00
10 Modification Date	2014-06-04T13:40:03
11 Spectrometer Frequency	282.40
12 Spectral Width	66964.3
13 Lowest Frequency	-61722.4
14 Nucleus	19F
15 Acquired Size	65536
16 Spectral Size	131072

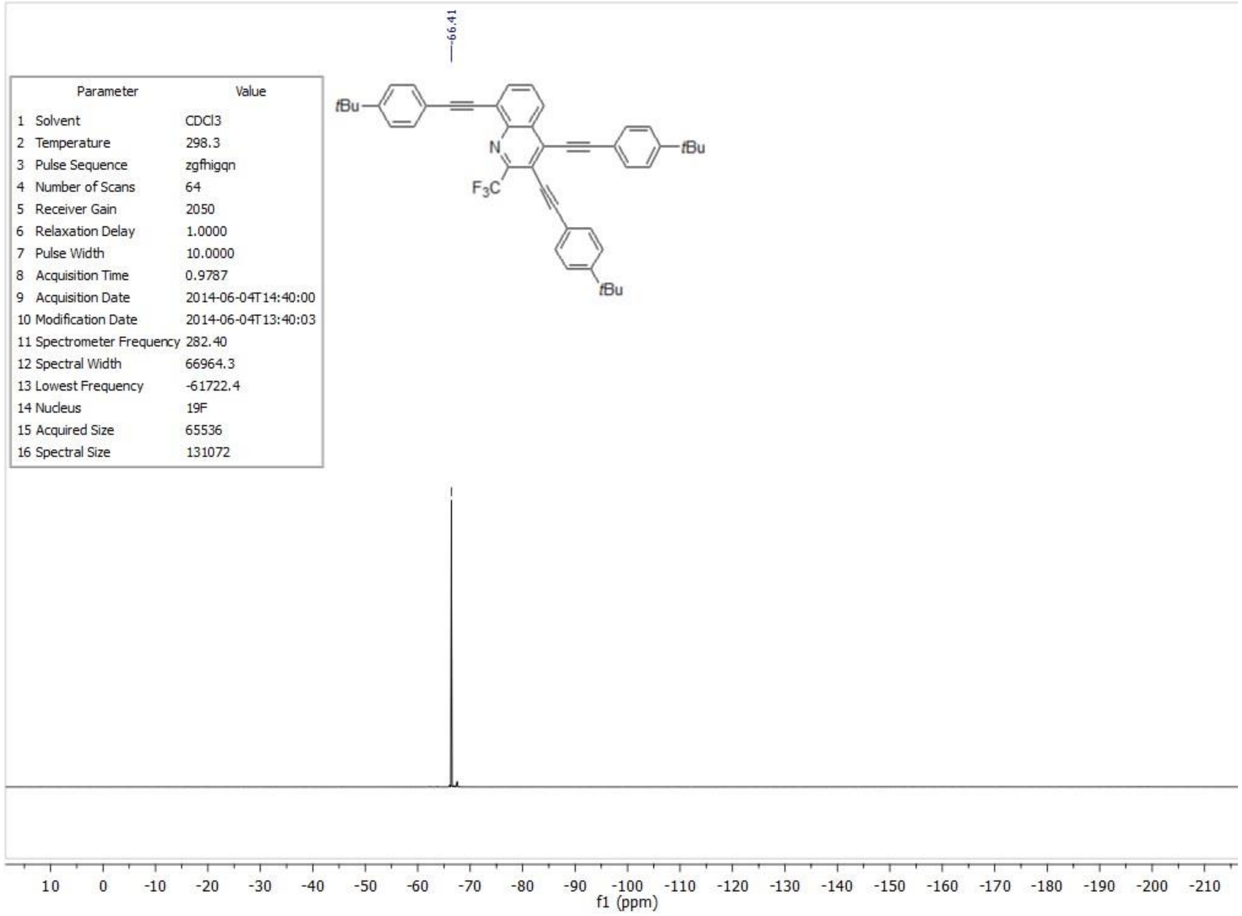

12d - 3,4,8-Tris(4′-fluorophenylethynyl)-2-trifluoromethylquinoline

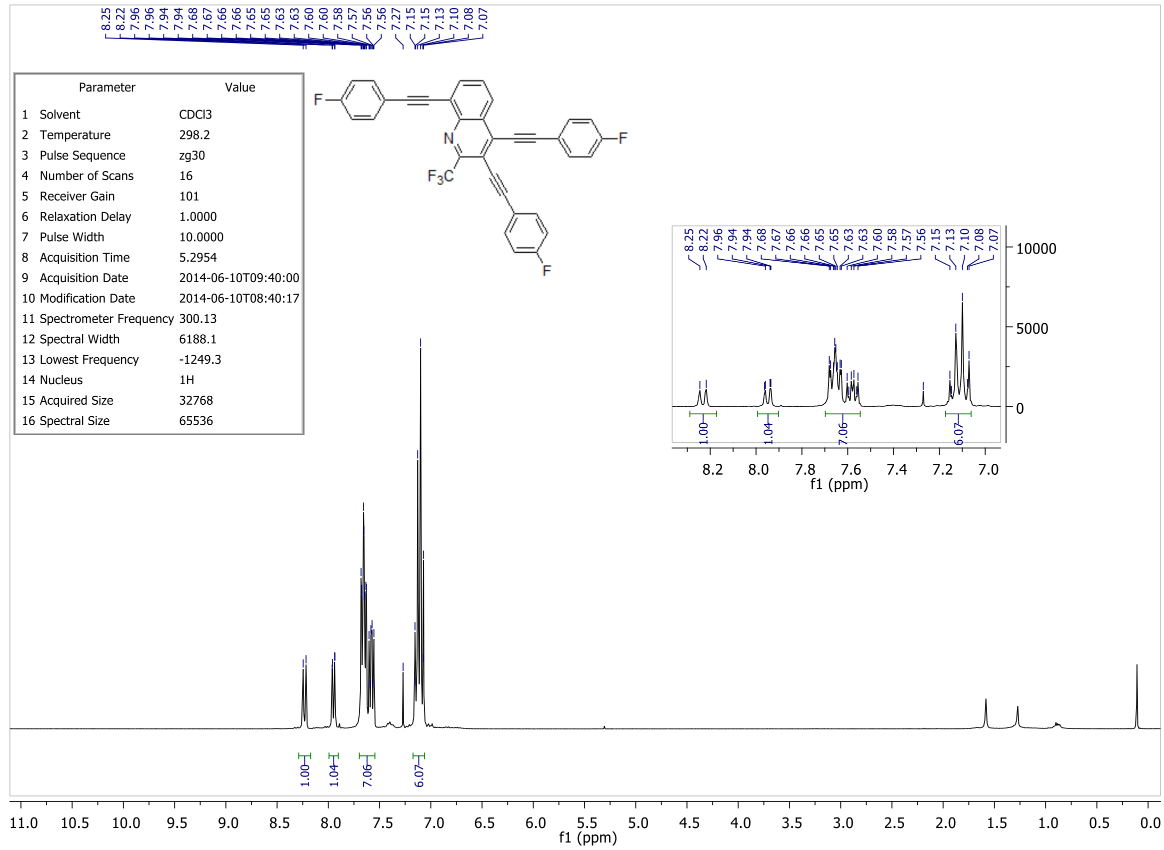

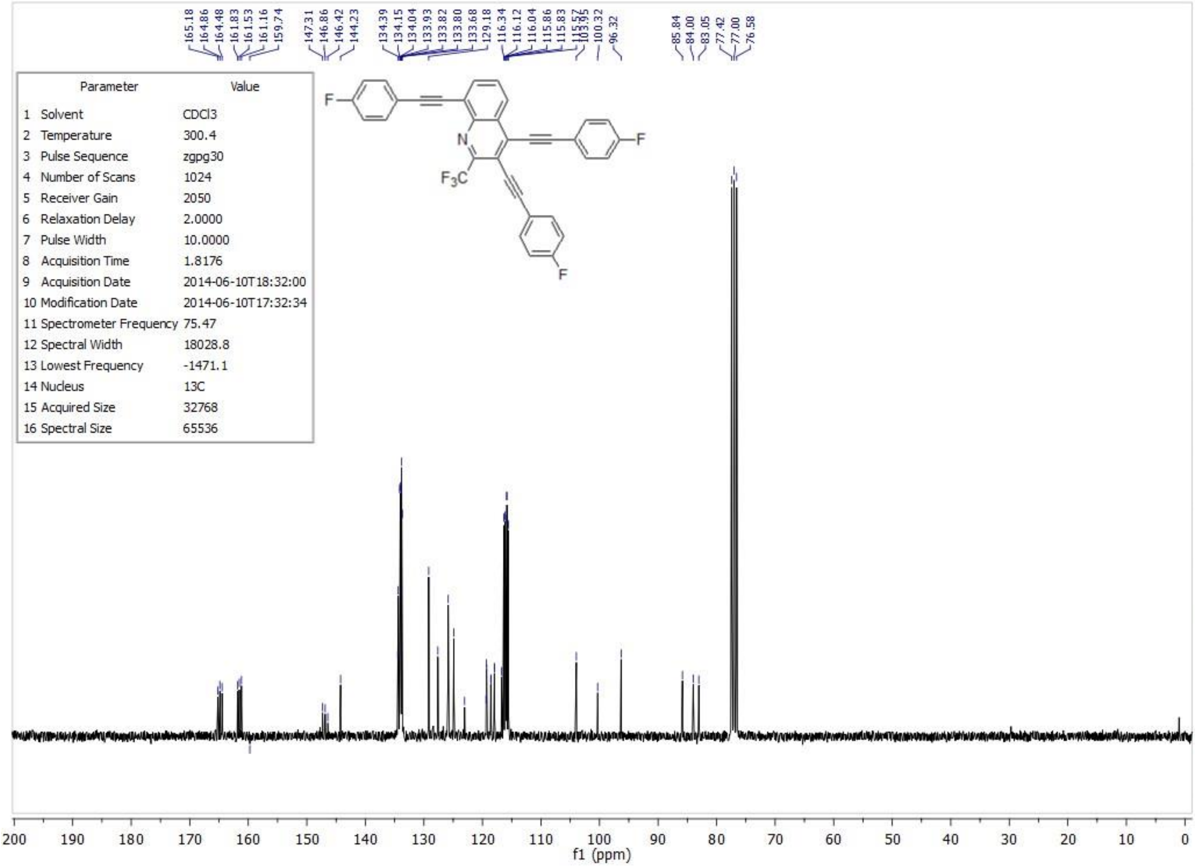

Parameter	Value
1 Solvent	CDCl3
2 Temperature	298.4
3 Pulse Sequence	dept135
4 Number of Scans	256
5 Receiver Gain	2050
6 Relaxation Delay	2.0000
7 Pulse Width	10.0000
8 Acquisition Time	1.8176
9 Spectrometer Frequency	75.47
10 Spectral Width	18028.8
11 Lowest Frequency	-1468.1
12 Nucleus	13C
13 Acquired Size	32768
14 Spectral Size	32768

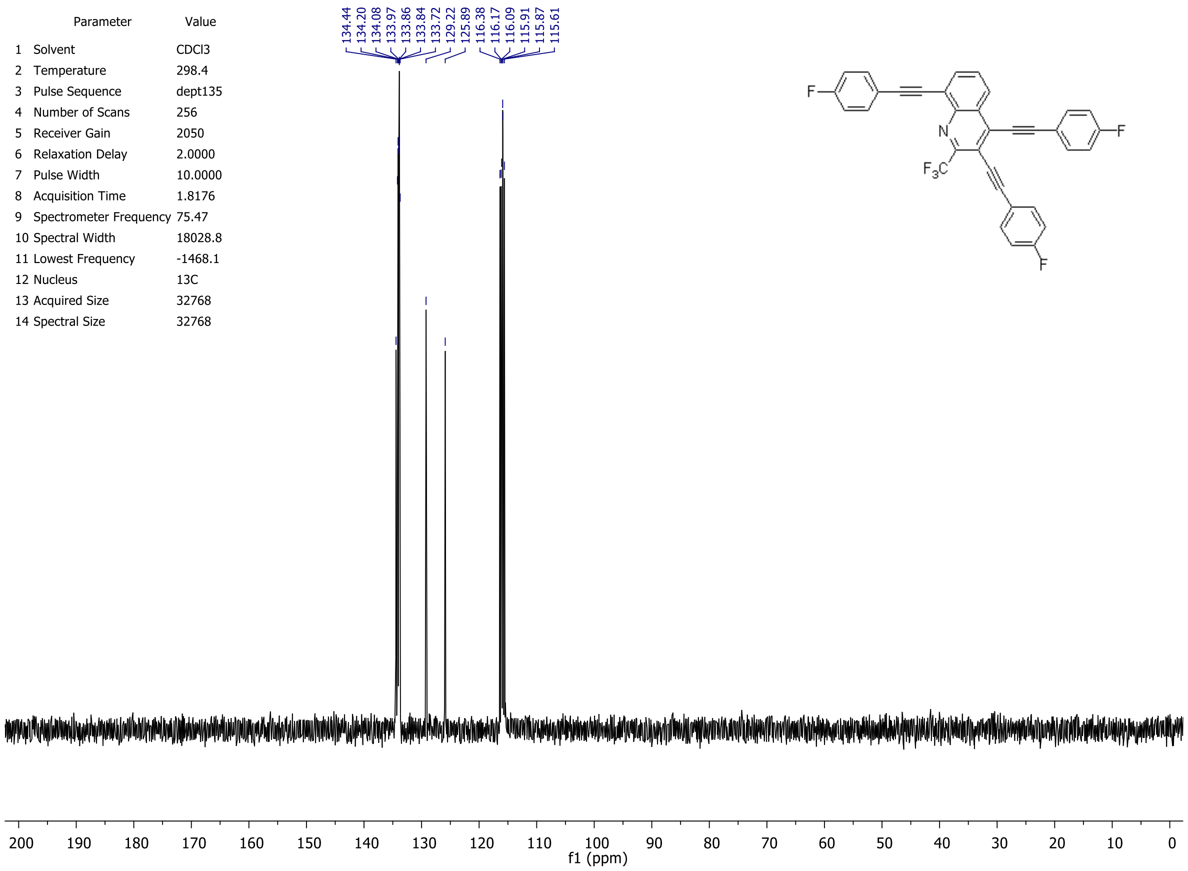

Parameter	Value
1 Solvent	CDCl3
2 Temperature	298.4
3 Pulse Sequence	zgfhigqn
4 Number of Scans	64
5 Receiver Gain	2050
6 Relaxation Delay	1.0000
7 Pulse Width	10.0000
8 Acquisition Time	0.9787
9 Acquisition Date	2014-06-10T09:43:00
10 Modification Date	2014-06-10T08:43:48
11 Spectrometer Frequency	282.40
12 Spectral Width	66964.3
13 Lowest Frequency	-61722.4
14 Nucleus	19F
15 Acquired Size	65536
16 Spectral Size	131072

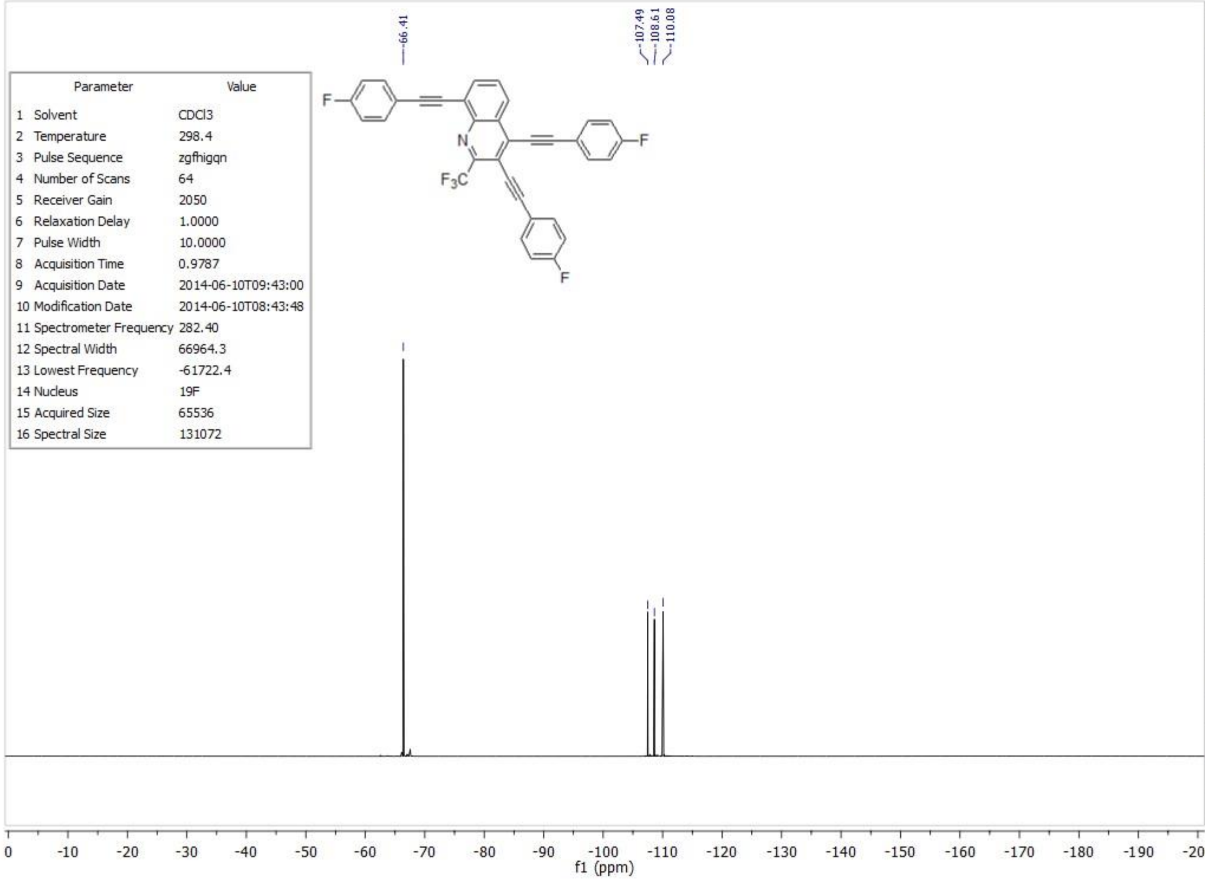

12e - 3,4,8-Tris(4′-methoxyphenylethynyl)-2-trifluoromethylquinoline

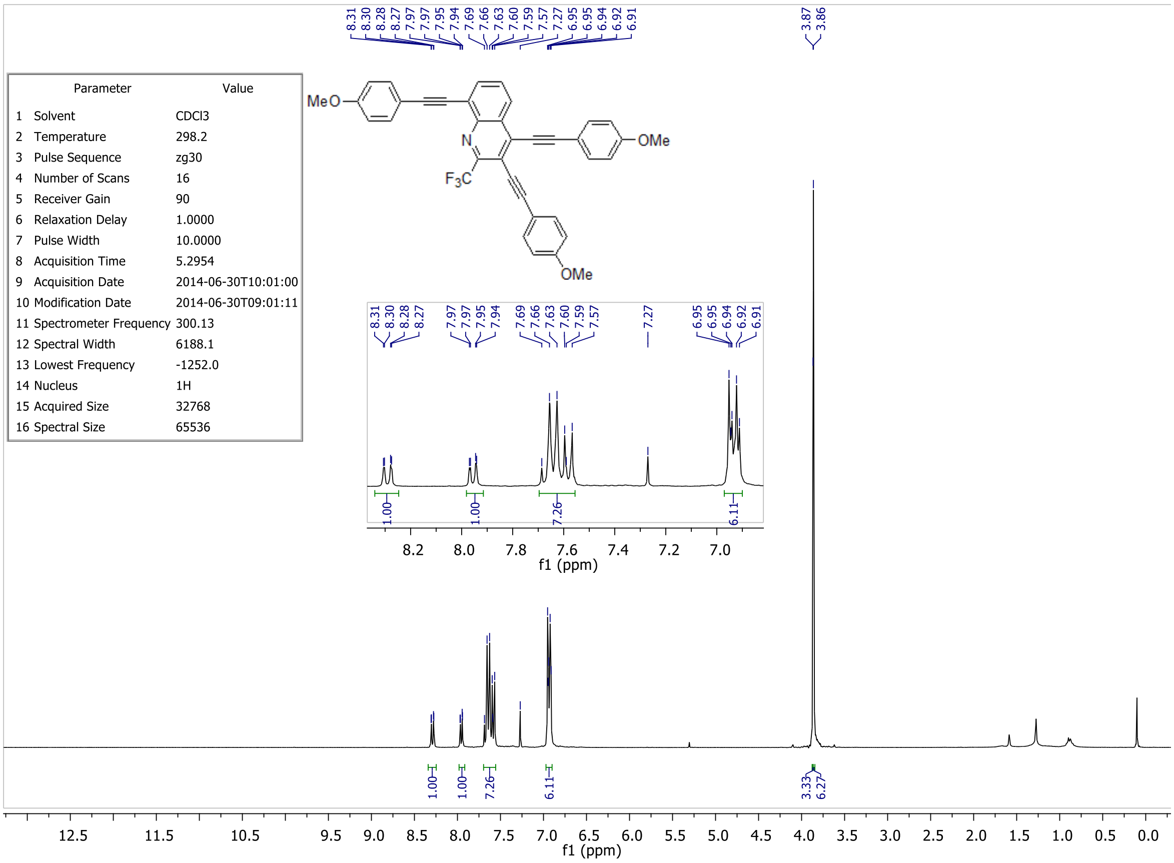

	Parameter	Value
1	Solvent	CDCl3
2	Temperature	298.2
3	Pulse Sequence	zg30
4	Number of Scans	16
5	Receiver Gain	90
6	Relaxation Delay	1.0000
7	Pulse Width	10.0000
8	Acquisition Time	5.2954
9	Acquisition Date	2014-06-30T10:01:00
10	Modification Date	2014-06-30T09:01:11
11	Spectrometer Frequency	300.13
12	Spectral Width	6188.1
13	Lowest Frequency	-1252.0
14	Nucleus	1H
15	Acquired Size	32768
16	Spectral Size	65536

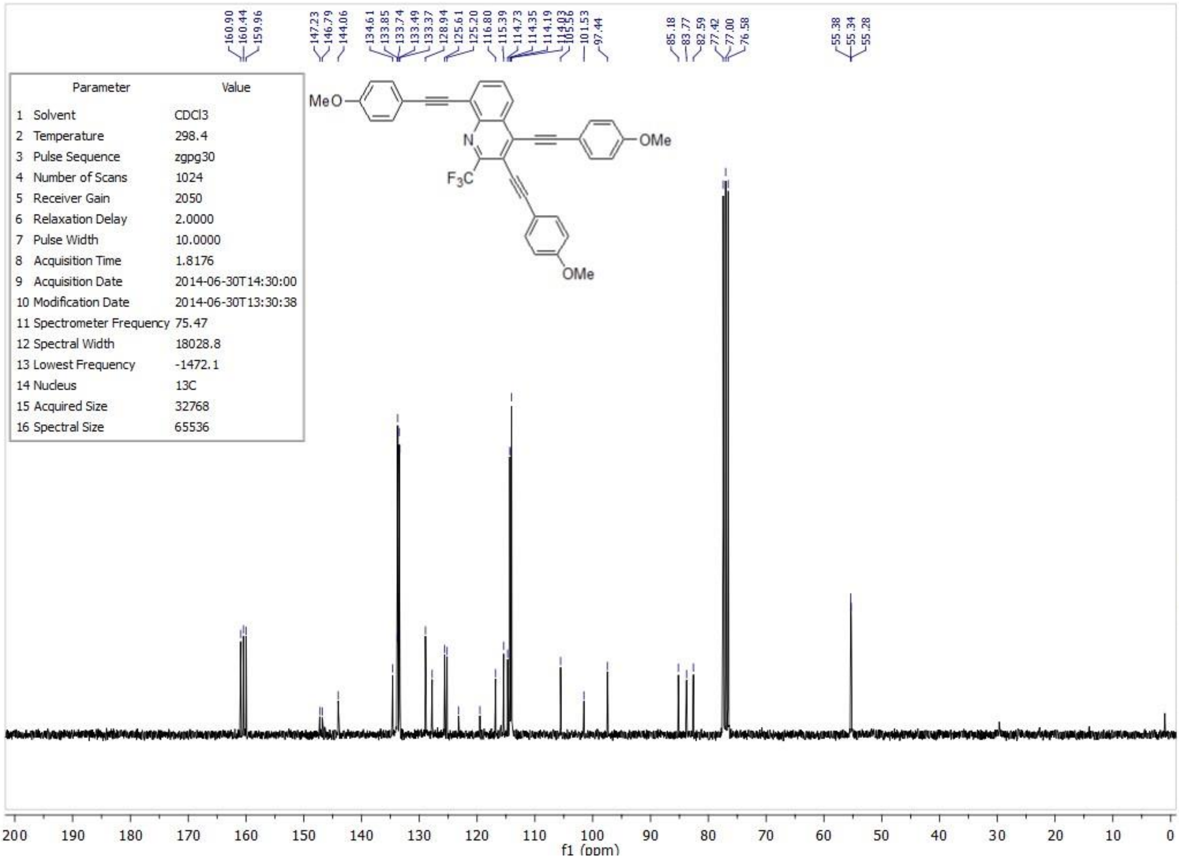

	Parameter	Value
1	Solvent	CDCl3
2	Temperature	298.4
3	Pulse Sequence	zgpg30
4	Number of Scans	1024
5	Receiver Gain	2050
6	Relaxation Delay	2.0000
7	Pulse Width	10.0000
8	Acquisition Time	1.8176
9	Acquisition Date	2014-06-30T14:30:00
10	Modification Date	2014-06-30T13:30:38
11	Spectrometer Frequency	75.47
12	Spectral Width	18028.8
13	Lowest Frequency	-1472.1
14	Nucleus	13C
15	Acquired Size	32768
16	Spectral Size	65536

	Parameter	Value
1	Solvent	CDCl3
2	Temperature	298.2
3	Pulse Sequence	dept135
4	Number of Scans	256
5	Receiver Gain	2050
6	Relaxation Delay	2.0000
7	Pulse Width	10.0000
8	Acquisition Time	1.8176
9	Spectrometer Frequency	75.47
10	Spectral Width	18028.8
11	Lowest Frequency	-1468.1
12	Nucleus	13C
13	Acquired Size	32768
14	Spectral Size	32768

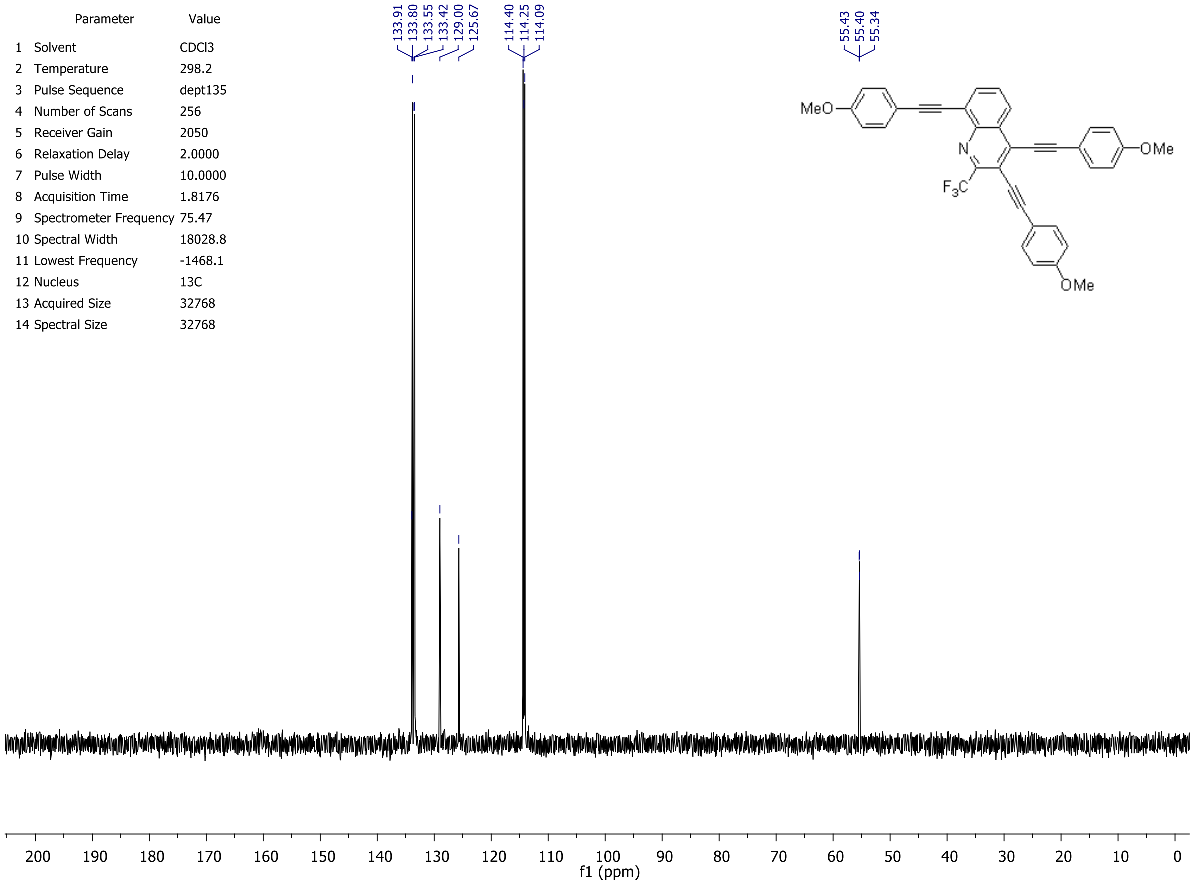

	Parameter	Value
1	Solvent	CDCl3
2	Temperature	298.2
3	Pulse Sequence	zgfhigqn
4	Number of Scans	64
5	Receiver Gain	2050
6	Relaxation Delay	1.0000
7	Pulse Width	10.0000
8	Acquisition Time	0.9787
9	Acquisition Date	2014-06-30T10:04:00
10	Modification Date	2014-06-30T09:04:39
11	Spectrometer Frequency	282.40
12	Spectral Width	66964.3
13	Lowest Frequency	-61722.4
14	Nucleus	19F
15	Acquired Size	65536
16	Spectral Size	131072

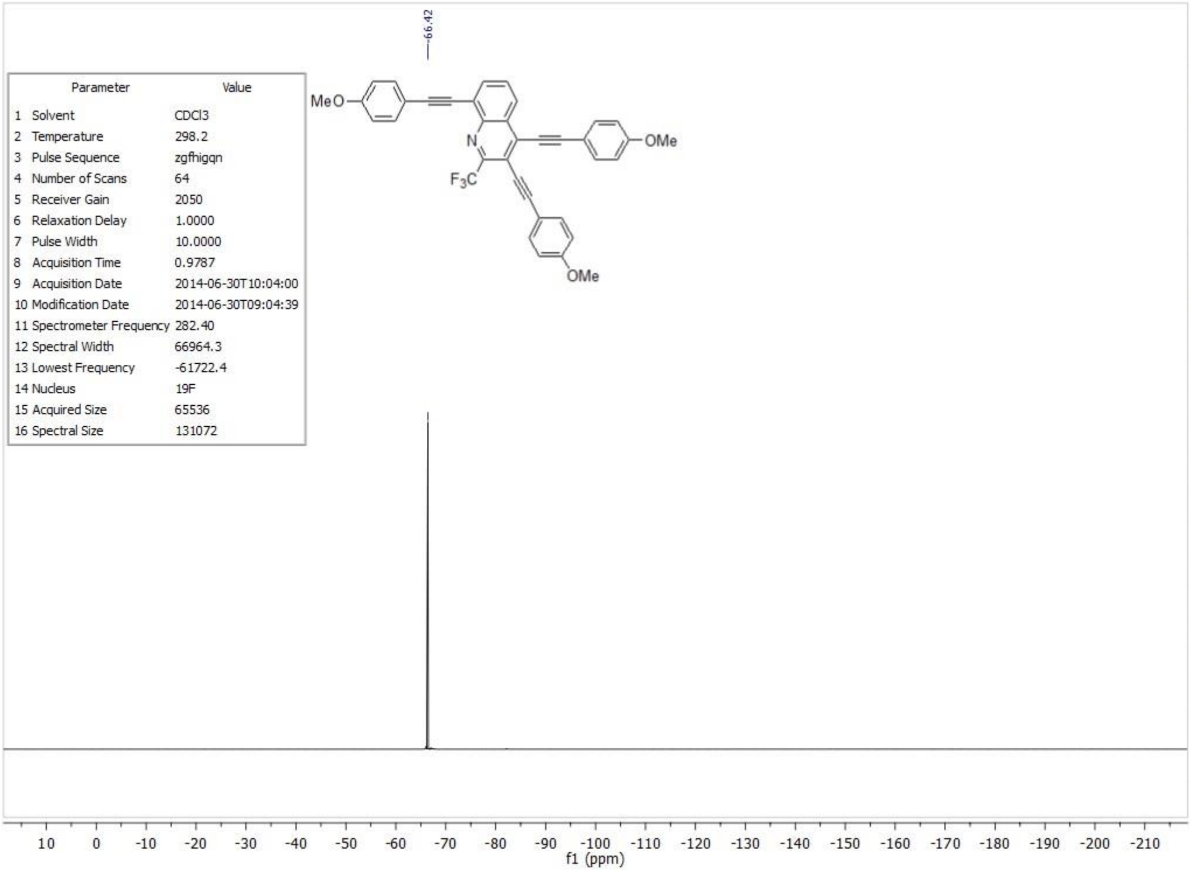

12f - 3,4,8-Tris(3′-thienylethynyl)-2-trifluoromethylquinoline

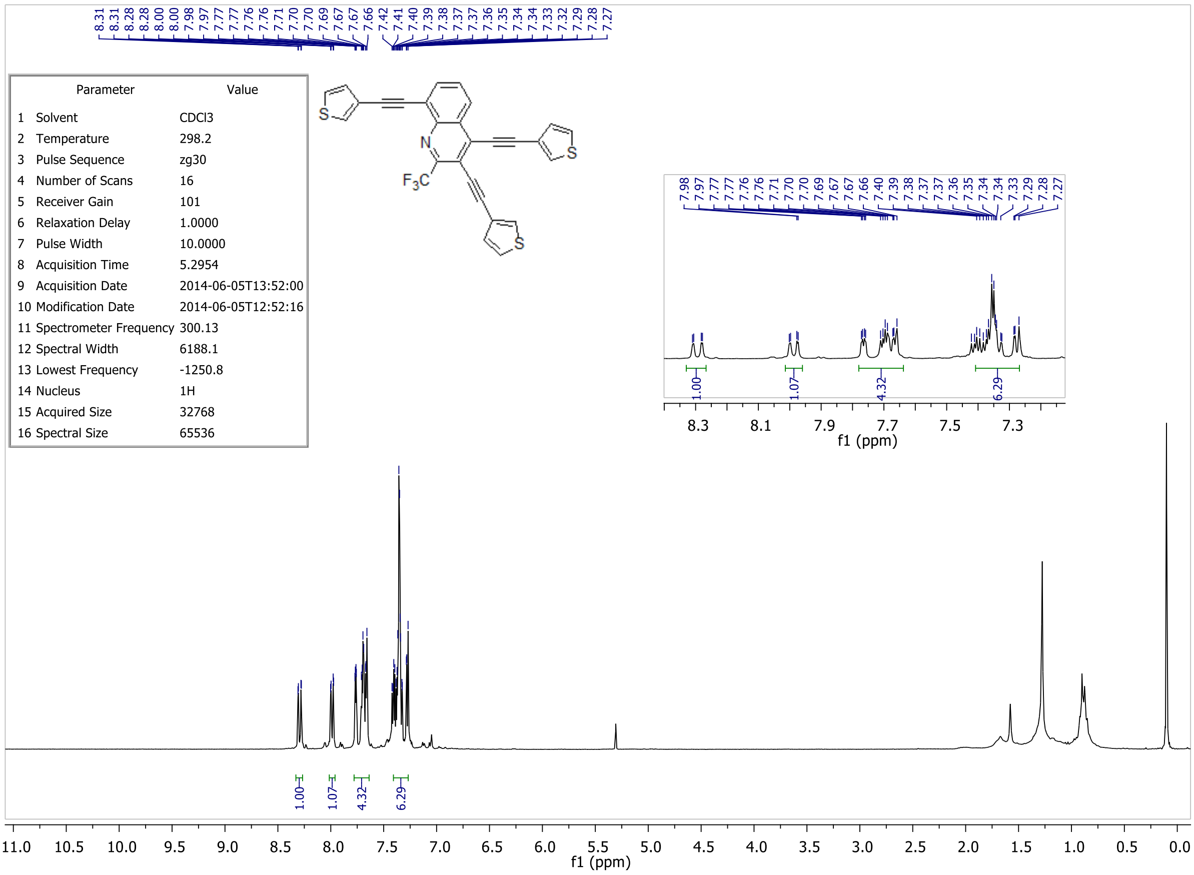

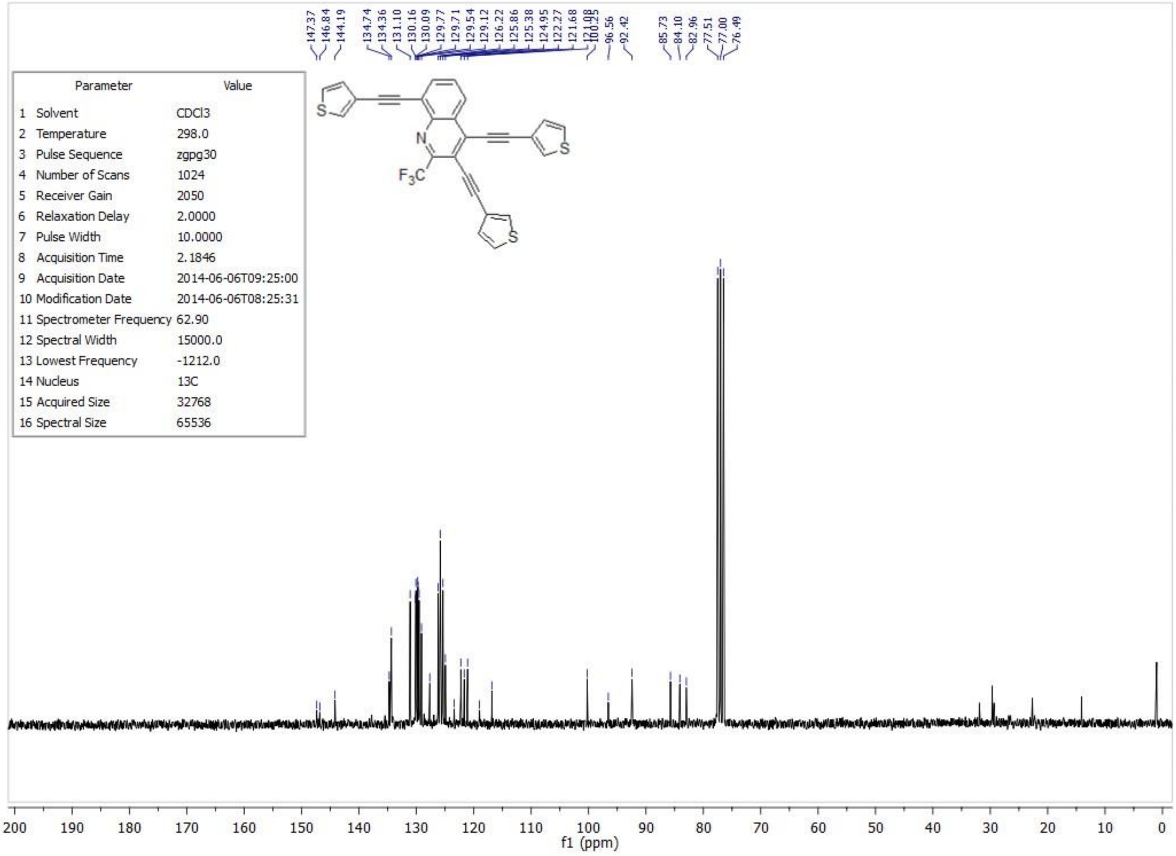

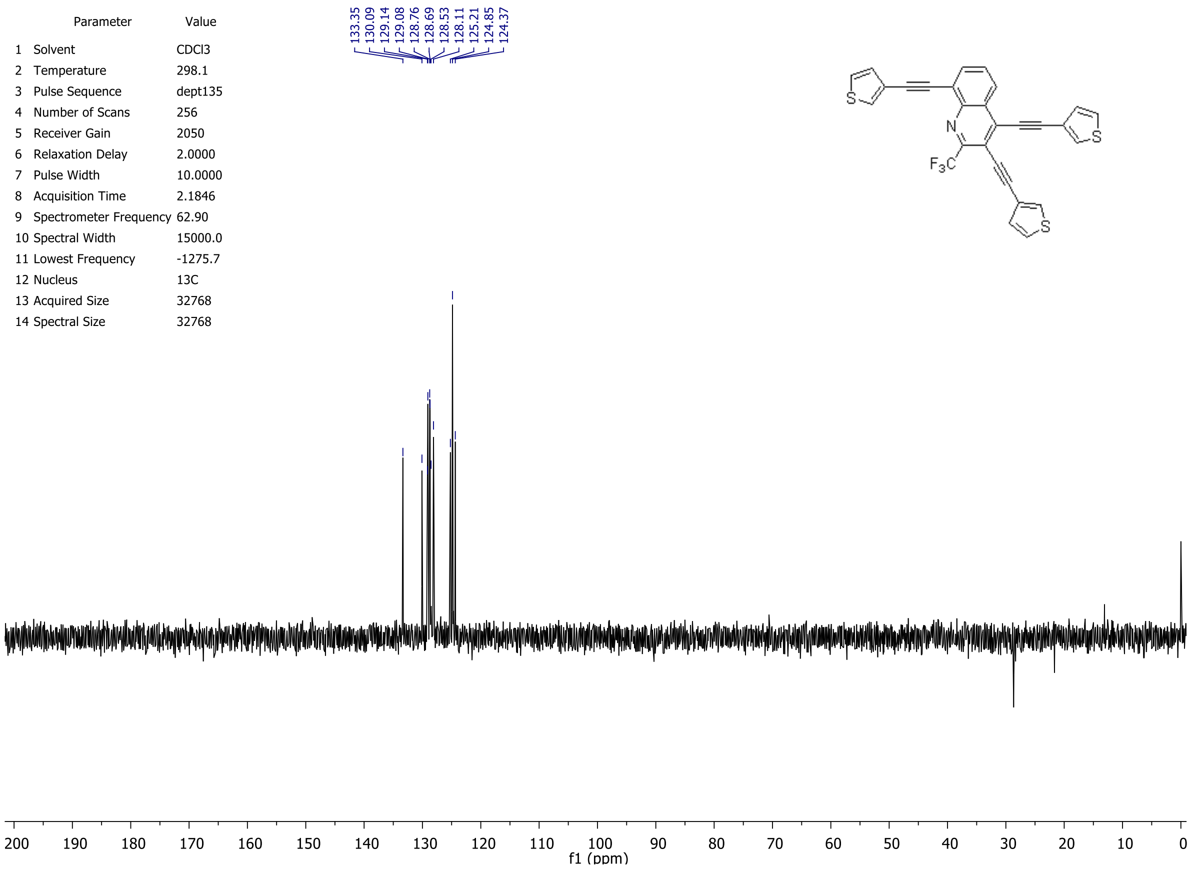

Parameter Value
1 Solvent CDCl3
2 Temperature 298.1
3 Pulse Sequence dept135
4 Number of Scans 256
5 Receiver Gain 2050
6 Relaxation Delay 2.0000
7 Pulse Width 10.0000
8 Acquisition Time 2.1846
9 Spectrometer Frequency 62.90
10 Spectral Width 15000.0
11 Lowest Frequency -1275.7
12 Nucleus 13C
13 Acquired Size 32768
14 Spectral Size 32768
133.35
130.09
129.14
129.08
128.76
128.69
128.53
128.11
125.21
124.85
124.37
S
N
F_3C
S
S
200 190 180 170 160 150 140 130 120 110 100 90 80 70 60 50 40 30 20 10 0
f1 (ppm)

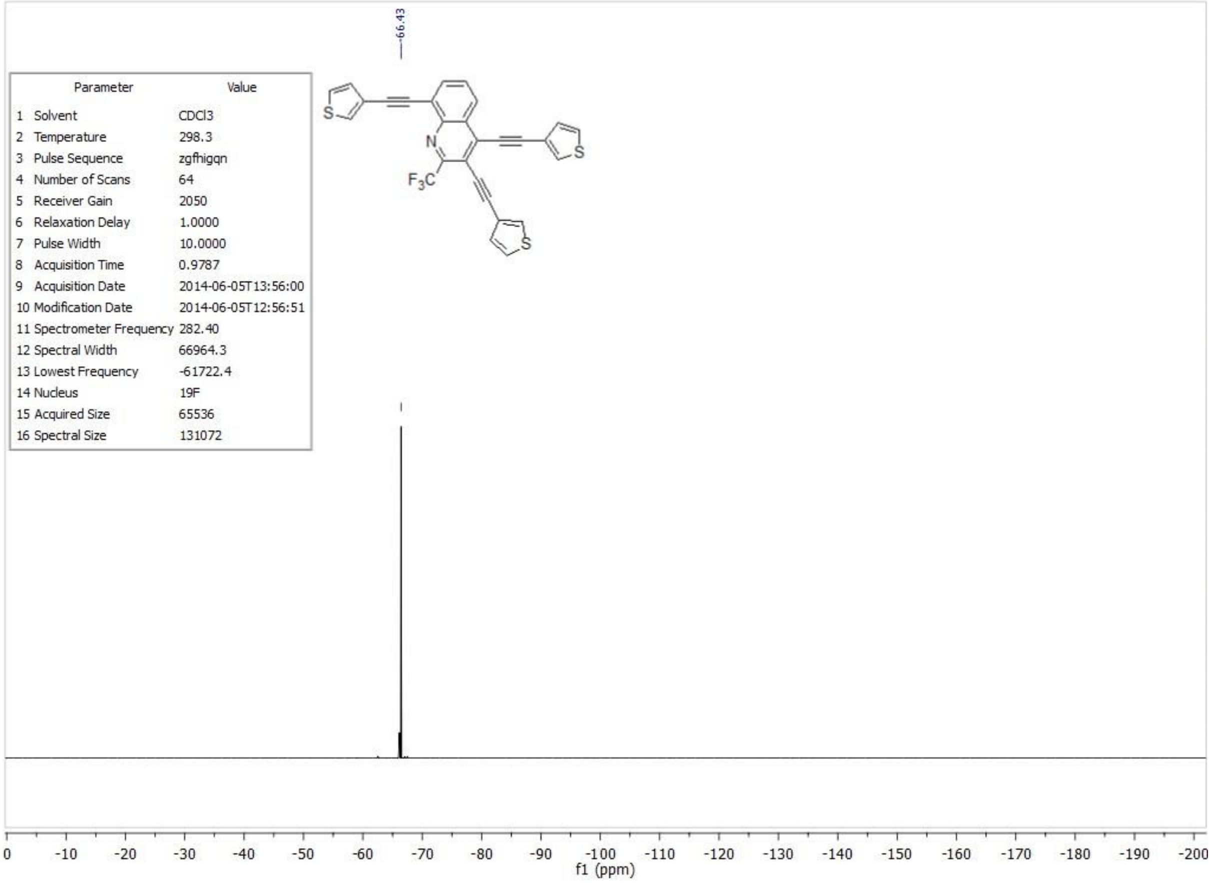

-66.43
Parameter Value
1 Solvent CDCl3
2 Temperature 298.3
3 Pulse Sequence zgfhigqn
4 Number of Scans 64
5 Receiver Gain 2050
6 Relaxation Delay 1.0000
7 Pulse Width 10.0000
8 Acquisition Time 0.9787
9 Acquisition Date 2014-06-05T13:56:00
10 Modification Date 2014-06-05T12:56:51
11 Spectrometer Frequency 282.40
12 Spectral Width 66964.3
13 Lowest Frequency -61722.4
14 Nucleus 19F
15 Acquired Size 65536
16 Spectral Size 131072
S
N
F_3C
S
S
0 -10 -20 -30 -40 -50 -60 -70 -80 -90 -100 -110 -120 -130 -140 -150 -160 -170 -180 -190 -200
f1 (ppm)

12g - 3,4,8-Tris(cyclopropylethynyl)-2-trifluoromethylquinoline

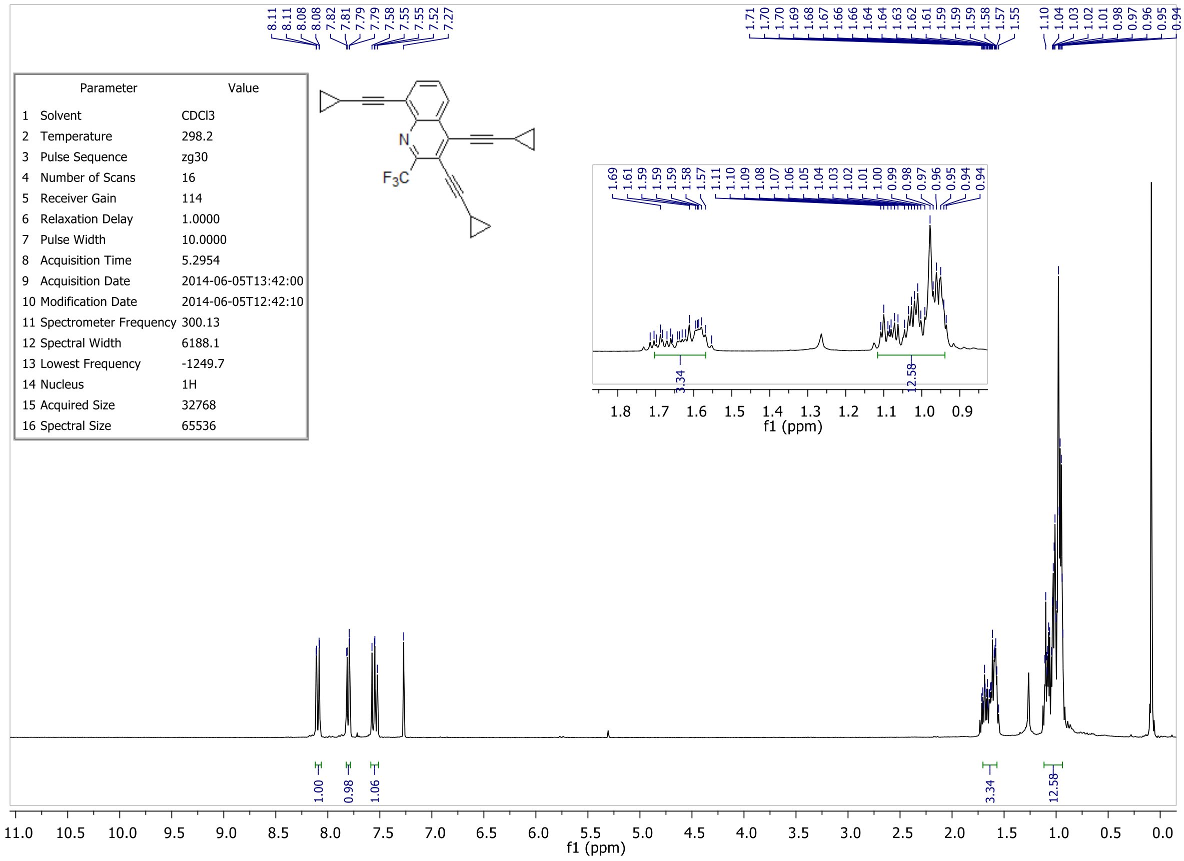

	Parameter	Value
1	Solvent	CDCl3
2	Temperature	298.2
3	Pulse Sequence	zg30
4	Number of Scans	16
5	Receiver Gain	114
6	Relaxation Delay	1.0000
7	Pulse Width	10.0000
8	Acquisition Time	5.2954
9	Acquisition Date	2014-06-05T13:42:00
10	Modification Date	2014-06-05T12:42:10
11	Spectrometer Frequency	300.13
12	Spectral Width	6188.1
13	Lowest Frequency	-1249.7
14	Nucleus	1H
15	Acquired Size	32768
16	Spectral Size	65536

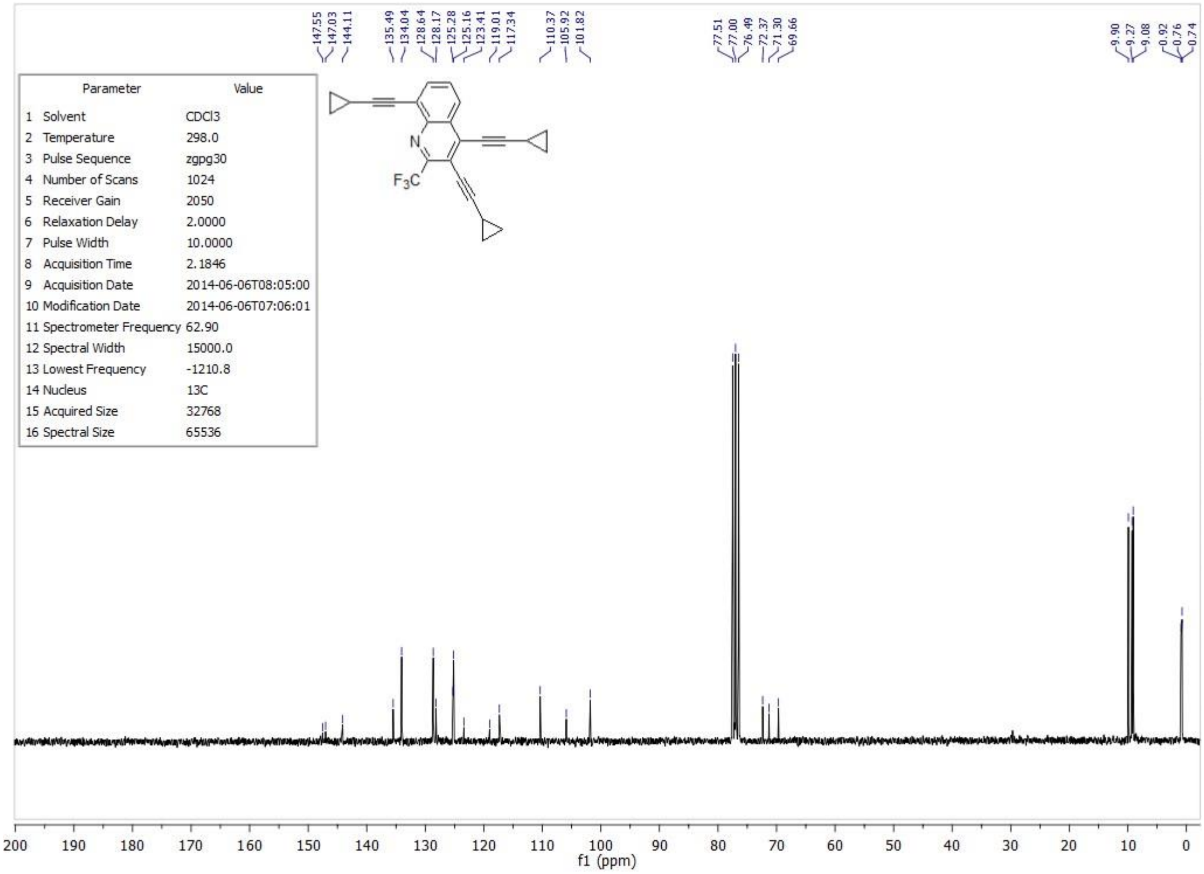

	Parameter	Value
1	Solvent	CDCl3
2	Temperature	298.0
3	Pulse Sequence	zgpg30
4	Number of Scans	1024
5	Receiver Gain	2050
6	Relaxation Delay	2.0000
7	Pulse Width	10.0000
8	Acquisition Time	2.1846
9	Acquisition Date	2014-06-06T08:05:00
10	Modification Date	2014-06-06T07:06:01
11	Spectrometer Frequency	62.90
12	Spectral Width	15000.0
13	Lowest Frequency	-1210.8
14	Nucleus	13C
15	Acquired Size	32768
16	Spectral Size	65536

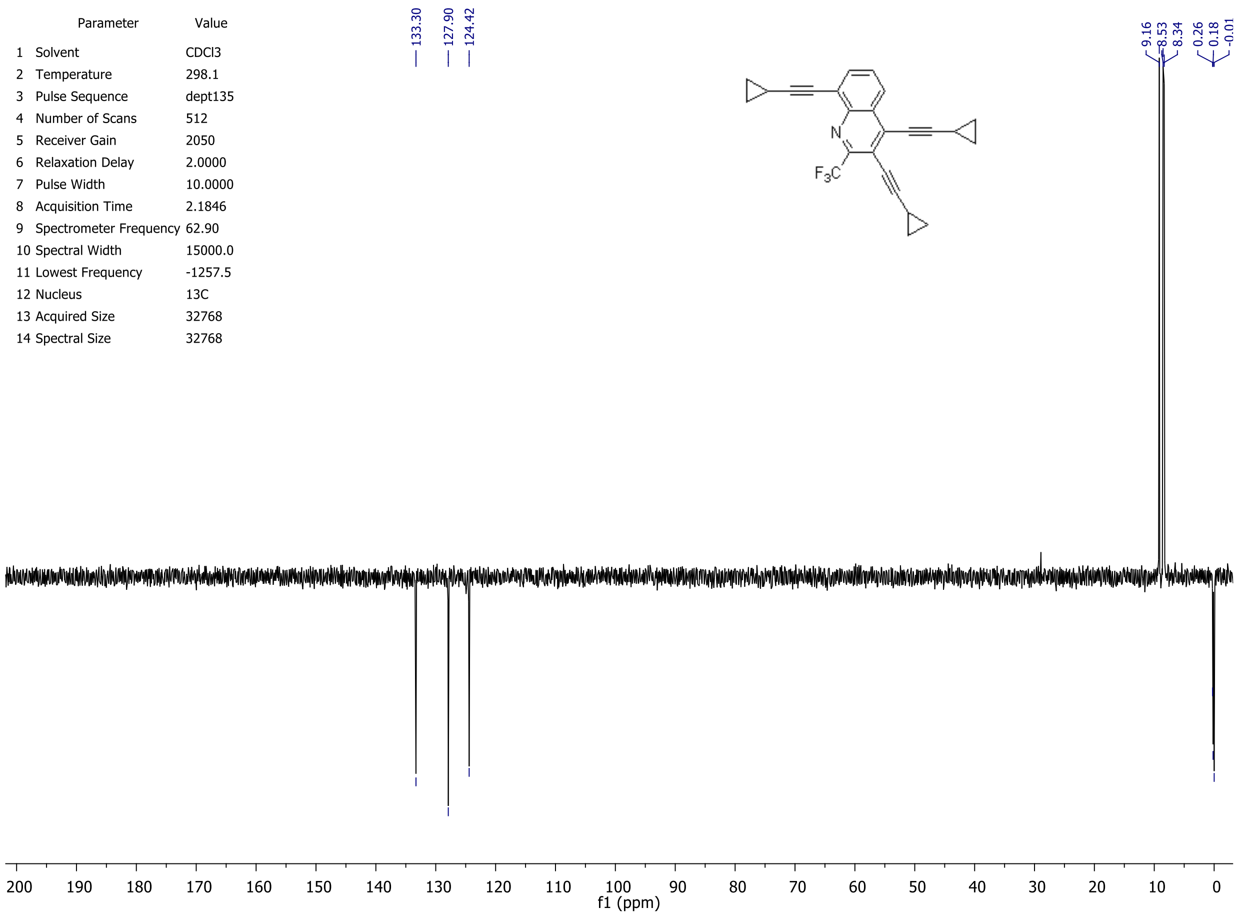

	Parameter	Value
1	Solvent	CDCl3
2	Temperature	298.1
3	Pulse Sequence	dept135
4	Number of Scans	512
5	Receiver Gain	2050
6	Relaxation Delay	2.0000
7	Pulse Width	10.0000
8	Acquisition Time	2.1846
9	Spectrometer Frequency	62.90
10	Spectral Width	15000.0
11	Lowest Frequency	-1257.5
12	Nucleus	13C
13	Acquired Size	32768
14	Spectral Size	32768

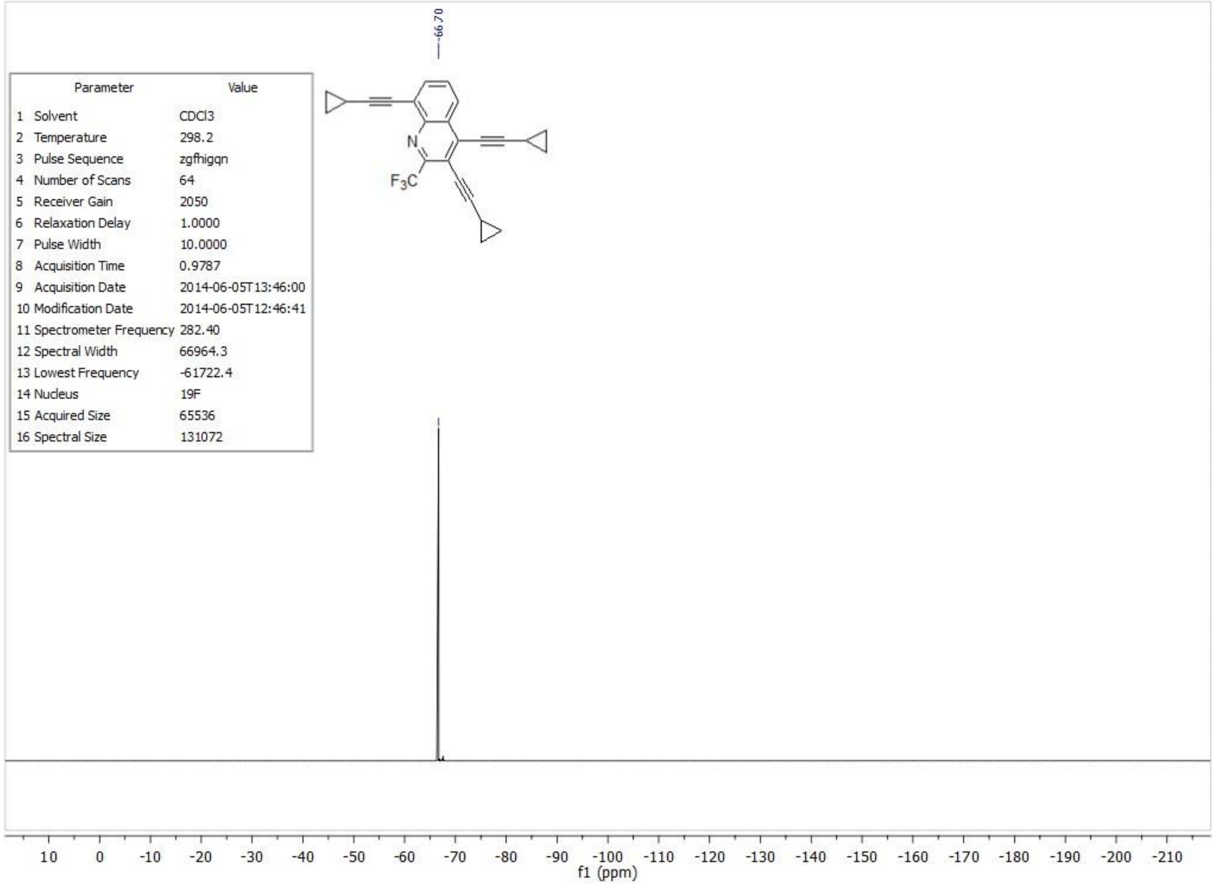

	Parameter	Value
1	Solvent	CDCl3
2	Temperature	298.2
3	Pulse Sequence	zgfhigqn
4	Number of Scans	64
5	Receiver Gain	2050
6	Relaxation Delay	1.0000
7	Pulse Width	10.0000
8	Acquisition Time	0.9787
9	Acquisition Date	2014-06-05T13:46:00
10	Modification Date	2014-06-05T12:46:41
11	Spectrometer Frequency	282.40
12	Spectral Width	66964.3
13	Lowest Frequency	-61722.4
14	Nucleus	19F
15	Acquired Size	65536
16	Spectral Size	131072

[i] Marull, M.; Schlosser, M. *European Journal of Organic Chemistry* **2003,** *2003* (8), 1576–1588. doi:10.1002/ejoc.200390217

[ii] Da Silva, E. T.; Andrade, G. F. de; Da Araújo, A. S.; Lourenço, M. C. S.; Souza, M. V. N. de. *European journal of pharmaceutical sciences : official journal of the European Federation for Pharmaceutical Sciences* **2021,** *157*, 105596. doi:10.1016/j.ejps.2020.105596

[iii] Upadhayaya, R. S.; Dixit, S. S.; Földesi, A.; Chattopadhyaya, J. *Bioorganic & Medicinal Chemistry Letters* **2013,** *23* (9), 2750–2758. doi:10.1016/j.bmcl.2013.02.054
